# Zinc Promoted Cross‐Electrophile Sulfonylation to Access Alkyl–Alkyl Sulfones

**DOI:** 10.1002/advs.202406228

**Published:** 2024-07-04

**Authors:** Zhuochen Wang, Rui Ma, Chang Gu, Xiaoqian He, Haiwei Shi, Ruopeng Bai, Renyi Shi

**Affiliations:** ^1^ School of Chemical Engineering and Technology Xi'an Jiaotong University Xi'an 710049 P. R. China; ^2^ School of Chemistry and Chemical Engineering Chongqing Key Laboratory of Chemical Theory and Mechanism Chongqing University Chongqing 401331 P. R. China; ^3^ NMPA Key Laboratory for Impurity Profile of Chemical Drugs Jiangsu Institute for Food and Drug Control Nanjing 210019 P. R. China

**Keywords:** alkyl–alkyl sulfone, catalyst‐free, cross‐electrophile coupling, organic halides, sulfonylation

## Abstract

The transition metal‐catalyzed multi‐component cross‐electrophile sulfonylation, which incorporates SO_2_ as a linker within organic frameworks, has proven to be a powerful, efficient, and cost‐effective means of synthesizing challenging alkyl–alkyl sulfones. Transition metal catalysts play a crucial role in this method by transferring electrons from reductants to electrophilic organohalides, thereby causing undesirable side reactions such as homocoupling, protodehalogenation, *β*‐hydride elimination, etc. It is worth noting that tertiary alkyl halides have rarely been demonstrated to be compatible with current methods owing to various undesired side reactions. In this work, a zinc‐promoted cross‐electrophile sulfonylation is developed through a radical‐polar crossover pathway. This approach enables the synthesis of various alkyl–alkyl sulfones, including 1°‐1°, 2°‐1°, 3°‐1°, 2°‐2°, and 3°‐2° types, from inexpensive and readily available alkyl halides. Various functional groups are well tolerated in the work, resulting in yields of up to 93%. Additionally, this protocol has been successfully applied to intramolecular sulfonylation and homo‐sulfonylation reactions. The insights gained from this work shall be useful for the further development of cross‐electrophile sulfonylation to access alkyl–alkyl sulfones.

## Graphical abstract

A zinc‐promoted cross‐electrophile sulfonylation through a radical‐polar crossover pathway has been developed. This approach enables the synthesis of various alkyl–alkyl sulfones, including 1°‐1°, 2°‐1°, 3°‐1°, 2°‐2°, and 3°‐2° types, from inexpensive and readily available alkyl halides. Additionally, this protocol has been successfully applied to intramolecular sulfonylation and homo‐sulfonylation reactions.

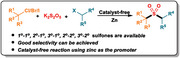

## 1 Introduction

**Figure 1:**
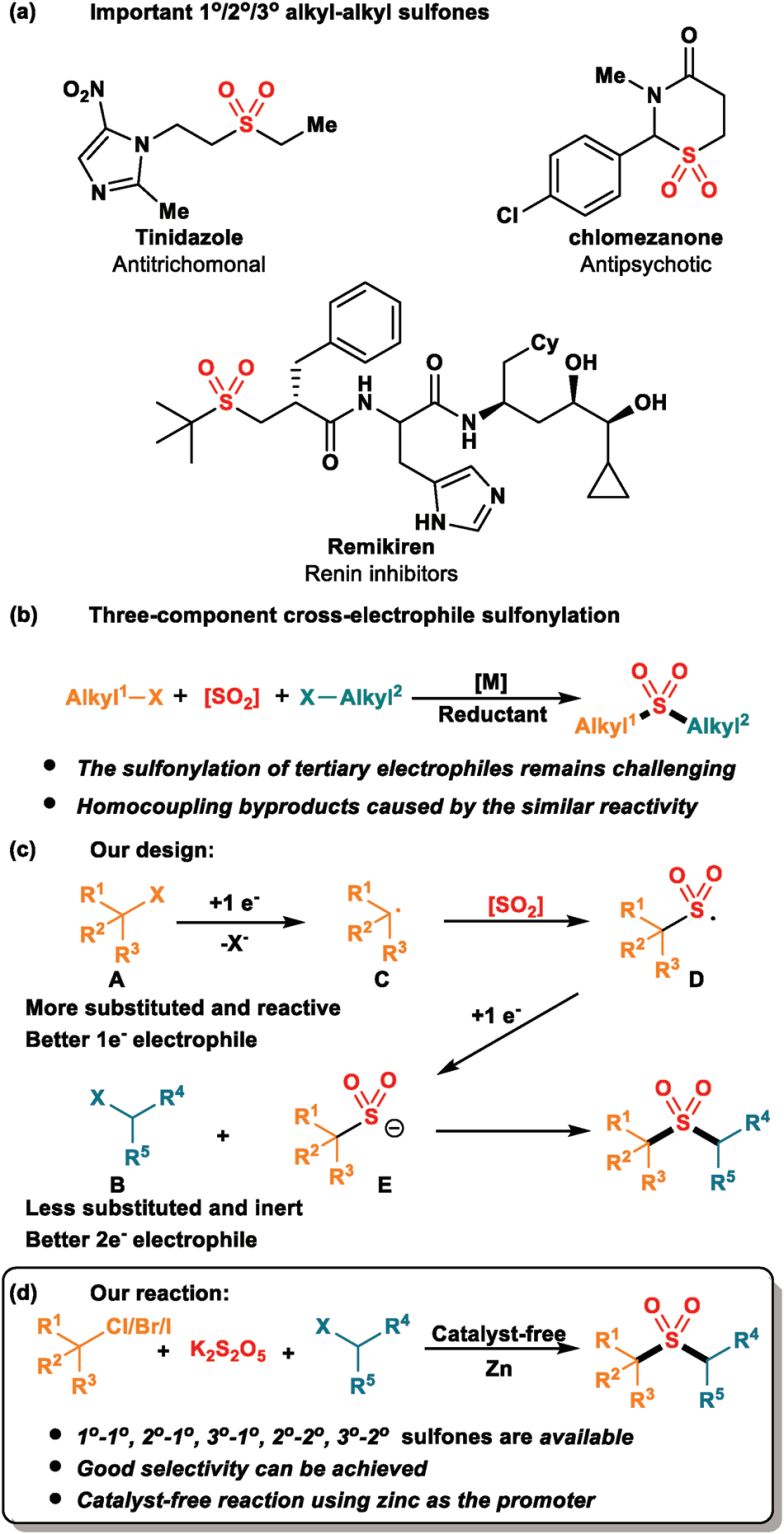
a) Important 1°/2°/3° alkyl–alkyl sulfones. b) Three‐component cross‐electrophile sulfonylation. c) Our design. d) Our reaction.

The chemical structure of sulfone, which has a profound impact on the stability, lipid solubility, and metabolism of molecules, serves as the foundation for numerous naturally occurring biologically active molecules and modern pharmaceutical compounds.^[^ ^1^ ^]^ 1°/2°/3° alkyl–alkyl sulfones are an important class of sulfones that occupy a pivotal position in pharmaceuticals because of their significant influence on the balance between lipid and water solubility.^[^ ^2^ ^]^ **Figure** **1**a depicts a selection of well‐known drugs or pharmacological inhibitors that possess an alkyl–alkyl sulfone structure, including Tinidazole^[^ ^3^ ^]^ (with 1°‐1° sulfone), chlomezanone^[^ ^4^ ^]^ (with intramolecular 2°‐1° sulfone), Remikiren^[^ ^5^ ^]^ (with 3°‐1° sulfone). Over this reason, organic chemists have dedicated considerable efforts over the past decades to developing highly selective, cost‐effective, and efficient synthetic methods for alkyl–alkyl sulfones.^[^ ^6^ ^]^ Traditionally, sulfones are prepared from oxidizing corresponding thioethers and sulfoxides with strong oxidants, resulting in low functional‐group compatibility.^[^ ^7^ ^]^ Alternatively, strategies for sulfone construction from sulfonic, sulfinic, or their derivatives are hampered by the need for complex and multi‐step substrate pre‐synthesis.^[^ ^8^ ^]^ An intensive effort has been made to identify mild and green alternatives for sulfone synthesis that begin with readily available substrate feedstocks.

In recent decades, transition metal‐catalyzed cross‐electrophile coupling (XEC) reactions have emerged as a reliable alternative to traditional cross‐coupling methods for the construction of C─C or C─X bonds.^[^ ^9^ ^]^ This approach involves the coupling of two electrophilic organohalides, facilitated by external reductants, thus circumventing the limitations associated with the use of preformed carbon nucleophiles.^[^ ^10^ ^]^ Consequently, transition metal‐catalyzed multi‐component cross‐electrophile sulfonylation, which incorporates sulfur dioxide as a linker into organic frameworks, has garnered significant interest among researchers due to its atom, step, and oxidation economy (Figure 1b).^[^ ^11^ ^]^ Despite the progress in synthesizing alkyl–alkyl sulfones using this protocol, the reliance on a transition metal catalyst poses inherent limitations. Homocoupling remains a major competing pathway, even in the presence of a large excess of the coupling partner, which has been attributed to the similar reactivity of the catalyst toward different types of alkyl halides. Furthermore, tertiary alkyl halides have rarely been demonstrated to be compatible with current methods due to various undesired side reactions, such as protodehalogenation and elimination. To address these limitations, we hypothesized that a zinc‐promoted radical‐polar crossover pathway could significantly contribute to the synthesis of 1°/2°/3° alkyl–alkyl sulfones (Figure 1c).^[^ ^12^ ^]^ This protocol involves selective single‐electron‐reduction of a more substituted and reactive alkyl halide **A** (alkyl‐I, alkyl‐Br or activated alkyl‐Cl), generating an alkyl radical **C**. Subsequently, the alkyl radical **C** attacks a SO_2_ surrogate, leading to the formation of a sulfonyl radical **D**. Due to the slightly higher electronegativity of sulfur atoms compared to carbon atoms,^[^ ^13^ ^]^ sulfonyl radicals were apt to be reduced to sulfonyl anion **E**. This anion then undergoes chemoselective nucleophilic substitution on a less hindered and inert alkyl halide **B** (Alkyl‐Cl), ultimately yielding the desired product. Herein, we have developed a catalyst‐free, selective cross‐electrophile sulfonylation protocol for the synthesis of alkyl–alkyl sulfones from cheap and readily available primary, secondary, and tertiary alkyl halides (Figure 1d). This method offers a straightforward and efficient approach to constructing a diverse range of sulfone‐containing compounds.

## 2 Results and Discussion

### 2.1 Screening of Reaction Conditions

Initially, we performed the three‐component cross‐electrophile sulfonylation using iodocyclohexane (**1a**) and 1‐chloro‐3‐phenylpropane (**2a**) as the model substrates. After systematic optimization, 92% GC yield of product (**3a**) could be obtained after reacting 24 h at 75 °C with Zn powder (5 equiv.) as a reductant, K_2_S_2_O_5_ (3 equiv.) as an SO_2_ source, NaH_2_PO_4_ (1.5 equiv.) as a base, DMSO 2 mL as a solvent (**Table** **1**, entry 1). The homocoupling by‐products **3a’** and **3a’’** were formed in 3% and 10% yield (relative to **3a**), indicating the high selectivity in our protocol. If we removed Zn or potassium metabisulfite from the reaction system, there were no products (Table 1, entries 2 and 3). 62% yield of the desired product was obtained without NaH_2_PO_4_ (Table 1, entry 4). We hypothesized that the addition of NaH_2_PO_4_ can protonate K_2_S_2_O_5_, thereby facilitating the release of SO_2_. When Mn was utilized instead of Zn as a reductant, a 75% yield of **3a** was achieved (Table 1, entry 5). When replacing K_2_S_2_O_5_ with Na_2_S_2_O_5_ or Na_2_S_2_O_4_, yields were 88% and 36%, respectively (Table 1, entries 6 and 7). The use of DMA instead of DMSO as a solvent led to 24% yield (Table 1, entry 8). Other solvents like toluene, cyclohexane, and CH_2_Cl_2_ were tested, and only a trace amount of product was found (Table 1, entry 9 and SI). Lowering or increasing the reaction temperature reduced the yields slightly (Table 1, entries 10 and 11). No desired product was observed at room temperature (Table 1, entry 12).

**Table 1:** Summary of the effects of reaction parameters and conditions on the reaction efficiency.

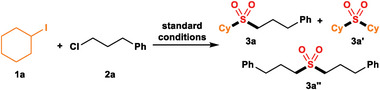				
Entry	Variation from standard conditions^a)^	3a%^b)^	3a’%	3a’’%
1	none	92(82^c)^)	3	10
2	No Zn	n.d	n.d	n.d
3	No K_2_S_2_O_5_	n.d	n.d	n.d
4	No NaH_2_PO_4_	62	4	37
5	Mn instead of Zn	75	2	16
6	Na_2_S_2_O_5_ instead of K_2_S_2_O_5_	88	2	15
7	Na_2_S_2_O_4_ instead of K_2_S_2_O_5_	36	3	48
8	DMA instead of DMSO	24	1	19
9	CH_2_Cl_2_ instead of DMSO	trace	trace	trace
10	60 °C instead of 75 °C	57	1	7
11	120 °C instead of 75 °C	47	5	63
12	RT instead of 75 °C	n.d	n.d	n.d

^a)^ The optimized reaction conditions were **1a** (0.2 mmol), **2a** (2 equiv.), Zn powder (5 equiv.), K_2_S_2_O_5_ (3 equiv.), NaH_2_PO_4_ (1.5 equiv.), and DMSO 2 mL, at 75 °C and under a nitrogen atmosphere, 24 h;

^b)^ Yields determined by GC using naphthalene as an internal standard;

^c)^ Isolated yield.

### 2.2 Substrate Scope Evaluation

**Scheme 1:**
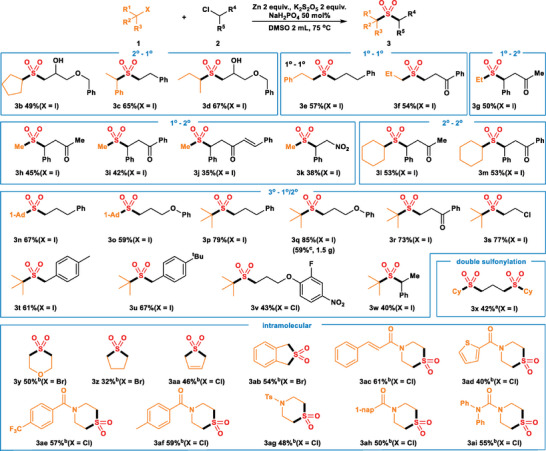
Catalyst‐free cross‐electrophile sulfonylation of **1** and **2**. The reaction conditions were **1** (0.4 mmol), Zn powder (2 equiv.), K_2_S_2_O_5_ (2 equiv.), NaH_2_PO_4_ (50 mol%), DMSO (1.5 mL), 75 °C, 2 h, then add **2** (0.2 mmol), DMSO (0.5 mL), 75 °C, 22 h (General Procedure A). Isolated yields. *^a^* Variation: **1** (4 equiv.), Zn powder (4 equiv.), K_2_S_2_O_5_ (4 equiv.), 48 h. *^b^* Variation: Add NaI (50 mol%), DMSO 4 mL, 48 h. *^c^* Variation: **1** (2 equiv.), Zn powder (2 equiv.), K_2_S_2_O_5_ (2 equiv.), NaH_2_PO_4_ (50 mol%), DMSO (7.5 mL), 75 °C, 6 h, then add **2** (10 mmol), DMSO (2.5 mL), 75 °C, 48 h.

The optimized reaction conditions were employed to synthesize a wide range of unsymmetric dialkyl sulfones (**Scheme** **1**). Various secondary (cyclic and acyclic) and primary alkyl iodides were suitable reactants (**3b**‐**3f**). When the reaction between bromocyclohexane and 1‐chloro‐3‐phenylpropane was carried out, only 23% yield of **3a** was achieved. To our surprise, hydroxyl groups were compatible in this reaction (**3b** and **3d**). 54% yield of 1°−1° sulfone could be achieved in our protocol utilizing primary alkyl iodide and primary alkyl chloride (**3f**). Methyl sulfones and ethyl sulfones, which widely exist in pharmaceuticals, could be synthesized from liquid methyl iodide and ethyl iodide through our method in moderate yields (**3g**‐**3k**). When secondary alkyl iodides and secondary alkyl chloride were used as the substrates, 2°−2° sulfones were achieved in 53% yield (**3l** and **3m**). Tertiary alkyl iodide which typically poses challenges in serving as viable coupling partners in nickel‐catalyzed due to the steric hindrance are compatible in this reaction (**3n**‐**3v**), affording 43% to 85% yields. Alkyl groups containing ester, ketone, chlorine, fluorine, and nitro groups were well tolerated (**3q**, **3r**, **3s,** and **3v**). To our delight, 40% yield of 3°−2° sulfone (1‐(*tert*‐butylsulfonyl)ethyl)benzene could be synthesized from *tert*‐butyl iodide and (1‐chloroethyl)benzene (**3w**). When a substrate containing two iodo groups, 1, 3‐diiodopropane, was used, the double sulfonylation product (**3x**) was obtained in 42% yield. When bromocyclohexane, an alkyl bromide, was reacted was used to reacted with 1‐chloro‐3‐phenylpropane, only 23% yield of **3a** was achieved, indicating the less reactivity of alkyl bromide. While the reaction between chlorocyclohexane and 1‐chloro‐3‐phenylpropane did not yield any product. Furthermore, this reaction was not only used to access intermolecular sulfones but also applicable to the intramolecular synthesis of cyclic sulfones (**3y**–**3ai**). Functional groups that exhibit good reactivity and are well tolerated include ether (**3y**), olefin (**3aa**), *o*‐xylene (**3ab**), cinnamamide (**3ac**), thiophene (**3ad**), trifluoromethyl (**3ae**), tosyl (**3ag**), naphthalene (**3ah**), and urea (**3ai**). A scale‐up experiment is carried out to obtain 1.5 g **3q** in 59% yield, demonstrating the protocol's good scalability.

**Scheme 2:**
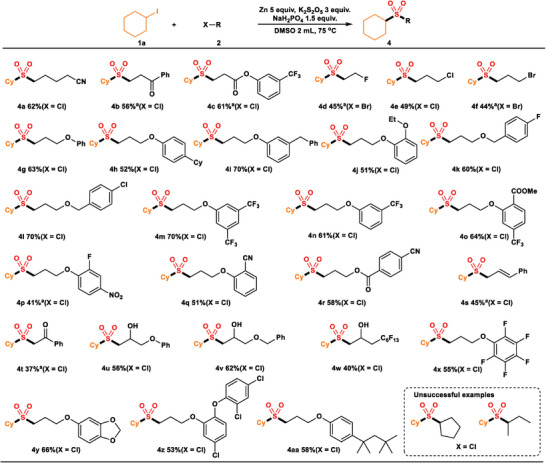
Catalyst‐free cross‐electrophile sulfonylation of **1a** and **2**. The reaction conditions were **1a** (0.2 mmol), **2** (2 equiv.), Zn powder (5 equiv.), K_2_S_2_O_5_ (3 equiv.), NaH_2_PO_4_ (1.5 equiv.), DMSO (2 mL), 75 °C, 24 h (General Procedure B). Isolated yields. *^a^* Using General Procedure A.

Furthermore, we investigated the scope of alkyl chloride **2** and functional group compatibility (**Scheme** **2**). Alkyl chlorides containing cyano (**4a**), ketone (**4b**), ester (**4c**), and ether (**4** **g**) ran the reaction smoothly, resulting in moderate to high yields of the corresponding products. To our surprise, substrates with halogens like ‐F (**4d**), ‐Cl (**4e**) and ‐Br (**4f**) are also suitable substrates, offering opportunities for subsequent functionalization. As substituents at the phenyl ring of the alkyl chlorides, both electron‐donating groups such as cyclohexyl (**4** **h**), benzyl(**4i**), ‐OEt (**4j**) and electron‐withdrawing groups such as ‐F (**4k**), ‐Cl (**4l**), ‐CF_3_ (**4m** and **4n**), ‐COOMe (**4o**), ‐NO_2_ (**4p**), ‐CN (**4q** and **4r**) are well tolerated. Activated alkyl chlorides like (3‐chloroprop‐1‐en‐1‐yl)benzene (**4s**) and 2‐chloro‐1‐phenylethan‐1‐one compound (**4t**) are compatible substrates. Alkyl chlorides substituted with hydroxyl group can also be well tolerated (**4u** and **4v**). When polyfluorinated substances (**4w** and **4x**) were used as the reactants, moderate yields were obtained. The derivatives of biologically active molecules like **4y**, **4z** and **4aa** produced the desired products in 66%, 53% and 58% yields, respectively. The unactivated secondary chloride compounds are incompatible with this reaction system due to their low reactivity and significant steric hindrance.

**Scheme 3:**
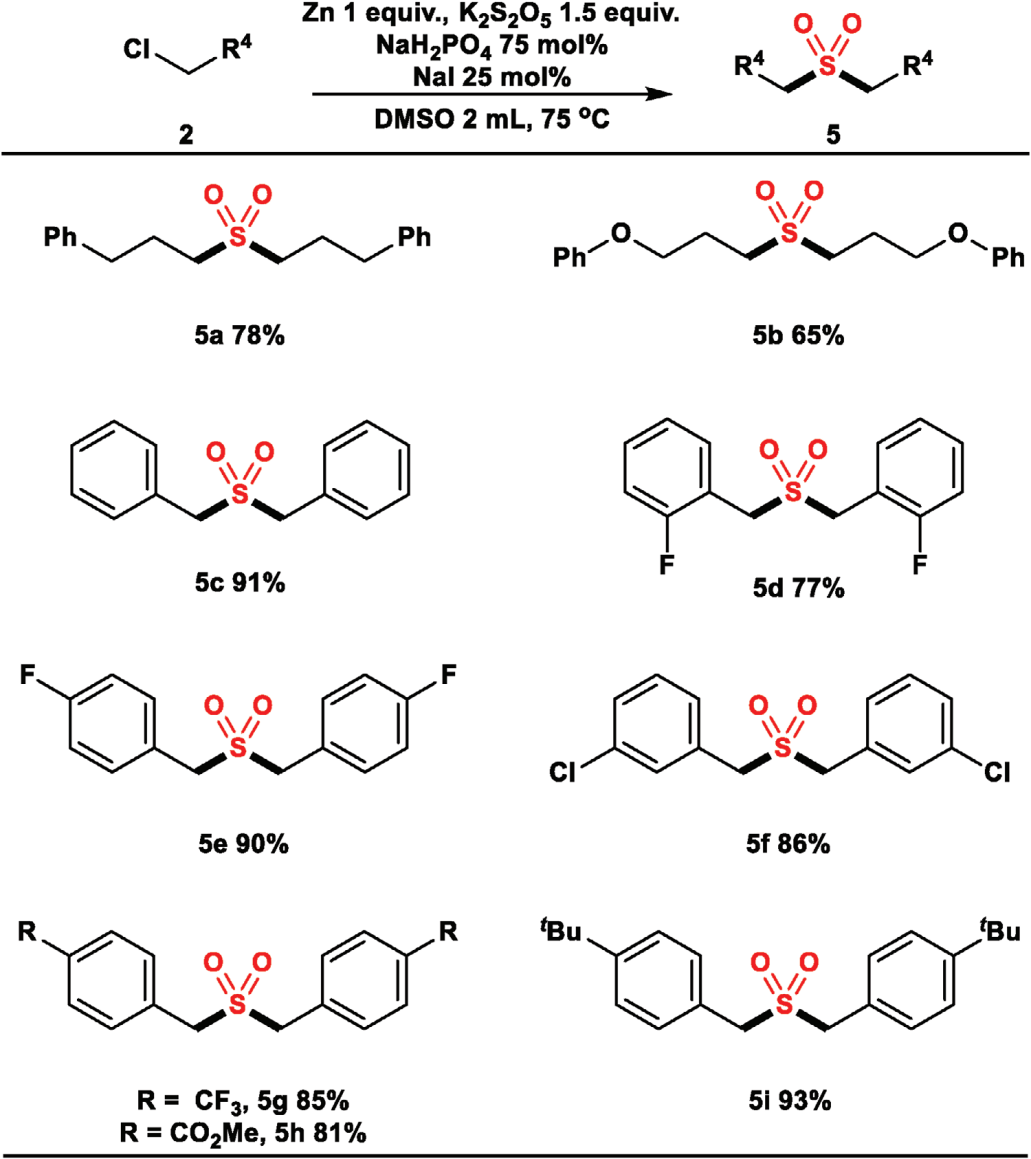
Catalyst‐free homo‐electrophile sulfonylation. The reaction conditions were **2** (0.4 mmol), Zn powder (1 equiv.), K_2_S_2_O_5_ (1.5 equiv.), NaH_2_PO_4_ (75 mol%), NaI (25 mol%), DMSO (2 mL), 75 °C, 24 h (General Procedure C). Isolated yields.

By using only one alkyl halide as a coupling partner, the above method can be adapted for the synthesis of symmetric dialkyl sulfones (**Scheme** **3**), which are less accessible using previously reported methods. Both alkyl chlorides and benzyl chlorides could be used (**5a** – **5i**). Various functional groups such as ether (**5b**), fluoro (**5d** and **5e**), chloro (**5f**), trifluoromethyl (**5** **g**), ester (**5** **h**), and *tert*‐butyl (**5i**) were well tolerated.

### 2.3 Mechanistic Investigation

**Figure 2:**
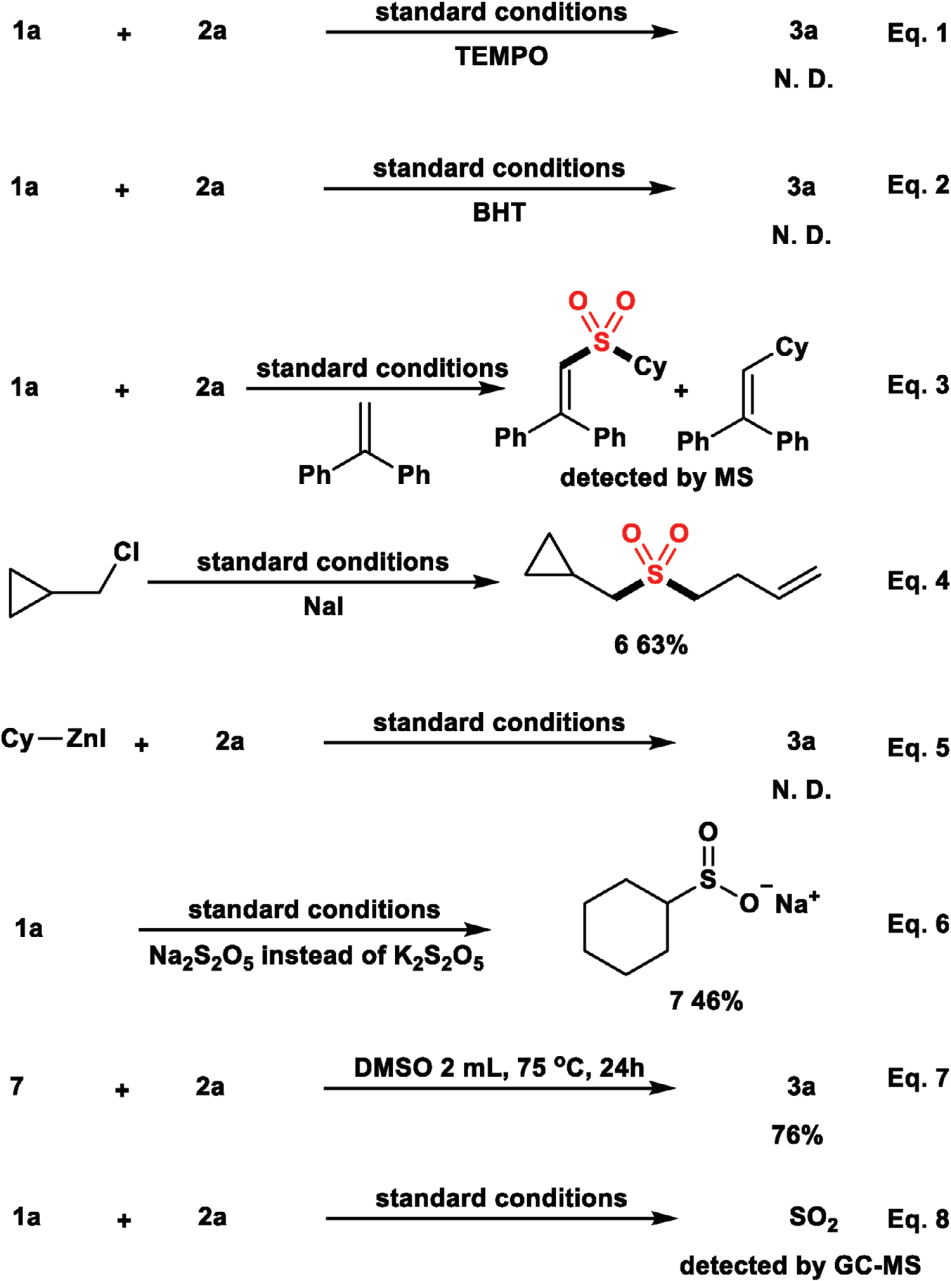
Control experiments.

To get a better understanding about the mechanism of this multicomponent reductive cross‐coupling reaction, several control experiments were carried out (**Figure** **2**). When 2,2,6,6‐tetramethylpiperidine‐1‐oxyl (TEMPO) or 2,6‐di‐*tert*‐butyl‐4‐methylphenol (BHT) were added to the system, no desired sulfone product was detected (Figure 2; Equations S1 and S2, Supporting Information). Moreover, the sulfonyl radical and cyclohexyl radical was trapped by the addition of the radical trapping reagent 1,1‐diphenylethylene to the reaction (Figure 2; Equation S3, Supporting Information). All these experiments indicates that cyclohexyl radical and sulfonyl radical might be involved in this mechanism. Subsequently, a radical clock experiment involving (chloromethyl)cyclopropane was conducted under the standard conditions (Figure 2; Equation S4, Supporting Information). Cyclopropane‐opened product **6** was generated in 63% isolated yield, indicating that an alkyl radical intermediate might be generated from the alkyl iodide. No target product was detected with organic zinc reagent instead of alkyl iodine as the reactant (Figure 2; Equation S5, Supporting Information), excluding the intermediacy of organozinc reagents. In our standard reaction conditions, cyclohexyl sulfonate (**7**) can be isolated after the reaction with only **1a** as the reactant (Figure 2; Equation S6, Supporting Information). When we employed cyclohexyl sulfonate (**7**) and **2a** as the reactants in DMSO at 75 °C for 24 h, the corresponding sulfone product was obtained in 76% yield, showing that alkyl sulfonate might be the key intermediate (Figure 2; Equation S7, Supporting Information).

**Figure 3:**
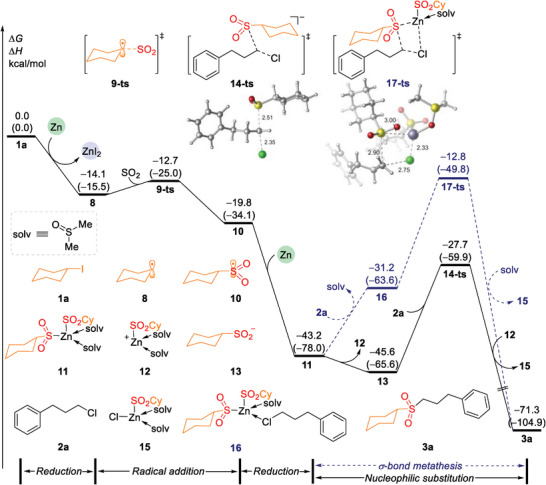
Free energy profiles of catalyst‐free cross‐electrophile sulfonylation process. Calculations were performed using Gaussian 16 at the M06‐L/6‐311 + G(d,p)‐SDD/SMD(DMSO)//B3LYP‐D3(BJ)/6‐31G(d)‐LANL2DZ level of theory. The bond lengths are shown in angstroms (Å). The Zn(II)I_2_ used in DFT calculations is Zn(II)I_2_‐complex.

To gain a deeper understanding of the reaction mechanism, density functional theory (DFT) calculations were employed to the catalyst‐free cross‐electrophile sulfonylation. As shown in **Figure** **3**, iodocyclohexane **1a** was chosen as the starting point of the cycle, which was reduced by Zn to generate cyclohexyl radical **8** (see the Supporting Information for further details). This process is exergonic, with an energy release of 14.1 kcal mol^−1^. The subsequent radical addition of **8** to sulfur dioxide gives cyclohexylsulfonyl radical intermediate **10** via transition state **9‐ts**, which is endergonic by 5.7 kcal mol^−1^. The energy barrier for this step is only 1.4 kcal mol^−1^, indicating that radical addition step can easily occur. Following this, 2^nd^ reduction of intermediate **10** by zinc irreversible generates bis(cyclohexylsulfonyl)zinc(II) intermediate **11**, which releases 23.4 kcal mol^−1^ of energy. Subsequently, the dissociation of the cyclohexylsulfonyl‐zinc(II) cationic intermediate **12** leads to the formation of the cyclohexylsulfonyl anion **13**. Finally, nucleophilic substitution takes place between 1‐chloro‐3‐phenylpropane **2a** and intermediate **13** via transition state **14‐ts**. This step has an energy barrier of 17.9 kcal mol^−1^ and leads to the formation of the final product (3‐(cyclohexylsulfonyl)propyl)benzene **3a**. An alternative pathway involves ligand exchange of intermediate **11** with **2a**, resulting in the formation of intermediate **16**. This step is endergonic 12.0 kcal mol^−1^. Subsequently, σ‐bond metathesis of intermediate **16** occurs via transition state **17‐ts** with an energy barrier of high 30.4 kcal mol^−1^ to product (3‐(cyclohexylsulfonyl)propyl)benzene **3a**. This step involving the cleavage of C─Cl bond and the formation of S─C bond. The difference of energy barriers of the transition states **14‐ts** and **17‐ts** is 14.9 kcal mol^−1^, indicating that nucleophilic substitution of **2a** by intermediate **13** is favorable pathway.

**Figure 4:**
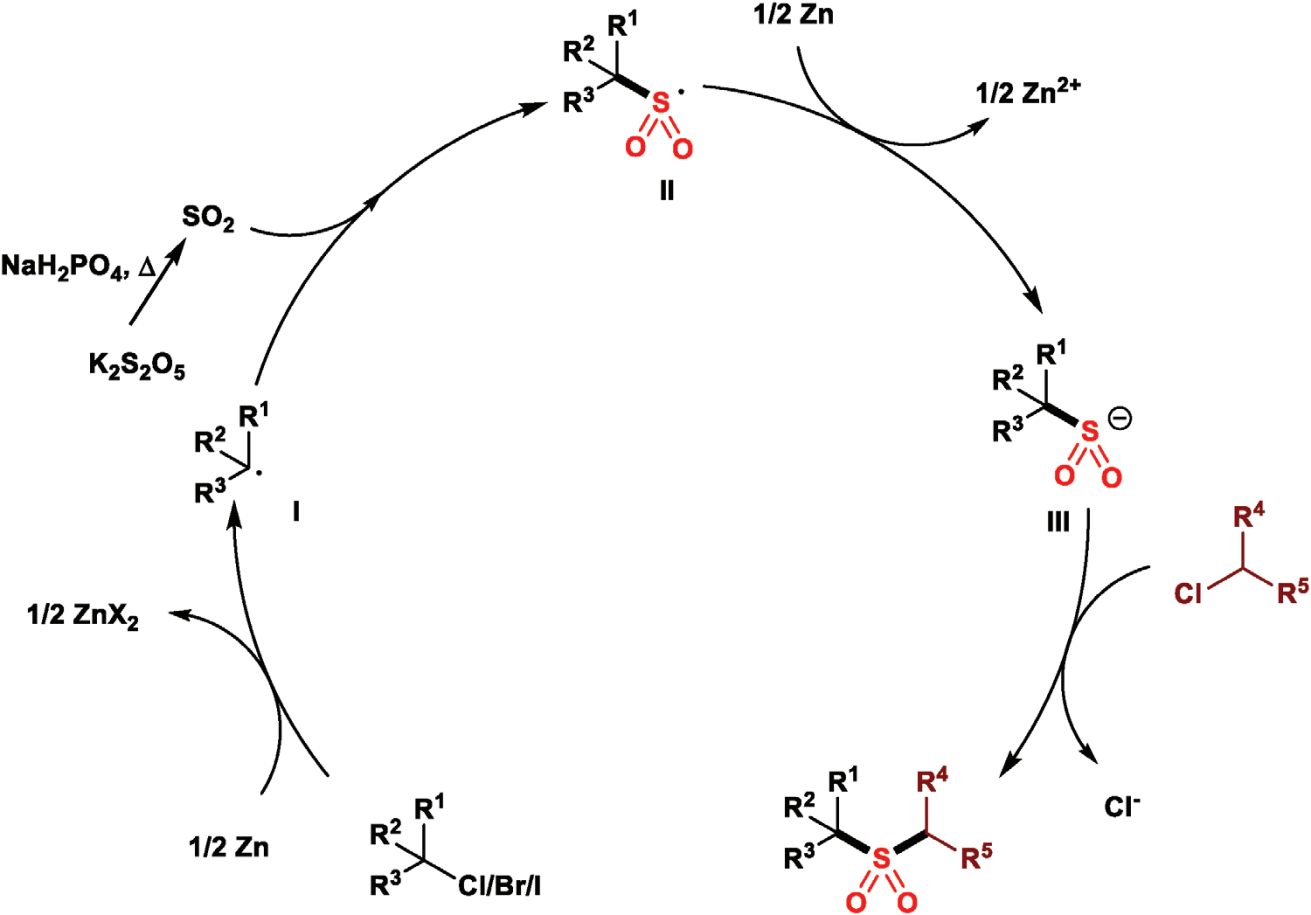
Proposed mechanism.

Based on our previous work^[^ ^12c^ ^]^ and the results described above, a plausible mechanism is proposed as shown in **Figure** **4**. Initially, the steric hindered but active alkyl halide is reduced by Zn to generate alkyl radical **I**. Subsequently, the reaction of the alkyl radical with SO_2_, derived from metabisulfite with the help of NaH_2_PO_4_, yields the sulfonyl radical **II**, which is then reduced by Zn to produce the sulfonyl anion **III**. A subsequent bi‐molecular substitution between less steric hindered alkyl chloride and sulfonyl anion gives the desired sulfone product.

## 3 Conclusion

In conclusion, we have developed a zinc‐promoted cross‐electrophile sulfonylation utilizing readily available alkyl halides and K_2_S_2_O_5_ as reactants. 1°−1°, 2°−1°, 3°−1°, 2°−2°, 3°−2° alkyl–alkyl sulfones could be synthesized through this protocol. Additionally, this protocol has been applied to intramolecular sulfonylation and homo‐sulfonylation reactions. High chemo‐selectivity can be achieved through regulating the steric hindrance and electron transfer between zinc and alkyl halide. A range of functional groups are well‐tolerated in our system, enabling the synthesis of sulfones with up to 93% yields. Mechanistic investigation indicates that the electron transfer between alkyl halides and zinc generates an alkyl radical, followed by SO_2_ insertion to form the sulfonyl radical. This method would significantly contribute to the development of easy and cost‐effective synthetic approaches for sulfones, which are prevalent intermediates and bioactive compounds.

## 4 Experimental Section

### General Procedure A for Cross‐Electrophile Sulfonylation

An oven‐dried 50 mL Schlenk tube equipped with a Teflon‐coated magnetic stir bar was sequentially charged with Zn powder (0.4 mmol, 2 equiv.), K_2_S_2_O_5_ (0.4 mmol, 2 equiv.), NaH_2_PO_4_ (0.1 mmol, 50 mol%) in the glovebox. Then 1.5 mL DMSO, alkyl halides **1** (0.4 mmol, 2 equiv.) were added into the tube in turn. All these procedures were conducted in the glovebox. The vial was sealed with a rubber stopper and removed from the glove box. Then the vial was placed on the heating‐base and reacted at 75 °C for 2 h. After which time the alkyl chloride **2** (0.2 mmol) was dissolved into 0.5 mL of DMSO and added to the Schlenk tube with a needle. Then the vial was placed on the heating‐base and reacted at 75 °C for 22 h. After which time the vial was removed from the heating source, and the product was extracted from the crude reaction mixture with ethyl acetate (3 × 10 mL). The organic layers were combined and washed with brine (30 mL). Dried over Na_2_SO_4_, filtered, and concentrated under reduced pressure. The crude product residue was purified by preparative TLC using a solvent mixture (EtOAc, petroleum ether) as an eluent to afford the purified product.

## Conflict of Interest

The authors declare no conflict of interest.

## Supporting information

Supporting Information

## Data Availability

The data that support the findings of this study are available in the supplementary material of this article.
