# Supplementary material for: Zinc Promoted Cross‐Electrophile Sulfonylation to Access Alkyl–Alkyl Sulfones
Source: Adv Sci (Weinh). 2024 Jul 4;11(32):2406228. doi: 10.1002/advs.202406228 (PMC11347995; doi:10.1002/advs.202406228)
Supplement: Supplementary file 1 — Supporting Information [file ADVS-11-2406228-s001.pdf]

ADVANCED SCIENCE Open Access

Supporting Information

for *Adv. Sci.*, DOI 10.1002/advs.202406228

Zinc Promoted Cross-Electrophile Sulfonylation to Access Alkyl–Alkyl Sulfones

Zhuochen Wang, Rui Ma, Chang Gu, Xiaoqian He, Haiwei Shi, Ruopeng Bai* and Renyi Shi**

Supporting Information

Zinc Promoted Cross-Electrophile Sulfonylation to Access Alkyl–Alkyl Sulfones

Zhuochen Wang[a], Rui Ma[a], Chang Gu[a], Xiaoqian He[b], Haiwei Shi [c], Ruopeng Bai*[b] and Renyi Shi*[a]*

1. General Information S2

2. Preparation of Alkyl Chlorides S2

3. General Procedure for Cross-Electrophile Sulfonylation S3

4. Summary of the effects of reaction parameters and conditions on the reaction efficiency S5

5. Control Experiments S8

6. Scale up reaction S12

7. Computational Details S13

8. Analytical Data of Substrates and Products S45

9. NMR Spectra of Substrates and Products S71

10. References S151

General Information

Unless otherwise noted, all chemicals used in the preparations of starting materials and in the Catalyst-Free Cross-Electrophile Sulfonylation were commercially available and were used as received without further purifications. All catalytic reactions were carried out under nitrogen in Schlenk tubes. Preparative Thin Layer Chromatography (PTLC) was performed using glass plates from Shanxi Nuotai Biotechnology Co., LTD. The eluents for column chromatography and PTLC were presented as ratios of solvent volumes. Nuclear Magnetic Resonance spectra were recorded on a Bruker 400 MHz (101 MHz and 376 MHz for ^{13}C and ^{19}F, respectively) instruments at ambient temperature. All ^{1}H NMR spectra were measured in part per million (ppm) relative to the signals of tetramethylsilane (TMS, 0.00 ppm) added into the deuterated chloroform ($CDCl_3$, 7.26 ppm) unless otherwise stated. Data for ^{1}H NMR were reported as follows: chemical shift, multiplicity (s = singlet, d = doublet, t = triplet, q = quartet, quint = quintet, m = multiplet, dd = doublet of doublets, dt = doublet of triplets, td = triplet of doublets, and br = broad signal), coupling constants, and integration. All ^{13}C NMR spectra were reported in ppm relative to tetramethylsilane (0.00 ppm) unless otherwise stated, and were obtained with complete ^{1}H decoupling. All GC analyses were performed on a Shimadzu GC-2014C with an FID detector. All GC-MS analyses were performed on a Shimadzu GCMS-QP2020NX. High-resolution mass spectra (HRMS) by electrospray ionization (ESI), atmospheric pressure chemical ionization (APCI), and atmospheric pressure photoionization (APPI) method were performed at the EPFL ISIC Mass Spectroscopy Service.

Preparation of Alkyl Chlorides

Substrates, **2c**[1], **2g – 2j**[2], **2l - 2q**[2], **2x – 2aa**[2], **2k - 2l**[3], **2r**[4] , **2u – 2w**[5] in Scheme 1 and **2h – 2j**[6], **2ac – 2ai**[7] in Scheme 2 were prepared by methods described in the references.

Preparation of Alkyl Chlorides **2g – 2j**, **2l - 2q, 2x – 2aa**

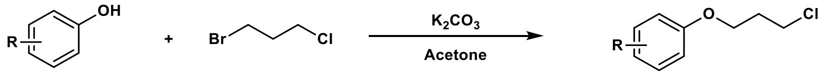

A flame-dried flask was charged with phenol (5.0 mmol), 1-bromo-3-chloropropane (5.5 mmol, 1.1 equiv.), K_2CO_3 (5.5 mmol, 1.1 equiv.) and Acetone (10 mL) in batches at 60 ºC, and the mixture was stirred for 24 h under nitrogen protection. Then the mixture was cooling to room temperature, dried with anhydrous Na_2SO_4, then the mixture was filtered over Celite. The organic phase was concentrated in vacuum. Purification by column chromatography affords desired product.

General Procedure A for Cross-Electrophile Sulfonylation

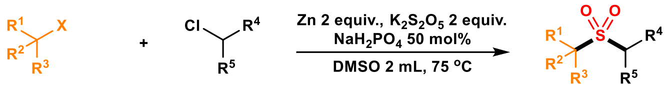

An oven-dried 50 mL Schlenk tube equipped with a Teflon-coated magnetic stir bar was sequentially charged with Zn powder (0.4 mmol, 2 equiv.), $K_2S_2O_5$ (0.4 mmol, 2 equiv.), NaH_2PO_4 (0.1 mmol, 50 mol%) in the glovebox. Then 1.5 mL DMSO, alkyl halides **1** (0.4 mmol, 2 equiv.) were added into the tube in turn. All these procedures were conducted in the glovebox. The vial was sealed with a rubber stopper and removed from the glove box. Then the vial was placed on the heating-base and reacted at 75 °C for 2 h. After which time the alkyl chloride **2** (0.2 mmol) was dissolved into 0.5 mL of DMSO and added to the Schlenk tube with a needle. Then the vial was placed on the heating-base and reacted at 75 °C for 22 h. After which time the vial was removed from the heating source, and the product was extracted from the crude reaction mixture with ethyl acetate (3 x 10 mL). The organic layers were combined, and washed with brine (30 mL). Dried over Na_2SO_4, filtered, and concentrated under reduced pressure. The crude product residue was purified by preparative TLC using a solvent mixture (EtOAc, petroleum ether) as an eluent to afford the purified product.
[a] Variation: **1** (0.8 mmol), Zn powder (0.8 mmol), $K_2S_2O_5$ (0.8 mmol), 48 h. [b] Variation: Add NaI (0.1 mmol), DMSO 4 mL, 48 h. [c] Variation: Scale up reaction.

General Procedure B for Cross-Electrophile Sulfonylation

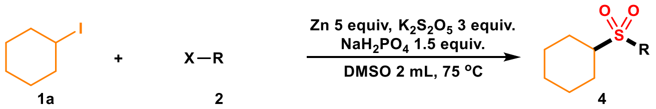

An oven-dried 50 mL Schlenk tube equipped with a Teflon-coated magnetic stir bar was sequentially charged with Zn powder (1 mmol, 5 equiv.), $K_2S_2O_5$ (0.6 mmol, 3 equiv.), NaH_2PO_4 (0.3 mmol, 1.5 equiv.) in the glovebox. Then 2 mL DMSO, alkyl chloride **2** (0.4 mmol, 2 equiv.), iodocyclohexane **1a** (0.2 mmol) were added into the tube in turn. All these procedures were conducted in the glovebox. The vial was sealed with a rubber stopper and removed from the glove box. Then the vial was placed on the heating-base and reacted at 75 °C for 24 h. After which time the vial was removed from the heating source, and the product was extracted from the crude reaction mixture with ethyl acetate (3 x 10 mL). The organic layers were combined, and washed with brine (30 mL). Dried over Na_2SO_4, filtered, and concentrated under reduced pressure. The crude product residue was purified by preparative TLC using a solvent mixture (EtOAc, petroleum ether) as an eluent to afford the purified product.

[a] Variation: Using General Procedure A.

General Procedure C for Homo-Electrophile Sulfonylation

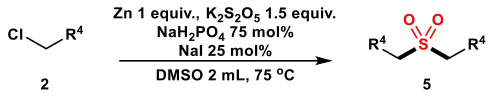

An oven-dried 50 mL Schlenk tube equipped with a Teflon-coated magnetic stir bar was sequentially charged with Zn powder (0.4 mmol, 1 equiv.), $K_2S_2O_5$ (0.6 mmol, 1.5 equiv.), NaH_2PO_4 (0.3 mmol, 75 mol%) in the glovebox. Then NaI (0.1 mmol, 25 mol%), 2 mL DMSO, alkyl chloride **2** (0.4 mmol), were added into the tube in turn. All these procedures were conducted in the glovebox. The vial was sealed with a rubber stopper and removed from the glove box. Then the vial was placed on the heating-base and reacted at 75 °C for 24 h. After which time the vial was removed from the heating source, and the product was extracted from the crude reaction mixture with ethyl acetate (3 x 10 mL). The organic layers were combined, and washed with brine (30 mL). Dried over Na_2SO_4, filtered, and concentrated under reduced pressure. The crude product residue was purified by preparative TLC using a solvent mixture (EtOAc, petroleum ether) as an eluent to afford the purified product.

Summary of the effects of reaction parameters and conditions on the reaction efficiency

Table S1. Screening of Solvents

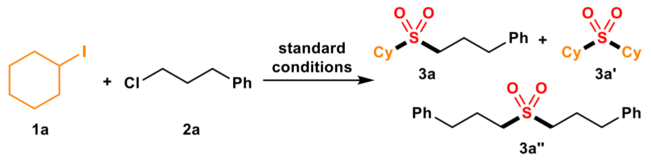

Entry	Variation from conditions	**3a** Yield (%)[a]	**3a'** Yield (%)[a]	**3a''** Yield (%)[a]
1	DMF instead of DMSO	65	2	15
2	DMA instead of DMSO	24	1	19
3	DMPU instead of DMSO	33	2	20
4	NMP instead of DMSO	36	3	25
5	MeCN instead of DMSO	5	trace	3
6	THF instead of DMSO	2	trace	8
7	PhMe instead of DMSO	trace	trace	trace
8	DCM instead of DMSO	trace	trace	trace

The reaction conditions：**1a** (0.2 mmol), **2a** (2 equiv.), $K_2S_2O_5$ (3 equiv.), NaH_2PO_4(1.5 equiv.), Zn powder(5 equiv.) and Solvents (2 mL), 24 h, 75 °C. [a] The yields were detected by GC using naphthalene as an internal standard.

Table S2. Screening of Temperatures

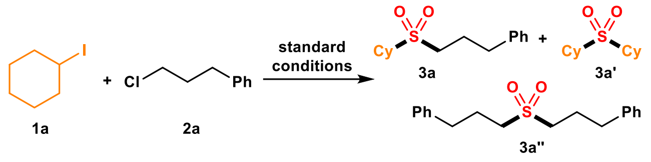

Entry	T / °C	**3a** Yield (%)[a]	**3a'** Yield (%)[a]	**3a''** Yield (%)[a]
1	120	47	5	63
2	100	62	4	51
3	80	81	3	20
4	60	57	1	7
5	20	n.d.	n.d.	n.d.

The reaction conditions：**1a** (0.2 mmol), **2a** (2 equiv.), $K_2S_2O_5$ (3 equiv.), NaH_2PO_4(1.5 equiv.), Zn powder(5 equiv.) and DMSO (2 mL), 24 h, T / °C. [a] The yields were detected by GC using naphthalene as an internal standard.

Table S3. Screening of Sulfur Dioxide Source

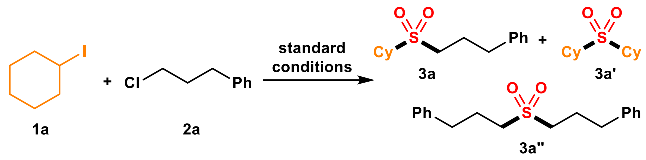

Entry	Variation from condition	**3a** Yield (%)[a]	**3a'** Yield (%)[a]	**3a''** Yield (%)[a]
1	$Na_2S_2O_5$ instead of $K_2S_2O_5$	88	2	15
2	$Na_2S_2O_4$ instead of $K_2S_2O_5$	36	3	48
3	DABSO instead of $K_2S_2O_5$	35	1	12

The reaction conditions: **1a** (0.2 mmol), **2a** (2 equiv.), Sulfur dioxide Source (3 equiv.), NaH_2PO_4(1.5 equiv.), Zn powder(5 equiv.) and DMSO (2 mL), 24 h, 75 °C. [a] The yields were detected by GC using naphthalene as an internal standard.

Table S4. Screening of Reducing Agent

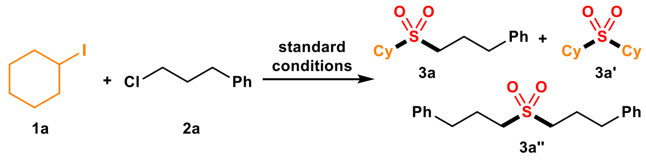

Entry	Variation from condition	**3a** Yield (%)[a]	**3a'** Yield (%)[a]	**3a''** Yield (%)[a]
1	Mn instead of Zn	75	2	16
2	Mg instead of Zn	trace	n.d.	trace
5	Al instead of Zn	n.d.	n.d.	n.d.

The reaction conditions: **1a** (0.2 mmol), **2a** (2 equiv.), $K_2S_2O_5$ (3 equiv.), NaH_2PO_4(1.5 equiv.), Reducing Agent (5 equiv.) and DMSO (2 mL), 24 h, 75 °C. [a] The yields were detected by GC using naphthalene as an internal standard.

Table S5. Screening of Equivalent of Sodium Dihydrogen Phosphate

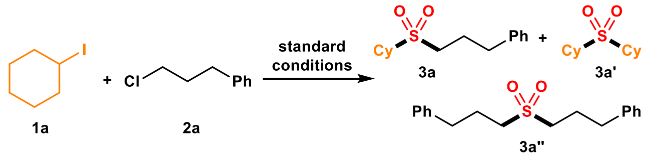

Entry	Variation from conditions	**3a** Yield (%)[a]	**3a'** Yield (%)[a]	**3a''** Yield (%)[a]
1	NaH_2PO_4 1 equiv.	83	2	21
2	NaH_2PO_4 2 equiv.	90	2	13

The reaction conditions: **1a** (0.2 mmol), **2a** (2 equiv.), $K_2S_2O_5$ (3 equiv.), NaH_2PO_4 , Zn powder(5 equiv.) and DMSO (2 mL), 24 h, 75 °C. [a] The yields were detected by GC using naphthalene as an internal standard.

Table S6. Screening of Reaction Conditions

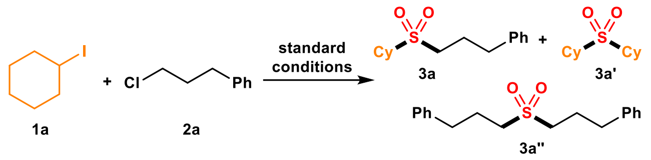

Entry	Variation from conditions	**3a** Yield (%)[a]	**3a'** Yield (%)[a]	**3a''** Yield (%)[a]
1	No Zn	n.d.	n.d.	n.d.
2	No $K_2S_2O_5$	n.d.	n.d.	n.d.
3	No NaH_2PO_4	62	4	37

The reaction conditions：**1a** (0.2 mmol), **2a** (2 equiv.), $K_2S_2O_5$ (3 equiv.), NaH_2PO_4(1.5 equiv.), Zn powder(5 equiv.) and DMSO (2 mL), 24 h, 75 °C. [a] The yields were detected by GC using naphthalene as an internal standard.

Control Experiments

Control Experiments

1) Radical quench experiment

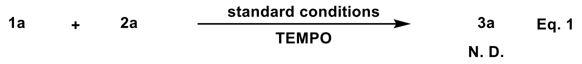

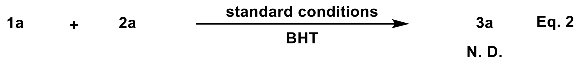

2) Radical trapping experiment

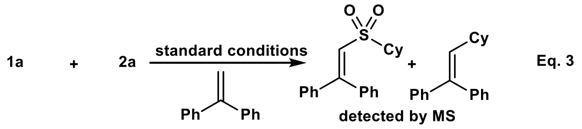

3) Radical clock experiment

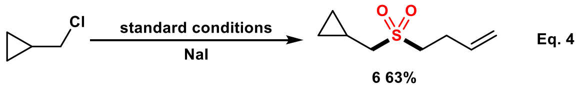

4) Excluding the mechanism of organozinc experiment

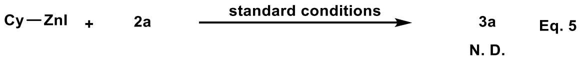

5) Alkyl sulfinate intermediate trapping experiment

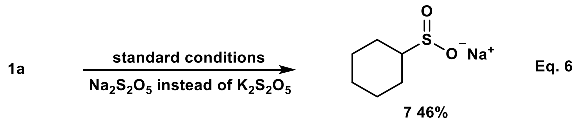

6) Alkylation of alkyl sulfinate intermediate

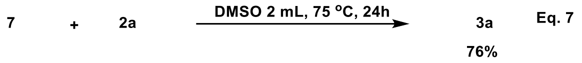

7) Sulfur dioxide detection experiment

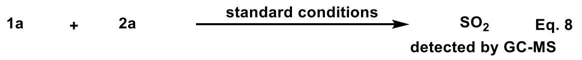

Equation 1 followed the standard conditions (General Procedure B) using TEMPO(5 equiv.) and BHT(5 equiv.) as the radical quench reagent. After reaction, the sulfone production(**3a**) was not detected.

Equation 2 followed the standard conditions (General Procedure B) using 1,1-Diphenylethylene(3 equiv.) as the radical trapping reagent. After reaction, radical addition of sulfone radical and radical addition of alkyl radical were detected by HRMS.

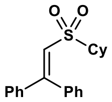

HRMS (ESI/QTOF) m/z: $[M + Na]^+$ Calcd for $C_{20}H_{23}O_2SNa^+$ 349.1233; Found 349.1238.

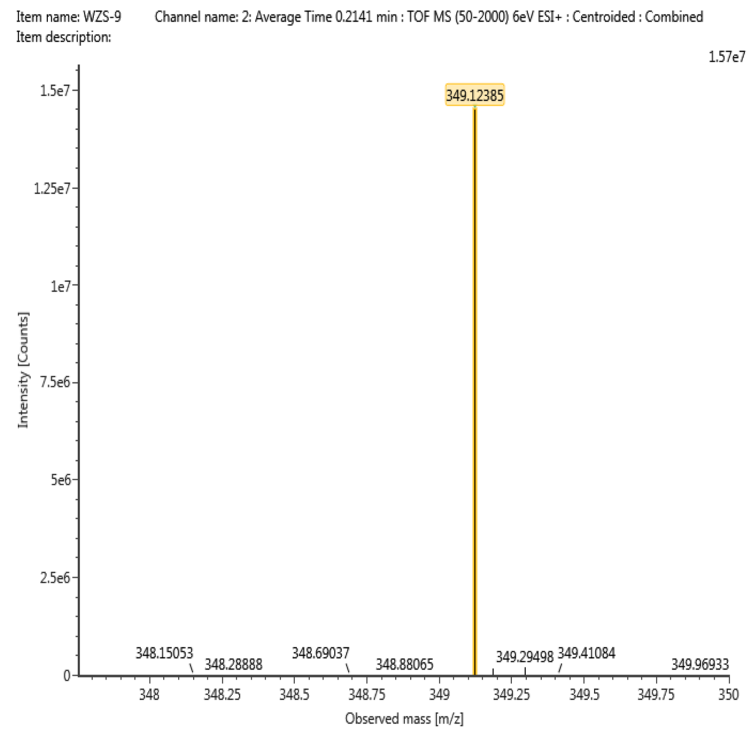

Figure S1. Sulfone radical detected by HRMS.

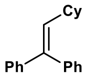

HRMS (ESI/QTOF) m/z: [M] Calcd for $C_{16}H_{22}$ 262.1722; Found 262.1742.

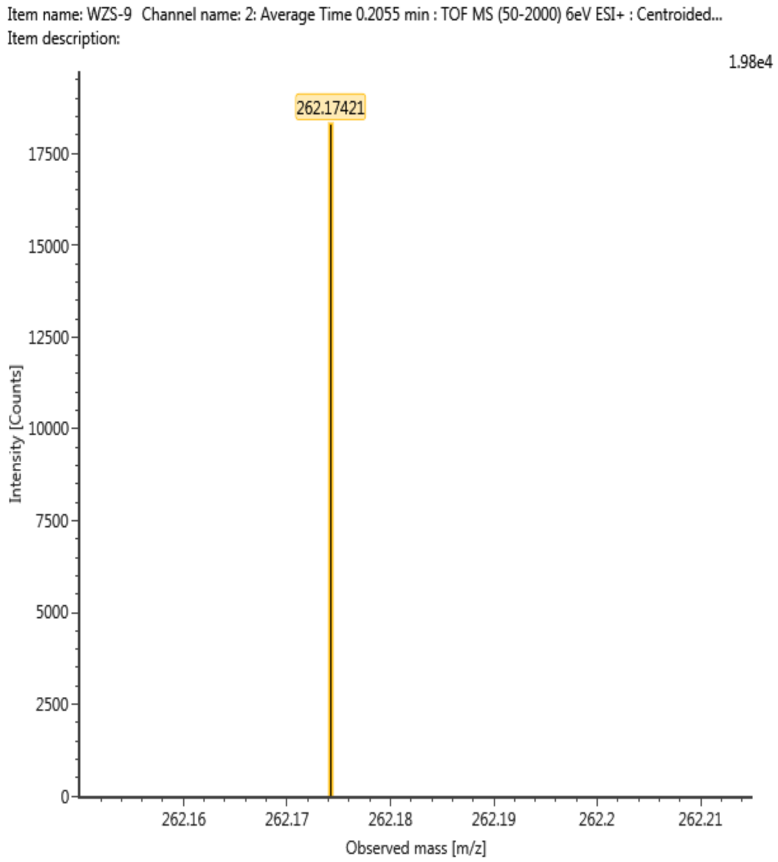

Figure S2. Alkyl radical detected by HRMS.

Equation 3 followed the standard conditions (General Procedure C) using (chloromethyl)cyclopropane (0.4 mmol) as the substrate.

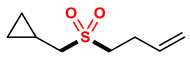

((but-3-en-1-ylsulfonyl)methyl)cyclopropane (6): Purified by preparative TLC, using petroleum ether /EA = 10/1 (v/v) as an eluent. Colorless liquid in 63% yield.

^{1}H NMR (400 MHz, $CDCl_3$) δ 5.85 – 5.71 (m, 1H), 5.14 – 4.98 (m, 2H), 3.03 (td, *J* = 8.2, 3.3 Hz, 2H), 2.85 (dd, *J* = 7.2, 3.1 Hz, 2H), 2.54 (ddt, *J* = 11.3, 9.6, 3.9 Hz, 2H), 1.12 (tq, *J* = 7.9, 4.7, 3.5 Hz, 1H), 0.70 (qd, *J* = 5.1, 2.5 Hz, 2H), 0.33 (d, *J* = 5.1 Hz, 2H).

^{13}C NMR (101 MHz, $CDCl_3$) δ 133.99, 117.33, 58.32, 51.29, 25.86, 4.62, 4.52.

HRMS (ESI/QTOF) m/z: $[M + Na]^+$ Calcd for $C_8H_{14}O_2SNa^+$ 197.0607; Found 197.0621.

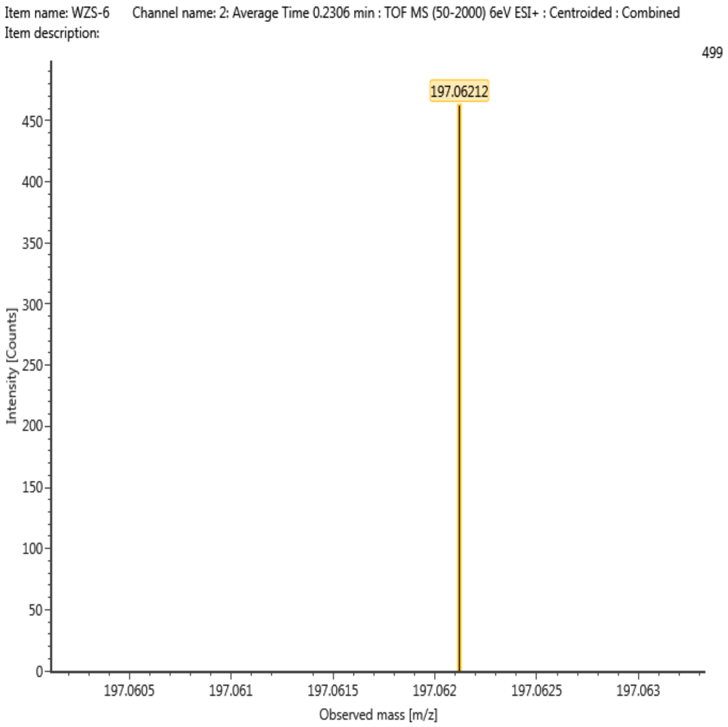

Figure S3. 6 detected by HRMS.

Equation 4 followed the standard conditions (General Procedure C) using cyclohexylzinc(II) iodide (0.2 mmol) and **2a** (2 equiv.) as the substrate. After reaction, the sulfone production(**3a**) was not detected.[8]

Equation 5 the detection of sulfinate **7**:

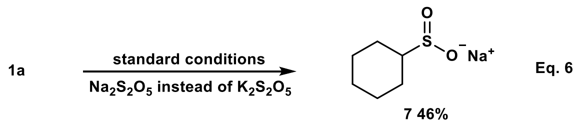

An oven-dried 50 mL schlenk tube equipped with a Teflon-coated magnetic stir bar was sequentially charged with Zn podwer (0.4 mmol), $Na_2S_2O_5$ (0.4 mmol), NaH_2PO_4 (0.1 mmol) in the glovebox. Then 1.5 mL DMSO, **1a** (0.2 mmol) were added into the tube in turn. All these procedures were conducted in the glovebox.

The vial was sealed with a rubber stopper and removed from the glove box. Then the vial was placed on the heating-base and reacted at 75 °C for 10 h. After cooling to room temperature, add ethanol, Na_2CO_3 and stir for a while, the mixture was filtered over Celite. The solvent was removed under reduced pressure. Then the remaining solid was extracted and recrystallized by MeCN to get a white solid[9].

^1H NMR (400 MHz, D_2O) δ 1.99 (tt, J = 11.4, 3.5 Hz, 1H), 1.85 (dddd, J = 17.3, 10.7, 4.8, 2.3 Hz, 4H), 1.70 – 1.61 (m, 1H), 1.35 – 1.15 (m, 5H). ^{13}C NMR (101 MHz, D_2O) δ 65.66, 25.48, 24.98, 24.44.

Equation 6 using sodium sulfinate **7** and **2a** to afford sulfone **3a**.

Equation 7 followed the standard conditions (General Procedure B). After reaction, the sulfur dioxide was detected by GC-MS.

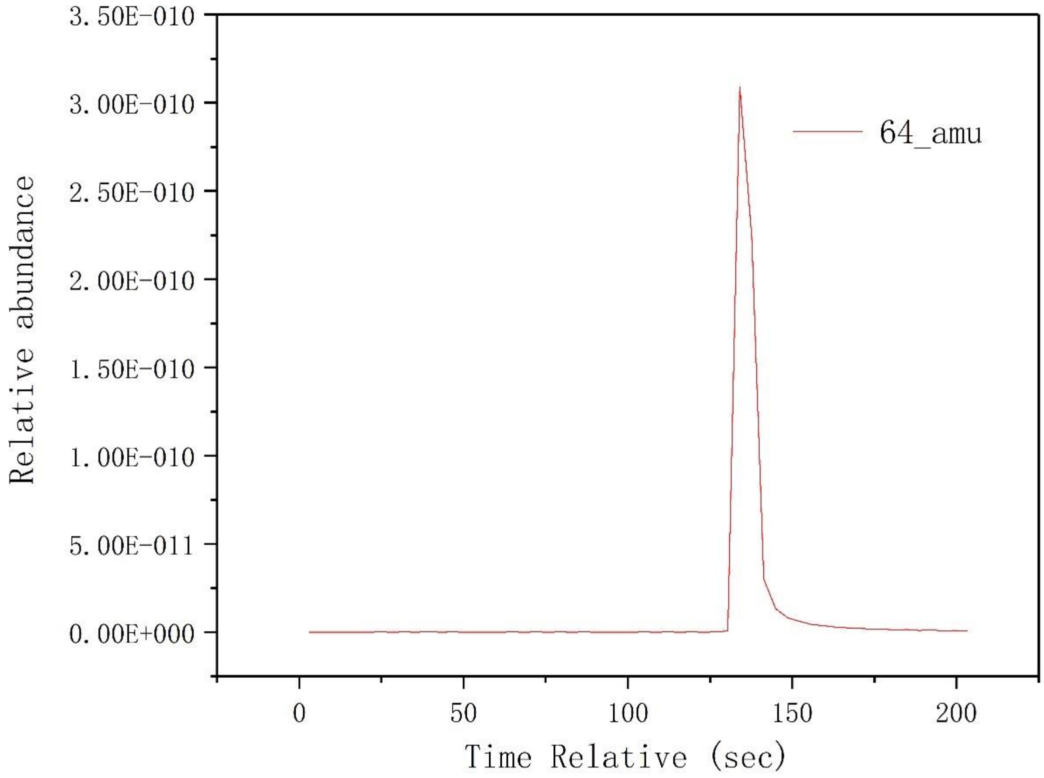

Figure S4. Sulfur dioxide(64_amu) was detected by GC-MS

Scale up reaction

An oven-dried 100 mL Schlenk tube equipped with a Teflon-coated magnetic stir bar was sequentially charged with Zn podwer (20 mmol, 2 equiv.), $K_2S_2O_5$ (20 mmol, 2 equiv.), NaH_2PO_4 (5 mmol, 0.5 equiv.) in the glovebox. Then 7.5 mL DMSO, *tert*-butyl iodide **1** (20 mmol, 2 equiv.) were added into the tube in turn. All these procedures were conducted in the glovebox. The vial was sealed with a rubber stopper and removed from the glove box. Then the vial was placed on the heating-base and reacted at 75 ^{o}C for 6 h. After which time the (3-chloropropoxy)benzene **2** (10 mmol) was dissolved into 2.5 mL of DMSO and added to the schlenk tube with a needle. Then the vial was placed on the heating-base and reacted at 75 ^{o}C for 48 h. After which time the vial was removed from the heating source, and the product was extracted from the crude reaction mixture with ethyl acetate (3 x 30 mL). The organic layers were combined, and washed with brine (3 x 30 mL). Dried over Na_2SO_4, filtered, and concentrated under reduced pressure. The crude product residue was purified by preparative TLC using a solvent mixture (EtOAc, petroleum ether) as an eluent to afford the purified product **3q** (1.5 g) in 59% yield..

Computational Details

a) Computational methods

All DFT calculations were carried out using the Gaussian 16 series of programs[10]. Geometries of intermediates and transition states were optimized using dispersion-corrected B3LYP-D3(BJ) functional[11] with a mixed basis set of LANL2DZ for Zn, I and 6-31G(d) for other atoms in the gas phase. Vibrational frequency calculations were performed for all stationary points to confirm if each optimized structure is a local minimum or a transition state structure. All optimized transition state structures have only one imaginary (negative) frequency, and all minima (reactants, products, and intermediates) have no imaginary frequencies. The M06-L functional[12] with a mixed basis set of SDD for Zn, I and 6-311+G(d,p) for other atoms was used for single-point energy calculations in solution. Solvation energy corrections were calculated in dimethylsulfoxide as solvent with the SMD continuum solvation model[13] based on the gas-phase optimized geometries. The 3D images of structures were prepared using CYLView[14].

b) Monomeric and dimeric ZnI_2 complexes

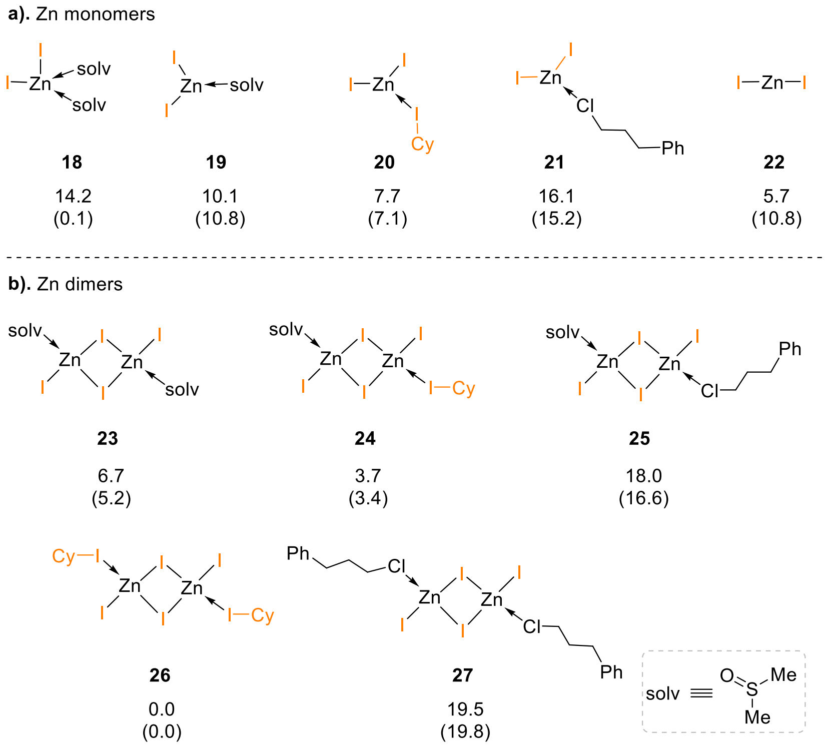

Figure S5. Gibbs energies of monomeric and dimeric ZnI_2 complexes. Calculations were performed using Gaussian 16 at the M06-L/SDD-6-311+G(d,p)/SMD(DMSO)//B3LYP-D3(BJ)/LANL2DZ-6-31G(d) level of theory. All energies are in kcal/mol relative to **26**.

c) $Zn(SO_2Cy)_2$ complexes

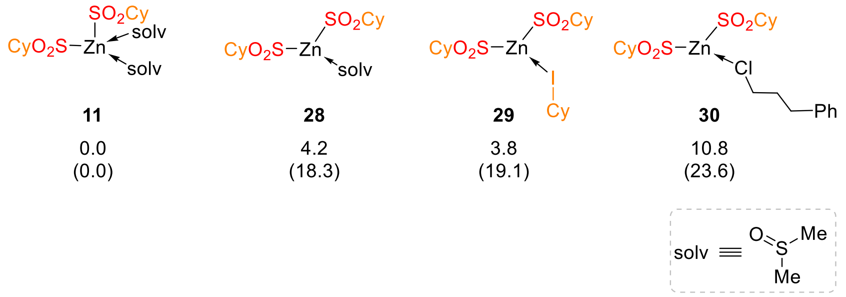

Figure S6. Gibbs energies of $Zn(SO_2Cy)_2$ complexes. Calculations were performed using Gaussian 16 at the M06-L/SDD-6-311+G(d,p)/SMD(DMSO)//B3LYP-D3(BJ)/LANL2DZ-6-31G(d) level of theory. All energies are in kcal/mol relative to **11**.

d) Calculations of the reduction of iodocyclohexane and cyclohexylsulfonyl radical by Zn

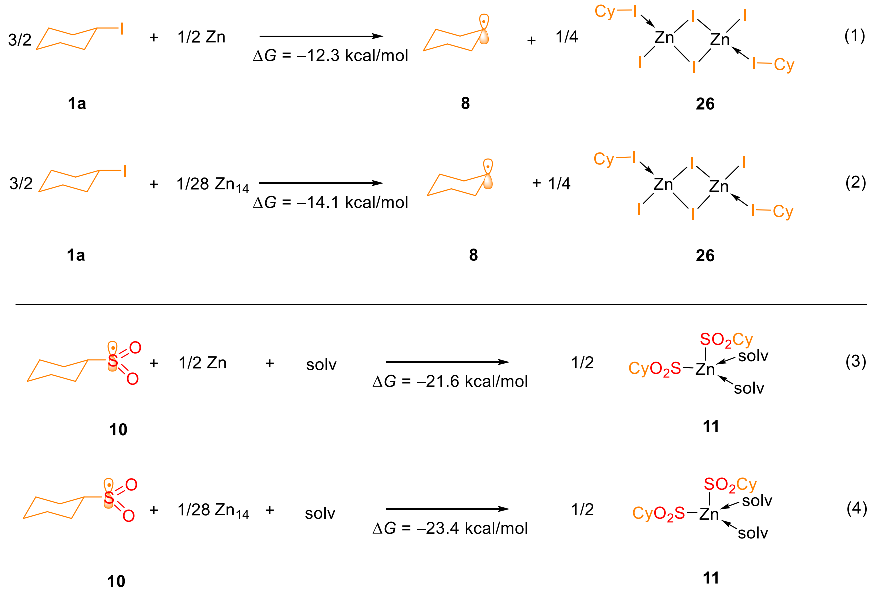

Figure S7. Gibbs energies of the reduction of iodocyclohexane **1a** and cyclohexylsulfonyl radical intermediate **10** by Zn. Calculations were performed using Gaussian 16 at the M06-L/6-311+G(d,p)-SDD/SMD(DMSO)//B3LYP-D3(BJ)/6-31G(d)-LANL2DZ level of theory.

e) Cartesian coordinates (Å) and energies of optimized structures

1a

B3LYP-D3(BJ) SCF energy: -246.69384617 a.u.

B3LYP-D3(BJ) enthalpy: -246.524280 a.u.

B3LYP-D3(BJ) free energy: -246.565358 a.u.

M06-L SCF energy in solution: -246.71526025 a.u.

M06-L enthalpy in solution: -246.545694 a.u.

M06-L free energy in solution: -246.586772 a.u.

Cartesian coordinates

ATOM	X	Y	Z
C	2.627366	-1.263969	0.187848
C	1.118906	-1.268142	-0.128337
C	0.477775	0.000000	0.419609
C	1.118906	1.268141	-0.128335
C	2.627366	1.263970	0.187846
C	3.308964	0.000000	-0.351165
H	0.971805	-1.307019	-1.214885
H	0.639147	-2.155432	0.295914
H	2.769955	-1.319120	1.276457
H	3.088754	-2.164092	-0.235796
H	0.971803	1.307021	-1.214883
H	0.639147	2.155431	0.295917
H	3.088751	2.164092	-0.235803
H	2.769959	1.319125	1.276455
H	3.254924	0.000000	-1.449237
H	4.373360	-0.000001	-0.087265
H	0.487411	-0.000002	1.511768
I	-1.711900	0.000000	-0.025348

8

B3LYP-D3(BJ) SCF energy: -235.23429255 a.u.
B3LYP-D3(BJ) enthalpy: -235.070820 a.u.
B3LYP-D3(BJ) free energy: -235.107543 a.u.
M06-L SCF energy in solution: -235.23910261 a.u.
M06-L enthalpy in solution: -235.075630 a.u.
M06-L free energy in solution: -235.112353 a.u.

Cartesian coordinates

ATOM	X	Y	Z
C	-1.265325	0.711889	-0.245309
C	-1.288554	-0.776291	0.158450
C	0.000000	-1.462258	-0.168525
C	1.288554	-0.776291	0.158450
C	1.265325	0.711889	-0.245309
C	0.000000	1.408131	0.273471
H	-1.474192	-0.828732	1.250031
H	-2.135790	-1.289340	-0.313133
H	-1.290586	0.786659	-1.341107
H	-2.164126	1.215977	0.130849
H	1.474193	-0.828732	1.250031
H	2.135790	-1.289340	-0.313133
H	2.164126	1.215977	0.130849
H	1.290586	0.786660	-1.341107

H	0.000000	1.390330	1.373843
H	0.000000	2.464311	-0.023731
H	0.000000	-2.526176	-0.390762

SO_2

B3LYP-D3(BJ) SCF energy: -548.58443759 a.u.
B3LYP-D3(BJ) enthalpy: -548.573603 a.u.
B3LYP-D3(BJ) free energy: -548.602509 a.u.
M06-L SCF energy in solution: -548.64107587 a.u.
M06-L enthalpy in solution: -548.630241 a.u.
M06-L free energy in solution: -548.659147 a.u.

Cartesian coordinates

ATOM	X	Y	Z
S	0.000000	0.371609	0.000000
O	1.260809	-0.371629	0.000000
O	-1.260809	-0.371588	0.000000

9-ts

B3LYP-D3(BJ) SCF energy: -783.83569776 a.u.
B3LYP-D3(BJ) enthalpy: -783.659319 a.u.
B3LYP-D3(BJ) free energy: -783.707337 a.u.
M06-L SCF energy in solution: -783.89731349 a.u.
M06-L enthalpy in solution: -783.720935 a.u.
M06-L free energy in solution: -783.768953 a.u.
Imaginary frequency: -57.4465 cm^{-1}

Cartesian coordinates

ATOM	X	Y	Z
S	2.087350	-0.003414	0.148273
O	2.013191	1.265637	-0.611346
O	2.003437	-1.266281	-0.620644
C	-2.073842	-1.247801	-0.222216
C	-2.031269	-0.000890	-1.109969
C	-2.069892	1.250590	-0.228487
C	-0.840269	1.325119	0.697618
C	-0.368809	0.002244	1.217788
C	-0.843891	-1.321648	0.703445
H	-2.115410	2.160536	-0.836514
H	-1.115787	-0.003978	-1.718482
H	-2.878765	-0.001252	-1.805114
H	-2.990649	-1.217249	0.381856
H	-2.122678	-2.160627	-0.825650
H	-0.014742	1.795589	0.138901

H	-1.025879	1.999215	1.545155
H	0.138859	0.003733	2.179568
H	-0.020057	-1.796152	0.145711
H	-1.030552	-1.991848	1.553825
H	-2.987122	1.226123	0.375215

10

B3LYP-D3(BJ) SCF energy: -783.85904197 a.u.

B3LYP-D3(BJ) enthalpy: -783.679601 a.u.

B3LYP-D3(BJ) free energy: -783.724577 a.u.

M06-L SCF energy in solution: -783.91495507 a.u.

M06-L enthalpy in solution: -783.735514 a.u.

M06-L free energy in solution: -783.780490 a.u.

Cartesian coordinates

ATOM	X	Y	Z
S	-1.834097	-0.000011	0.263074
O	-2.405084	-1.295706	-0.204746
O	-2.404975	1.295768	-0.204621
C	2.123714	-1.266388	-0.236459
C	2.846173	0.000004	0.242240
C	2.123711	1.266377	-0.236514
C	0.649498	1.274454	0.202769
C	-0.035320	-0.000021	-0.282559
C	0.649497	-1.274464	0.202822
H	2.621673	2.164278	0.147280
H	2.887119	0.000042	1.340905
H	3.883688	-0.000022	-0.111881
H	2.173541	-1.322687	-1.332954
H	2.621665	-2.164267	0.147407
H	0.596863	1.318867	1.299420
H	0.126906	2.153403	-0.184166
H	-0.126395	-0.000027	-1.376418
H	0.596867	-1.318845	1.299474
H	0.126929	-2.153439	-0.184086
H	2.173525	1.322614	-1.333017

11

B3LYP-D3(BJ) SCF energy: -2739.91987287 a.u.

B3LYP-D3(BJ) enthalpy: -2739.376534 a.u.

B3LYP-D3(BJ) free energy: -2739.479150 a.u.

M06-L SCF energy in solution: -2901.60961695 a.u.

M06-L enthalpy in solution: -2901.066278 a.u.

M06-L free energy in solution: -2901.168894 a.u.

Cartesian coordinates

ATOM	X	Y	Z
Zn	-0.351515	0.888094	0.291685
O	-0.051246	0.780739	2.279421
O	-0.893375	2.821652	0.060787
S	-1.047433	3.642925	-1.262426
S	-1.099646	0.852047	3.435300
C	0.601947	4.346076	-1.547652
H	1.323069	3.522064	-1.587459
H	0.579088	4.896970	-2.493020
H	0.807735	5.030708	-0.721851
C	-1.084649	2.456833	-2.633650
H	-1.889930	1.741190	-2.418583
H	-1.310618	3.022228	-3.543389
H	-0.106697	1.968204	-2.709624
C	-2.580079	1.656952	2.751784
H	-3.026715	1.002161	1.991755
H	-3.274691	1.831242	3.579321
H	-2.254958	2.607370	2.322063
C	-1.752776	-0.834678	3.587574
H	-2.493269	-0.838618	4.393601
H	-2.205886	-1.120028	2.631200
H	-0.910058	-1.476891	3.852611
S	-2.312467	-0.370861	-0.397628
C	-1.637396	-1.954573	-1.008619
C	-0.825817	-2.628343	0.097248
C	-2.732922	-2.867463	-1.554170
H	-0.952229	-1.648701	-1.808340
C	-0.176496	-3.906841	-0.446214
H	-1.492998	-2.865916	0.935670
H	-0.050227	-1.947881	0.471863
C	-2.101622	-4.163358	-2.087407
H	-3.441041	-3.092963	-0.746447
H	-3.289400	-2.345946	-2.339345
C	-1.238317	-4.856083	-1.021893
H	0.390219	-4.407753	0.349151
H	0.542701	-3.623712	-1.225089
H	-2.886910	-4.843389	-2.440522
H	-1.475329	-3.923133	-2.957960
H	-0.759425	-5.747696	-1.445474
H	-1.887983	-5.204540	-0.205394
S	1.585917	0.327663	-1.054871
C	3.068554	0.123053	0.013444

C	2.823488	-0.989650	1.034247
C	4.295446	-0.150887	-0.858820
H	3.172434	1.091032	0.523019
C	4.069816	-1.210139	1.904328
H	2.583140	-1.909886	0.487303
H	1.958773	-0.745995	1.664989
C	5.537485	-0.375133	0.015493
H	4.093769	-1.045505	-1.460857
H	4.442401	0.680849	-1.554565
C	5.307507	-1.492992	1.042013
H	3.892993	-2.036861	2.603535
H	4.248539	-0.313121	2.514852
H	6.399760	-0.612059	-0.619867
H	5.783376	0.557155	0.544786
H	6.193289	-1.614863	1.677724
H	5.164639	-2.444994	0.511009
O	-3.048826	0.256516	-1.553479
O	-3.167904	-0.729284	0.807801
O	1.396484	-0.998041	-1.737809
O	1.916234	1.502345	-1.954153

12

B3LYP-D3(BJ) SCF energy: -1955.83692063 a.u.
B3LYP-D3(BJ) enthalpy: -1955.476033 a.u.
B3LYP-D3(BJ) free energy: -1955.558190 a.u.
M06-L SCF energy in solution: -2117.51684426 a.u.
M06-L enthalpy in solution: -2117.155957 a.u.
M06-L free energy in solution: -2117.238114 a.u.

Cartesian coordinates

ATOM	X	Y	Z
Zn	-0.906888	0.319997	0.218186
O	-0.423072	2.084061	0.973758
O	-2.887254	0.213674	0.209268
S	-3.636150	-1.112712	0.595007
S	0.261593	3.284430	0.245947
C	-5.369794	-0.596686	0.526415
H	-5.565603	-0.125742	-0.439903
H	-6.002568	-1.476147	0.674741
H	-5.526752	0.117509	1.337148
C	-3.567310	-2.148782	-0.890539
H	-2.526538	-2.463571	-0.987295
H	-4.213801	-3.018216	-0.740286
H	-3.888006	-1.563730	-1.756495

C	1.952389	2.749293	-0.148001
H	1.908301	1.819103	-0.719166
H	2.435259	3.550240	-0.715885
H	2.474614	2.605574	0.800276
C	-0.412131	3.287022	-1.442793
H	0.052429	4.106164	-1.999499
H	-0.206453	2.326143	-1.924306
H	-1.486497	3.467458	-1.358535
S	0.583255	-1.501558	-1.060350
C	2.205523	-1.684695	-0.202658
C	3.323753	-1.234371	-1.150591
C	2.235788	-0.961737	1.143694
H	2.269927	-2.769762	-0.041088
C	4.694065	-1.392697	-0.472754
H	3.163773	-0.181047	-1.413232
H	3.288762	-1.805980	-2.085907
C	3.617544	-1.113834	1.801279
H	2.029407	0.104252	0.984493
H	1.451800	-1.356109	1.797292
C	4.741381	-0.647700	0.867078
H	5.477550	-1.027702	-1.145829
H	4.894318	-2.459577	-0.304826
H	3.636555	-0.551406	2.741741
H	3.775485	-2.168556	2.063610
H	5.715221	-0.793680	1.346878
H	4.640126	0.432759	0.684817
O	-0.460407	-1.686159	0.112642
O	0.463599	0.060257	-1.298264

13

B3LYP-D3(BJ) SCF energy: -783.92525817 a.u.

B3LYP-D3(BJ) enthalpy: -783.747459 a.u.

B3LYP-D3(BJ) free energy: -783.791567 a.u.

M06-L SCF energy in solution: -784.06836807 a.u.

M06-L enthalpy in solution: -783.890569 a.u.

M06-L free energy in solution: -783.934677 a.u.

Cartesian coordinates

ATOM	X	Y	Z
S	1.861351	0.000046	-0.359191
O	2.369911	-1.285692	0.292746
O	2.370051	1.285565	0.293068
C	-2.126650	-1.269263	0.240708
C	-2.850977	-0.000038	-0.234946

C	-2.126702	1.269268	0.240572
C	-0.652270	1.264457	-0.191706
C	0.070507	0.000051	0.257994
C	-0.652218	-1.264433	-0.191569
H	-2.645642	2.163268	-0.138711
H	-2.881531	-0.000097	-1.335760
H	-3.896306	-0.000043	0.108190
H	-2.179937	-1.317625	1.339179
H	-2.645552	-2.163325	-0.138482
H	-0.604749	1.328770	-1.292050
H	-0.114126	2.135243	0.197078
H	0.195114	0.000105	1.350338
H	-0.604691	-1.328860	-1.291906
H	-0.114043	-2.135159	0.197312
H	-2.179990	1.317744	1.339039

2a

B3LYP-D3(BJ) SCF energy: -809.81688073 a.u.
B3LYP-D3(BJ) enthalpy: -809.628529 a.u.
B3LYP-D3(BJ) free energy: -809.676225 a.u.
M06-L SCF energy in solution: -809.83916565 a.u.
M06-L enthalpy in solution: -809.650814 a.u.
M06-L free energy in solution: -809.698510 a.u.

Cartesian coordinates

ATOM	X	Y	Z
C	3.074987	-1.206254	-0.358504
C	3.711913	0.000269	-0.653758
C	3.074799	1.206566	-0.357963
C	1.808984	1.203521	0.228125
C	1.159937	-0.000194	0.528472
C	1.809166	-1.203669	0.227586
H	3.565052	-2.150068	-0.581194
H	4.698781	0.000448	-1.107676
H	3.564720	2.150556	-0.580229
H	1.317575	2.146039	0.459209
H	1.317905	-2.146368	0.458242
C	-0.232960	-0.000420	1.113444
C	-1.313486	-0.000013	0.013015
H	-0.368830	0.880193	1.754428
H	-0.368817	-0.881515	1.753767
H	-1.186242	0.880062	-0.627141
H	-1.186216	-0.879593	-0.627815
C	-2.710542	-0.000260	0.613157

H	-2.888801	0.887637	1.224103
H	-2.888786	-0.888638	1.223408
Cl	-3.992537	0.000233	-0.674738

14-ts

B3LYP-D3(BJ) SCF energy: -1593.78069590 a.u.
B3LYP-D3(BJ) enthalpy: -1593.413247 a.u.
B3LYP-D3(BJ) free energy: -1593.485478 a.u.
M06-L SCF energy in solution: -1593.89975438 a.u.
M06-L enthalpy in solution: -1593.532305 a.u.
M06-L free energy in solution: -1593.604536 a.u.
Imaginary frequency: -323.3039 cm^{-1}

Cartesian coordinates

ATOM	X	Y	Z
S	0.903739	-0.724142	0.638298
O	0.835217	-0.864639	2.140688
O	0.226137	-1.816590	-0.169174
C	5.053656	-0.166195	0.682752
C	5.305025	-0.111082	-0.830947
C	4.437462	-1.135021	-1.576852
C	2.948138	-0.950036	-1.251176
C	2.704644	-0.993403	0.256511
C	3.564940	0.020519	1.009720
H	4.602602	-1.056861	-2.660085
H	5.062543	0.897528	-1.196997
H	6.368557	-0.278642	-1.049445
H	5.387601	-1.141796	1.066845
H	5.654301	0.596763	1.196132
H	2.616204	0.024173	-1.641369
H	2.333473	-1.712349	-1.738942
H	2.894558	-2.006002	0.638895
H	3.259217	1.037303	0.720637
H	3.377147	-0.073207	2.083588
H	4.746732	-2.149411	-1.283419
C	-5.722315	-0.225482	-0.275905
C	-5.558204	-1.580849	-0.560482
C	-4.289297	-2.157400	-0.451835
C	-3.194542	-1.389121	-0.059693
C	-3.351867	-0.024459	0.233065
C	-4.624546	0.543911	0.117010
H	-6.704289	0.235403	-0.359046
H	-6.410643	-2.183798	-0.865520
H	-4.151954	-3.213402	-0.673400

H	-2.201815	-1.829857	0.013441
H	-4.753452	1.601502	0.336899
C	-2.151306	0.794216	0.641008
C	-1.172196	1.012717	-0.525617
H	-1.619442	0.276202	1.448920
H	-2.459652	1.771750	1.028833
H	-0.993167	0.054775	-1.029080
H	-1.608772	1.692947	-1.262291
C	0.142459	1.570177	-0.051986
H	0.974104	1.659060	-0.733278
H	0.326272	1.771643	0.989995
Cl	-0.247240	3.871260	-0.302920

DMSO

B3LYP-D3(BJ) SCF energy: -553.19405230 a.u.
B3LYP-D3(BJ) enthalpy: -553.107513 a.u.
B3LYP-D3(BJ) free energy: -553.142435 a.u.
M06-L SCF energy in solution: -553.23015908 a.u.
M06-L enthalpy in solution: -553.143620 a.u.
M06-L free energy in solution: -553.178542 a.u.

Cartesian coordinates

ATOM	X	Y	Z
S	0.000025	0.244294	-0.440985
O	0.000333	1.506254	0.388877
C	1.357789	-0.822535	0.180147
H	1.265687	-0.930603	1.264955
H	1.322738	-1.797847	-0.314862
H	2.296990	-0.318110	-0.058679
C	-1.358098	-0.822027	0.180176
H	-1.323971	-1.796986	-0.315611
H	-1.265602	-0.931038	1.264853
H	-2.297055	-0.316772	-0.057846

15

B3LYP-D3(BJ) SCF energy: -2416.24157256 a.u.
B3LYP-D3(BJ) enthalpy: -2415.877377 a.u.
B3LYP-D3(BJ) free energy: -2415.962135 a.u.
M06-L SCF energy in solution: -2577.91570364 a.u.
M06-L enthalpy in solution: -2577.551508 a.u.
M06-L free energy in solution: -2577.636266 a.u.

Cartesian coordinates

ATOM	X	Y	Z

Zn	-0.858867	-0.202146	-0.826200
O	-0.236273	1.722559	-1.148967
S	-0.843036	3.033280	-0.589299
C	-2.623397	2.734224	-0.358977
H	-2.763562	1.904565	0.336607
H	-3.079177	3.656240	0.014094
H	-3.036347	2.465663	-1.333254
C	-0.367891	3.091328	1.166435
H	-0.845934	3.961932	1.625597
H	-0.674918	2.158174	1.645597
H	0.718211	3.199776	1.202036
S	1.172712	-1.509152	-0.738627
C	2.471815	-0.260380	-0.353061
C	2.252853	0.314745	1.044685
C	3.865920	-0.868404	-0.502910
H	2.321290	0.521213	-1.110316
C	3.308887	1.385327	1.351017
H	2.326828	-0.506039	1.767502
H	1.240409	0.720961	1.139673
C	4.935347	0.189033	-0.190122
H	3.954181	-1.711953	0.193758
H	3.984134	-1.270097	-1.513360
C	4.727348	0.815915	1.196797
H	3.166155	1.779748	2.365872
H	3.181731	2.232046	0.658489
H	5.934946	-0.258090	-0.256449
H	4.893753	0.979674	-0.953592
H	5.474155	1.599367	1.376982
H	4.883827	0.046145	1.965918
O	1.163883	-2.421709	0.469321
O	1.561824	-2.114607	-2.049490
Cl	-2.906989	-0.616033	-1.754616
O	-1.332765	0.004346	1.201739
S	-1.978372	-1.135636	2.053415
C	-1.779161	-2.686468	1.133553
H	-2.159559	-3.490879	1.770668
H	-2.334984	-2.633608	0.193008
H	-0.698743	-2.810167	0.952865
C	-3.768739	-0.909294	1.819259
H	-3.969511	-0.849398	0.745486
H	-4.291579	-1.753751	2.277508
H	-4.052603	0.018693	2.321080

3a

B3LYP-D3(BJ) SCF energy: -1133.51281672 a.u.
B3LYP-D3(BJ) enthalpy: -1133.144831 a.u.
B3LYP-D3(BJ) free energy: -1133.212293 a.u.
M06-L SCF energy in solution: -1133.57644305 a.u.
M06-L enthalpy in solution: -1133.208457 a.u.
M06-L free energy in solution: -1133.275919 a.u.

Cartesian coordinates

ATOM	X	Y	Z
S	1.283122	-0.000413	1.128857
O	1.067417	1.278314	1.832101
O	1.067412	-1.279656	1.831163
C	4.823966	1.268180	-0.665708
C	5.194598	0.000535	-1.447840
C	4.824025	-1.267653	-0.666559
C	3.337690	-1.273974	-0.275914
C	3.000378	-0.000178	0.505151
C	3.337631	1.274162	-0.275063
H	5.053415	-2.162855	-1.256347
H	4.660725	0.000846	-2.409405
H	6.265467	0.000638	-1.683152
H	5.434530	1.325747	0.246342
H	5.053317	2.163789	-1.254894
H	2.729462	-1.316799	-1.189966
H	3.089590	-2.153924	0.324310
H	3.557752	-0.000477	1.452192
H	2.729408	1.317568	-1.189090
H	3.089485	2.153698	0.325749
H	5.434593	-1.325806	0.245450
C	-5.647710	-1.206461	-0.085121
C	-6.315855	-0.000009	0.130712
C	-5.647698	1.206563	-0.084386
C	-4.320136	1.203880	-0.512648
C	-3.640018	0.000236	-0.733217
C	-4.320150	-1.203535	-0.513379
H	-6.160752	-2.150314	0.077533
H	-7.350330	-0.000108	0.462190
H	-6.160725	2.150325	0.078841
H	-3.803515	2.146381	-0.679677
H	-3.803535	-2.145938	-0.680980
C	-2.186302	0.000359	-1.145076
C	-1.248759	-0.000046	0.075896
H	-1.973550	0.881655	-1.763952
H	-1.973558	-0.880535	-1.764529

H	-1.447145	0.879918	0.696491
H	-1.447098	-0.880450	0.695879
C	0.220366	0.000144	-0.347205
H	0.470880	-0.891465	-0.929696
H	0.470855	0.892228	-0.928979

16

B3LYP-D3(BJ) SCF energy: -2996.49797838 a.u.
B3LYP-D3(BJ) enthalpy: -2995.853228 a.u.
B3LYP-D3(BJ) free energy: -2995.972368 a.u.
M06-L SCF energy in solution: -3158.19530254 a.u.
M06-L enthalpy in solution: -3157.550552 a.u.
M06-L free energy in solution: -3157.669692 a.u.

Cartesian coordinates

ATOM	X	Y	Z
Zn	0.783262	-0.131584	0.930450
O	1.954055	-0.179267	2.555100
S	2.487311	-1.372541	3.413239
C	1.129822	-2.574136	3.528757
H	0.901359	-2.964878	2.528111
H	1.457958	-3.380163	4.192627
H	0.279447	-2.052673	3.974412
C	3.591195	-2.306385	2.316211
H	4.017432	-3.133201	2.892842
H	3.011049	-2.680256	1.464383
H	4.383663	-1.620374	2.008979
S	0.484055	-2.250479	-0.199104
C	1.341282	-1.976869	-1.789417
C	2.799653	-1.602902	-1.529073
C	1.212841	-3.183505	-2.716466
H	0.826447	-1.104872	-2.210037
C	3.493723	-1.279479	-2.857842
H	3.300723	-2.441697	-1.029496
H	2.857840	-0.730626	-0.863246
C	1.918458	-2.881208	-4.048189
H	1.671880	-4.054575	-2.231555
H	0.154958	-3.416590	-2.873021
C	3.380121	-2.460040	-3.834187
H	4.548197	-1.033423	-2.679371
H	3.021410	-0.385594	-3.283526
H	1.868035	-3.759458	-4.703553
H	1.381083	-2.071378	-4.560926
H	3.842480	-2.201230	-4.794700

H	3.944589	-3.314309	-3.432144
S	0.817906	1.751276	-0.569860
C	2.110418	2.929543	-0.003003
C	3.453931	2.207768	0.111276
C	2.174583	4.122302	-0.959172
H	1.766213	3.254790	0.988063
C	4.558678	3.184049	0.541579
H	3.696572	1.775299	-0.867367
H	3.384153	1.378126	0.829348
C	3.282312	5.094495	-0.528557
H	2.378564	3.742686	-1.967978
H	1.199242	4.617464	-0.989744
C	4.638441	4.384965	-0.411261
H	5.522597	2.661543	0.580974
H	4.349889	3.540282	1.560589
H	3.346531	5.922950	-1.244461
H	3.019983	5.537696	0.443059
H	5.408368	5.087887	-0.070058
H	4.948113	4.033460	-1.405848
O	-0.949507	-2.563363	-0.520120
O	1.265471	-3.346281	0.506770
O	1.289781	1.257774	-1.909517
O	-0.474927	2.516084	-0.533408
Cl	-1.521511	0.091067	2.012545
C	-2.424507	0.143890	0.366334
C	-3.912905	0.264577	0.598483
H	-2.132209	-0.794211	-0.110624
H	-1.986534	1.013896	-0.128678
C	-4.637697	0.305385	-0.765397
H	-4.281041	-0.586709	1.181547
H	-4.141407	1.176439	1.160906
H	-4.258654	1.153083	-1.348624
H	-4.395559	-0.604226	-1.328001
C	-6.133556	0.423452	-0.588437
C	-6.930286	-0.721138	-0.463989
C	-6.745037	1.679586	-0.496455
C	-8.305208	-0.614763	-0.253362
H	-6.467572	-1.702720	-0.537495
C	-8.119596	1.790982	-0.285865
H	-6.137436	2.576240	-0.595381
C	-8.904221	0.642935	-0.163143
H	-8.909035	-1.513726	-0.164190
H	-8.578204	2.773977	-0.222053
H	-9.975287	0.727744	-0.002487

17-ts

B3LYP-D3(BJ) SCF energy: -2996.47917995 a.u.
B3LYP-D3(BJ) enthalpy: -2995.837928 a.u.
B3LYP-D3(BJ) free energy: -2995.949804 a.u.
M06-L SCF energy in solution: -3158.16966605 a.u.
M06-L enthalpy in solution: -3157.528414 a.u.
M06-L free energy in solution: -3157.640290 a.u.
Imaginary frequency: -241.1866 cm^{-1}

Cartesian coordinates

ATOM	X	Y	Z
Zn	2.130385	-0.434980	1.218205
O	4.122435	-0.345131	1.403954
S	5.131052	0.223203	0.357978
C	5.521515	-1.161421	-0.746835
H	4.594829	-1.661248	-1.063488
H	6.082689	-0.765130	-1.598786
H	6.150927	-1.848714	-0.176606
C	4.185639	1.270721	-0.790259
H	3.716928	2.046624	-0.181137
H	4.901341	1.714491	-1.489139
H	3.414007	0.704558	-1.328156
S	0.094675	1.763475	1.064073
C	-0.069947	2.132139	-0.738789
C	-1.545220	2.243197	-1.111846
C	0.708275	3.398746	-1.096044
H	0.393653	1.262959	-1.218893
C	-1.681089	2.511315	-2.618502
H	-1.996111	3.068582	-0.544046
H	-2.082101	1.327972	-0.842730
C	0.577116	3.660773	-2.605183
H	0.307106	4.242887	-0.522369
H	1.758665	3.280749	-0.814710
C	-0.893844	3.761954	-3.032484
H	-2.740783	2.616823	-2.882345
H	-1.299928	1.640504	-3.169561
H	1.114393	4.580692	-2.867583
H	1.059733	2.839201	-3.152033
H	-0.964515	3.911632	-4.116721
H	-1.346942	4.645478	-2.559912
S	1.740644	-1.457654	-0.978000
C	0.121304	-2.334010	-0.998520
C	-1.020102	-1.337053	-0.811794

C	-0.026575	-3.129251	-2.296679
H	0.177082	-3.013435	-0.138080
C	-2.382073	-2.043199	-0.846828
H	-0.967662	-0.604803	-1.625243
H	-0.895304	-0.782963	0.128877
C	-1.386199	-3.842805	-2.332038
H	0.052413	-2.430876	-3.139962
H	0.800362	-3.839222	-2.386479
C	-2.545663	-2.855909	-2.137474
H	-3.186665	-1.307296	-0.758107
H	-2.468792	-2.712817	0.021106
H	-1.498213	-4.381153	-3.281372
H	-1.417813	-4.600006	-1.535166
H	-3.505606	-3.386886	-2.127971
H	-2.576155	-2.163784	-2.991606
O	1.640102	1.503607	1.171810
O	-0.346092	3.006939	1.791959
O	1.615358	-0.359550	-2.020354
O	2.763244	-2.524087	-1.290034
C	-5.613240	0.479822	-0.811281
C	-6.180816	-0.769999	-0.559526
C	-5.837884	-1.457278	0.605510
C	-4.924024	-0.905358	1.503559
C	-4.332581	0.339664	1.252928
C	-4.700000	1.028082	0.089722
H	-5.874167	1.026758	-1.712898
H	-6.884790	-1.203796	-1.263563
H	-6.277063	-2.428616	0.814428
H	-4.665074	-1.461138	2.400241
H	-4.259699	1.999580	-0.114128
C	-3.310498	0.943020	2.193953
C	-2.347117	-0.095696	2.800990
H	-3.815237	1.470271	3.014765
H	-2.729430	1.704903	1.665560
H	-2.829294	-0.712804	3.572823
H	-2.052305	-0.826229	2.032378
C	-1.113302	0.481234	3.362142
H	-0.640377	0.004249	4.209238
H	-0.847043	1.521430	3.178349
Cl	0.725944	-1.474165	2.753356

18

B3LYP-D3(BJ) SCF energy: -1194.97215427 a.u.

B3LYP-D3(BJ) enthalpy: -1194.787282 a.u.

B3LYP-D3(BJ) free energy: -1194.858330 a.u.
M06-L SCF energy in solution: -1356.62963076 a.u.
M06-L enthalpy in solution: -1356.444758 a.u.
M06-L free energy in solution: -1356.515806 a.u.

Cartesian coordinates

ATOM	X	Y	Z
Zn	-0.000162	0.000411	0.455164
O	-1.278340	-0.959406	1.700906
O	1.279322	0.958457	1.700936
I	-1.338060	1.869697	-0.945141
I	1.337447	-1.869078	-0.945916
S	2.650792	1.604078	1.376135
S	-2.649755	-1.605212	1.376147
C	3.850862	0.237527	1.360062
H	3.509023	-0.542274	0.673811
H	4.824514	0.637173	1.060909
H	3.903561	-0.150147	2.379676
C	2.667245	1.995236	-0.401396
H	1.849627	2.695535	-0.586601
H	3.629950	2.461557	-0.631389
H	2.519614	1.085644	-0.990254
C	-3.850150	-0.238913	1.360466
H	-3.508989	0.540875	0.673874
H	-4.823908	-0.638879	1.062101
H	-3.902215	0.148943	2.380049
C	-2.666463	-1.996032	-0.401437
H	-3.629566	-2.461538	-0.631412
H	-2.518397	-1.086408	-0.990158
H	-1.849312	-2.696765	-0.586924

19

B3LYP-D3(BJ) SCF energy: -641.71948649 a.u.
B3LYP-D3(BJ) enthalpy: -641.623630 a.u.
B3LYP-D3(BJ) free energy: -641.679753 a.u.
M06-L SCF energy in solution: -803.38357542 a.u.
M06-L enthalpy in solution: -803.287719 a.u.
M06-L free energy in solution: -803.343842 a.u.

Cartesian coordinates

ATOM	X	Y	Z
Zn	0.353978	0.064808	-0.001275
O	-0.487000	1.894224	-0.000500
I	2.923124	0.167986	0.000345

I	-1.497040	-1.803784	0.000177
S	-1.922158	2.484186	0.000422
C	-2.809259	1.703390	-1.381516
H	-2.781581	0.615432	-1.278627
H	-3.837672	2.076493	-1.380378
H	-2.303344	2.015920	-2.297809
C	-2.808679	1.702948	1.382407
H	-3.837268	2.075604	1.380872
H	-2.780518	0.614993	1.279618
H	-2.303203	2.015858	2.298787

20

B3LYP-D3(BJ) SCF energy: -335.18550190 a.u.
B3LYP-D3(BJ) enthalpy: -335.006945 a.u.
B3LYP-D3(BJ) free energy: -335.070801 a.u.
M06-L SCF energy in solution: -496.87058500 a.u.
M06-L enthalpy in solution: -496.692028 a.u.
M06-L free energy in solution: -496.755884 a.u.

Cartesian coordinates

ATOM	X	Y	Z
Zn	1.090052	0.495278	0.000343
I	3.549047	-0.237810	-0.000133
I	-0.572613	2.471515	-0.000084
I	-0.347590	-2.072558	0.000059
C	-2.549619	-1.484874	0.000111
C	-2.864174	-0.724632	1.275287
C	-2.864294	-0.725225	-1.275371
H	-2.996691	-2.481304	0.000555
C	-4.357043	-0.329730	1.262178
H	-2.255095	0.183795	1.324243
H	-2.634349	-1.333478	2.154907
C	-4.357157	-0.330293	-1.262262
H	-2.255103	0.183118	-1.324758
H	-2.634584	-1.334438	-2.154765
C	-4.711296	0.464826	-0.000204
H	-4.572187	0.253570	2.164657
H	-4.976489	-1.236109	1.314157
H	-4.572473	0.252550	-2.164995
H	-4.976553	-1.236738	-1.313721
H	-5.778129	0.716802	-0.000224
H	-4.157172	1.412657	-0.000428

21

B3LYP-D3(BJ) SCF energy: -898.30866186 a.u.
B3LYP-D3(BJ) enthalpy: -898.111359 a.u.
B3LYP-D3(BJ) free energy: -898.181344 a.u.
M06-L SCF energy in solution: -1059.98164771 a.u.
M06-L enthalpy in solution: -1059.784345 a.u.
M06-L free energy in solution: -1059.854330 a.u.

Cartesian coordinates

ATOM	X	Y	Z
Zn	1.642977	0.176354	0.107404
I	3.764118	-0.884117	-0.844196
I	0.006631	2.166012	0.270413
C	-0.986198	-1.009528	2.178683
C	-1.964059	-1.161102	1.032134
H	-0.823882	0.033269	2.452513
H	-1.249805	-1.596387	3.059337
C	-3.307361	-0.486516	1.378707
H	-1.555639	-0.681437	0.137486
H	-2.118970	-2.219329	0.797223
H	-3.757377	-0.979435	2.249033
H	-3.114858	0.556160	1.660032
C	-4.255795	-0.533577	0.202709
C	-4.117451	0.384429	-0.846670
C	-5.249323	-1.514183	0.115321
C	-4.956555	0.323114	-1.958428
H	-3.347771	1.150785	-0.788184
C	-6.091469	-1.577781	-0.996014
H	-5.366828	-2.230550	0.925322
C	-5.946547	-0.659060	-2.036088
H	-4.839966	1.044336	-2.762411
H	-6.861313	-2.342666	-1.047168
H	-6.602263	-0.705707	-2.900789
Cl	0.691069	-1.638521	1.704932

22

B3LYP-D3(BJ) SCF energy: -88.46637748 a.u.
B3LYP-D3(BJ) enthalpy: -88.459570 a.u.
B3LYP-D3(BJ) free energy: -88.491429 a.u.
M06-L SCF energy in solution: -250.14720410 a.u.
M06-L enthalpy in solution: -250.140397 a.u.
M06-L free energy in solution: -250.172256 a.u.

Cartesian coordinates

ATOM	X	Y	Z

Zn	0.000000	-0.000361	0.000012
I	-2.203142	-0.039501	-1.243529
I	2.203142	0.039706	1.243522

23

B3LYP-D3(BJ) SCF energy: -1283.48921199 a.u.

B3LYP-D3(BJ) enthalpy: -1283.295574 a.u.

B3LYP-D3(BJ) free energy: -1283.387945 a.u.

M06-L SCF energy in solution: -1606.78767214 a.u.

M06-L enthalpy in solution: -1606.594034 a.u.

M06-L free energy in solution: -1606.686405 a.u.

Cartesian coordinates

ATOM	X	Y	Z
Zn	1.536731	-0.384704	-0.373778
Zn	-2.148712	0.106622	-0.093351
I	-0.122332	-0.272585	1.914783
I	3.459206	1.437915	-0.188821
I	-0.370696	-0.145060	-2.313002
I	-4.236720	-1.400978	0.087810
O	2.241991	-2.264149	-0.365077
O	-2.493481	2.086412	-0.126443
S	-1.802538	3.428134	0.218680
S	3.390972	-2.926360	0.441520
C	-0.029498	3.252056	-0.139773
H	0.451134	4.214595	0.056996
H	0.416340	2.472723	0.479343
H	0.068556	2.994808	-1.196027
C	-1.703929	3.481945	2.033038
H	-1.187782	2.592082	2.400999
H	-1.172784	4.393415	2.322681
H	-2.729429	3.511087	2.407289
C	4.935316	-2.327169	-0.307597
H	4.942801	-1.234050	-0.320706
H	5.774384	-2.725883	0.270380
H	4.965030	-2.720118	-1.326173
C	3.501442	-2.054537	2.034127
H	2.550140	-2.204743	2.549066
H	4.315879	-2.505944	2.608313
H	3.680900	-0.988219	1.871046

24

B3LYP-D3(BJ) SCF energy: -976.95497338 a.u.

B3LYP-D3(BJ) enthalpy: -976.678723 a.u.

B3LYP-D3(BJ) free energy: -976.779070 a.u.
M06-L SCF energy in solution: -1300.27530280 a.u.
M06-L enthalpy in solution: -1299.999052 a.u.
M06-L free energy in solution: -1300.099399 a.u.

Cartesian coordinates

ATOM	X	Y	Z
Zn	-0.919616	0.361170	-0.342835
Zn	2.685755	-0.150378	0.373277
I	1.243094	0.427246	-2.060877
I	-2.496326	2.442934	-0.178138
I	0.343567	-0.421991	1.981122
I	4.403362	-2.067449	0.238863
O	3.359903	1.629811	1.010446
I	-2.286761	-1.998090	-1.278144
S	3.062464	3.145330	0.904797
C	1.258924	3.354664	0.808812
H	1.044060	4.426968	0.795480
H	0.860781	2.878705	-0.088125
H	0.827029	2.898676	1.701624
C	3.477943	3.614429	-0.801488
H	2.921909	2.988451	-1.503310
H	3.232453	4.671724	-0.937947
H	4.552498	3.461169	-0.921504
C	-4.079093	-1.784794	0.109667
C	-3.600551	-1.339645	1.479146
C	-5.089615	-0.848655	-0.526808
H	-4.427232	-2.819606	0.120677
C	-4.821997	-1.198403	2.411270
H	-3.110009	-0.362834	1.400943
H	-2.873406	-2.044876	1.891366
C	-6.298388	-0.704059	0.423621
H	-4.634136	0.134911	-0.684038
H	-5.409918	-1.229645	-1.501170
C	-5.860576	-0.239480	1.817649
H	-4.475322	-0.845642	3.389102
H	-5.276478	-2.186001	2.571839
H	-7.007976	0.004136	-0.019055
H	-6.819463	-1.668542	0.503026
H	-6.729876	-0.166016	2.481422
H	-5.425240	0.766079	1.745121

25

B3LYP-D3(BJ) SCF energy: -1540.08073574 a.u.

B3LYP-D3(BJ) enthalpy: -1539.785755 a.u.
B3LYP-D3(BJ) free energy: -1539.891136 a.u.
M06-L SCF energy in solution: -1863.37812236 a.u.
M06-L enthalpy in solution: -1863.083142 a.u.
M06-L free energy in solution: -1863.188523 a.u.

Cartesian coordinates

ATOM	X	Y	Z
Zn	0.301718	1.184990	-0.229889
Zn	-2.399008	-0.286371	0.347927
I	-2.100770	2.461023	0.451355
I	2.023771	1.426733	-2.191516
I	0.048975	-1.048161	1.608617
I	-4.522072	-1.754353	0.277811
O	-1.160864	-0.168055	-1.367261
C	2.726225	1.500477	2.514374
C	3.551092	0.688304	1.535563
H	2.047172	0.878250	3.098379
H	3.333621	2.114698	3.180954
C	4.327357	-0.427079	2.262437
H	2.893132	0.225639	0.795485
H	4.237048	1.335267	0.979630
H	5.066317	0.012453	2.943346
H	3.625602	-1.003845	2.878563
C	5.006799	-1.343823	1.271474
C	4.264419	-2.328809	0.606470
C	6.362390	-1.198531	0.960720
C	4.865516	-3.150120	-0.345939
H	3.209061	-2.449776	0.840956
C	6.967789	-2.019399	0.007720
H	6.948505	-0.437045	1.470018
C	6.220494	-2.997632	-0.648588
H	4.277756	-3.912987	-0.849353
H	8.022787	-1.894925	-0.220067
H	6.690444	-3.638484	-1.389205
S	-0.475457	-1.293159	-2.244010
C	-1.581494	-2.723025	-2.105830
H	-2.615386	-2.425141	-2.291854
H	-1.244297	-3.473214	-2.827223
H	-1.483595	-3.114136	-1.091078
C	-0.878066	-0.757818	-3.925109
H	-0.549703	-1.530460	-4.626132
H	-1.953915	-0.582169	-3.995678
H	-0.318011	0.164599	-4.090440

Cl	1.634491	2.696151	1.643704

26

B3LYP-D3(BJ) SCF energy: -670.42543446 a.u.

B3LYP-D3(BJ) enthalpy: -670.066515 a.u.

B3LYP-D3(BJ) free energy: -670.173612 a.u.

M06-L SCF energy in solution: -993.76539398 a.u.

M06-L enthalpy in solution: -993.406475 a.u.

M06-L free energy in solution: -993.513572 a.u.

Cartesian coordinates

ATOM	X	Y	Z
Zn	-1.218838	0.184707	-0.474599
Zn	2.224618	-1.011018	0.120501
I	0.870366	-0.222756	-2.231637
I	-2.770420	2.272561	-0.534910
I	0.130135	-0.374985	1.895114
I	3.641849	-3.146773	0.288554
I	-2.686977	-2.265440	-0.947592
I	3.802321	1.416473	0.480304
C	1.974171	2.747206	0.222814
C	1.731128	3.521623	1.501050
C	2.143862	3.606181	-1.012408
H	1.207251	1.987413	0.072334
C	0.457441	4.374078	1.311756
H	2.588508	4.173007	1.709993
H	1.611191	2.839019	2.347185
C	0.866671	4.457248	-1.184404
H	3.015387	4.261323	-0.893048
H	2.308522	2.982360	-1.895559
C	0.560875	5.276056	0.075749
H	0.295029	4.969822	2.217549
H	-0.409345	3.709940	1.207305
H	0.994723	5.111925	-2.054198
H	0.018947	3.796934	-1.405316
H	-0.373999	5.831691	-0.059069
H	1.355696	6.019888	0.230739
C	-4.405095	-1.811237	0.475993
C	-3.854518	-1.174173	1.738295
C	-5.434663	-0.962766	-0.246968
H	-4.764107	-2.825911	0.659450
C	-5.027211	-0.870125	2.694108
H	-3.355829	-0.230662	1.489292
H	-3.117430	-1.823850	2.218520

C	-6.593736	-0.653959	0.725909
H	-4.976148	-0.022684	-0.571983
H	-5.806456	-1.479818	-1.136600
C	-6.082641	0.007484	2.011212
H	-4.629132	-0.380513	3.590015
H	-5.484751	-1.813327	3.023634
H	-7.316205	-0.007731	0.214648
H	-7.121054	-1.585509	0.975069
H	-6.917246	0.196524	2.696262
H	-5.640237	0.982505	1.766945

27

B3LYP-D3(BJ) SCF energy: -1796.66638249 a.u.
B3LYP-D3(BJ) enthalpy: -1796.269854 a.u.
B3LYP-D3(BJ) free energy: -1796.390536 a.u.
M06-L SCF energy in solution: -2119.98187723 a.u.
M06-L enthalpy in solution: -2119.585349 a.u.
M06-L free energy in solution: -2119.706031 a.u.

Cartesian coordinates

ATOM	X	Y	Z
Zn	0.086840	0.994650	-1.312767
Zn	-2.786380	-0.288004	0.502588
I	-2.191225	-0.211526	-2.248415
I	2.484311	0.069811	-1.744143
I	-0.674805	1.350850	1.331462
I	-5.149432	-0.328144	1.492444
C	1.692578	3.877446	-2.084788
C	2.012142	3.883856	-0.604910
H	1.734808	4.862480	-2.550416
H	2.300786	3.164449	-2.642487
C	3.513927	4.127752	-0.357841
H	1.408800	4.632939	-0.082031
H	1.757545	2.912508	-0.171728
H	4.089855	3.408549	-0.953518
H	3.794121	5.131678	-0.697919
C	3.837659	3.956480	1.109248
C	4.033011	5.061309	1.943195
C	3.889940	2.670560	1.664601
C	4.278199	4.889064	3.306864
H	3.994230	6.063421	1.522328
C	4.132146	2.495975	3.025974
H	3.741134	1.804528	1.023218
C	4.326822	3.605843	3.851637

H	4.430734	5.757324	3.941762
H	4.171470	1.493409	3.442482
H	4.516424	3.470184	4.912502
C	0.018133	-2.485621	0.280786
C	0.791538	-3.750683	0.590464
H	0.471232	-1.595432	0.717509
H	-0.143481	-2.337096	-0.787213
C	2.207837	-3.670962	-0.020323
H	0.865517	-3.888514	1.674495
H	0.264549	-4.621726	0.186559
H	2.129335	-3.520224	-1.103102
H	2.726038	-2.791050	0.377643
C	2.999015	-4.923147	0.277518
C	3.758859	-5.024933	1.448891
C	2.949094	-6.022051	-0.588293
C	4.453394	-6.196711	1.749539
H	3.810019	-4.176027	2.127057
C	3.641963	-7.195975	-0.291989
H	2.366753	-5.953543	-1.504382
C	4.396034	-7.286612	0.879216
H	5.042432	-6.257011	2.660458
H	3.596489	-8.037626	-0.977474
H	4.938628	-8.198842	1.110052
Cl	-1.678767	-2.568968	1.015510
Cl	-0.054410	3.316860	-2.372014

28

B3LYP-D3(BJ) SCF energy: -2186.64249075 a.u.
B3LYP-D3(BJ) enthalpy: -2186.188427 a.u.
B3LYP-D3(BJ) free energy: -2186.278453 a.u.
M06-L SCF energy in solution: -2348.34757525 a.u.
M06-L enthalpy in solution: -2347.893511 a.u.
M06-L free energy in solution: -2347.983537 a.u.

Cartesian coordinates

ATOM	X	Y	Z
Zn	-0.199648	0.891425	-0.962409
O	0.227117	2.696062	-0.203480
S	-0.502886	3.599467	0.846317
C	-2.174187	3.888659	0.200047
H	-2.707748	2.932002	0.128229
H	-2.683089	4.566957	0.892189
H	-2.058156	4.374023	-0.771738
C	-0.925815	2.498476	2.226843

H	-1.433954	3.094110	2.991350
H	-1.582929	1.702614	1.855510
H	0.014632	2.110395	2.624759
S	-2.484689	0.183856	-0.665647
C	-2.179079	-1.534112	-0.108651
C	-1.343006	-1.512476	1.169948
C	-3.480918	-2.312566	0.068133
H	-1.579602	-1.964453	-0.921397
C	-1.003655	-2.947745	1.590406
H	-1.911961	-1.001957	1.956314
H	-0.410602	-0.951503	1.011879
C	-3.156813	-3.751401	0.501570
H	-4.090810	-1.814248	0.832638
H	-4.050375	-2.297228	-0.866135
C	-2.282401	-3.781327	1.764104
H	-0.427582	-2.935590	2.524118
H	-0.358838	-3.389767	0.821283
H	-4.087920	-4.305933	0.670277
H	-2.628724	-4.260115	-0.316792
H	-2.025141	-4.816445	2.019715
H	-2.860246	-3.381344	2.610198
S	1.575421	-0.614656	-1.525265
C	3.006020	-0.129931	-0.472216
C	2.558795	0.014658	0.981974
C	4.127778	-1.158758	-0.629797
H	3.321509	0.842667	-0.872493
C	3.748079	0.378957	1.883015
H	2.119442	-0.936559	1.306555
H	1.776971	0.784961	1.066494
C	5.315233	-0.796312	0.274015
H	3.732259	-2.144183	-0.354445
H	4.427976	-1.211176	-1.680689
C	4.881785	-0.646137	1.738839
H	3.419072	0.441943	2.927867
H	4.119765	1.375794	1.606013
H	6.094798	-1.562371	0.184693
H	5.760443	0.147868	-0.071325
H	5.735880	-0.353418	2.361528
H	4.534433	-1.619356	2.113806
O	-3.350339	0.134819	-1.877665
O	-3.041844	0.911478	0.544466
O	1.085610	-1.914892	-0.955134
O	2.063646	-0.593594	-2.932438

29

B3LYP-D3(BJ) SCF energy: -1880.10420796 a.u.

B3LYP-D3(BJ) enthalpy: -1879.567349 a.u.

B3LYP-D3(BJ) free energy: -1879.665523 a.u.

M06-L SCF energy in solution: -2041.83110835 a.u.

M06-L enthalpy in solution: -2041.294249 a.u.

M06-L free energy in solution: -2041.392423 a.u.

Cartesian coordinates

ATOM	X	Y	Z
Zn	-0.050231	0.056571	-1.197463
S	1.998118	1.303738	-1.071089
C	1.256209	2.830959	-0.374203
C	0.647168	2.518012	0.990925
C	2.274845	3.967369	-0.306459
H	0.453310	3.076910	-1.082797
C	-0.037861	3.768287	1.556121
H	1.442334	2.170341	1.661386
H	-0.092861	1.709423	0.906738
C	1.600084	5.223542	0.267941
H	3.106843	3.656077	0.337879
H	2.684649	4.156154	-1.303388
C	0.947190	4.944572	1.629718
H	-0.445627	3.549349	2.550652
H	-0.887963	4.019969	0.910344
H	2.337752	6.030130	0.356838
H	0.833095	5.571943	-0.437570
H	0.433724	5.843137	1.992495
H	1.732457	4.711160	2.363412
S	-2.321861	0.826407	-1.256720
C	-3.190915	-0.182335	0.011323
C	-2.424910	-0.122469	1.330378
C	-4.636389	0.293562	0.165371
H	-3.169117	-1.202356	-0.395463
C	-3.132319	-0.962466	2.402438
H	-2.361123	0.924492	1.649638
H	-1.392441	-0.479531	1.194896
C	-5.350040	-0.536279	1.243561
H	-4.623529	1.352785	0.450613
H	-5.149924	0.221341	-0.798004
C	-4.588839	-0.507444	2.576714
H	-2.590415	-0.887497	3.353148
H	-3.110876	-2.020747	2.105476
H	-6.371416	-0.162063	1.381193

H	-5.439660	-1.576492	0.899079
H	-5.094646	-1.138652	3.317124
H	-4.597367	0.517061	2.974805
O	2.555711	1.635086	-2.411078
O	2.936495	0.777754	-0.024755
O	-2.217291	2.208399	-0.684853
O	-3.058881	0.629774	-2.535135
I	0.111135	-2.831833	-0.880334
C	1.724381	-2.699176	0.759855
C	3.080039	-2.514027	0.115542
C	1.349621	-1.598640	1.730281
H	1.595986	-3.693288	1.192540
C	4.137382	-2.479121	1.245564
H	3.110270	-1.559640	-0.418197
H	3.294888	-3.323881	-0.587334
C	2.415888	-1.557849	2.849183
H	1.363507	-0.633807	1.211543
H	0.353954	-1.763496	2.152970
C	3.824229	-1.381057	2.269087
H	5.119709	-2.314708	0.789320
H	4.175322	-3.458883	1.743100
H	2.167675	-0.736621	3.531419
H	2.361155	-2.486459	3.434828
H	4.564730	-1.399583	3.077433
H	3.889420	-0.405626	1.777001

30

B3LYP-D3(BJ) SCF energy: -2443.23308832 a.u.
B3LYP-D3(BJ) enthalpy: -2442.677228 a.u.
B3LYP-D3(BJ) free energy: -2442.778108 a.u.
M06-L SCF energy in solution: -2604.94815488 a.u.
M06-L enthalpy in solution: -2604.392295 a.u.
M06-L free energy in solution: -2604.493175 a.u.

Cartesian coordinates

ATOM	X	Y	Z
Zn	0.275460	1.977057	-0.348785
S	2.516452	1.181985	-0.710362
C	3.012731	0.599119	0.967433
C	1.800463	-0.020478	1.666209
C	4.186662	-0.374683	0.837859
H	3.325953	1.513375	1.486458
C	2.190526	-0.621386	3.024871
H	1.383330	-0.807832	1.026276

H	1.010499	0.726678	1.833571
C	4.561687	-0.953409	2.209996
H	3.893941	-1.185517	0.160577
H	5.037605	0.138466	0.378178
C	3.352128	-1.612954	2.884750
H	1.314269	-1.105910	3.470543
H	2.481942	0.190204	3.705081
H	5.376928	-1.676777	2.090244
H	4.944154	-0.150282	2.855536
H	3.631805	-2.007406	3.868840
H	3.025952	-2.472171	2.279955
S	-1.591471	2.339388	1.097368
C	-2.667582	0.886893	0.766305
C	-1.903241	-0.402125	1.060984
C	-3.957808	0.986782	1.582812
H	-2.893757	0.965973	-0.306489
C	-2.784302	-1.625467	0.780541
H	-1.593070	-0.388061	2.112350
H	-0.983429	-0.464358	0.464223
C	-4.843162	-0.239933	1.312764
H	-3.691941	1.031804	2.646307
H	-4.478229	1.916966	1.336347
C	-4.091429	-1.551622	1.581306
H	-2.230612	-2.539395	1.015456
H	-3.012082	-1.671131	-0.293511
H	-5.747378	-0.183439	1.930526
H	-5.176074	-0.220199	0.264916
H	-4.729706	-2.410682	1.341404
H	-3.859246	-1.620111	2.653694
O	3.537694	2.158585	-1.172908
O	2.317692	-0.066682	-1.528570
O	-1.064301	2.167556	2.486082
O	-2.387132	3.552473	0.765491
Cl	-0.688783	2.211200	-2.793064
C	-0.125564	0.561642	-3.433185
C	-0.559637	-0.534842	-2.484852
H	0.959584	0.634777	-3.489325
H	-0.572154	0.492822	-4.425546
C	-0.023444	-1.909931	-2.927395
H	-0.127947	-0.329815	-1.500972
H	-1.649549	-0.556887	-2.378571
H	-0.536428	-2.235633	-3.839978
H	1.041557	-1.805030	-3.159212
C	-0.202733	-2.926659	-1.822197

C	0.692490	-2.933940	-0.742363
C	-1.267341	-3.832619	-1.823968
C	0.519378	-3.825575	0.315182
H	1.525407	-2.236420	-0.750722
C	-1.437727	-4.732641	-0.769441
H	-1.965228	-3.838349	-2.658136
C	-0.546926	-4.729411	0.304581
H	1.222503	-3.822175	1.144072
H	-2.266809	-5.434484	-0.787500
H	-0.678627	-5.429072	1.125016

Zn14

B3LYP-D3(BJ) SCF energy: -918.54714412 a.u.

B3LYP-D3(BJ) enthalpy: -918.508878 a.u.

B3LYP-D3(BJ) free energy: -918.622471 a.u.

M06-L SCF energy in solution: -3180.39032183 a.u.

M06-L enthalpy in solution: -3180.352056 a.u.

M06-L free energy in solution: -3180.465649 a.u.

Cartesian coordinates

ATOM	X	Y	Z
Zn	5.479246	0.000964	0.003065
Zn	2.886641	-1.405593	-1.262135
Zn	2.883476	-0.391734	1.847948
Zn	0.002177	-2.762290	-2.472269
Zn	-0.002485	-0.760755	3.627839
Zn	-0.001592	-1.901284	0.623460
Zn	2.886524	1.794907	-0.587889
Zn	-0.002524	0.412178	-1.958604
Zn	0.000785	1.490269	1.333689
Zn	0.001378	3.522386	-1.154451
Zn	-5.476565	-0.001540	-0.003740
Zn	-2.887289	-0.395725	1.849597
Zn	-2.883199	-1.402195	-1.268894
Zn	-2.886574	1.800412	-0.577616

Zn

B3LYP-D3(BJ) SCF energy: -65.59580510 a.u.

B3LYP-D3(BJ) enthalpy: -65.593445 a.u.

B3LYP-D3(BJ) free energy: -65.611682 a.u.

M06-L SCF energy in solution: -227.16585970 a.u.

M06-L enthalpy in solution: -227.163500 a.u.

M06-L free energy in solution: -227.181737 a.u.

Cartesian coordinates

ATOM	X	Y	Z
Zn	0.000000	0.000000	0.000000

Analytical Data of Substrates and Products

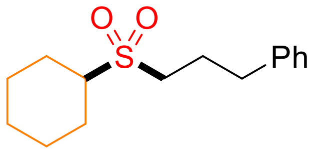

(3-(cyclohexylsulfonyl)propyl)benzene (3a): Following the General Procedure B, purified by preparative TLC, using petroleum ether /EA = 10/1 (v/v) as an eluent. White solid in 82% yield.

^{1}H NMR (400 MHz, $CDCl_3$) δ 7.22 (dd, J = 8.1, 6.6 Hz, 2H), 7.17 – 7.08 (m, 3H), 2.84 – 2.77 (m, 2H), 2.77 – 2.67 (m, 3H), 2.14 – 2.05 (m, 2H), 2.05 – 1.97 (m, 2H), 1.83 (dt, J = 12.9, 3.2 Hz, 2H), 1.67 – 1.60 (m, 1H), 1.43 (qd, J = 12.4, 3.5 Hz, 2H), 1.26 – 1.08 (m, 3H).

^{13}C NMR (101 MHz, $CDCl_3$) δ 139.89, 128.52, 128.34, 126.34, 60.72, 48.28, 34.26, 24.94, 24.85, 22.77.

HRMS (ESI/QTOF) m/z: $[M + Na]^+$ Calcd for $C_{15}H_{22}O_2SNa^+$ 289.1233; Found 289.1248.

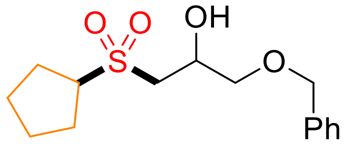

1-(benzyloxy)-3-(cyclopentylsulfonyl)propan-2-ol (3b): Following the General Procedure A, purified by preparative TLC, using petroleum ether /EA = 2/1 (v/v) as an eluent. White solid in 49% yield.

^{1}H NMR (400 MHz, $CDCl_3$) δ 7.38 – 7.21 (m, 5H), 4.59 – 4.48 (m, 2H), 4.41 (dtd, J = 8.3, 5.1, 2.6 Hz, 1H), 3.62 – 3.52 (m, 1H), 3.50 (d, J = 5.3 Hz, 2H), 3.43 (s, 1H), 3.17 (dd, J = 14.5, 9.0 Hz, 1H), 3.06 (dd, J = 14.6, 2.7 Hz, 1H), 2.14 – 2.04 (m, 1H), 2.03 – 1.91 (m, 3H), 1.83 – 1.71 (m, 2H), 1.68 – 1.56 (m, 2H).

^{13}C NMR (101 MHz, $CDCl_3$) δ 137.31, 128.22, 127.65, 127.54, 73.14, 72.47, 65.27, 61.98, 54.72, 27.03, 25.75, 25.68.

HRMS (ESI/QTOF) m/z: $[M + Na]^+$ Calcd for $C_{15}H_{22}O_4SNa^+$ 321.1131; Found 321.1141.

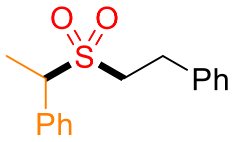

(1-(phenethylsulfonyl)ethyl)benzene (3c): Following the General Procedure A, purified by preparative TLC, using petroleum ether /EA = 10/1 (v/v) as an eluent. White solid in 65% yield.

^{1}H NMR (400 MHz, $CDCl_3$) δ 7.44 – 7.36 (m, 5H), 7.30 – 7.19 (m, 3H), 7.11 – 7.06 (m, 2H), 4.16 (q, *J* = 7.1 Hz, 1H), 3.11 – 2.89 (m, 4H), 1.80 (d, *J* = 7.1 Hz, 3H).

^{13}C NMR (101 MHz, $CDCl_3$) δ 137.78, 134.31, 129.16, 129.06, 128.92, 128.80, 128.42, 126.93, 63.69, 51.53, 27.74, 13.70.

HRMS (ESI/QTOF) m/z: $[M + Na]^+$ Calcd for $C_{16}H_{18}O_2SNa^+$ 297.0920; Found 297.0933.

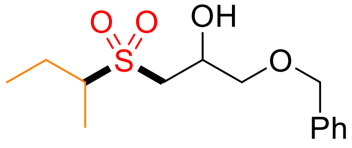

1-(benzyloxy)-3-(*sec*-butylsulfonyl)propan-2-ol (3d): Following the General Procedure A, purified by preparative TLC, using petroleum ether /EA = 2/1 (v/v) as an eluent. White solid in 67% yield.

^{1}H NMR (400 MHz, $CDCl_3$) δ 7.40 – 7.27 (m, 5H), 4.61 – 4.53 (m, 2H), 4.46 (dp, *J* = 8.1, 2.7 Hz, 1H), 3.55 (t, *J* = 4.6 Hz, 2H), 3.22 (ddd, *J* = 14.3, 9.0, 5.4 Hz, 1H), 3.06 (ddd, *J* = 19.5, 14.5, 2.7 Hz, 2H), 2.15 – 2.01 (m, 1H), 1.57 (dddd, *J* = 13.8, 10.0, 6.1, 2.6 Hz, 1H), 1.38 (dd, *J* = 6.9, 1.3 Hz, 3H), 1.05 (td, *J* = 7.5, 3.1 Hz, 3H).

^{13}C NMR (101 MHz, $CDCl_3$) δ 137.42 (d, *J* = 1.4 Hz), 128.51, 127.98, 127.80, 73.50 (d, *J* = 2.0 Hz), 72.51 (d, *J* = 3.6 Hz), 65.43 (d, *J* = 6.9 Hz), 60.16 (d, *J* = 11.1 Hz), 53.09 (d, *J* = 11.1 Hz), 21.93 (d, *J* = 104.3 Hz), 12.08 (d, *J* = 123.0 Hz), 11.07 (d, *J* = 9.8 Hz).

HRMS (ESI/QTOF) m/z: $[M + Na]^+$ Calcd for $C_{14}H_{22}O_4SNa^+$ 309.1131; Found 309.1142.

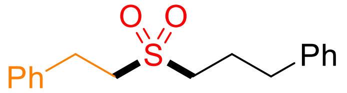

(3-(phenethylsulfonyl)propyl)benzene (3e): Following the General Procedure A, purified by preparative TLC, using petroleum ether /EA = 10/1 (v/v) as an eluent. White solid in 57% yield.

^{1}H NMR (400 MHz, $CDCl_3$) δ 7.36 – 7.15 (m, 10H), 3.23 – 3.16 (m, 2H), 3.14 – 3.08 (m, 2H), 2.89 – 2.83 (m, 2H), 2.72 (t, *J* = 7.5 Hz, 2H), 2.18 – 2.09 (m, 2H).

^{13}C NMR (101 MHz, $CDCl_3$) δ 139.60, 137.34, 128.58, 128.34, 128.13, 126.74, 126.17, 53.63, 51.92, 33.84, 27.68, 23.20.

HRMS (ESI/QTOF) m/z: $[M + Na]^+$ Calcd for $C_{17}H_{20}O_2SNa^+$ 311.1076; Found 311.1067.

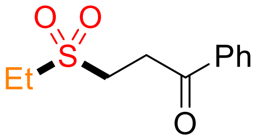

3-(ethylsulfonyl)-1-phenylpropan-1-one (3f): Following the General Procedure A, purified by preparative TLC, using petroleum ether /EA = 5/1 (v/v) as an eluent. White solid in 54% yield.

^{1}H NMR (400 MHz, $CDCl_3$) δ 7.91 – 7.85 (m, 2H), 7.54 – 7.48 (m, 1H), 7.39 (t, J = 7.7 Hz, 2H), 3.47 (dd, J = 7.6, 6.2 Hz, 2H), 3.34 (t, J = 7.5 Hz, 2H), 2.98 (q, J = 7.5 Hz, 2H), 1.34 (t, J = 7.5 Hz, 3H).

^{13}C NMR (101 MHz, $CDCl_3$) δ 195.66, 135.58, 133.65, 128.62, 127.93, 47.86, 46.19, 30.53, 6.46.

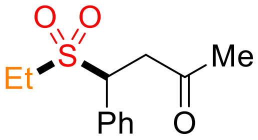

4-(ethylsulfonyl)-4-phenylbutan-2-one (3g): Following the General Procedure A, purified by preparative TLC, using petroleum ether /EA = 5/1 (v/v) as an eluent. White solid in 50% yield.

^{1}H NMR (400 MHz, $CDCl_3$) δ 7.39 (dd, J = 7.6, 2.1 Hz, 2H), 7.33 – 7.26 (m, 3H), 4.67 (dd, J = 9.0, 4.1 Hz, 1H), 3.48 (dd, J = 18.0, 4.1 Hz, 1H), 3.13 (dd, J = 18.0, 9.0 Hz, 1H), 2.75 – 2.55 (m, 2H), 2.07 (s, 3H), 1.18 (t, J = 7.5 Hz, 3H).

^{13}C NMR (101 MHz, $CDCl_3$) δ 203.18, 133.05, 129.19, 128.96, 128.89, 62.14, 44.70, 41.02, 30.21, 5.91.

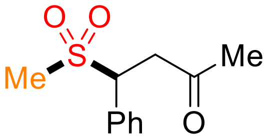

4-(methylsulfonyl)-4-phenylbutan-2-one (3h): Following the General Procedure A, purified by preparative TLC, using petroleum ether /EA = 5/1 (v/v) as an eluent. White solid in 45% yield.

^{1}H NMR (400 MHz, $CDCl_3$) δ 7.39 (dd, J = 7.4, 2.3 Hz, 2H), 7.33 – 7.26 (m, 3H), 4.68 (dd, J = 8.9, 4.2 Hz, 1H), 3.47 (dd, J = 18.0, 4.2 Hz, 1H), 3.14 (dd, J = 18.0, 8.9 Hz, 1H), 2.56 (s, 3H), 2.07 (s, 3H).

^{13}C NMR (101 MHz, $CDCl_3$) δ 203.09, 133.02, 129.21, 129.02, 128.88, 64.22, 40.85, 38.27, 30.15.

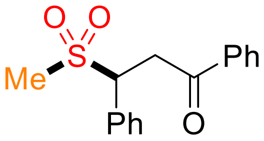

4-(methylsulfonyl)-4-phenylbutan-2-one (3i): Following the General Procedure A, purified by preparative TLC, using petroleum ether /EA = 5/1 (v/v) as an eluent. White solid in 42% yield.

^{1}H NMR (400 MHz, $CDCl_3$) δ 7.39 (dd, *J* = 7.4, 2.3 Hz, 2H), 7.33 – 7.26 (m, 3H), 4.68 (dd, *J* = 8.9, 4.2 Hz, 1H), 3.47 (dd, *J* = 18.0, 4.2 Hz, 1H), 3.14 (dd, *J* = 18.0, 8.9 Hz, 1H), 2.56 (s, 3H), 2.07 (s, 3H).

^{13}C NMR (101 MHz, $CDCl_3$) δ 203.09, 133.02, 129.21, 129.02, 128.88, 64.22, 40.85, 38.27, 30.15.

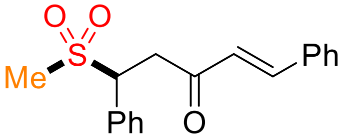

(*E*)-5-(methylsulfonyl)-1,5-diphenylpent-1-en-3-one (3j): Following the General Procedure A, purified by preparative TLC, using petroleum ether /EA = 5/1 (v/v) as an eluent. White solid in 35% yield.

^{1}H NMR (400 MHz, $CDCl_3$) δ 7.50 (d, *J* = 16.2 Hz, 1H), 7.44 (ddt, *J* = 6.6, 4.7, 2.4 Hz, 4H), 7.30 (tdd, *J* = 7.2, 5.0, 2.0 Hz, 6H), 6.61 (d, *J* = 16.3 Hz, 1H), 4.80 (dd, *J* = 9.4, 3.8 Hz, 1H), 3.71 (dd, *J* = 17.6, 3.8 Hz, 1H), 3.45 (dd, *J* = 17.7, 9.4 Hz, 1H), 2.60 (s, 3H).

^{13}C NMR (101 MHz, $CDCl_3$) δ 194.49, 144.02, 133.91, 133.18, 130.85, 129.41, 129.20, 129.06, 128.94, 128.38, 125.38, 64.75, 38.65, 38.29.

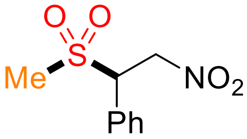

(1-(methylsulfonyl)-2-nitroethyl)benzene (3k): Following the General Procedure A, purified by preparative TLC, using petroleum ether /EA = 5/1 (v/v) as an eluent. White solid in 38% yield.

^{1}H NMR (400 MHz, $CDCl_3$) δ 7.40 (q, *J* = 3.1 Hz, 5H), 5.27 (q, *J* = 9.7 Hz, 1H), 4.95 (d, *J* = 8.9 Hz, 2H), 2.66 (s, 3H).

^{13}C NMR (101 MHz, $CDCl_3$) δ 130.47, 129.70, 129.36, 129.16, 72.41, 66.51, 39.19.

HRMS (ESI/QTOF) m/z: $[M + Na]^+$ Calcd for $C_9H_{11}NO_4SNa^+$ 252.0301; Found 252.0297.

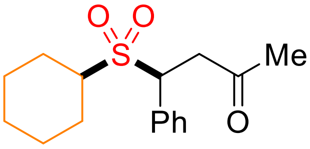

4-(cyclohexylsulfonyl)-4-phenylbutan-2-one (3l): Following the General Procedure A, purified

by preparative TLC, using petroleum ether /EA = 5/1 (v/v) as an eluent. White solid in 53% yield.

^{1}H NMR (400 MHz, $CDCl_3$) δ 7.40 (dd, *J* = 7.6, 2.0 Hz, 2H), 7.34 – 7.26 (m, 3H), 4.76 (dd, *J* = 9.1, 3.9 Hz, 1H), 3.47 (dd, *J* = 18.0, 3.9 Hz, 1H), 3.11 (dd, *J* = 18.0, 9.1 Hz, 1H), 2.47 (tt, *J* = 12.1, 3.5 Hz, 1H), 2.07 (s, 4H), 1.90 (dt, *J* = 14.0, 3.6 Hz, 1H), 1.79 – 1.72 (m, 2H), 1.60 – 1.52 (m, 1H), 1.43 (dqd, *J* = 21.7, 12.5, 3.5 Hz, 2H), 1.05 (dddd, *J* = 16.8, 14.3, 8.2, 2.2 Hz, 3H).

^{13}C NMR (101 MHz, $CDCl_3$) δ 203.50, 133.35, 129.26, 128.98, 128.94, 59.63, 57.82, 41.47, 30.42, 26.29, 24.91, 24.88, 24.65, 22.98.

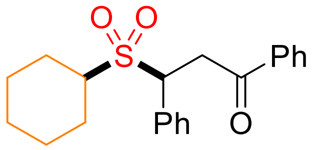

3-(cyclohexylsulfonyl)-1,3-diphenylpropan-1-one (3m): Following the General Procedure A, purified by preparative TLC, using petroleum ether /EA = 5/1 (v/v) as an eluent. White solid in 53% yield.

^{1}H NMR (400 MHz, $CDCl_3$) δ 7.86 (d, *J* = 7.7 Hz, 2H), 7.48 (d, *J* = 6.9 Hz, 3H), 7.40 – 7.26 (m, 5H), 4.97 (dd, *J* = 9.8, 3.3 Hz, 1H), 3.98 (dd, *J* = 17.9, 3.3 Hz, 1H), 3.76 (dd, *J* = 17.9, 9.7 Hz, 1H), 2.52 (tt, *J* = 11.9, 3.4 Hz, 1H), 2.10 (d, *J* = 13.0 Hz, 1H), 1.98 – 1.89 (m, 1H), 1.81 – 1.72 (m, 2H), 1.59 – 1.42 (m, 3H), 1.06 (dt, *J* = 24.1, 12.7 Hz, 3H).

^{13}C NMR (101 MHz, $CDCl_3$) δ 195.12, 136.07, 133.58, 133.46, 129.40, 128.98, 128.92, 128.65, 128.08, 60.02, 57.89, 37.13, 26.36, 24.95, 24.70, 23.04.

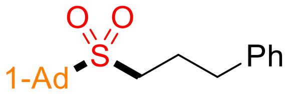

(3*S*,5*S*,7*S*)-1-((3-phenylpropyl)sulfonyl)adamantane (3n): Following the General Procedure A, purified by preparative TLC, using petroleum ether /EA = 10/1 (v/v) as an eluent. White solid in 67% yield.

^{1}H NMR (400 MHz, DMSO-*d*$_6$) δ 7.28 (t, *J* = 7.6 Hz, 2H), 7.22 – 7.15 (m, 3H), 2.99 – 2.92 (m, 2H), 2.70 (t, *J* = 7.7 Hz, 2H), 2.10 – 2.03 (m, 3H), 1.99 – 1.89 (m, 3H), 1.87 (d, *J* = 3.0 Hz, 5H), 1.69 – 1.59 (m, 6H).

^{13}C NMR (101 MHz, DMSO-*d*$_6$) δ 141.86, 129.36, 129.29, 127.02, 60.16, 44.24, 36.20, 34.91, 34.65, 28.49, 23.28.

HRMS (ESI/QTOF) m/z: $[M + Na]^+$ Calcd for $C_{19}H_{26}O_2SNa^+$ 341.1546; Found 341.1549.

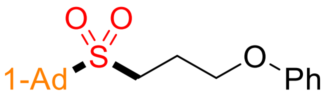

(3*S*,5*S*,7*S*)-1-((3-phenoxypropyl)sulfonyl)adamantane (3o): Following the General Procedure A, purified by preparative TLC, using petroleum ether /EA = 10/1 (v/v) as an eluent. White solid in 59% yield.

^{1}H NMR (400 MHz, $CDCl_3$) δ 7.19 (dd, *J* = 8.7, 7.3 Hz, 2H), 6.86 (t, *J* = 7.3 Hz, 1H), 6.81 (d, *J* = 8.1 Hz, 2H), 4.02 (t, *J* = 5.8 Hz, 2H), 3.03 – 2.94 (m, 2H), 2.26 (dq, *J* = 7.7, 5.8 Hz, 2H), 2.09 (d, *J* = 3.5 Hz, 3H), 1.96 (d, *J* = 3.0 Hz, 5H), 1.84 – 1.71 (m, 1H), 1.71 – 1.57 (m, 6H).

^{13}C NMR (101 MHz, $CDCl_3$) δ 158.28, 129.32, 120.82, 114.26, 65.76, 60.21, 41.43, 35.58, 34.52, 27.89, 20.67.

HRMS (ESI/QTOF) m/z: $[M + Na]^+$ Calcd for $C_{19}H_{26}O_3SNa^+$ 357.1495; Found 357.1497.

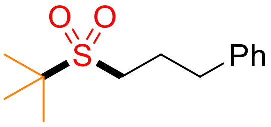

(3-(*tert*-butylsulfonyl)propyl)benzene (3p): Following the General Procedure A, purified by preparative TLC, using petroleum ether /EA = 10/1 (v/v) as an eluent. White solid in 79% yield.

^{1}H NMR (400 MHz, $CDCl_3$) δ 7.22 – 7.17 (m, 2H), 7.11 (td, *J* = 6.6, 1.6 Hz, 3H), 2.84 – 2.77 (m, 2H), 2.70 (t, *J* = 7.5 Hz, 2H), 2.13 (tt, *J* = 9.8, 6.9 Hz, 2H), 1.28 (s, 9H).

^{13}C NMR (101 MHz, $CDCl_3$) δ 139.99, 128.32, 128.20, 126.09, 58.65, 44.37, 34.24, 23.11, 22.02.

HRMS (ESI/QTOF) m/z: $[M + Na]^+$ Calcd for $C_{13}H_{20}O_2SNa^+$ 263.1076; Found 263.1087.

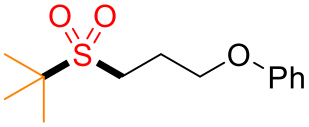

(3-(*tert*-butylsulfonyl)propoxy)benzene (3q): Following the General Procedure A, purified by preparative TLC, using petroleum ether /EA = 10/1 (v/v) as an eluent. White solid in 85% yield.

^{1}H NMR (400 MHz, $CDCl_3$) δ 7.17 (dd, *J* = 8.7, 7.2 Hz, 2H), 6.85 (t, *J* = 7.4 Hz, 1H), 6.79 (d, *J* = 8.2 Hz, 2H), 3.99 (t, *J* = 5.8 Hz, 2H), 3.07 – 2.98 (m, 2H), 2.29 – 2.19 (m, 2H), 1.31 (s, 9H).

^{13}C NMR (101 MHz, $CDCl_3$) δ 158.20, 129.25, 120.76, 114.19, 65.55, 58.71, 42.33, 23.08, 20.83.

HRMS (ESI/QTOF) m/z: $[M + Na]^+$ Calcd for $C_{13}H_{20}O_3SNa^+$ 279.1025; Found 279.1008.

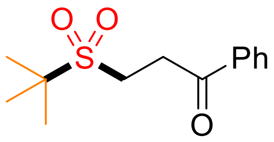

3-(*tert*-butylsulfonyl)-1-phenylpropan-1-one (3r): Following the General Procedure A, purified by preparative TLC, using petroleum ether /EA = 5/1 (v/v) as an eluent. White solid in 73% yield.

^{1}H NMR (400 MHz, $CDCl_3$) δ 7.93 – 7.89 (m, 2H), 7.54 – 7.49 (m, 1H), 7.43 – 7.37 (m, 2H), 3.54 – 3.47 (m, 2H), 3.34 – 3.28 (m, 2H), 1.39 (s, 9H).

^{13}C NMR (101 MHz, $CDCl_3$) δ 196.14, 135.77, 133.61, 128.64, 127.99, 58.97, 40.28, 29.47, 23.22.

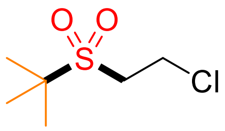

2-((2-chloroethyl)sulfonyl)-2-methylpropane (3s): Following the General Procedure A, purified by preparative TLC, using petroleum ether /EA = 10/1 (v/v) as an eluent. White solid in 77% yield.

^{1}H NMR (400 MHz, $CDCl_3$) δ 3.87 (ddd, *J* = 10.0, 6.0, 2.1 Hz, 2H), 3.31 (ddd, *J* = 10.1, 6.0, 2.1 Hz, 2H), 1.37 (d, *J* = 2.2 Hz, 9H).

^{13}C NMR (101 MHz, $CDCl_3$) δ 59.77, 48.30, 35.13, 23.21.

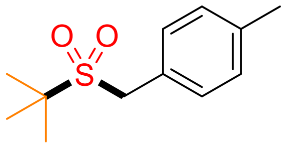

1-((*tert*-butylsulfonyl)methyl)-4-methylbenzene (3t): Following the General Procedure A, purified by preparative TLC, using petroleum ether /EA = 10/1 (v/v) as an eluent. White solid in 61% yield.

^{1}H NMR (400 MHz, $CDCl_3$) δ 7.21 (d, *J* = 8.1 Hz, 2H), 7.08 (d, *J* = 7.8 Hz, 2H), 4.05 (s, 2H), 2.25 (s, 3H), 1.32 (d, *J* = 0.9 Hz, 9H).

^{13}C NMR (101 MHz, $CDCl_3$) δ 138.34, 130.89, 129.23, 123.87, 59.59, 52.21, 23.55, 21.03.

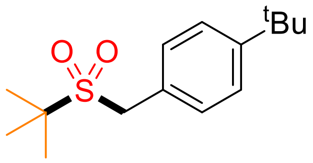

1-(*tert*-butyl)-4-((*tert*-butylsulfonyl)methyl)benzene (3u): Following the General Procedure A, purified by preparative TLC, using petroleum ether /EA = 10/1 (v/v) as an eluent. White solid in

67% yield.

^{1}H NMR (400 MHz, CDCl$_3$) δ 7.34 – 7.25 (m, 4H), 4.09 (s, 2H), 1.36 (s, 9H), 1.24 (s, 9H).

^{13}C NMR (101 MHz, CDCl$_3$) δ 151.62, 130.82, 125.63, 123.85, 59.68, 52.10, 34.52, 31.18, 23.68.

HRMS (ESI/QTOF) m/z: [M + Na]$^+$ Calcd for $C_{15}H_{24}O_2SNa^+$ 291.1389; Found 291.1394.

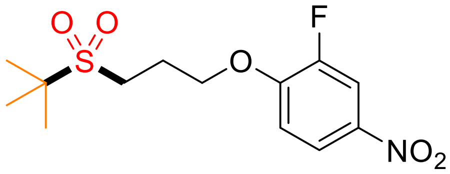

1-(3-(*tert*-butylsulfonyl)propoxy)-2-fluoro-4-nitrobenzene (3v): Following the General Procedure A, purified by preparative TLC, using petroleum ether /EA = 5/1 (v/v) as an eluent. White solid in 43% yield.

^{1}H NMR (400 MHz, CDCl$_3$) δ 8.05 (ddd, *J* = 9.1, 2.7, 1.5 Hz, 1H), 7.99 (dd, *J* = 10.6, 2.7 Hz, 1H), 7.06 (dd, *J* = 9.1, 8.0 Hz, 1H), 4.36 (t, *J* = 5.9 Hz, 2H), 3.19 (t, *J* = 7.2 Hz, 2H), 2.50 (ddd, *J* = 13.1, 7.2, 5.9 Hz, 2H), 1.45 (s, 9H).

^{13}C NMR (101 MHz, CDCl$_3$) δ 151.20 (d, *J* = 251.5 Hz), 152.25 (d, *J* = 10.7 Hz), 141.08 (d, *J* = 7.5 Hz), 120.91 (d, *J* = 3.6 Hz), 113.22 (d, *J* = 2.1 Hz), 112.36 (d, *J* = 22.9 Hz), 67.72, 59.24, 41.71, 23.35, 21.02.

^{19}F NMR (376 MHz, CDCl$_3$) δ -130.28 (dd, *J* = 10.4, 8.3 Hz).

HRMS (ESI/QTOF) m/z: [M + H]$^+$ Calcd for $C_{13}H_{18}FNO_5SH^+$ 320.0962; Found 320.0948.

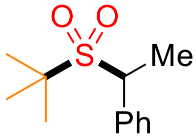

(1-(*tert*-butylsulfonyl)ethyl)benzene (3w): Following the General Procedure A, purified by preparative TLC, using petroleum ether /EA = 10/1 (v/v) as an eluent. White solid in 40% yield.

^{1}H NMR (400 MHz, CDCl$_3$) δ 7.45 – 7.40 (m, 2H), 7.33 – 7.25 (m, 3H), 4.31 (q, *J* = 7.1 Hz, 1H), 1.71 (d, *J* = 7.1 Hz, 3H), 1.13 (s, 9H).

^{13}C NMR (101 MHz, CDCl$_3$) δ 136.33, 128.85, 128.82, 128.69, 61.61, 59.85, 24.20, 16.43.

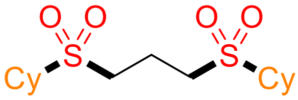

1,3-bis(cyclohexylsulfonyl)propane (3x): Following the General Procedure A, purified by

preparative TLC, using petroleum ether /EA = 2/1 (v/v) as an eluent. White solid in 42% yield.

^{1}H NMR (400 MHz, $CDCl_3$) δ 3.18 (t, *J* = 7.1 Hz, 4H), 2.87 (tt, *J* = 12.2, 3.5 Hz, 2H), 2.43 (p, *J* = 7.1 Hz, 2H), 2.21 – 2.12 (m, 4H), 1.94 (dt, *J* = 12.9, 3.2 Hz, 4H), 1.78 – 1.71 (m, 2H), 1.54 (qd, *J* = 12.4, 3.4 Hz, 4H), 1.28 (ddtd, *J* = 25.1, 15.9, 12.7, 3.3 Hz, 6H).

^{13}C NMR (101 MHz, $CDCl_3$) δ 61.44, 47.08, 24.94, 14.19.

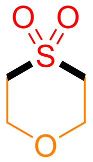

1,4-oxathiane 4,4-dioxide (3y): Following the General Procedure A, purified by preparative TLC, using petroleum ether /EA = 5/1 (v/v) as an eluent. White solid in 50% yield.

^{1}H NMR (400 MHz, $CDCl_3$) δ 4.07 (dt, *J* = 6.4, 3.1 Hz, 4H), 3.05 (q, *J* = 4.8, 4.0 Hz, 4H).

^{13}C NMR (101 MHz, $CDCl_3$) δ 66.10, 52.81.

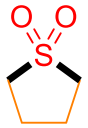

tetrahydrothiophene 1,1-dioxide (3z): Following the General Procedure A, purified by preparative TLC, using petroleum ether /EA = 10/1 (v/v) as an eluent. White solid in 32% yield.

^{1}H NMR (400 MHz, $CDCl_3$) δ 2.96 (tt, *J* = 7.3, 3.0 Hz, 4H), 2.16 (td, *J* = 7.5, 3.0 Hz, 4H).

^{13}C NMR (101 MHz, $CDCl_3$) δ 50.99, 22.59.

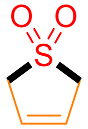

2,5-dihydrothiophene 1,1-dioxide (3aa): Following the General Procedure A, purified by preparative TLC, using petroleum ether /EA = 5/1 (v/v) as an eluent. White solid in 46% yield.

^{1}H NMR (400 MHz, $CDCl_3$) δ 6.01 (d, *J* = 1.8 Hz, 2H), 3.68 (t, *J* = 1.7 Hz, 4H).

^{13}C NMR (101 MHz, $CDCl_3$) δ 124.49, 55.55.

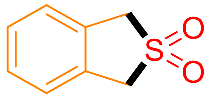

1,3-dihydrobenzo[*c*]thiophene 2,2-dioxide (3ab): Following the General Procedure A, purified by preparative TLC, using petroleum ether /EA = 10/1 (v/v) as an eluent. White solid in 54% yield.

^{1}H NMR (400 MHz, $CDCl_3$) δ 7.27 (dt, *J* = 7.3, 3.7 Hz, 2H), 7.24 – 7.20 (m, 2H), 4.27 (s, 4H).

^{13}C NMR (101 MHz, $CDCl_3$) δ 131.13, 128.67, 125.92, 56.74.

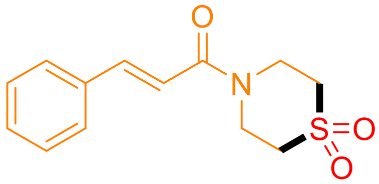

(*E*)-1-(1,1-dioxidothiomorpholino)-3-phenylprop-2-en-1-one (3ac): Following the General Procedure A, purified by preparative TLC, using petroleum ether /EA = 2/1 (v/v) as an eluent. White solid in 61% yield.

^{1}H NMR (400 MHz, $CDCl_3$) δ 7.67 (dd, *J* = 15.5, 3.2 Hz, 1H), 7.49 – 7.43 (m, 2H), 7.36 – 7.30 (m, 3H), 6.78 (dd, *J* = 15.6, 3.1 Hz, 1H), 4.10 (s, 4H), 3.03 (d, *J* = 5.8 Hz, 4H).

^{13}C NMR (101 MHz, $CDCl_3$) δ 165.86, 145.29, 134.46, 130.35, 128.95, 127.96, 115.09, 52.27, 29.66.

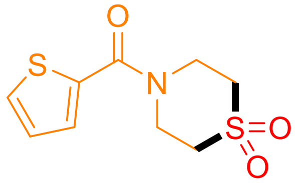

(1,1-dioxidothiomorpholino)(thiophen-2-yl)methanone (3ad): Following the General Procedure A, purified by preparative TLC, using petroleum ether /EA = 2/1 (v/v) as an eluent. White solid in 40% yield.

^{1}H NMR (400 MHz, $CDCl_3$) δ 7.66 – 7.01 (m, 3H), 4.40 – 4.01 (m, 4H), 3.26 – 2.94 (m, 4H).

^{13}C NMR (101 MHz, $CDCl_3$) δ 164.13, 134.98, 129.84, 129.72, 127.12, 51.97, 43.83.

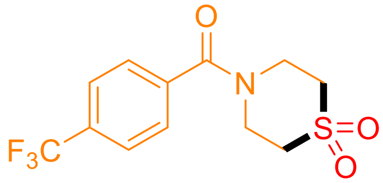

(1,1-dioxidothiomorpholino)(4-(trifluoromethyl)phenyl)methanone (3ae): Following the General Procedure A, purified by preparative TLC, using petroleum ether /EA = 2/1 (v/v) as an eluent. White solid in 57% yield.

^{1}H NMR (400 MHz, $CDCl_3$) δ 7.67 (d, *J* = 7.8 Hz, 2H), 7.49 (d, *J* = 7.9 Hz, 2H), 4.01 (d, *J* = 123.1 Hz, 4H), 3.02 (d, *J* = 58.4 Hz, 4H).

^{13}C NMR (101 MHz, $CDCl_3$) δ 169.47, 137.47, 132.62 (q, J = 33.0 Hz), 127.32, 126.03 (q, J = 3.7 Hz), 123.39(q, J = 273.6 Hz), 51.88, 29.64.

^{19}F NMR (377 MHz, $CDCl_3$) δ -63.02.

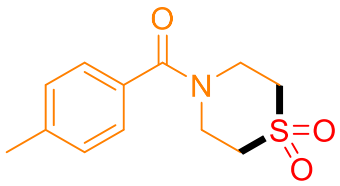

(1,1-dioxidothiomorpholino)(*p*-tolyl)methanone (3af): Following the General Procedure A, purified by preparative TLC, using petroleum ether /EA = 2/1 (v/v) as an eluent. White solid in 59% yield.

^1H NMR (400 MHz, $CDCl_3$) δ 7.29 – 7.11 (m, 4H), 3.98 (s, 4H), 2.98 (s, 4H), 2.31 (d, J = 3.0 Hz, 3H).

^{13}C NMR (101 MHz, $CDCl_3$) δ 170.97, 140.85, 130.90, 129.28, 126.92, 51.76, 21.23.

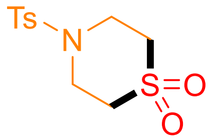

4-tosylthiomorpholine 1,1-dioxide (3ag): Following the General Procedure A, purified by preparative TLC, using petroleum ether /EA = 1/1 (v/v) as an eluent. White solid in 48% yield.

^1H NMR (400 MHz, $CDCl_3$) δ 7.61 – 7.55 (m, 2H), 7.29 (dd, J = 8.3, 2.7 Hz, 2H), 3.64 – 3.51 (m, 4H), 3.06 (d, J = 5.1 Hz, 4H), 2.38 (s, 3H).

^{13}C NMR (101 MHz, $CDCl_3$) δ 144.65, 133.63, 130.14, 127.23, 51.25, 44.77, 21.50.

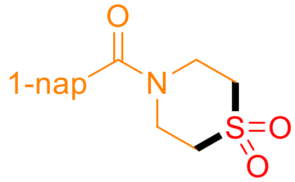

(1,1-dioxidothiomorpholino)(naphthalen-1-yl)methanone (3ah): Following the General Procedure A, purified by preparative TLC, using petroleum ether /EA = 2/1 (v/v) as an eluent. White solid in 50% yield.

^1H NMR (400 MHz, $CDCl_3$) δ 7.90 – 7.77 (m, 2H), 7.69 (dd, J = 6.2, 3.1 Hz, 1H), 7.52 – 7.31 (m, 4H), 4.32 (d, J = 5.2 Hz, 2H), 3.71 – 3.48 (m, 2H), 3.17 (q, J = 5.3, 4.8 Hz, 2H), 2.88 – 2.65 (m, 2H).

^{13}C NMR (101 MHz, $CDCl_3$) δ 169.79, 133.39, 132.15, 129.98, 129.23, 128.67, 127.56, 126.79, 125.06, 123.92, 123.84, 52.21, 51.91, 45.36, 40.11.

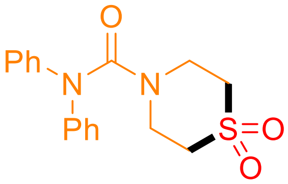

***N,N*-diphenylthiomorpholine-4-carboxamide 1,1-dioxide (3ai):** Following the General Procedure A, purified by preparative TLC, using petroleum ether /EA = 2/1 (v/v) as an eluent. White solid in 55% yield.

^{1}H NMR (400 MHz, $CDCl_3$) δ 7.26 (t, J = 7.7 Hz, 4H), 7.11 (t, J = 7.4 Hz, 2H), 6.98 (d, J = 7.9 Hz, 4H), 3.74 (t, J = 5.3 Hz, 4H), 2.73 (t, J = 5.2 Hz, 4H).

^{13}C NMR (101 MHz, $CDCl_3$) δ 159.41, 144.10, 129.43, 125.63, 125.31, 51.04, 44.10.

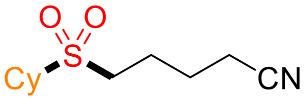

5-(cyclohexylsulfonyl)pentanenitrile (4a): Following the General Procedure B, purified by preparative TLC, using petroleum ether /EA = 5/1 (v/v) as an eluent. White solid in 62% yield.

^{1}H NMR (400 MHz, $CDCl_3$) δ 2.96 (t, J = 7.2 Hz, 2H), 2.85 (td, J = 12.2, 3.2 Hz, 1H), 2.44 (t, J = 6.7 Hz, 2H), 2.16 (d, J = 12.9 Hz, 2H), 2.09 – 1.82 (m, 6H), 1.79 – 1.68 (m, 1H), 1.54 (q, J = 12.6, 11.5 Hz, 2H), 1.39 – 1.18 (m, 3H).

^{13}C NMR (101 MHz, $CDCl_3$) δ 118.88, 61.27, 47.98, 25.01, 24.97, 24.95, 20.56, 16.84.

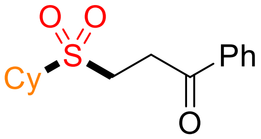

3-(cyclohexylsulfonyl)-1-phenylpropan-1-one (4b): Following the General Procedure A, purified by preparative TLC, using petroleum ether /EA = 8/1 (v/v) as an eluent. White solid in 56% yield.

^{1}H NMR (400 MHz, $CDCl_3$) δ 7.97 – 7.86 (m, 2H), 7.56 – 7.49 (m, 1H), 7.41 (dd, J = 8.4, 7.1 Hz, 2H), 3.49 (dd, J = 8.4, 6.6 Hz, 2H), 3.30 (dd, J = 8.3, 6.6 Hz, 2H), 2.82 (tt, J = 12.2, 3.5 Hz, 1H), 2.22 – 2.11 (m, 2H), 1.87 (dt, J = 12.2, 2.8 Hz, 2H), 1.73 – 1.62 (m, 1H), 1.50 (qd, J = 12.4, 3.5 Hz, 2H), 1.31 – 1.09 (m, 3H).

^{13}C NMR (101 MHz, $CDCl_3$) δ 195.97, 135.78, 133.70, 128.71, 128.05, 61.75, 43.80, 30.00, 25.06,

24.99, 24.97.

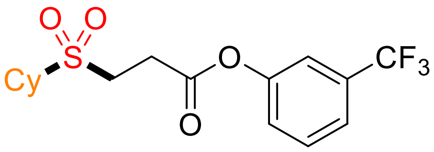

3-(trifluoromethyl)phenyl 3-(cyclohexylsulfonyl)propanoate (4c): Following the General Procedure A, purified by preparative TLC, using petroleum ether /EA = 5/1 (v/v) as an eluent. White solid in 61% yield.

^{1}H NMR (400 MHz, $CDCl_3$) δ 7.44 (d, *J* = 4.7 Hz, 2H), 7.32 (s, 1H), 7.25 (td, *J* = 5.2, 4.7, 2.6 Hz, 1H), 3.28 (t, *J* = 7.3 Hz, 2H), 3.08 (t, *J* = 7.3 Hz, 2H), 2.92 – 2.77 (m, 1H), 2.14 (dd, *J* = 13.3, 3.5 Hz, 2H), 1.92 – 1.83 (m, 2H), 1.70 – 1.63 (m, 1H), 1.50 (qd, *J* = 12.4, 3.5 Hz, 2H), 1.31 – 1.16 (m, 3H).

^{13}C NMR (101 MHz, $CDCl_3$) δ 169.05, 150.36, 131.93(q, *J* = 33.1 Hz), 130.09, 127.43, 123.36(d, *J* = 272.5 Hz), 122.94 (q, *J* = 3.8 Hz), 118.66 (q, *J* = 3.7 Hz), 61.74, 44.35, 26.33, 25.02, 24.95, 24.93.

^{19}F NMR (377 MHz, $CDCl_3$) δ -62.67.

HRMS (ESI/QTOF) m/z: $[M + Na]^+$ Calcd for $C_{16}H_{19}F_3O_4SNa^+$ 387.0848; Found 387.0850.

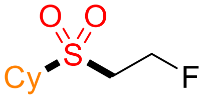

((2-fluoroethyl)sulfonyl)cyclohexane (4d): Following the General Procedure A, purified by preparative TLC, using petroleum ether /EA = 10/1 (v/v) as an eluent. Pale yellow liquid in 45% yield.

^{1}H NMR (400 MHz, $CDCl_3$) δ 4.93 (d, *J* = 5.0 Hz, 1H), 4.82 (d, *J* = 5.0 Hz, 1H), 3.34 (dt, *J* = 25.4, 5.8 Hz, 2H), 2.94 (td, *J* = 12.2, 3.6 Hz, 1H), 2.16 (d, *J* = 12.7 Hz, 2H), 1.93 (d, *J* = 10.3 Hz, 2H), 1.79 – 1.66 (m, 1H), 1.54 (q, *J* = 12.5 Hz, 2H), 1.29 (dtt, *J* = 24.9, 15.9, 8.0 Hz, 3H).

^{13}C NMR (101 MHz, $CDCl_3$) δ 77.29 (d, *J* = 170.1 Hz), 61.85, 50.12 (d, *J* = 21.5 Hz), 24.74, 24.65, 24.37.

^{19}F NMR (376 MHz, $CDCl_3$) δ -220.38 (tt, *J* = 46.6, 25.3 Hz).

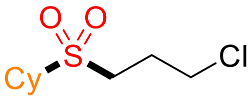

((3-chloropropyl)sulfonyl)cyclohexane (4e): Following the General Procedure B, purified by

preparative TLC, using petroleum ether /EA = 10/1 (v/v) as an eluent. White solid in 49% yield.

^{1}H NMR (400 MHz, $CDCl_3$) δ 3.72 (t, *J* = 6.2 Hz, 2H), 3.14 – 3.06 (m, 2H), 2.88 (tt, *J* = 12.2, 3.5 Hz, 1H), 2.37 – 2.27 (m, 2H), 2.21 – 2.12 (m, 2H), 1.94 (dt, *J* = 12.9, 3.2 Hz, 2H), 1.78 – 1.69 (m, 1H), 1.54 (qd, *J* = 12.4, 3.4 Hz, 2H), 1.29 (ddtd, *J* = 25.9, 15.9, 12.7, 3.3 Hz, 3H).

^{13}C NMR (101 MHz, $CDCl_3$) δ 61.04, 46.14, 42.99, 24.72, 24.21.

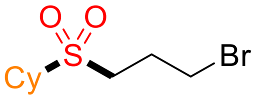

((3-bromopropyl)sulfonyl)cyclohexane (4f): Following the General Procedure A, purified by preparative TLC, using petroleum ether /EA = 10/1 (v/v) as an eluent. White solid in 44% yield.

^{1}H NMR (400 MHz, $CDCl_3$) δ 3.57 (t, *J* = 6.2 Hz, 2H), 3.12 – 3.05 (m, 2H), 2.86 (tt, *J* = 12.2, 3.5 Hz, 1H), 2.46 – 2.37 (m, 2H), 2.22 – 2.14 (m, 2H), 1.99 – 1.90 (m, 2H), 1.78 – 1.70 (m, 1H), 1.56 (qd, *J* = 12.4, 4.0 Hz, 2H), 1.39 – 1.20 (m, 3H).

^{13}C NMR (101 MHz, $CDCl_3$) δ 61.45, 47.53, 31.48, 25.00, 24.98, 24.96, 24.45.

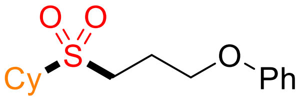

(3-(cyclohexylsulfonyl)propoxy)benzene (4g): Following the General Procedure B, purified by preparative TLC, using petroleum ether /EA = 10/1 (v/v) as an eluent. White solid in 63% yield.

^{1}H NMR (400 MHz, $CDCl_3$) δ 7.19 (t, *J* = 7.9 Hz, 2H), 6.93 – 6.73 (m, 3H), 3.98 (t, *J* = 5.8 Hz, 2H), 3.11 – 2.97 (m, 2H), 2.76 (tt, *J* = 12.2, 3.4 Hz, 1H), 2.27 – 2.16 (m, 2H), 2.08 (dd, *J* = 12.8, 3.6 Hz, 2H), 1.83 (dt, *J* = 12.6, 3.3 Hz, 2H), 1.67 – 1.58 (m, 1H), 1.45 (qd, *J* = 12.4, 3.6 Hz, 2H), 1.17 (tq, *J* = 16.4, 12.5, 10.8 Hz, 3H).

^{13}C NMR (101 MHz, $CDCl_3$) δ 158.15, 129.30, 120.85, 114.19, 65.39, 60.87, 46.01, 24.80, 24.80, 21.38.

HRMS (ESI/QTOF) m/z: $[M + Na]^+$ Calcd for $C_{16}H_{22}O_3SNa^+$ 305.1182; Found 305.1192.

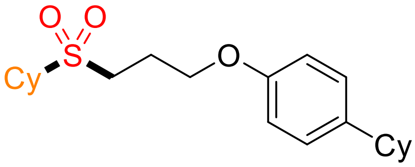

1-cyclohexyl-4-(3-(cyclohexylsulfonyl)propoxy)benzene (4h): Following the General Procedure B, purified by preparative TLC, using petroleum ether /EA = 10/1 (v/v) as an eluent. White solid in

52% yield.

^{1}H NMR (400 MHz, $CDCl_3$) δ 7.11 (d, *J* = 8.6 Hz, 2H), 6.81 (d, *J* = 8.6 Hz, 2H), 4.06 (t, *J* = 5.8 Hz, 2H), 3.17 – 3.09 (m, 2H), 2.86 (tt, *J* = 12.2, 3.4 Hz, 1H), 2.43 (p, *J* = 7.1, 5.9 Hz, 1H), 2.34 – 2.25 (m, 2H), 2.17 (d, *J* = 13.0 Hz, 2H), 1.97 – 1.68 (m, 8H), 1.54 (qd, *J* = 12.3, 4.3 Hz, 2H), 1.41 – 1.17 (m, 8H).

^{13}C NMR (101 MHz, $CDCl_3$) δ 156.32, 140.74, 127.57, 114.10, 65.62, 61.00, 46.18, 43.52, 34.54, 26.77, 25.99, 24.92, 24.86, 21.53.

HRMS (ESI/QTOF) m/z: $[M + H]^+$ Calcd for $C_{21}H_{32}O_3SH^+$ 365.2145; Found 365.2144.

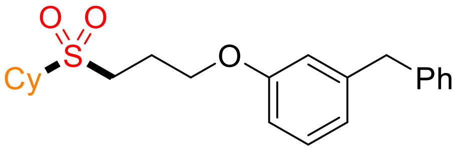

1-benzyl-3-(3-(cyclohexylsulfonyl)propoxy)benzene (4i): Following the General Procedure B, purified by preparative TLC, using petroleum ether /EA = 10/1 (v/v) as an eluent. White solid in 70% yield.

^{1}H NMR (400 MHz, $CDCl_3$) δ 7.27 – 7.12 (m, 7H), 6.92 (td, *J* = 7.5, 1.0 Hz, 1H), 6.83 (d, *J* = 8.3 Hz, 1H), 4.04 (t, *J* = 5.6 Hz, 2H), 3.97 (s, 2H), 2.83 – 2.68 (m, 3H), 2.25 (dq, *J* = 7.7, 5.6 Hz, 2H), 2.09 – 2.01 (m, 2H), 1.96 – 1.88 (m, 2H), 1.76 – 1.68 (m, 1H), 1.46 (qd, *J* = 12.4, 3.6 Hz, 2H), 1.32 – 1.20 (m, 3H).

^{13}C NMR (101 MHz, $CDCl_3$) δ 156.15, 141.02, 130.92, 128.91, 128.41, 128.18, 127.69, 125.68, 120.84, 111.00, 65.58, 61.08, 46.02, 36.42, 24.98, 24.96, 24.86, 21.30.

HRMS (ESI/QTOF) m/z: $[M + Na]^+$ Calcd for $C_{22}H_{28}O_3SNa^+$ 395.1651; Found 395.1655.

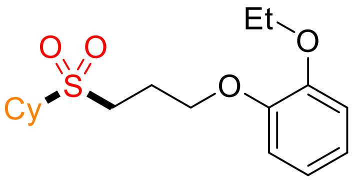

1-(3-(cyclohexylsulfonyl)propoxy)-2-ethoxybenzene (4j): Following the General Procedure B, purified by preparative TLC, using petroleum ether /EA = 10/1 (v/v) as an eluent. White solid in 51% yield.

^{1}H NMR (400 MHz, $CDCl_3$) δ 6.97 – 6.84 (m, 4H), 4.14 (t, *J* = 5.8 Hz, 2H), 4.06 (q, *J* = 7.0 Hz, 2H), 3.26 – 3.17 (m, 2H), 2.86 (tt, *J* = 12.2, 3.5 Hz, 1H), 2.38 – 2.26 (m, 2H), 2.24 – 2.13 (m, 2H),

1.99 – 1.88 (m, 2H), 1.77 – 1.68 (m, 1H), 1.56 (qd, J = 12.3, 3.5 Hz, 2H), 1.43 (t, J = 7.0 Hz, 3H), 1.35 – 1.18 (m, 3H).

^{13}C NMR (101 MHz, $CDCl_3$) δ 149.15, 148.04, 122.03, 120.93, 114.99, 113.52, 67.47, 64.24, 60.94, 46.34, 25.02, 24.94, 21.96, 14.87.

HRMS (ESI/QTOF) m/z: $[M + Na]^+$ Calcd for $C_{17}H_{26}O_4SNa^+$ 349.1444; Found 349.1448.

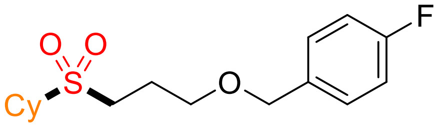

1-((3-(cyclohexylsulfonyl)propoxy)methyl)-4-fluorobenzene (4k): Following the General Procedure B, purified by preparative TLC, using petroleum ether /EA = 10/1 (v/v) as an eluent. White solid in 60% yield.

^{1}H NMR (400 MHz, $CDCl_3$) δ 7.32 – 7.25 (m, 2H), 7.03 (t, J = 8.7 Hz, 2H), 4.47 (s, 2H), 3.60 (t, J = 5.9 Hz, 2H), 3.06 – 2.99 (m, 2H), 2.83 (tt, J = 12.2, 3.5 Hz, 1H), 2.19 – 2.09 (m, 4H), 1.97 – 1.88 (m, 2H), 1.76 – 1.70 (m, 1H), 1.54 (qd, J = 12.3, 3.1 Hz, 2H), 1.27 (dtt, J = 23.8, 12.4, 6.6 Hz, 3H).

^{13}C NMR (101 MHz, $CDCl_3$) δ 162.34 (d, J = 245.9 Hz), 133.71 (d, J = 3.2 Hz), 129.39 (d, J = 8.1 Hz), 115.27 (d, J = 21.4 Hz), 72.25, 68.04, 61.05, 46.46, 25.05, 25.01, 21.92.

^{19}F NMR (376 MHz, $CDCl_3$) δ -114.61 (ddd, J = 14.1, 8.9, 5.5 Hz).

HRMS (ESI/QTOF) m/z: $[M + Na]^+$ Calcd for $C_{16}H_{23}FO_3SNa^+$ 337.1244; Found 337.1260.

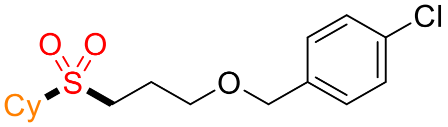

1-chloro-4-((3-(cyclohexylsulfonyl)propoxy)methyl)benzene (4l): Following the General Procedure B, purified by preparative TLC, using petroleum ether /EA = 10/1 (v/v) as an eluent. White solid in 70% yield.

^{1}H NMR (400 MHz, $CDCl_3$) δ 7.32 (d, J = 8.4 Hz, 2H), 7.25 (d, J = 8.4 Hz, 2H), 4.47 (s, 2H), 3.60 (t, J = 5.9 Hz, 2H), 3.03 (dd, J = 8.8, 6.8 Hz, 2H), 2.83 (tt, J = 12.2, 3.4 Hz, 1H), 2.20 – 2.10 (m, 4H), 1.93 (d, J = 12.9 Hz, 2H), 1.73 (d, J = 10.5 Hz, 1H), 1.59 – 1.47 (m, 2H), 1.27 (tt, J = 21.4, 11.0 Hz, 3H).

^{13}C NMR (101 MHz, $CDCl_3$) δ 136.43, 133.40, 128.88, 128.51, 72.10, 68.10, 60.98, 46.36, 24.99,

24.95, 21.85.

HRMS (ESI/QTOF) m/z: $[M + Na]^+$ Calcd for $C_{16}H_{23}ClO_3SNa^+$ 353.0949; Found 353.0959.

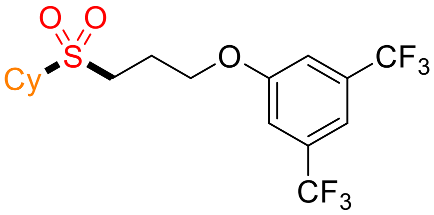

1-(3-(cyclohexylsulfonyl)propoxy)-3,5-bis(trifluoromethyl)benzene (4m): Following the General Procedure B, purified by preparative TLC, using petroleum ether /EA = 10/1 (v/v) as an eluent). White solid in 70% yield.

^1H NMR (400 MHz, $CDCl_3$) δ 7.47 (s, 1H), 7.32 (d, *J* = 1.5 Hz, 2H), 4.23 (t, *J* = 5.9 Hz, 2H), 3.19 – 3.10 (m, 2H), 2.89 (tt, *J* = 12.2, 3.6 Hz, 1H), 2.46 – 2.36 (m, 2H), 2.25 – 2.14 (m, 2H), 2.00 – 1.90 (m, 2H), 1.79 – 1.70 (m, 1H), 1.57 (qd, *J* = 12.5, 3.5 Hz, 2H), 1.39 – 1.20 (m, 3H).

^{13}C NMR (101 MHz, $CDCl_3$) δ 158.94, 132.80 (q, *J* = 33.4 Hz), 123.01 (q, *J* = 272.8 Hz), 114.73 (d, *J* = 4.0 Hz), 114.55 (p, *J* = 3.9 Hz), 66.60, 61.47, 45.65, 25.04, 24.96, 24.94, 21.23.

^{19}F NMR (376 MHz, $CDCl_3$) δ -63.08.

HRMS (ESI/QTOF) m/z: $[M + Na]^+$ Calcd for $C_{17}H_{20}F_6O_3SNa^+$ 441.0930; Found 441.0935.

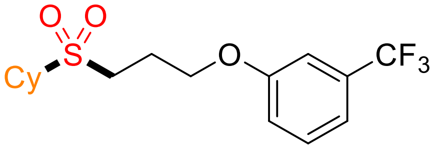

1-(3-(cyclohexylsulfonyl)propoxy)-3-(trifluoromethyl)benzene (4n): Following the General Procedure B, purified by preparative TLC, using petroleum ether /EA = 10/1 (v/v) as an eluent. White solid in 61% yield.

^1H NMR (400 MHz, $CDCl_3$) δ 7.31 (t, *J* = 8.0 Hz, 1H), 7.14 (d, *J* = 7.7 Hz, 1H), 7.04 (s, 1H), 6.98 (dd, *J* = 8.3, 2.6 Hz, 1H), 4.07 (t, *J* = 5.8 Hz, 2H), 3.11 – 3.01 (m, 2H), 2.80 (tt, *J* = 12.2, 3.5 Hz, 1H), 2.33 – 2.23 (m, 2H), 2.16 – 2.06 (m, 2H), 1.86 (dt, *J* = 12.8, 3.3 Hz, 2H), 1.71 – 1.60 (m, 1H), 1.48 (qd, *J* = 12.4, 3.6 Hz, 2H), 1.29 – 1.15 (m, 3H).

^{13}C NMR (101 MHz, $CDCl_3$) δ 158.44, 131.78 (q, *J* = 32.3 Hz), 130.03, 123.80 (q, *J* = 273.7 Hz), 117.70 (d, *J* = 3.5 Hz), 111.26 (q, *J* = 3.9 Hz)., 65.99, 61.30, 45.92, 25.00, 24.97, 24.95, 21.37.

^{19}F NMR (377 MHz, $CDCl_3$) δ -62.69.

HRMS (ESI/QTOF) m/z: $[M + H]^+$ Calcd for $C_{16}H_{21}F_3O_3SH^+$ 351.1236; Found 351.1235.

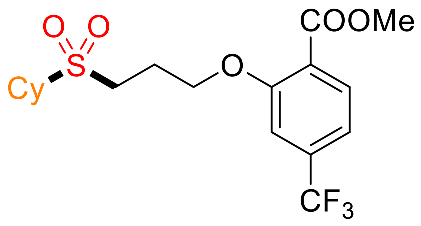

methyl 2-(3-(cyclohexylsulfonyl)propoxy)-4-(trifluoromethyl)benzoate (4o): Following the General Procedure B, purified by preparative TLC, using petroleum ether /EA = 5/1 (v/v) as an eluent. White solid in 64% yield.

^{1}H NMR (400 MHz, $CDCl_3$) δ 7.89 (d, *J* = 8.0 Hz, 1H), 7.28 – 7.24 (m, 1H), 7.16 (s, 1H), 4.25 (t, *J* = 5.7 Hz, 2H), 3.91 (s, 3H), 3.30 – 3.23 (m, 2H), 2.88 (tt, *J* = 12.2, 3.4 Hz, 1H), 2.45 – 2.36 (m, 2H), 2.28 – 2.17 (m, 2H), 1.95 (d, *J* = 13.1 Hz, 2H), 1.74 (d, *J* = 10.8 Hz, 1H), 1.58 (dd, *J* = 12.5, 3.2 Hz, 2H), 1.34 – 1.24 (m, 3H).

^{13}C NMR (101 MHz, $CDCl_3$) δ 165.29, 157.93, 135.02(d, *J* = 32.7 Hz), 132.18, 123.52, 123.27 (d, *J* = 273.0 Hz), 117.35 (d, *J* = 3.8 Hz), 110.03 (d, *J* = 3.8 Hz), 66.99, 61.43, 52.26, 45.76, 25.10, 25.04, 21.65.

^{19}F NMR (376 MHz, $CDCl_3$) δ -63.22.

HRMS (ESI/QTOF) m/z: $[M + Na]^+$ Calcd for $C_{18}H_{23}F_3O_5SNa^+$ 431.1111; Found 431.1114.

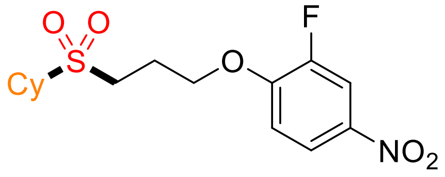

1-(3-(cyclohexylsulfonyl)propoxy)-2-fluoro-4-nitrobenzene (4p): Following the General Procedure B, purified by preparative TLC, using petroleum ether /EA = 5/1 (v/v) as an eluent. White solid in 41% yield.

^{1}H NMR (400 MHz, $CDCl_3$) δ 8.05 (ddd, *J* = 9.1, 2.7, 1.5 Hz, 1H), 7.97 (dd, *J* = 10.6, 2.7 Hz, 1H), 7.07 (dd, *J* = 9.1, 8.0 Hz, 1H), 4.34 (t, *J* = 5.9 Hz, 2H), 3.18 (t, *J* = 7.4 Hz, 2H), 2.91 (tt, *J* = 12.2, 3.5 Hz, 1H), 2.45 (tt, *J* = 7.2, 5.9 Hz, 2H), 2.20 (ddd, *J* = 13.3, 3.7, 1.8 Hz, 2H), 1.99 – 1.92 (m, 2H), 1.78 – 1.72 (m, 1H), 1.56 (qd, *J* = 12.4, 3.5 Hz, 2H), 1.39 – 1.23 (m, 3H).

^{13}C NMR (101 MHz, $CDCl_3$) δ 152.14 (d, *J* = 10.6 Hz), 151.04 (d, *J* = 251.6 Hz) 140.94 (d, *J* = 7.4 Hz), 120.83 (d, *J* = 3.5 Hz), 113.13 (d, *J* = 2.0 Hz), 112.20 (d, *J* = 22.8 Hz), 67.52, 61.43, 45.34,

24.95, 24.91, 24.90, 21.24.

^{19}F NMR (376 MHz, $CDCl_3$) δ -130.19 – -130.29 (m).

HRMS (ESI/QTOF) m/z: $[M + H]^+$ Calcd for $C_{15}H_{20}FO_5SH^+$ 346.1119; Found 346.1133.

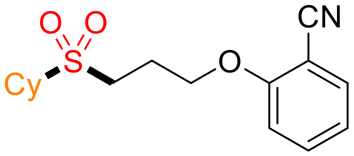

2-(3-(cyclohexylsulfonyl)propoxy)benzonitrile (4q): Following the General Procedure B, purified by preparative TLC, using petroleum ether /EA = 5/1 (v/v) as an eluent. White solid in 51% yield.

^{1}H NMR (400 MHz, $CDCl_3$) δ 7.63 – 7.47 (m, 2H), 7.09 – 6.95 (m, 2H), 4.27 (t, *J* = 5.8 Hz, 2H), 3.28 – 3.15 (m, 2H), 2.89 (tt, *J* = 12.2, 3.5 Hz, 1H), 2.42 (tt, *J* = 7.2, 5.8 Hz, 2H), 2.26 – 2.16 (m, 2H), 1.94 (ddd, *J* = 10.4, 5.3, 2.5 Hz, 2H), 1.78 – 1.69 (m, 1H), 1.56 (qd, *J* = 12.4, 3.5 Hz, 2H), 1.38 – 1.19 (m, 3H).

^{13}C NMR (101 MHz, $CDCl_3$) δ 159.91, 134.40, 133.60, 121.22, 116.17, 112.33, 102.00, 66.70, 61.47, 45.38, 24.95, 24.92, 21.35.

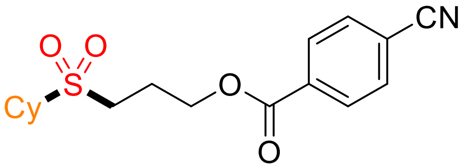

3-(cyclohexylsulfonyl)propyl 4-cyanobenzoate (4r): Following the General Procedure B, purified by preparative TLC, using petroleum ether /EA = 5/1 (v/v) as an eluent. White solid in 58% yield.

^{1}H NMR (400 MHz, $CDCl_3$) δ 8.14 (d, *J* = 8.3 Hz, 2H), 7.77 (d, *J* = 8.4 Hz, 2H), 4.52 (t, *J* = 6.2 Hz, 2H), 3.11 – 3.04 (m, 2H), 2.88 (tt, *J* = 12.1, 3.5 Hz, 1H), 2.41 – 2.32 (m, 2H), 2.18 (dd, *J* = 12.7, 2.8 Hz, 2H), 1.99 – 1.92 (m, 2H), 1.78 – 1.72 (m, 1H), 1.56 (td, *J* = 12.6, 9.2 Hz, 2H), 1.37 – 1.23 (m, 3H).

^{13}C NMR (101 MHz, $CDCl_3$) δ 164.61, 133.45, 132.24, 130.05, 117.79, 116.56, 63.67, 61.30, 45.96, 24.99, 24.94, 24.90, 20.91.

HRMS (ESI/QTOF) m/z: $[M + H]^+$ Calcd for $C_{17}H_{21}NO_4SH^+$ 336.1264; Found 336.1279.

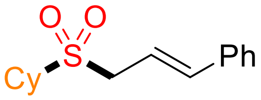

(*E*)-(3-(cyclohexylsulfonyl)prop-1-en-1-yl)benzene (4s): Following the General Procedure A,

purified by preparative TLC, using petroleum ether /EA = 8/1 (v/v) as an eluent. White solid in 45% yield.

^{1}H NMR (400 MHz, CDCl$_3$) δ 7.34 (dd, *J* = 6.1, 2.5 Hz, 2H), 7.30 – 7.18 (m, 3H), 6.61 (dd, *J* = 15.9, 3.3 Hz, 1H), 6.17 (dddd, *J* = 15.8, 7.5, 4.5, 2.2 Hz, 1H), 3.77 (dd, *J* = 7.9, 3.1 Hz, 2H), 2.93 – 2.81 (m, 1H), 2.09 (d, *J* = 12.2 Hz, 2H), 1.85 (dt, *J* = 10.4, 3.2 Hz, 2H), 1.67 – 1.45 (m, 3H), 1.27 – 1.12 (m, 3H).

^{13}C NMR (101 MHz, CDCl$_3$) δ 138.41, 135.66, 128.68, 128.55, 126.65, 115.42, 59.47, 54.24, 25.01, 24.95, 24.83.

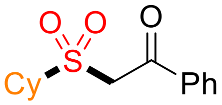

2-(cyclohexylsulfonyl)-1-phenylethan-1-one (4t): Following the General Procedure A, purified by preparative TLC, using petroleum ether /EA = 8/1 (v/v) as an eluent. White solid in 37% yield.

^{1}H NMR (400 MHz, CDCl$_3$) δ 7.97 – 7.90 (m, 2H), 7.59 – 7.51 (m, 1H), 7.42 (t, *J* = 7.8 Hz, 2H), 4.49 (s, 2H), 3.21 (tt, *J* = 12.1, 3.5 Hz, 1H), 2.19 – 2.07 (m, 2H), 1.85 (dt, *J* = 12.6, 3.1 Hz, 2H), 1.71 – 1.60 (m, 1H), 1.51 (qd, *J* = 12.4, 3.6 Hz, 2H), 1.33 – 1.07 (m, 3H).

^{13}C NMR (101 MHz, CDCl$_3$) δ 189.28, 135.79, 134.36, 129.22, 128.78, 61.22, 56.67, 24.90, 24.80, 24.63.

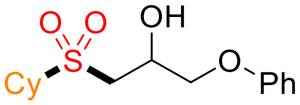

1-(cyclohexylsulfonyl)-3-phenoxypropan-2-ol (4u): Following the General Procedure B, purified by preparative TLC, using petroleum ether /EA = 2/1 (v/v) as an eluent. White solid in 56% yield.

^{1}H NMR (400 MHz, CDCl$_3$) δ 7.33 – 7.23 (m, 2H), 6.98 (t, *J* = 7.4 Hz, 1H), 6.87 (dd, *J* = 27.3, 8.4 Hz, 2H), 4.66 (tt, *J* = 5.5, 2.8 Hz, 1H), 4.05 (qdd, *J* = 9.3, 5.2, 1.6 Hz, 2H), 3.36 – 3.17 (m, 3H), 3.04 (tt, *J* = 12.2, 3.5 Hz, 1H), 2.21 (dd, *J* = 9.2, 5.1 Hz, 2H), 1.93 (dd, *J* = 7.6, 3.9 Hz, 2H), 1.74 (d, *J* = 10.8 Hz, 1H), 1.63 – 1.49 (m, 2H), 1.36 – 1.18 (m, 3H).

^{13}C NMR (101 MHz, CDCl$_3$) δ 157.97, 129.56, 121.50, 114.51, 70.18, 65.00, 62.23, 52.64, 25.26, 24.97, 24.92, 24.34.

HRMS (ESI/QTOF) m/z: $[M + Na]^+$ Calcd for $C_{15}H_{22}O_4SNa^+$ 321.1131; Found 321.1136.

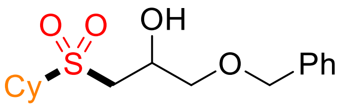

1-(benzyloxy)-3-(cyclohexylsulfonyl)propan-2-ol (4v): Following the General Procedure B, purified by preparative TLC, using petroleum ether /EA = 2/1 (v/v) as an eluent. White solid in 62% yield.

^{1}H NMR (400 MHz, $CDCl_3$) δ 7.38 – 7.27 (m, 5H), 4.61 – 4.51 (m, 2H), 4.44 (dtd, J = 8.8, 5.2, 2.5 Hz, 1H), 3.57 – 3.49 (m, 2H), 3.20 (dd, J = 14.4, 9.1 Hz, 2H), 3.07 – 2.98 (m, 2H), 2.17 (t, J = 13.0 Hz, 2H), 1.91 (dq, J = 8.7, 3.2 Hz, 2H), 1.74 – 1.67 (m, 1H), 1.53 (qt, J = 12.6, 3.6 Hz, 2H), 1.35 – 1.17 (m, 3H).

^{13}C NMR (101 MHz, $CDCl_3$) δ 137.38, 128.41, 127.86, 127.70, 73.36, 72.50, 65.31, 61.90, 52.73, 25.32, 24.91, 24.84, 23.99.

HRMS (ESI/QTOF) m/z: [M + Na]$^+$ Calcd for $C_{16}H_{24}O_4SNa^+$ 335.1288; Found 335.1295.

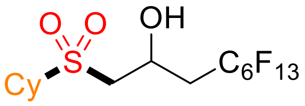

1-(cyclohexylsulfonyl)-4,4,5,5,6,6,7,7,8,8,9,9,9-tridecafluorononan-2-ol (4w): Following the General Procedure B, purified by preparative TLC, using petroleum ether /EA = 2/1 (v/v) as an eluent. White solid in 40% yield.

^{1}H NMR (400 MHz, $CDCl_3$) δ 4.82 (d, J = 7.2 Hz, 1H), 3.58 – 3.39 (m, 1H), 3.27 – 3.09 (m, 2H), 2.99 (tt, J = 12.2, 3.5 Hz, 1H), 2.62 – 2.27 (m, 2H), 2.19 (dt, J = 11.9, 1.8 Hz, 2H), 2.04 – 1.88 (m, 2H), 1.75 (ddd, J = 10.8, 3.1, 1.6 Hz, 1H), 1.62 – 1.48 (m, 2H), 1.39 – 1.19 (m, 3H).

^{13}C NMR (101 MHz, $CDCl_3$) δ 62.65, 60.71 (t, J = 3.3 Hz), 60.68, 55.01, 37.25 (t, J = 20.6 Hz), 24.95, 24.93, 24.83 (d, J = 41.6 Hz).

^{19}F NMR (376 MHz, $CDCl_3$) δ -80.81 (dt, J = 12.6, 5.7 Hz), -112.60 (tdd, J = 22.7, 12.3, 4.2 Hz), -121.81 (q, J = 13.2, 11.2 Hz), -122.76 – -123.01 (m), -123.61 (td, J = 14.9, 6.8 Hz), -125.95 – -126.30 (m).

HRMS (ESI/QTOF) m/z: [M + H]$^+$ Calcd for $C_{15}H_{17}F_{13}O_3SH^+$ 525.0764; Found 525.0782.

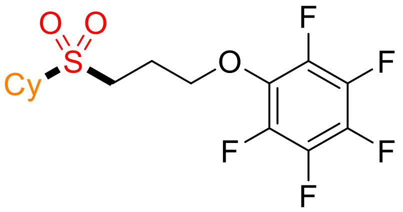

1-(3-(cyclohexylsulfonyl)propoxy)-2,3,4,5,6-pentafluorobenzene (4x): Following the General Procedure B, purified by preparative TLC, using petroleum ether /EA = 10/1 (v/v) as an eluent. White solid in 55% yield.

^{1}H NMR (400 MHz, $CDCl_3$) δ 4.31 (t, *J* = 5.8 Hz, 2H), 3.22 – 3.13 (m, 2H), 2.88 (tt, *J* = 12.2, 3.5 Hz, 1H), 2.39 – 2.29 (m, 2H), 2.26 – 2.15 (m, 2H), 1.96 (ddd, *J* = 10.4, 5.3, 2.6 Hz, 2H), 1.80 – 1.70 (m, 1H), 1.57 (qd, *J* = 12.4, 3.2 Hz, 2H), 1.30 (dddd, *J* = 23.9, 15.6, 12.6, 9.4 Hz, 3H).

^{13}C NMR (101 MHz, $CDCl_3$) δ 143.03 (m), 140.52 (m), 139.07 (m), 136.73 (m), 133.12 (m), 73.51, 61.51, 45.36, 25.07, 25.05, 25.01, 22.11.

^{19}F NMR (376 MHz, $CDCl_3$) δ -156.78 (dd, *J* = 19.7, 2.4 Hz), -162.45 – -162.67 (m), -162.74 – -162.98 (m).

HRMS (ESI/QTOF) m/z: $[M + H]^+$ Calcd for $C_{15}H_{17}F_5O_3SH^+$ 373.0891; Found 373.0892.

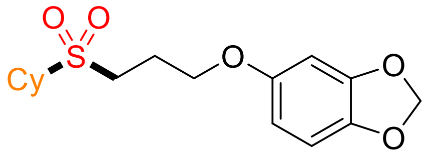

5-(3-(cyclohexylsulfonyl)propoxy)benzo[*d*][1,3]dioxole (4y): Following the General Procedure B, purified by preparative TLC, using petroleum ether /EA = 10/1 (v/v) as an eluent. White solid in 66% yield.

^{1}H NMR (400 MHz, $CDCl_3$) δ 6.70 (dd, *J* = 8.5, 1.3 Hz, 1H), 6.47 (d, *J* = 2.2 Hz, 1H), 6.30 (dt, *J* = 8.5, 2.0 Hz, 1H), 5.91 (d, *J* = 1.4 Hz, 2H), 4.02 (td, *J* = 5.7, 1.3 Hz, 2H), 3.16 – 3.08 (m, 2H), 2.86 (ttd, *J* = 12.4, 3.5, 1.3 Hz, 1H), 2.29 (dddd, *J* = 11.5, 7.4, 5.8, 1.3 Hz, 2H), 2.23 – 2.13 (m, 2H), 1.98 – 1.89 (m, 2H), 1.78 – 1.69 (m, 1H), 1.56 (qd, *J* = 12.3, 3.5 Hz, 2H), 1.37 – 1.20 (m, 3H).

^{13}C NMR (101 MHz, $CDCl_3$) δ 153.79, 148.25, 141.88, 107.89, 105.57, 101.13, 98.01, 66.66, 61.17, 46.17, 24.99, 21.55.

HRMS (ESI/QTOF) m/z: $[M + H]^+$ Calcd for $C_{16}H_{22}O_5SH^+$ 327.1261; Found 327.1274.

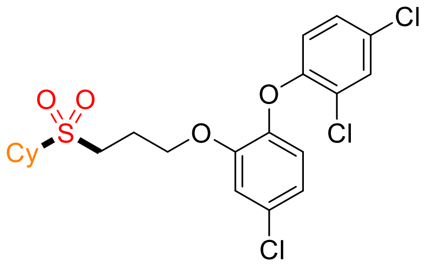

2,4-dichloro-1-(4-chloro-2-(3-(cyclohexylsulfonyl)propoxy)phenoxy)benzene (4z): Following

the General Procedure B, purified by preparative TLC, using petroleum ether /EA = 5/1 (v/v) as an eluent. White solid in 53% yield.

^{1}H NMR (400 MHz, $CDCl_3$) δ 7.44 (d, *J* = 2.5 Hz, 1H), 7.10 (dd, *J* = 8.8, 2.5 Hz, 1H), 6.98 (d, *J* = 4.9 Hz, 3H), 6.62 (d, *J* = 8.8 Hz, 1H), 4.09 (t, *J* = 5.6 Hz, 2H), 2.83 – 2.77 (m, 2H), 2.73 (dt, *J* = 12.2, 3.5 Hz, 1H), 2.25 – 2.16 (m, 2H), 2.12 – 2.04 (m, 2H), 1.93 (dt, *J* = 12.6, 3.1 Hz, 2H), 1.76 – 1.69 (m, 1H), 1.47 (qd, *J* = 12.4, 3.4 Hz, 2H), 1.35 – 1.19 (m, 3H).

^{13}C NMR (101 MHz, $CDCl_3$) δ 152.46, 150.29, 142.58, 130.93, 130.12, 127.71, 127.65, 123.89, 122.49, 121.69, 117.33, 114.98, 66.99, 61.39, 45.37, 25.00, 24.98, 24.95, 21.23.

HRMS (ESI/QTOF) m/z: $[M + Na]^+$ Calcd for $C_{21}H_{23}Cl_3O_4SNa^+$ 499.0275; Found 499.0282.

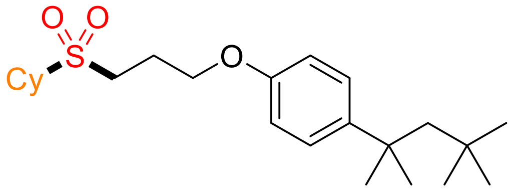

1-(3-(cyclohexylsulfonyl)propoxy)-4-(2,4,4-trimethylpentan-2-yl)benzene (4aa): Following the General Procedure B, purified by preparative TLC, using petroleum ether /EA = 10/1 (v/v) as an eluent. White solid in 58% yield.

^{1}H NMR (400 MHz, $CDCl_3$) δ 7.25 (d, *J* = 8.8 Hz, 2H), 6.78 (d, *J* = 8.8 Hz, 2H), 4.06 (t, *J* = 5.7 Hz, 2H), 3.15 – 3.10 (m, 2H), 2.85 (tt, *J* = 12.2, 3.5 Hz, 1H), 2.34 – 2.25 (m, 2H), 2.21 – 2.13 (m, 2H), 1.92 (dd, *J* = 13.2, 3.3 Hz, 2H), 1.69 (s, 3H), 1.54 (qd, *J* = 12.4, 3.7 Hz, 2H), 1.35 – 1.21 (m, 9H), 0.70 (s, 9H).

^{13}C NMR (101 MHz, $CDCl_3$) δ 155.93, 142.67, 127.08, 113.54, 65.60, 61.13, 56.85, 46.31, 37.88, 32.23, 31.69, 31.60, 25.01, 24.99, 21.64.

HRMS (ESI/QTOF) m/z: $[M + Na]^+$ Calcd for $C_{21}H_{38}O_3SNa^+$ 417.2434; Found 417.2440.

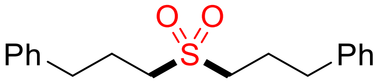

(sulfonylbis(propane-3,1-diyl))dibenzene (5a): Following the General Procedure C, purified by preparative TLC, using petroleum ether /EA = 10/1 (v/v) as an eluent. White solid in 78% yield.

^{1}H NMR (400 MHz, $CDCl_3$) δ 7.24 – 7.18 (m, 4H), 7.16 – 7.10 (m, 3H), 7.07 (d, *J* = 7.8 Hz, 3H), 2.85 – 2.75 (m, 4H), 2.66 (t, *J* = 7.5 Hz, 4H), 2.09 – 1.97 (m, 4H).

^{13}C NMR (101 MHz, $CDCl_3$) δ 139.72, 128.58, 128.34, 126.44, 51.79, 34.11, 23.30.

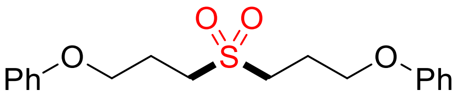

((sulfonylbis(propane-3,1-diyl))bis(oxy))dibenzene (5b): Following the General Procedure C, purified by preparative TLC, using petroleum ether /EA = 8/1 (v/v) as an eluent. White solid in 65% yield.

^{1}H NMR (400 MHz, $CDCl_3$) δ 7.24 – 7.18 (m, 4H), 6.89 (tt, *J* = 7.4, 1.1 Hz, 2H), 6.83 – 6.78 (m, 4H), 4.03 (t, *J* = 5.8 Hz, 4H), 3.22 – 3.14 (m, 4H), 2.32 – 2.23 (m, 4H).

^{13}C NMR (101 MHz, $CDCl_3$) δ 158.28, 129.54, 121.19, 114.41, 65.39, 50.07, 22.21.

HRMS (ESI/QTOF) m/z: $[M + Na]^+$ Calcd for $C_{18}H_{22}O_4SNa^+$ 357.1131; Found 357.1134.

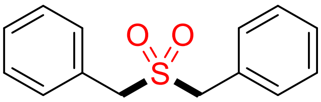

(sulfonylbis(methylene))dibenzene (5c): Following the General Procedure C, purified by preparative TLC, using petroleum ether /EA = 10/1 (v/v) as an eluent. White solid in 91% yield.

^{1}H NMR (400 MHz, $CDCl_3$) δ 7.31 (s, 10H), 4.04 (s, 4H).

^{13}C NMR (101 MHz, $CDCl_3$) δ 130.80, 128.96, 128.91, 127.48, 57.94.

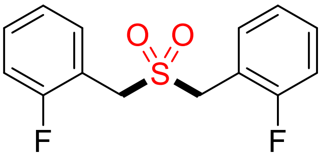

2,2'-(sulfonylbis(methylene))bis(fluorobenzene) (5d): Following the General Procedure C, purified by preparative TLC, using petroleum ether /EA = 10/1 (v/v) as an eluent. White solid in 77% yield.

^{1}H NMR (400 MHz, $CDCl_3$) δ 7.41 (t, *J* = 7.3 Hz, 2H), 7.31 (q, *J* = 6.8, 6.3 Hz, 2H), 7.09 (dt, *J* = 24.6, 8.3 Hz, 4H), 4.24 (s, 4H).

^{13}C NMR (101 MHz, $CDCl_3$) δ 161.16 (d, *J* = 249.0 Hz), 132.79 (d, *J* = 2.1 Hz), 131.17 (d, *J* = 8.8 Hz), 124.73 (d, *J* = 3.6 Hz), 115.80 (d, *J* = 22.1 Hz), 114.80 (d, *J* = 14.3 Hz), 51.70.

^{19}F NMR (377 MHz, $CDCl_3$) δ -116.32.

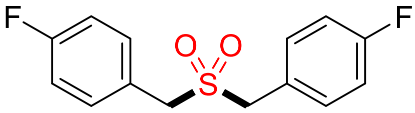

4,4'-(sulfonylbis(methylene))bis(fluorobenzene) (5e): (Following the General Procedure C, purified by preparative TLC, using petroleum ether /EA = 10/1 (v/v) as an eluent. White solid in

90% yield.

^{1}H NMR (400 MHz, $CDCl_3$) δ 7.31 – 7.25 (m, 4H), 7.03 (t, *J* = 8.0 Hz, 4H), 4.04 (s, 4H).

^{13}C NMR (101 MHz, $CDCl_3$) δ 163.22 (d, *J* = 249.2 Hz), 132.59 (d, *J* = 8.4 Hz), 123.05 (d, *J* = 3.3 Hz), 116.14 (d, *J* = 21.8 Hz), 57.18.

^{19}F NMR (377 MHz, $CDCl_3$) δ -111.89.

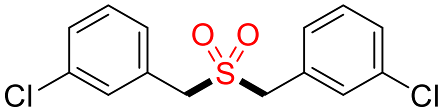

3,3'-(sulfonylbis(methylene))bis(chlorobenzene) (5f): (Following the General Procedure C, purified by preparative TLC, using petroleum ether /EA = 5/1 (v/v) as an eluent. White solid in 86% yield.

^{1}H NMR (400 MHz, DMSO-d_6) δ 7.44 – 7.41 (m, 6H), 7.36 – 7.32 (m, 2H), 4.54 (s, 4H).

^{13}C NMR (101 MHz, DMSO-d_6) δ 133.91, 131.76, 131.33, 131.25, 130.82, 129.39, 58.01.

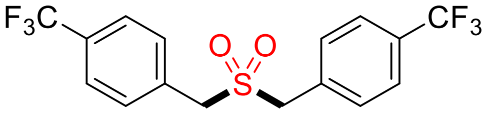

4,4'-(sulfonylbis(methylene))bis((trifluoromethyl)benzene) (5g): Following the General Procedure C, purified by preparative TLC, using petroleum ether /EA = 5/1 (v/v) as an eluent. White solid in 85% yield.

^{1}H NMR (400 MHz, $CDCl_3$) δ 7.61 (d, *J* = 7.8 Hz, 4H), 7.44 (d, *J* = 7.8 Hz, 4H), 4.15 (s, 4H).

^{13}C NMR (101 MHz, $CDCl_3$) δ 131.67, 131.33, 131.00, 126.02 (q, *J* = 3.8 Hz), 123.74 (q, *J* = 278.10 Hz)125.13, 122.35, 58.01.

^{19}F NMR (377 MHz, $CDCl_3$) δ -62.85.

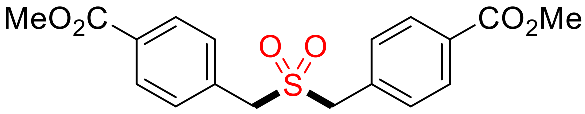

dimethyl 4,4'-(sulfonylbis(methylene))dibenzoate (5h): Following the General Procedure C, purified by preparative TLC, using petroleum ether /EA = 3/1 (v/v) as an eluent. White solid in 81% yield.

^{1}H NMR (400 MHz, DMSO-d_6) δ 7.94 (d, *J* = 8.0 Hz, 4H), 7.50 (d, *J* = 7.7 Hz, 4H), 4.60 (s, 4H), 3.83 (s, 6H).

^{13}C NMR (101 MHz, DMSO-d_6) δ 166.92, 134.37, 132.55, 130.56, 130.21, 58.48, 53.22.

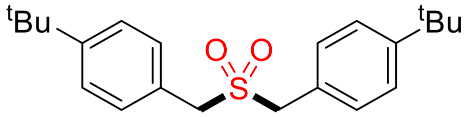

4,4'-(sulfonylbis(methylene))bis(*tert*-butylbenzene) (5i): Following the General Procedure C, purified by preparative TLC, using petroleum ether /EA = 10/1 (v/v) as an eluent. White solid in 93% yield.

^{1}H NMR (400 MHz, $CDCl_3$) δ 7.35 – 7.31 (m, 4H), 7.24 (d, *J* = 8.4 Hz, 4H), 4.02 (s, 4H), 1.24 (s, 18H).

^{13}C NMR (101 MHz, $CDCl_3$) δ 151.94, 130.48, 125.87, 124.45, 57.37, 34.58, 31.18.

NMR Spectra of Substrates and Products

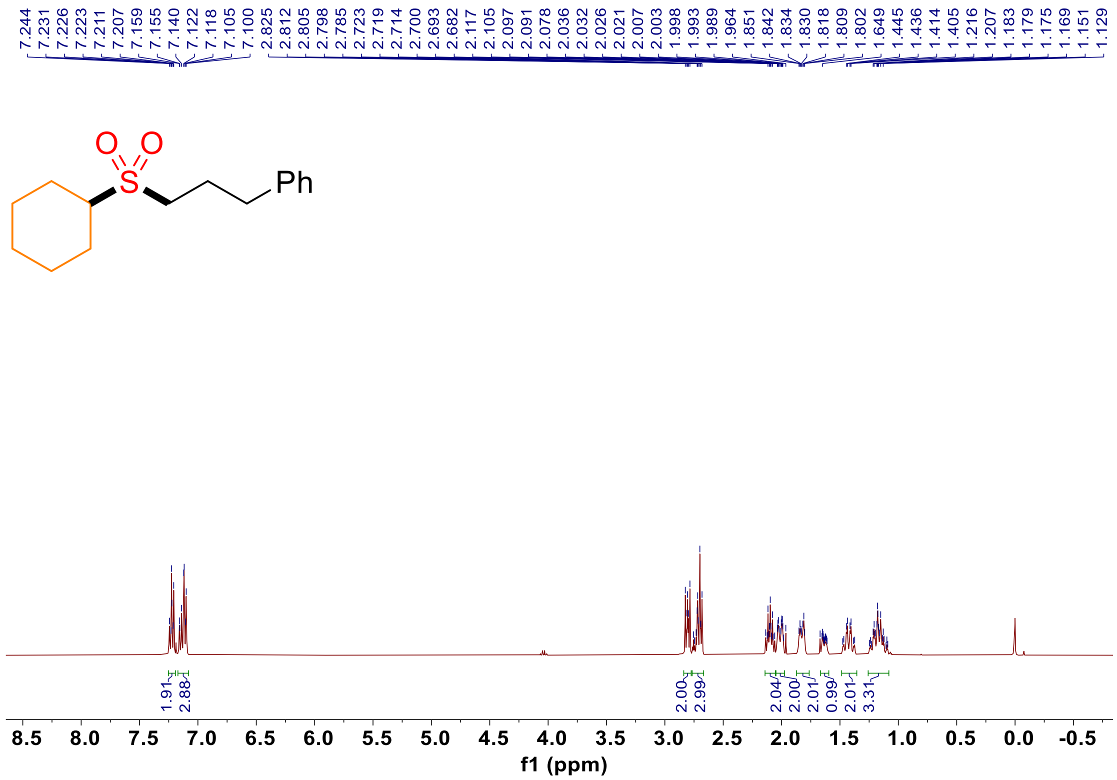

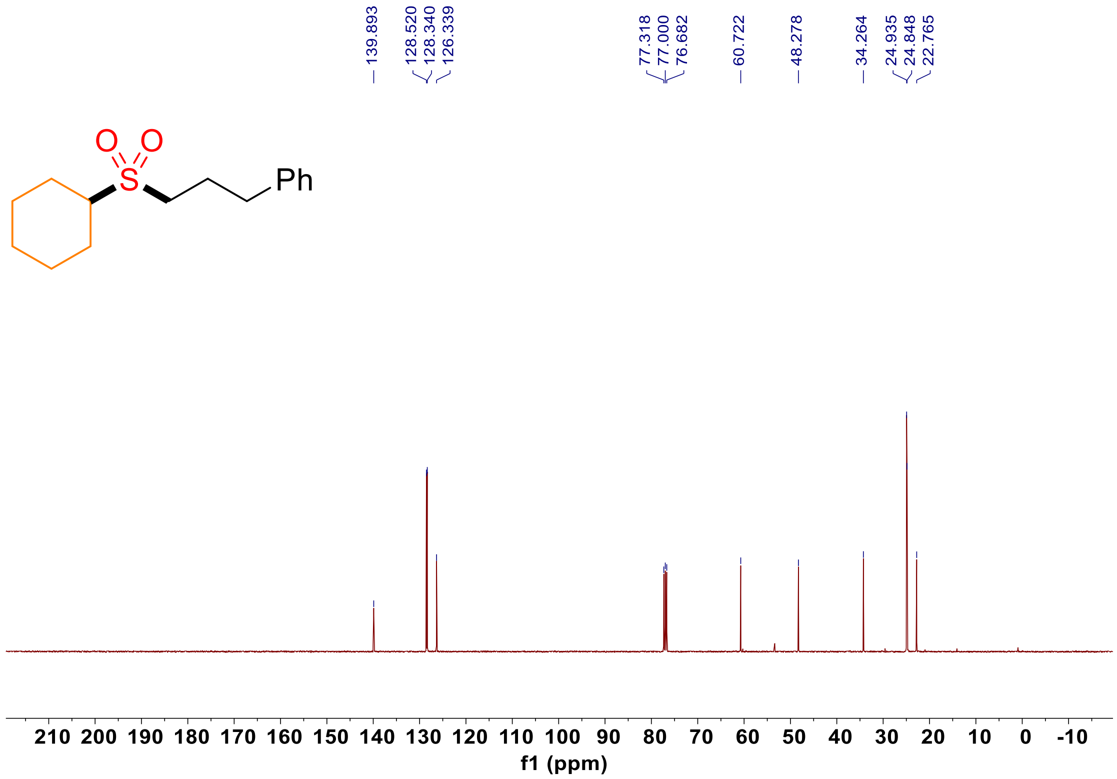

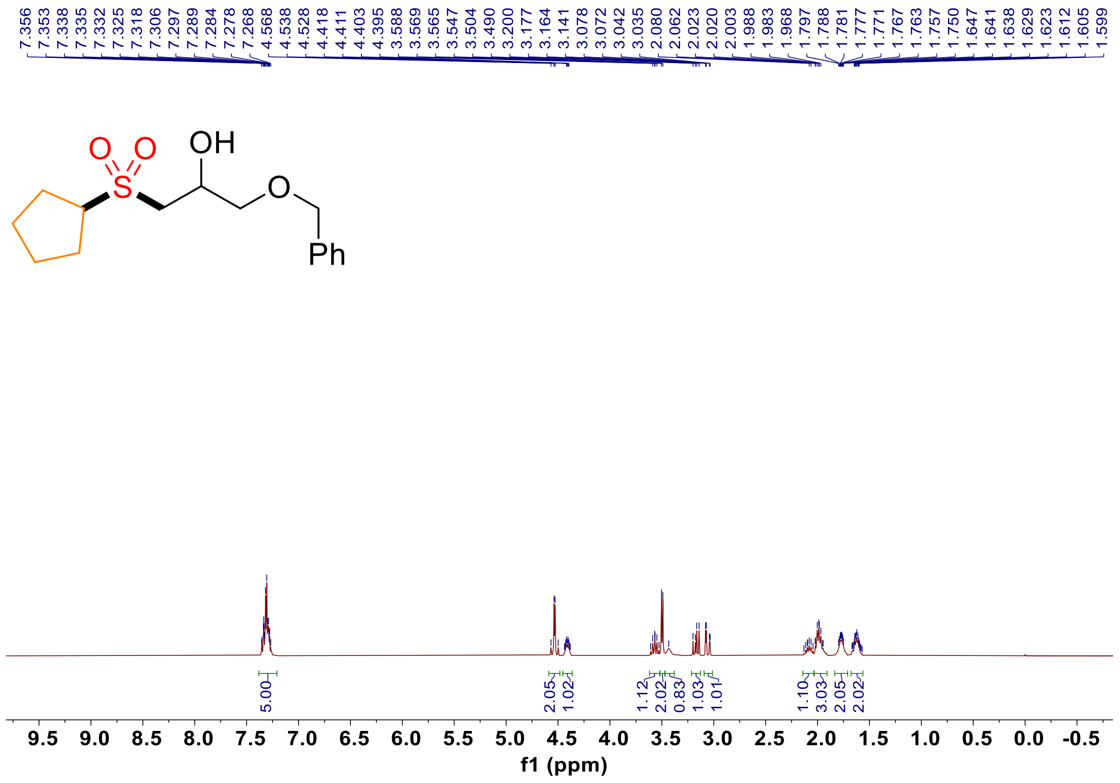

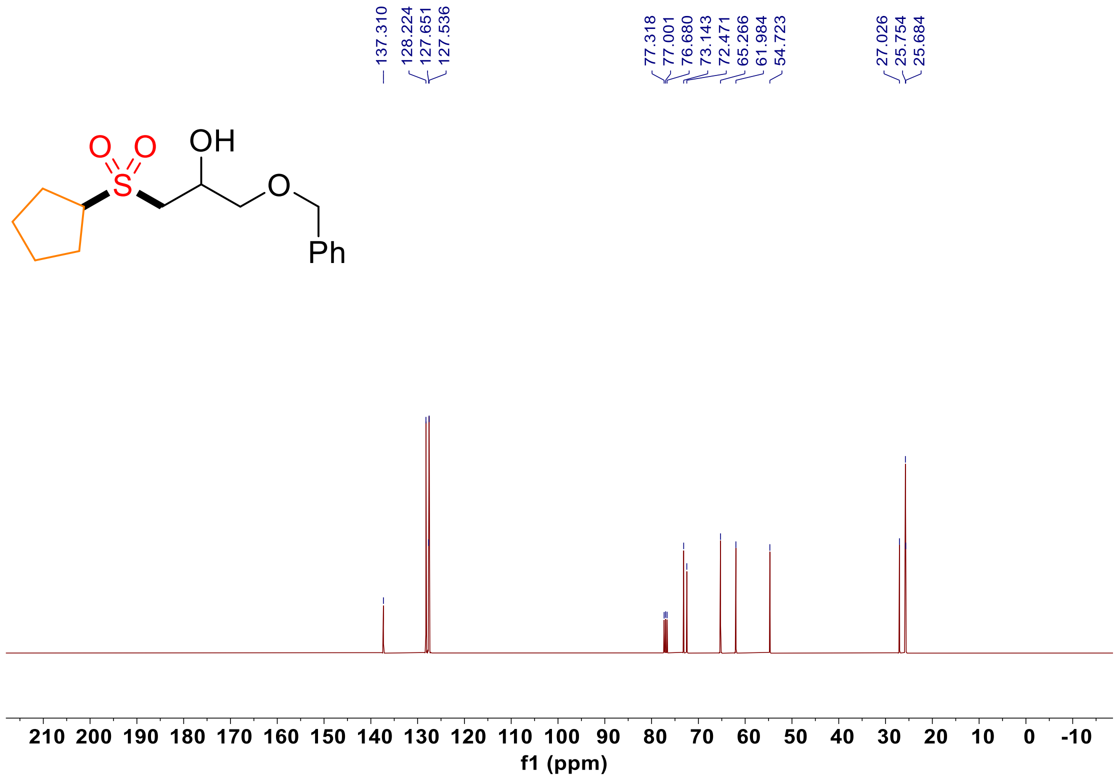

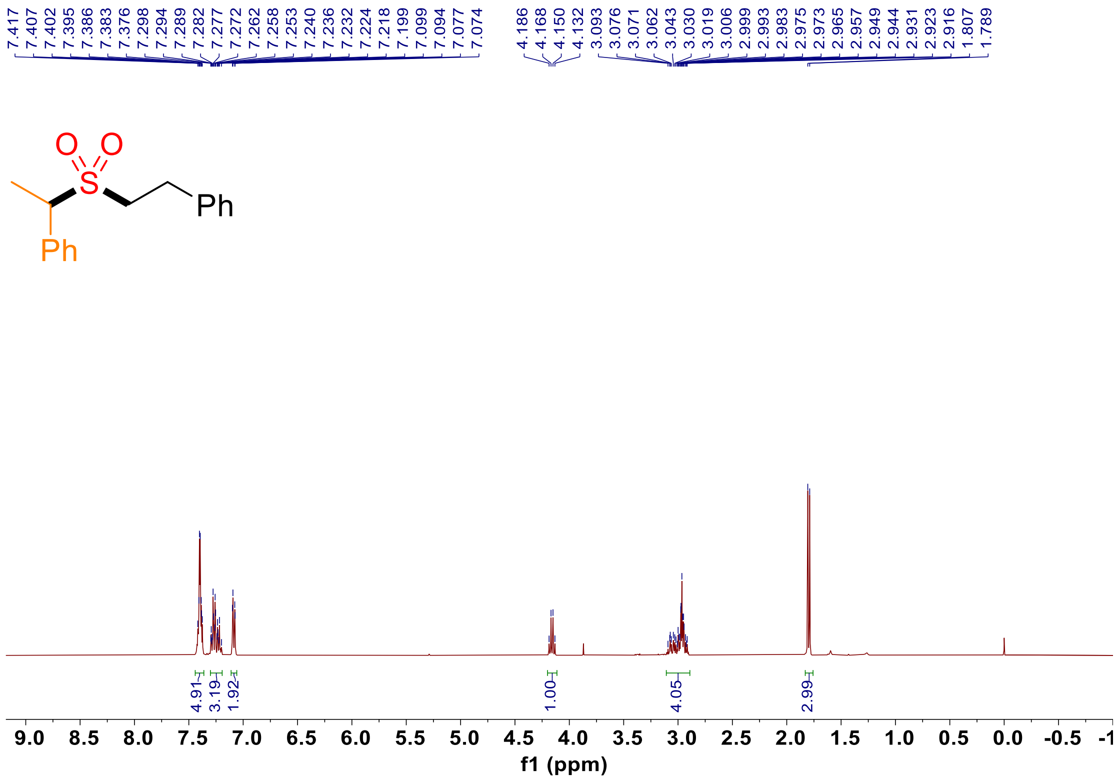
7.417 7.407 7.402 7.395 7.386 7.383 7.376 7.298 7.294 7.289 7.282 7.277 7.272 7.262 7.258 7.253 7.240 7.236 7.232 7.224 7.218 7.199 7.099 7.094 7.077 7.074
4.186 4.168 4.150 4.132 3.093 3.076 3.071 3.062 3.043 3.030 3.019 3.006 2.999 2.993 2.983 2.975 2.973 2.965 2.957 2.949 2.944 2.931 2.923 2.916 1.807 1.789
O O
S
Ph
Ph
4.91
3.19
1.92
1.00
4.05
2.99
9.0 8.5 8.0 7.5 7.0 6.5 6.0 5.5 5.0 4.5 4.0 3.5 3.0 2.5 2.0 1.5 1.0 0.5 0.0 -0.5 -1
f1 (ppm)

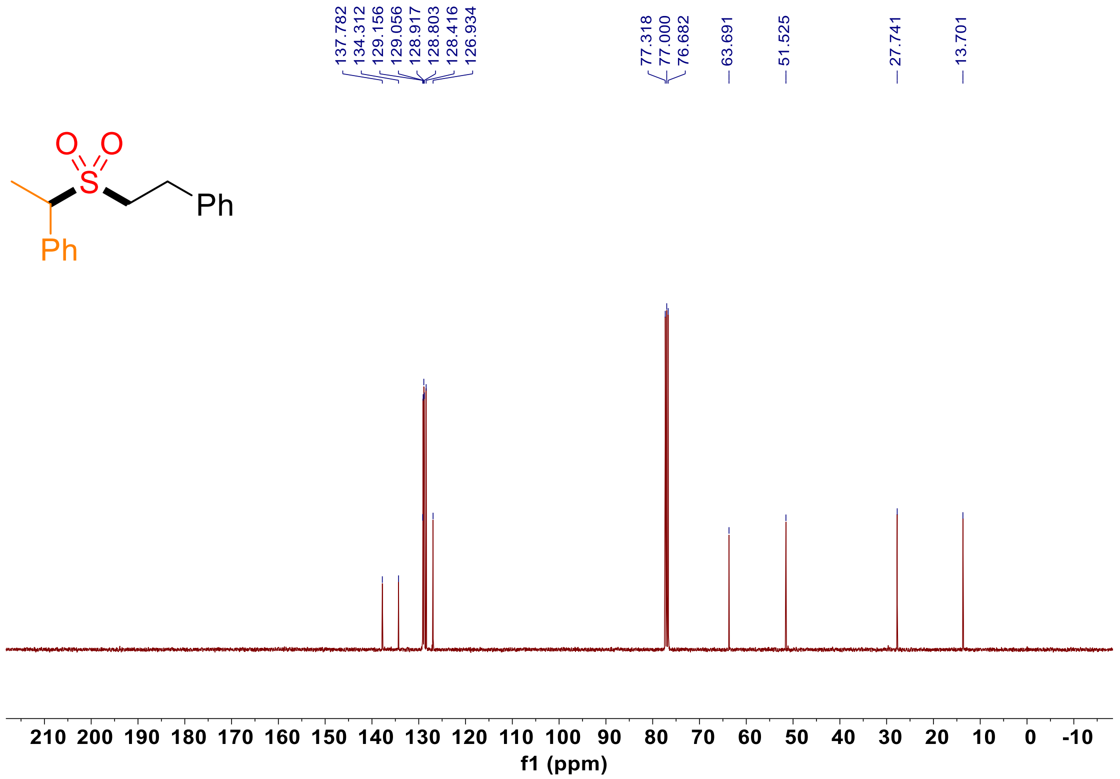
137.782 134.312 129.156 129.056 128.917 128.803 128.416 126.934
77.318 77.000 76.682
63.691
51.525
27.741
13.701
O O
S
Ph
Ph
210 200 190 180 170 160 150 140 130 120 110 100 90 80 70 60 50 40 30 20 10 0 -10
f1 (ppm)

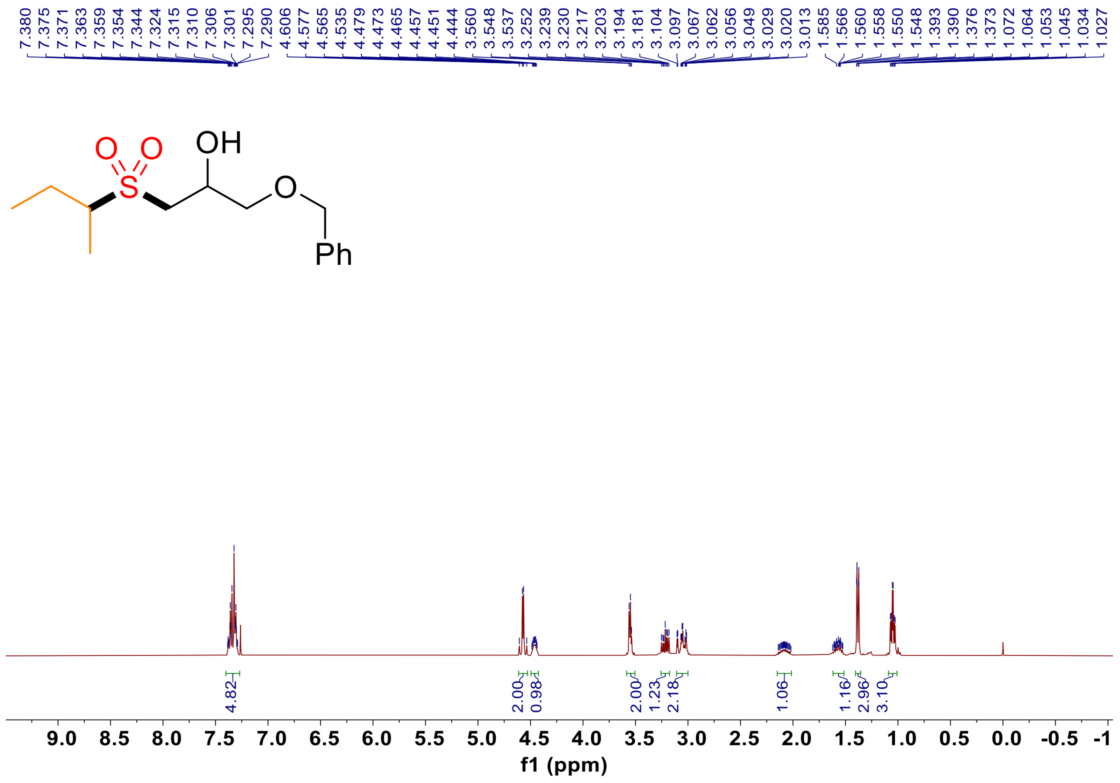

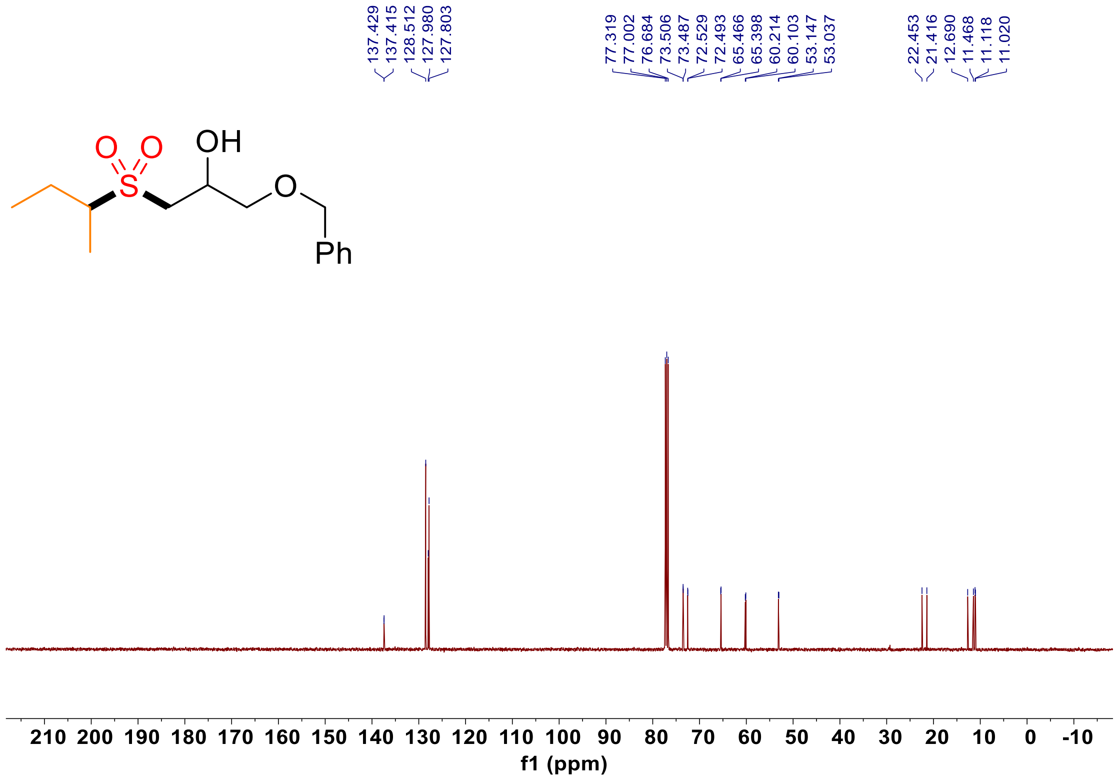

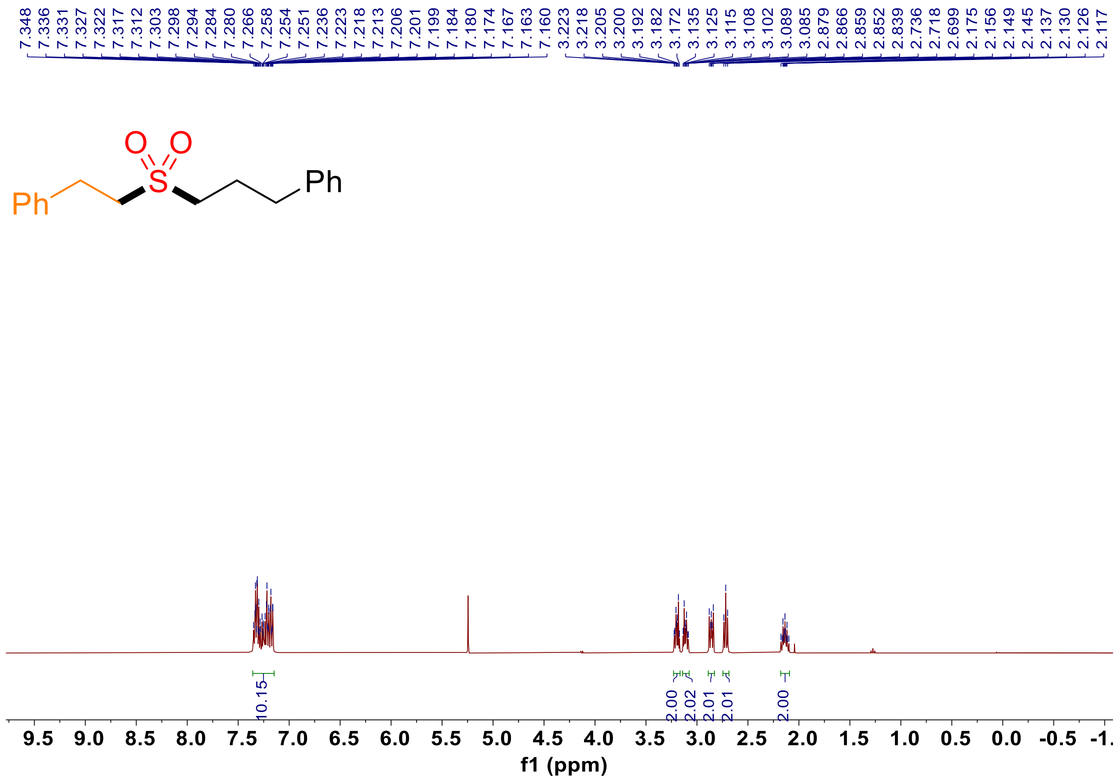

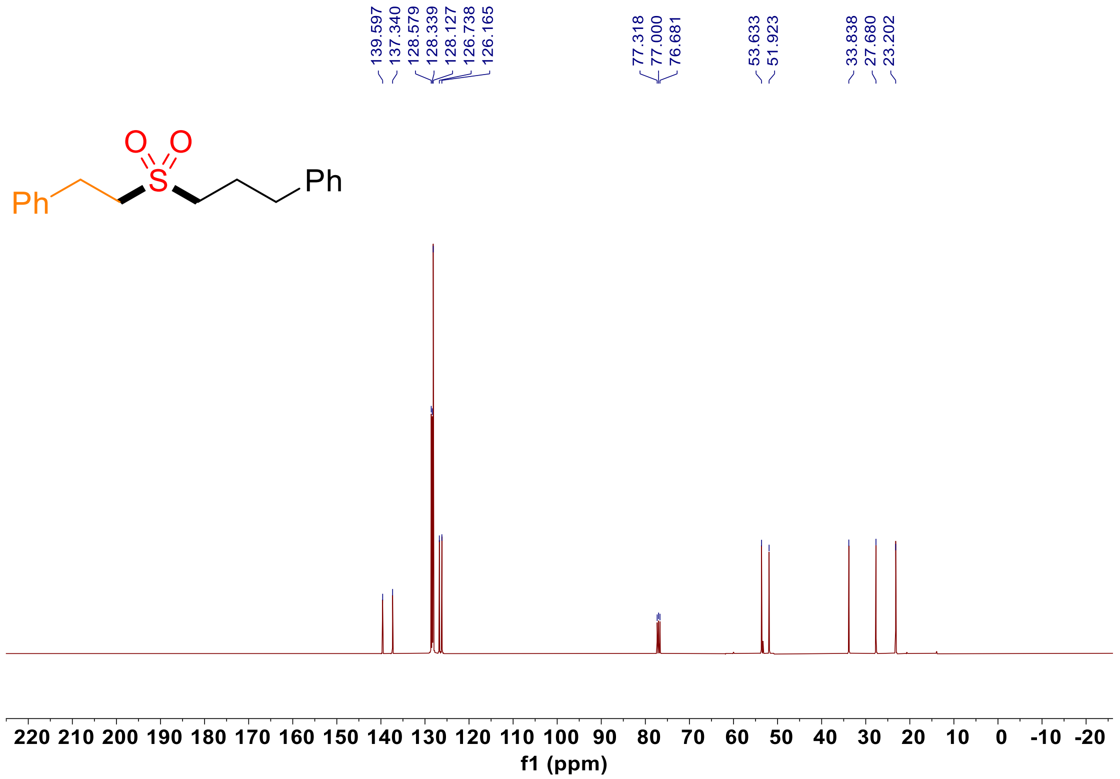

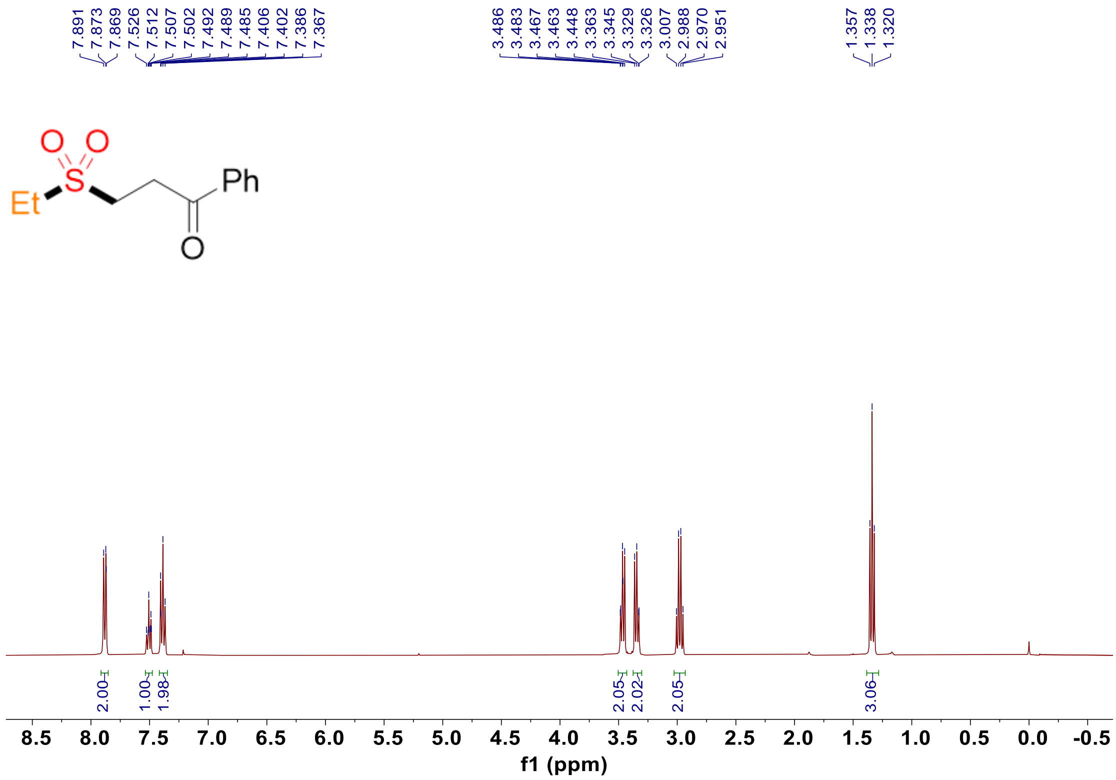
7.891
7.873
7.869
7.526
7.512
7.507
7.502
7.492
7.489
7.485
7.406
7.402
7.386
7.367
3.486
3.483
3.467
3.463
3.448
3.363
3.345
3.329
3.326
3.007
2.988
2.970
2.951
1.357
1.338
1.320
Et
S
O
O
Ph
O
2.00
1.00
1.98
2.05
2.02
2.05
3.06
8.5 8.0 7.5 7.0 6.5 6.0 5.5 5.0 4.5 4.0 3.5 3.0 2.5 2.0 1.5 1.0 0.5 0.0 -0.5
f1 (ppm)

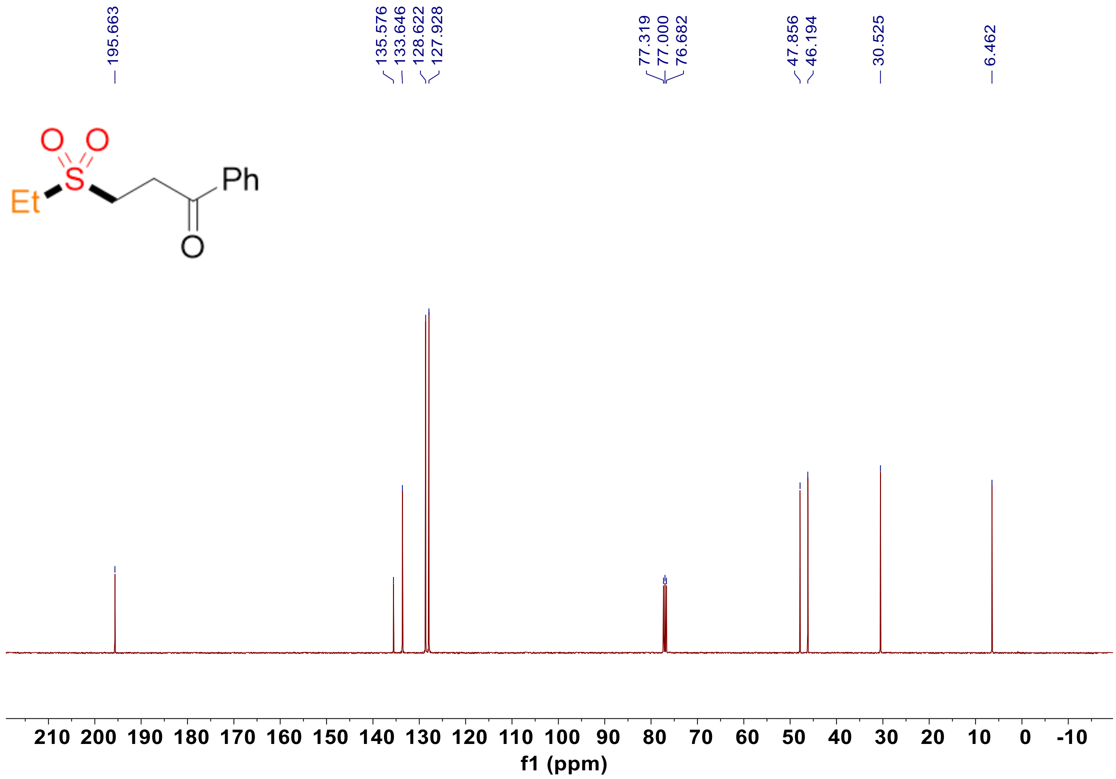
195.663
135.576
133.646
128.622
127.928
77.319
77.000
76.682
47.856
46.194
30.525
6.462
Et
S
O
O
Ph
O
210 200 190 180 170 160 150 140 130 120 110 100 90 80 70 60 50 40 30 20 10 0 -10
f1 (ppm)

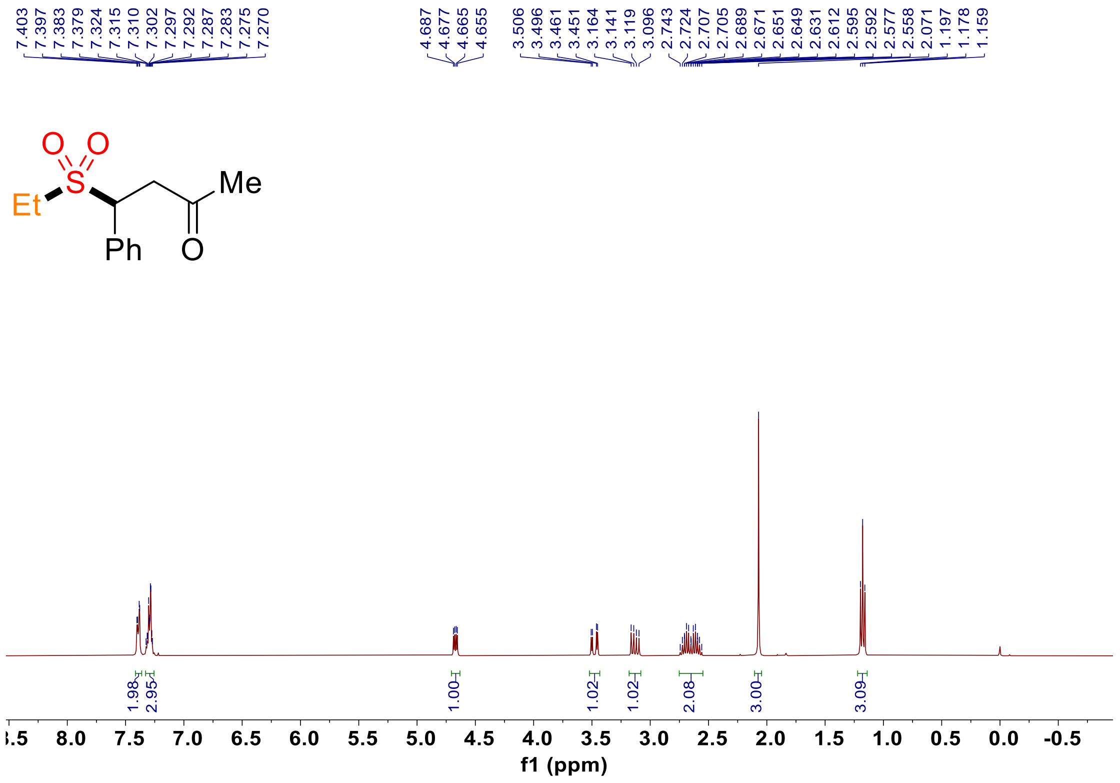

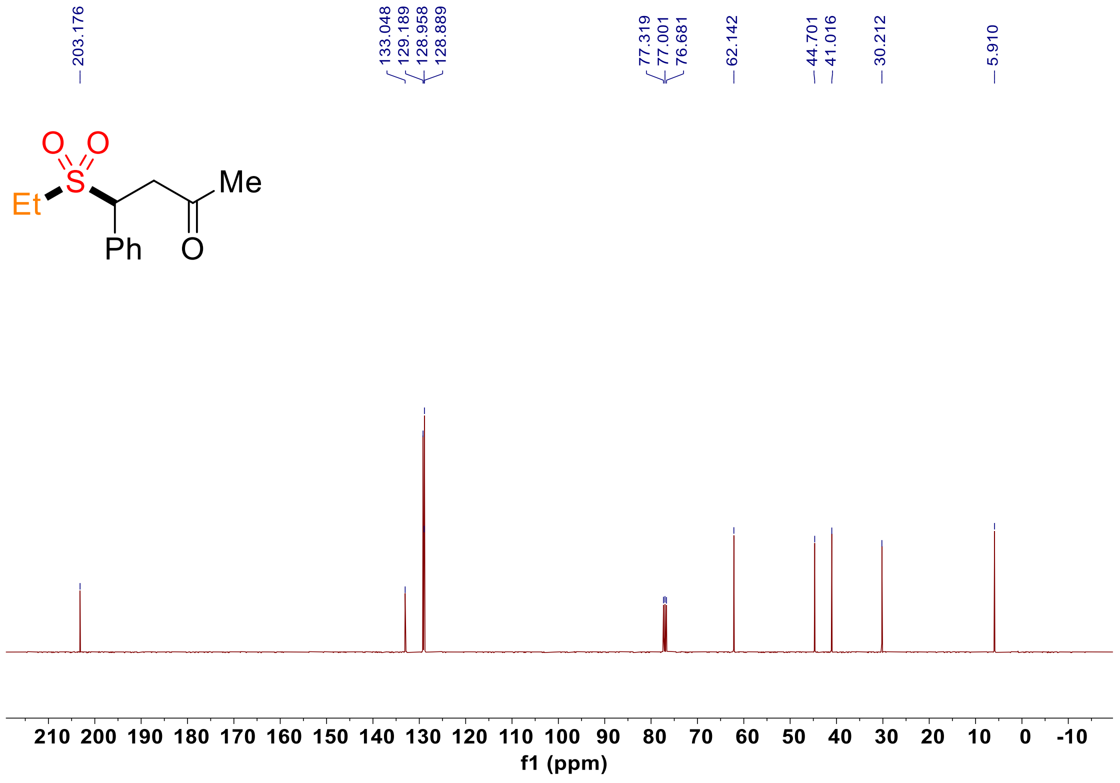

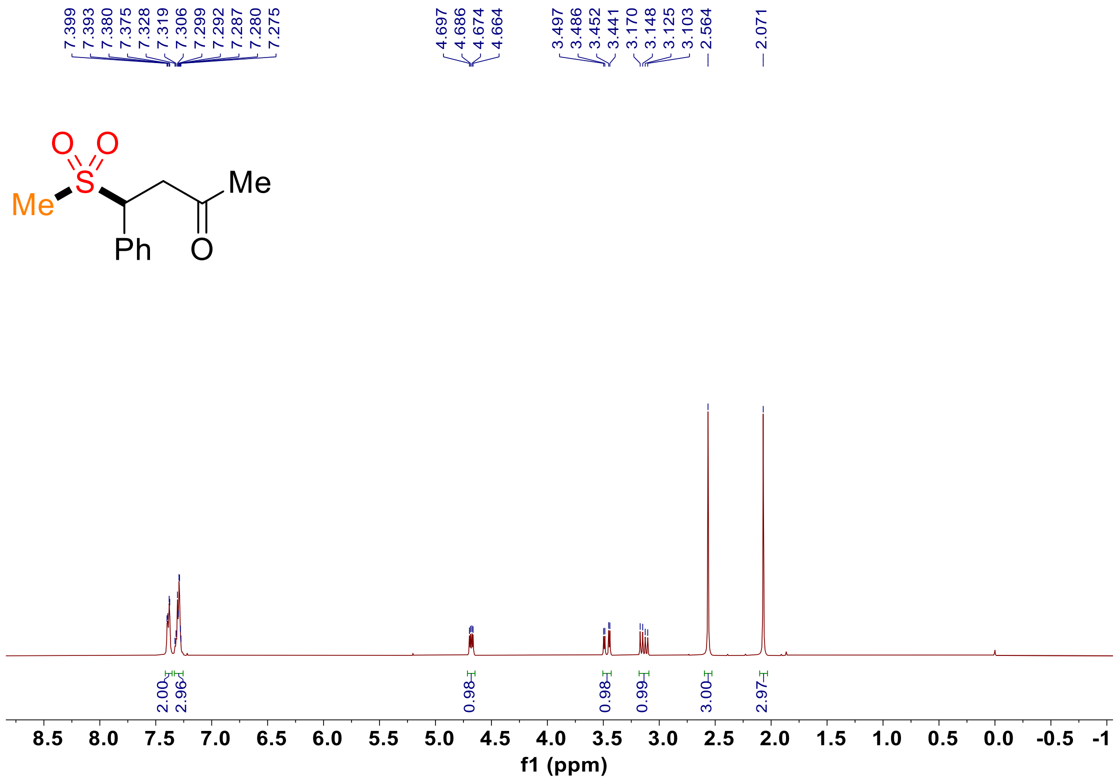
7.399 7.393 7.380 7.375 7.328 7.319 7.306 7.299 7.292 7.287 7.280 7.275
4.697 4.686 4.674 4.664
3.497 3.486 3.452 3.441 3.170 3.148 3.125 3.103 2.564
2.071
O O
Me
S
Me
Ph O
2.00 2.96 0.98 0.98 0.99 3.00 2.97
8.5 8.0 7.5 7.0 6.5 6.0 5.5 5.0 4.5 4.0 3.5 3.0 2.5 2.0 1.5 1.0 0.5 0.0 -0.5 -1
f1 (ppm)

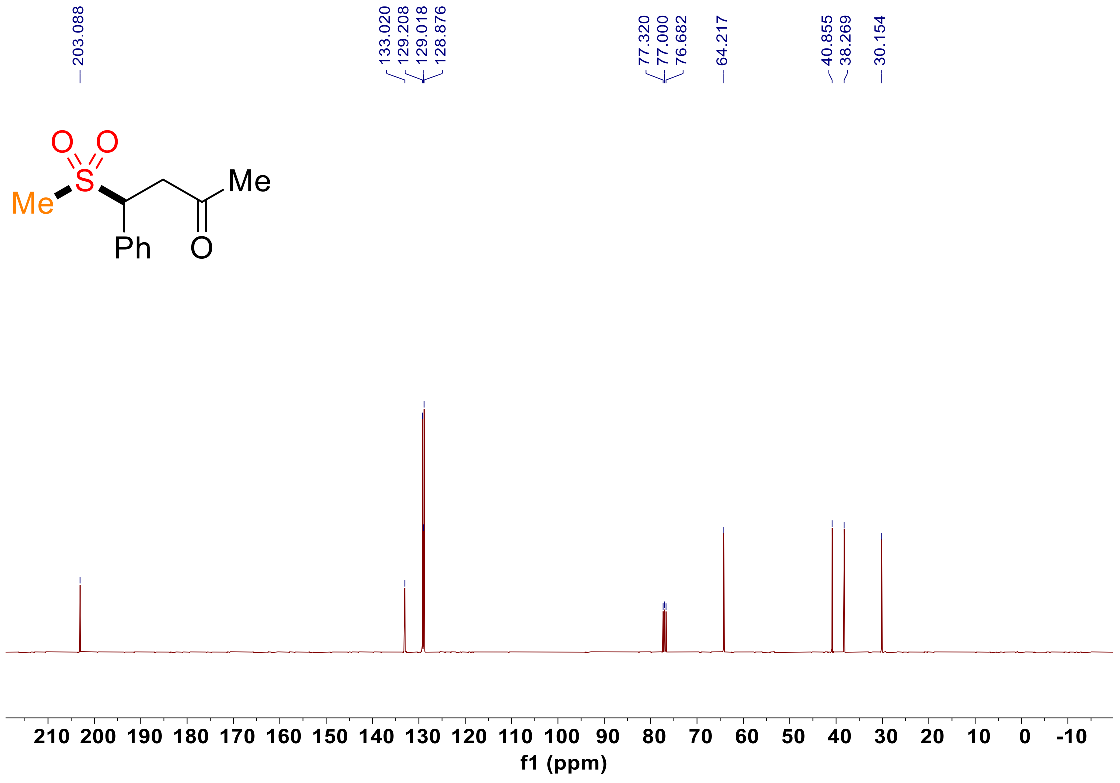
203.088
133.020 129.208 129.018 128.876
77.320 77.000 76.682
64.217
40.855 38.269
30.154
O O
Me
S
Me
Ph O
210 200 190 180 170 160 150 140 130 120 110 100 90 80 70 60 50 40 30 20 10 0 -10
f1 (ppm)

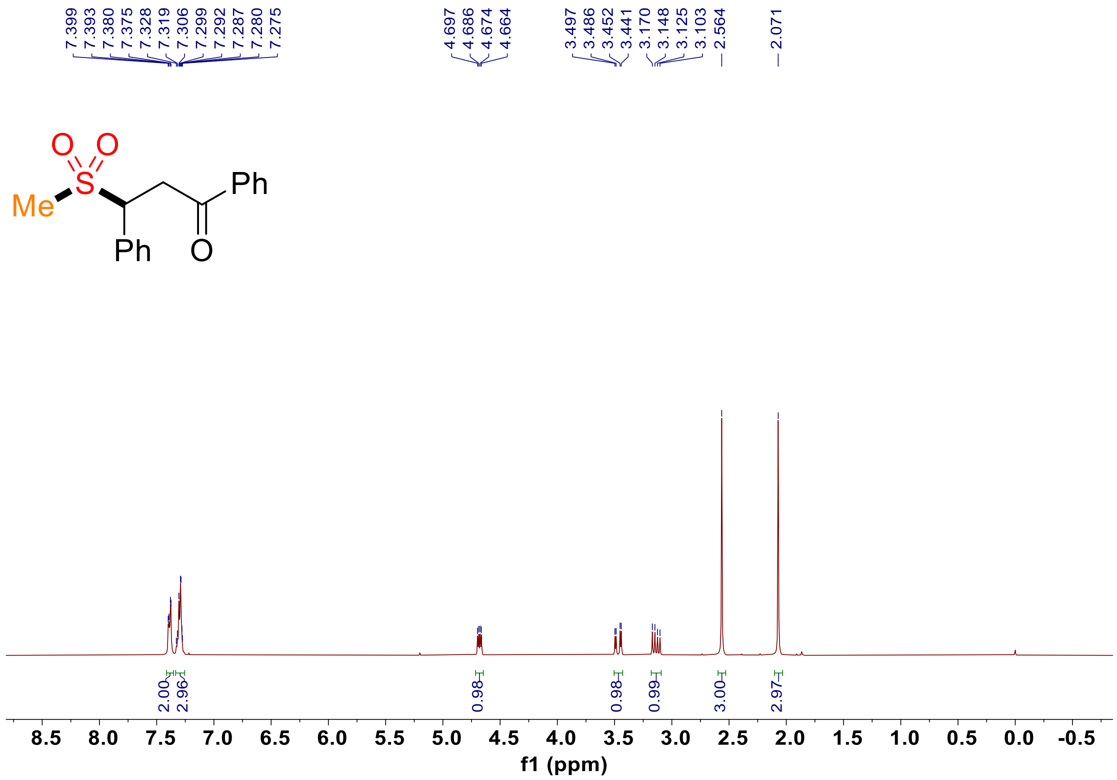
7.399
7.393
7.380
7.375
7.328
7.319
7.306
7.299
7.292
7.287
7.280
7.275
4.697
4.686
4.674
4.664
3.497
3.486
3.452
3.441
3.170
3.148
3.125
3.103
2.564
2.071
Me
S
O
O
Ph
Ph
O
2.00
2.96
0.98
0.98
0.99
3.00
2.97
8.5 8.0 7.5 7.0 6.5 6.0 5.5 5.0 4.5 4.0 3.5 3.0 2.5 2.0 1.5 1.0 0.5 0.0 -0.5
f1 (ppm)

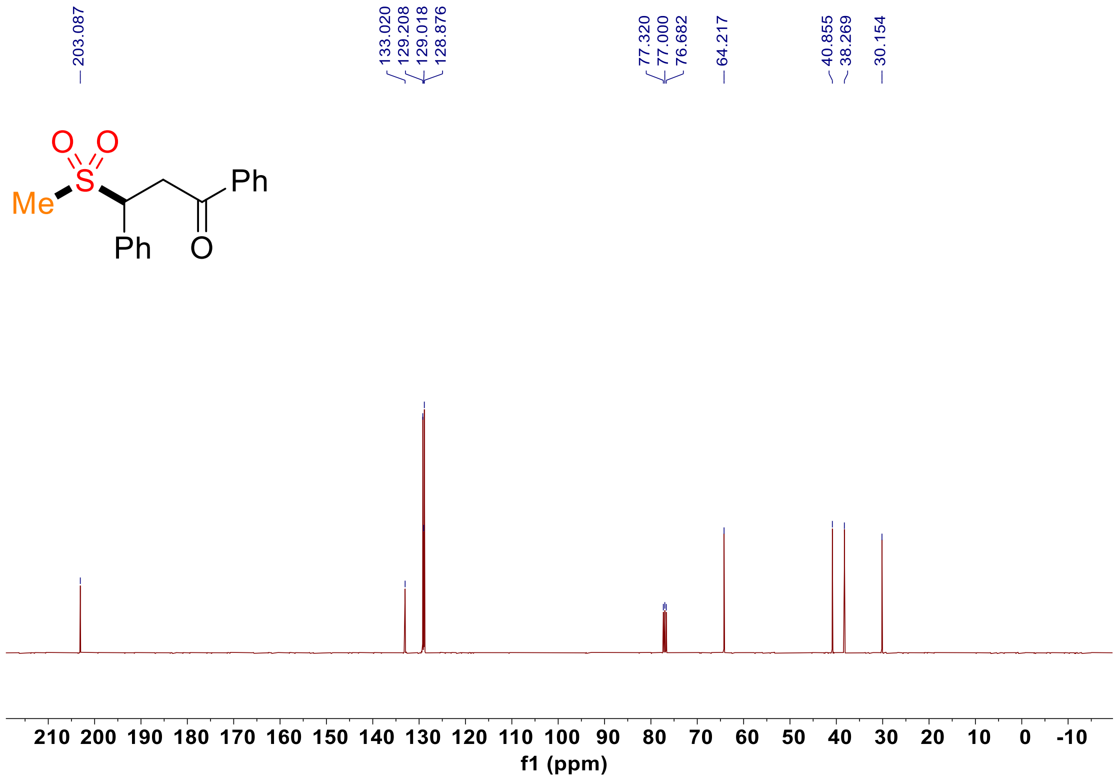
203.087
133.020
129.208
129.018
128.876
77.320
77.000
76.682
64.217
40.855
38.269
30.154
Me
S
O
O
Ph
Ph
O
210 200 190 180 170 160 150 140 130 120 110 100 90 80 70 60 50 40 30 20 10 0 -10
f1 (ppm)

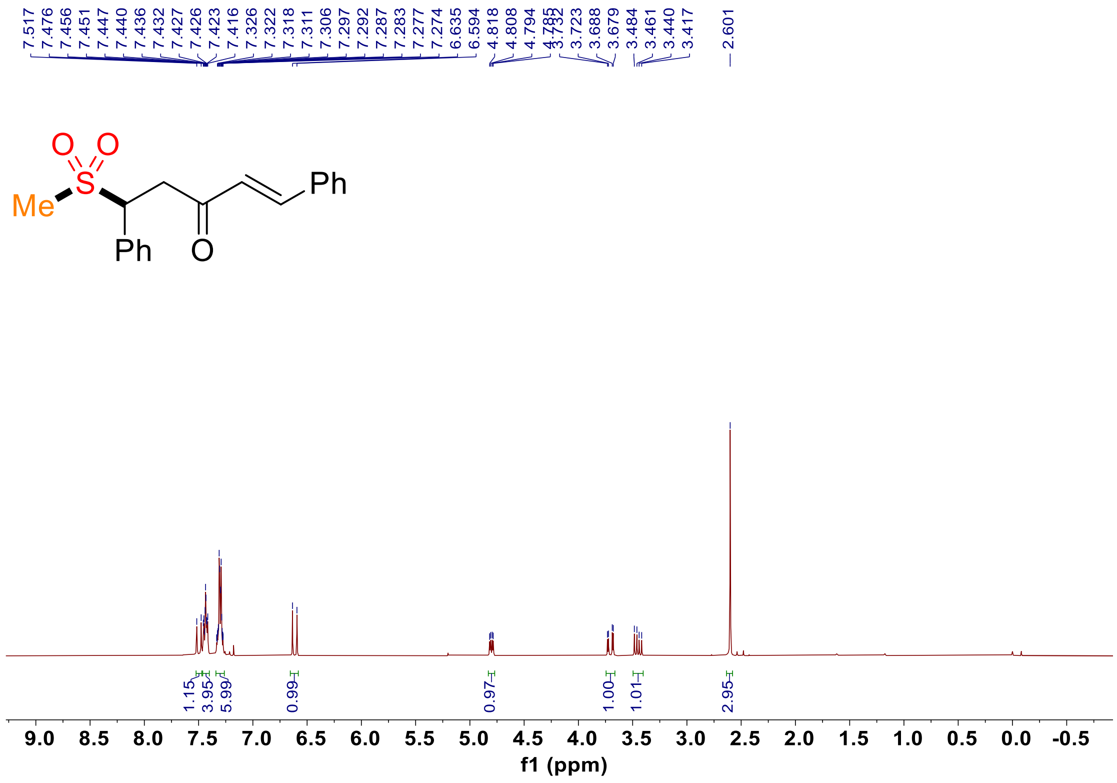
7.517
7.476
7.456
7.451
7.447
7.440
7.436
7.432
7.427
7.426
7.423
7.416
7.326
7.322
7.318
7.311
7.306
7.297
7.292
7.287
7.283
7.277
7.274
6.635
6.594
4.818
4.808
4.794
4.785
3.732
3.723
3.688
3.679
3.484
3.461
3.440
3.417
2.601
O
O
S
Me
Ph
Ph
O
1.15
3.95
5.99
0.99
0.97
1.00
1.01
2.95
9.0 8.5 8.0 7.5 7.0 6.5 6.0 5.5 5.0 4.5 4.0 3.5 3.0 2.5 2.0 1.5 1.0 0.5 0.0 -0.5
f1 (ppm)

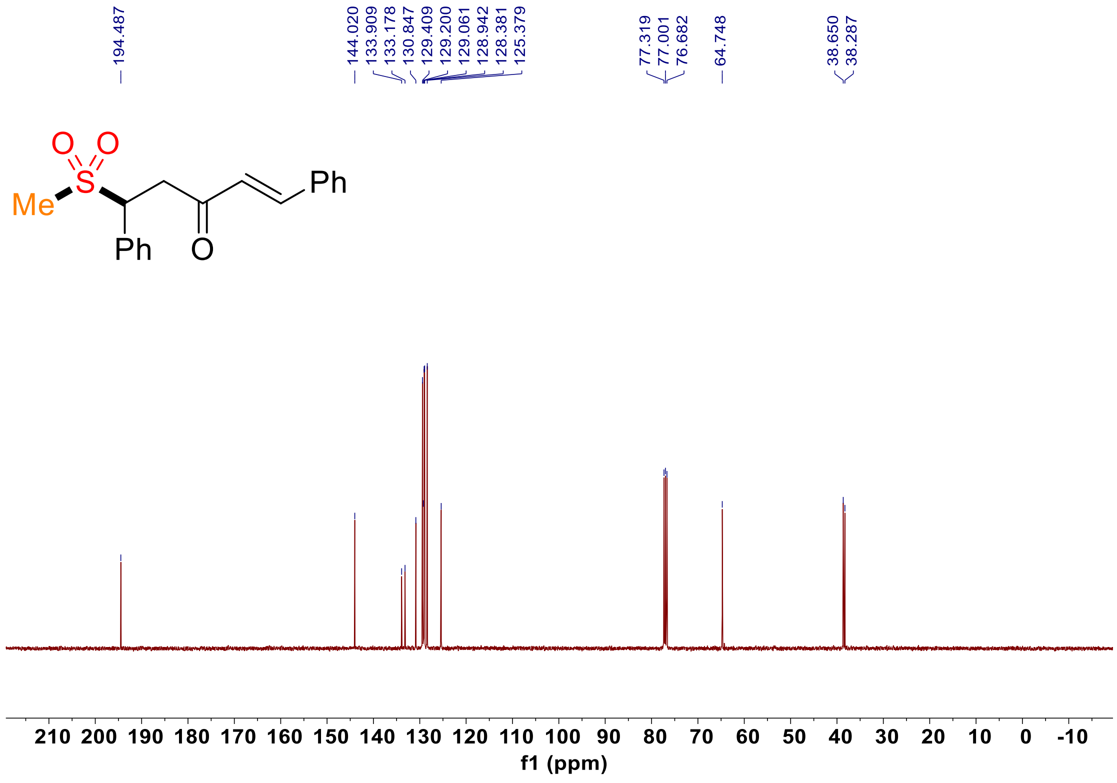
194.487
144.020
133.909
133.178
130.847
129.409
129.200
129.061
128.942
128.381
125.379
77.319
77.001
76.682
64.748
38.650
38.287
O
O
S
Me
Ph
Ph
O
210 200 190 180 170 160 150 140 130 120 110 100 90 80 70 60 50 40 30 20 10 0 -10
f1 (ppm)

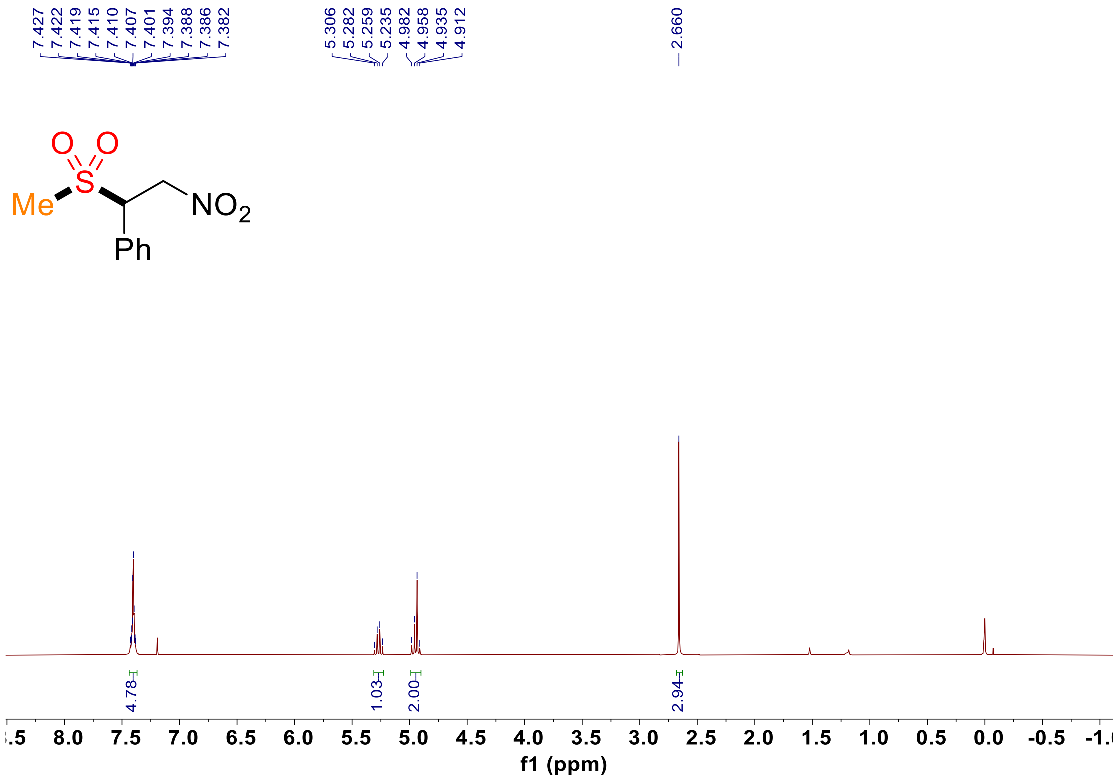
7.427
7.422
7.419
7.415
7.410
7.407
7.401
7.394
7.388
7.386
7.382
5.306
5.282
5.259
5.235
4.982
4.958
4.935
4.912
2.660
Me
O
O
S
NO2
Ph
4.78
1.03
2.00
2.94
f1 (ppm)

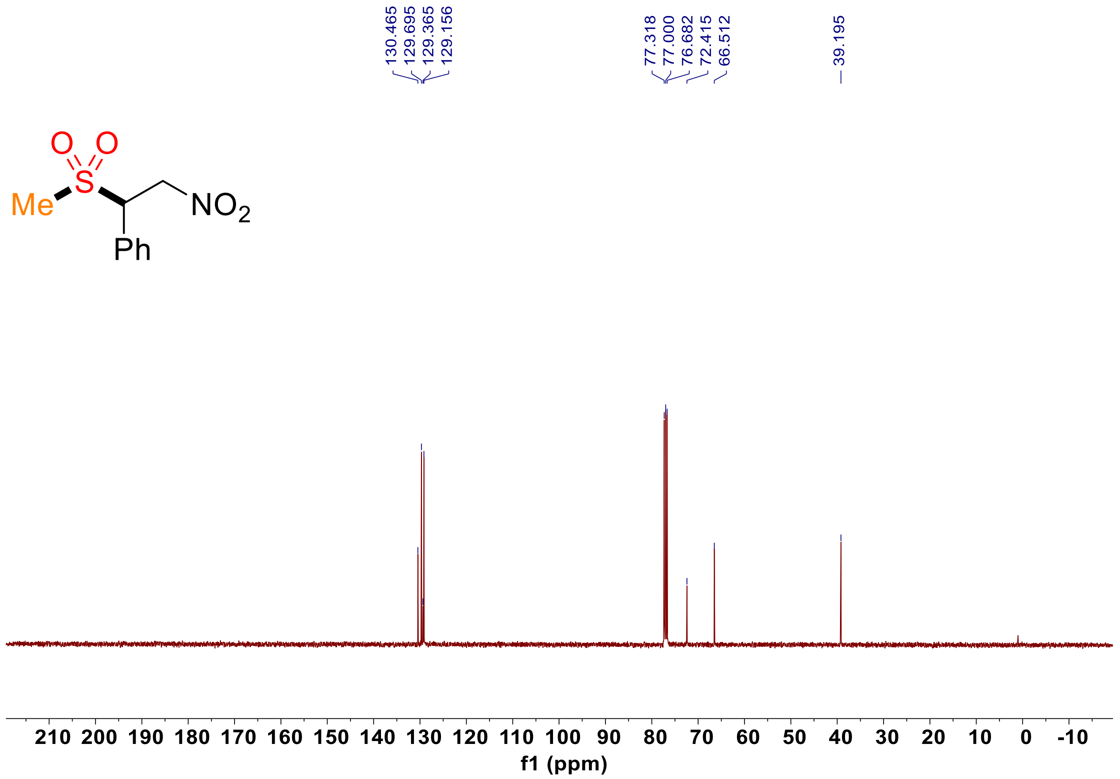
130.465
129.695
129.365
129.156
77.318
77.000
76.682
72.415
66.512
39.195
Me
O
O
S
NO2
Ph
f1 (ppm)

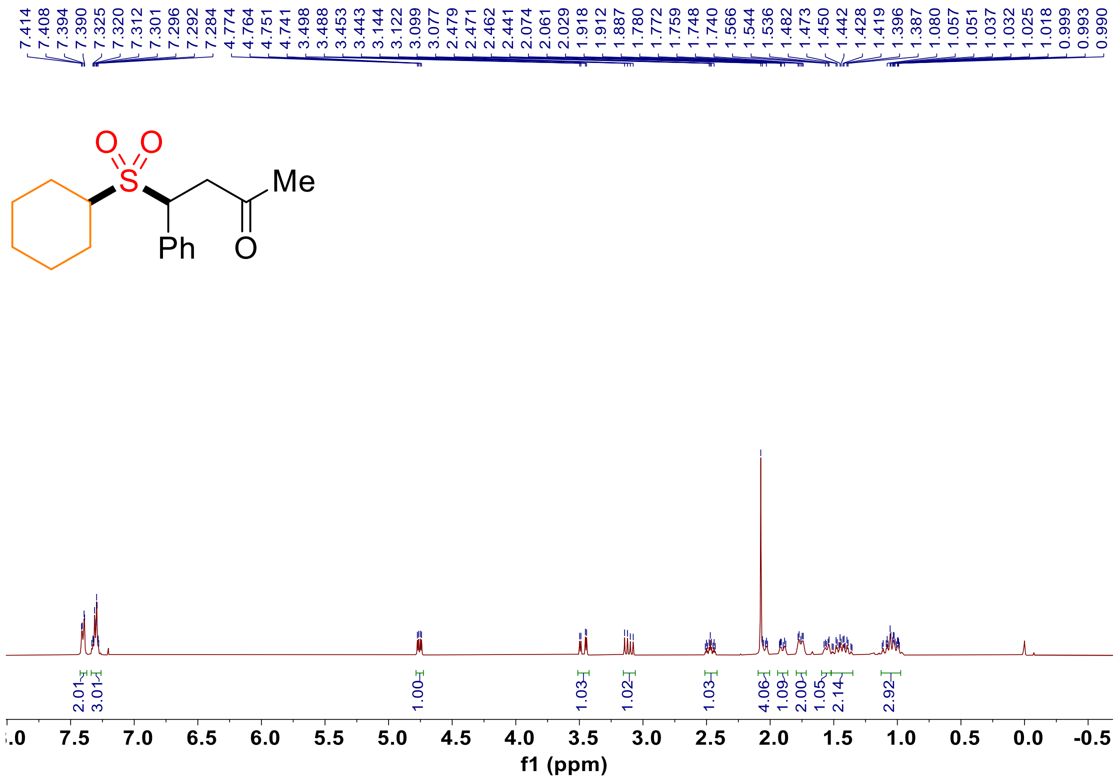

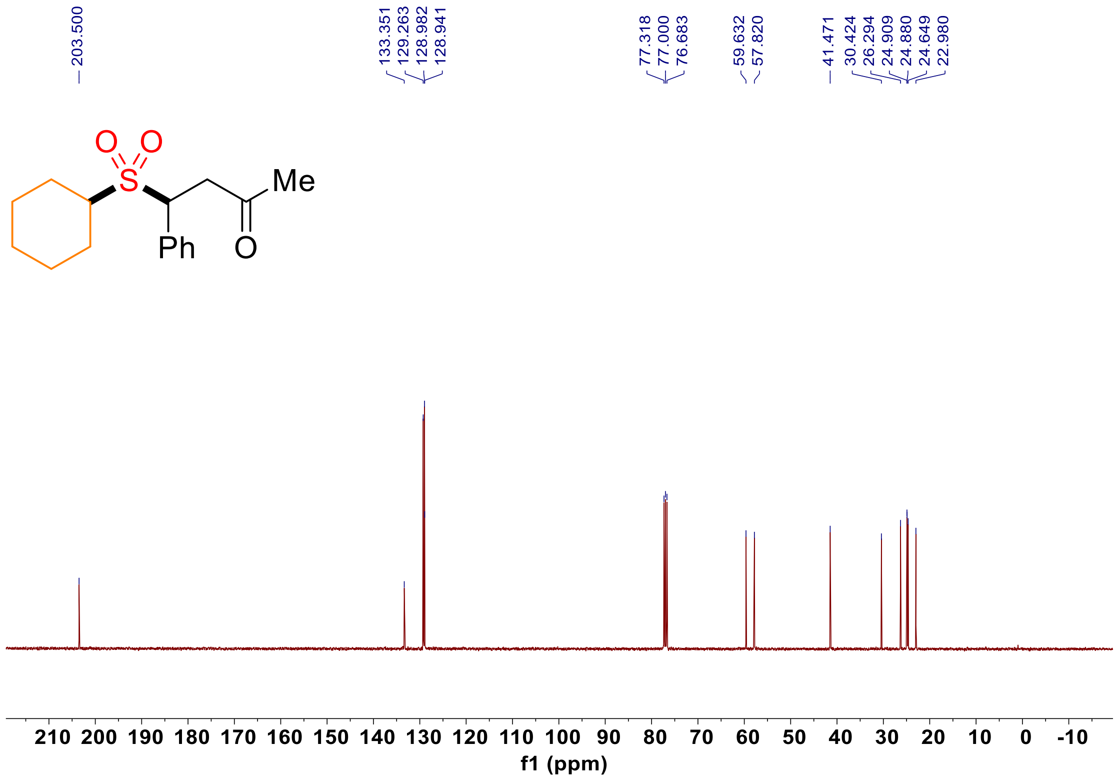

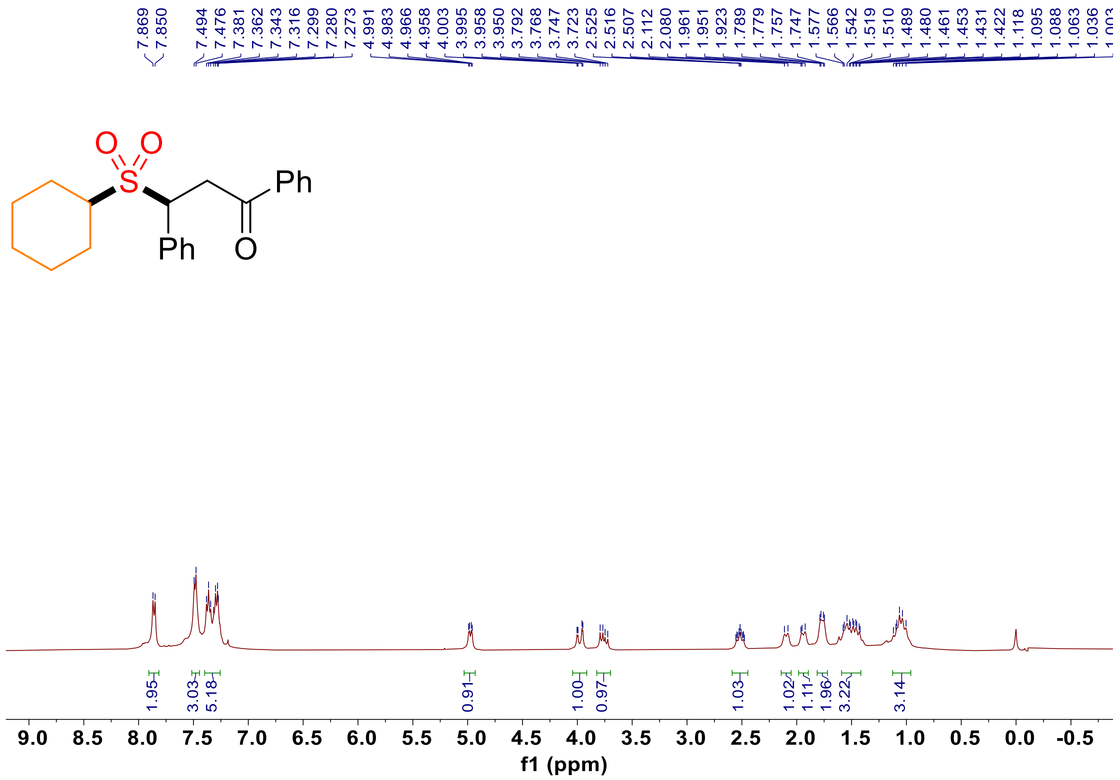

7.869
7.850
7.494
7.476
7.381
7.362
7.343
7.316
7.299
7.280
7.273
4.991
4.983
4.966
4.958
4.003
3.995
3.958
3.950
3.792
3.768
3.747
3.723
2.525
2.516
2.507
2.112
2.080
1.961
1.951
1.923
1.789
1.779
1.757
1.747
1.577
1.566
1.542
1.519
1.510
1.489
1.480
1.461
1.453
1.431
1.422
1.118
1.095
1.088
1.063
1.036
O
O
S
Ph
Ph
O
1.95
3.03
5.18
0.91
1.00
0.97
1.03
1.02
1.11
1.96
3.22
3.14
9.0 8.5 8.0 7.5 7.0 6.5 6.0 5.5 5.0 4.5 4.0 3.5 3.0 2.5 2.0 1.5 1.0 0.5 0.0 -0.5
f1 (ppm)

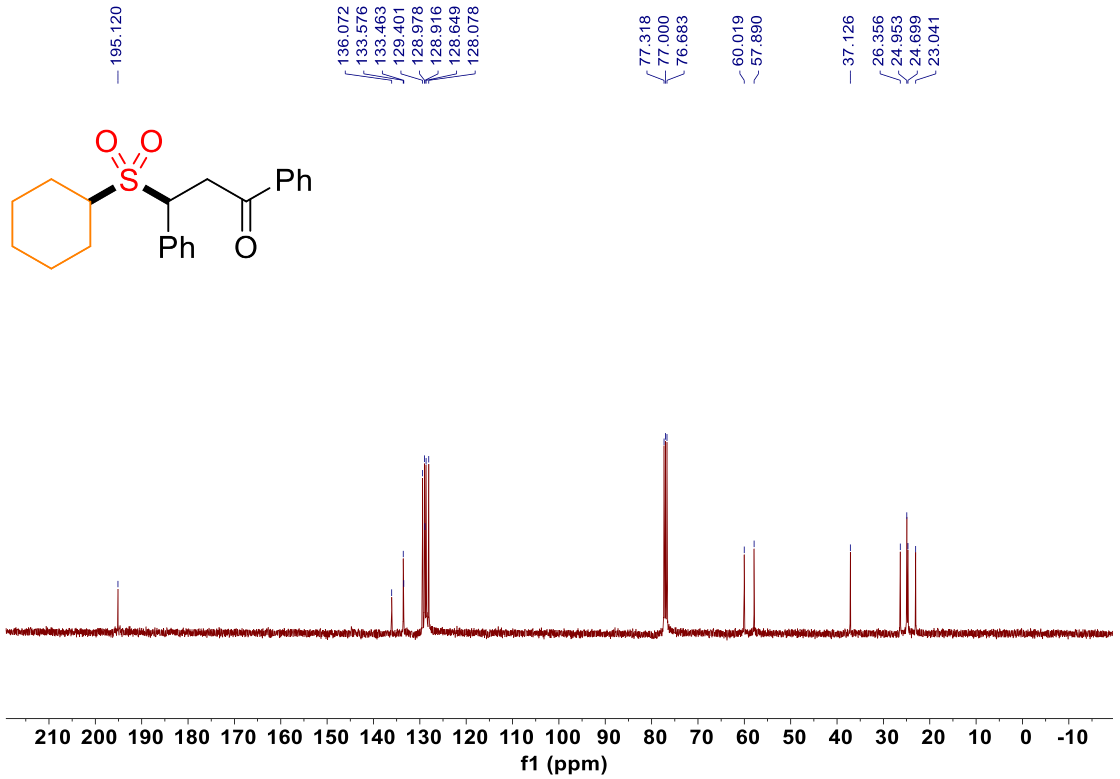

195.120
136.072
133.576
133.463
129.401
128.978
128.916
128.649
128.078
77.318
77.000
76.683
60.019
57.890
37.126
26.356
24.953
24.699
23.041
O
O
S
Ph
Ph
O
210 200 190 180 170 160 150 140 130 120 110 100 90 80 70 60 50 40 30 20 10 0 -10
f1 (ppm)

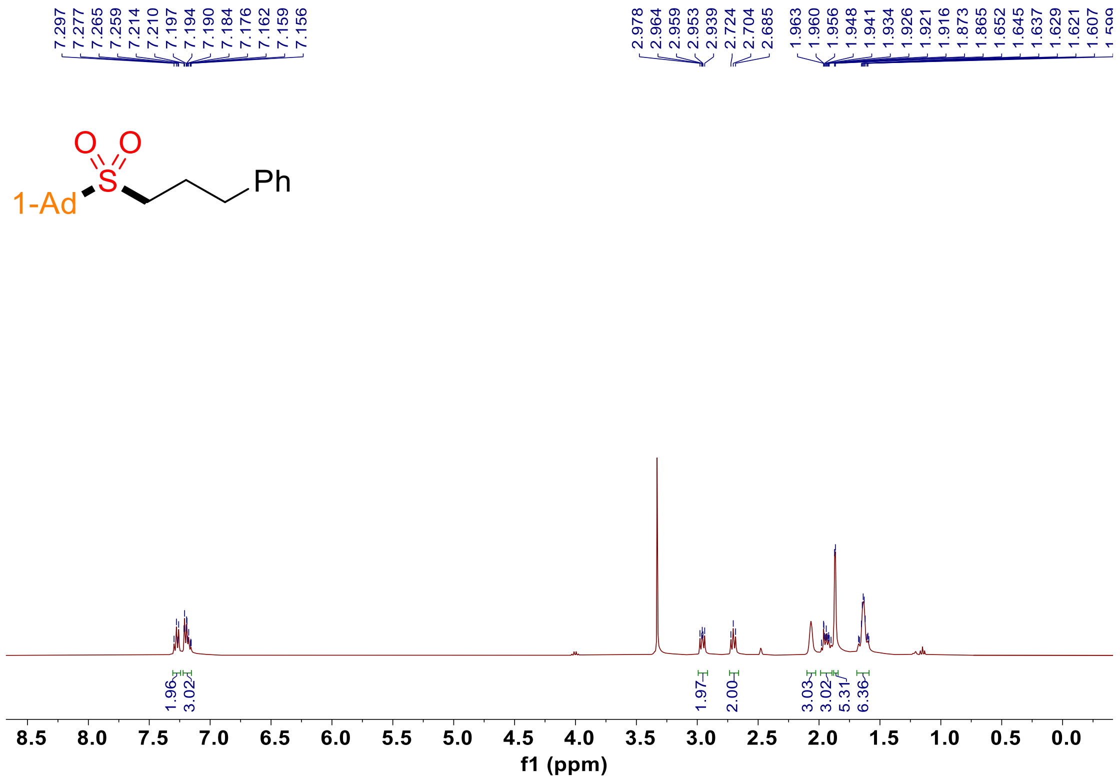
7.297
7.277
7.265
7.259
7.214
7.210
7.197
7.194
7.190
7.184
7.176
7.162
7.159
7.156
2.978
2.964
2.959
2.953
2.939
2.724
2.704
2.685
1.963
1.960
1.956
1.948
1.941
1.934
1.926
1.921
1.916
1.873
1.865
1.652
1.645
1.637
1.629
1.621
1.607
O
O
S
1-Ad
Ph
1.96
3.02
1.97
2.00
3.03
3.02
5.31
6.36
8.5 8.0 7.5 7.0 6.5 6.0 5.5 5.0 4.5 4.0 3.5 3.0 2.5 2.0 1.5 1.0 0.5 0.0
f1 (ppm)

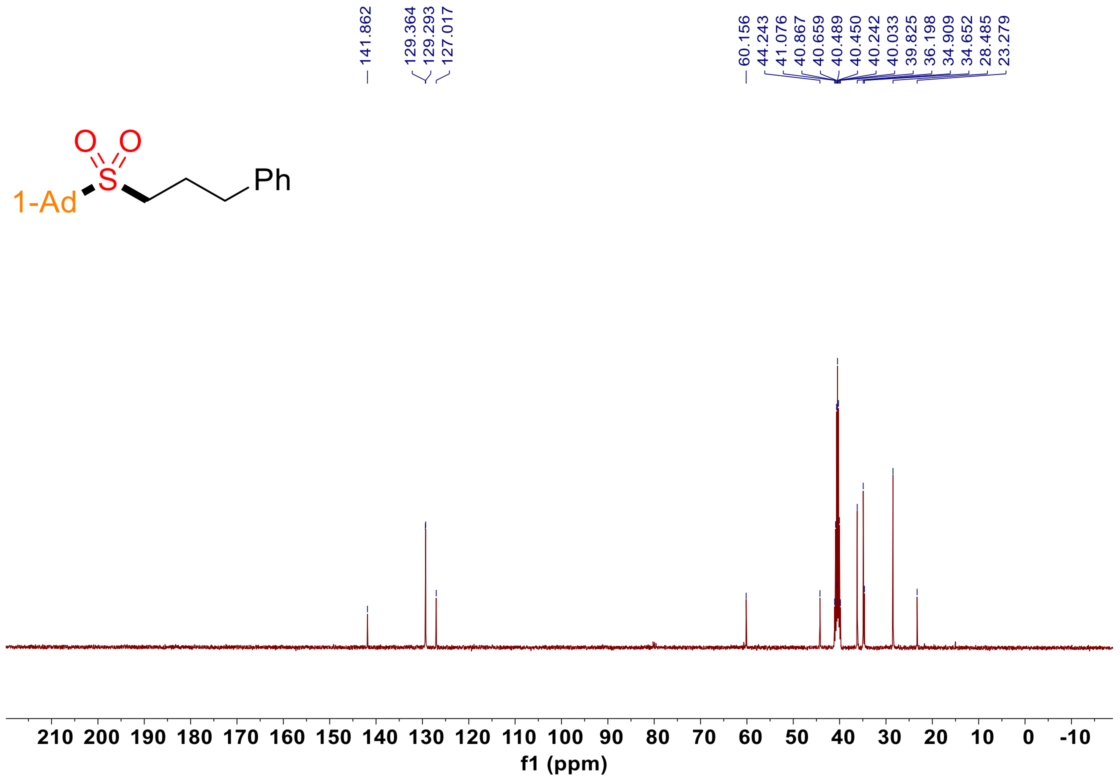
141.862
129.364
129.293
127.017
60.156
44.243
41.076
40.867
40.659
40.489
40.450
40.242
40.033
39.825
36.198
34.909
34.652
28.485
23.279
O
O
S
1-Ad
Ph
210 200 190 180 170 160 150 140 130 120 110 100 90 80 70 60 50 40 30 20 10 0 -10
f1 (ppm)

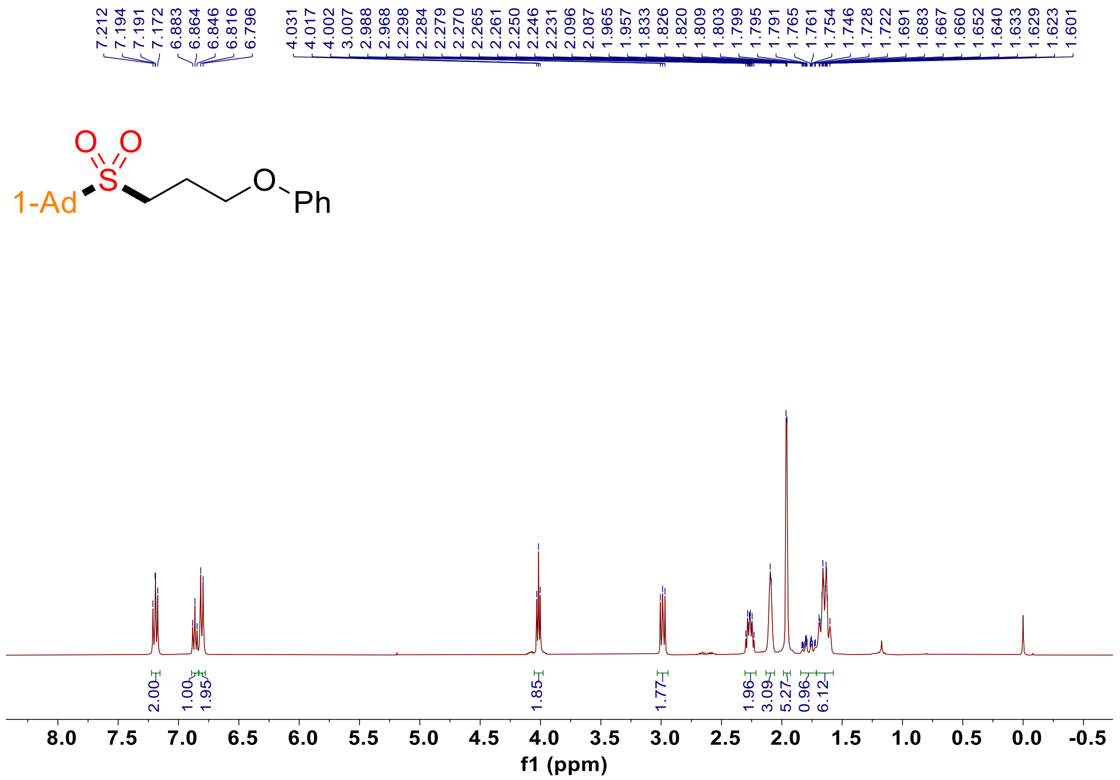
7.212
7.194
7.191
7.172
6.883
6.864
6.846
6.816
6.796
4.031
4.017
4.002
3.007
2.988
2.968
2.298
2.284
2.279
2.270
2.265
2.261
2.250
2.246
2.231
2.096
2.087
1.965
1.957
1.833
1.826
1.820
1.809
1.803
1.799
1.795
1.791
1.765
1.761
1.754
1.746
1.728
1.722
1.691
1.683
1.667
1.660
1.652
1.640
1.633
1.629
1.623
1.601
O O
S
1-Ad
O
Ph
2.00
1.00
1.95
1.85
1.77
1.96
3.09
5.27
0.96
6.12
8.0 7.5 7.0 6.5 6.0 5.5 5.0 4.5 4.0 3.5 3.0 2.5 2.0 1.5 1.0 0.5 0.0 -0.5
f1 (ppm)

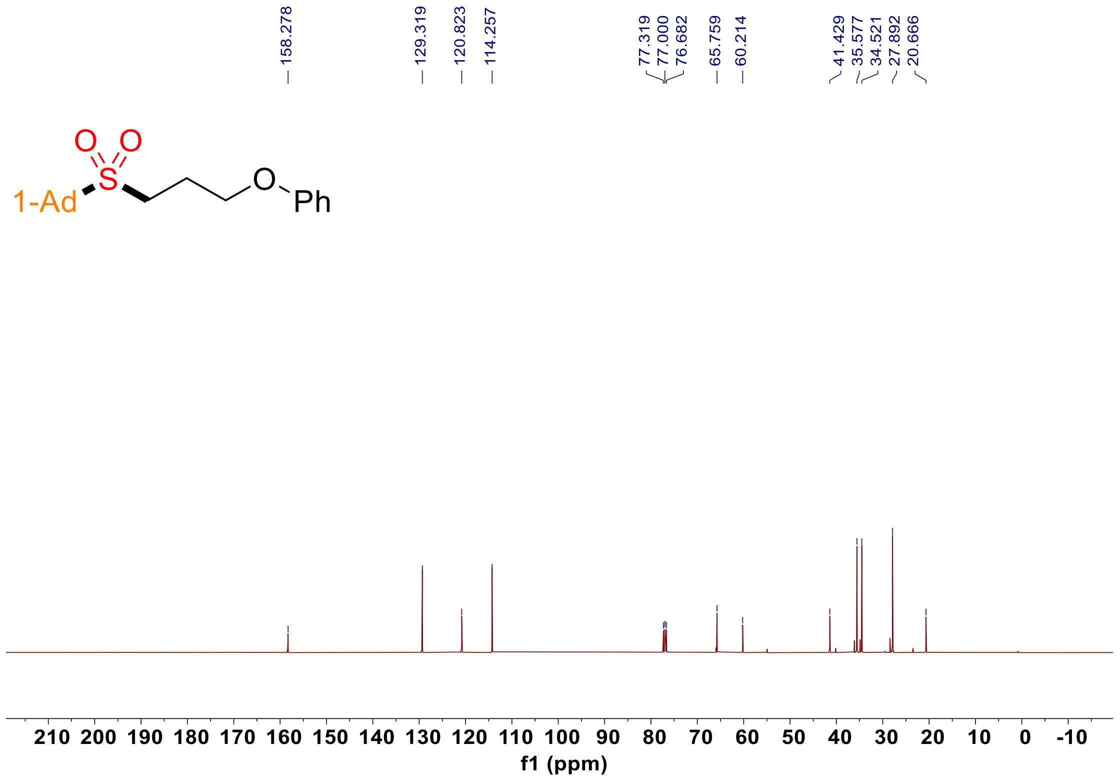
158.278
129.319
120.823
114.257
77.319
77.000
76.682
65.759
60.214
41.429
35.577
34.521
27.892
20.666
O O
S
1-Ad
O
Ph
210 200 190 180 170 160 150 140 130 120 110 100 90 80 70 60 50 40 30 20 10 0 -10
f1 (ppm)

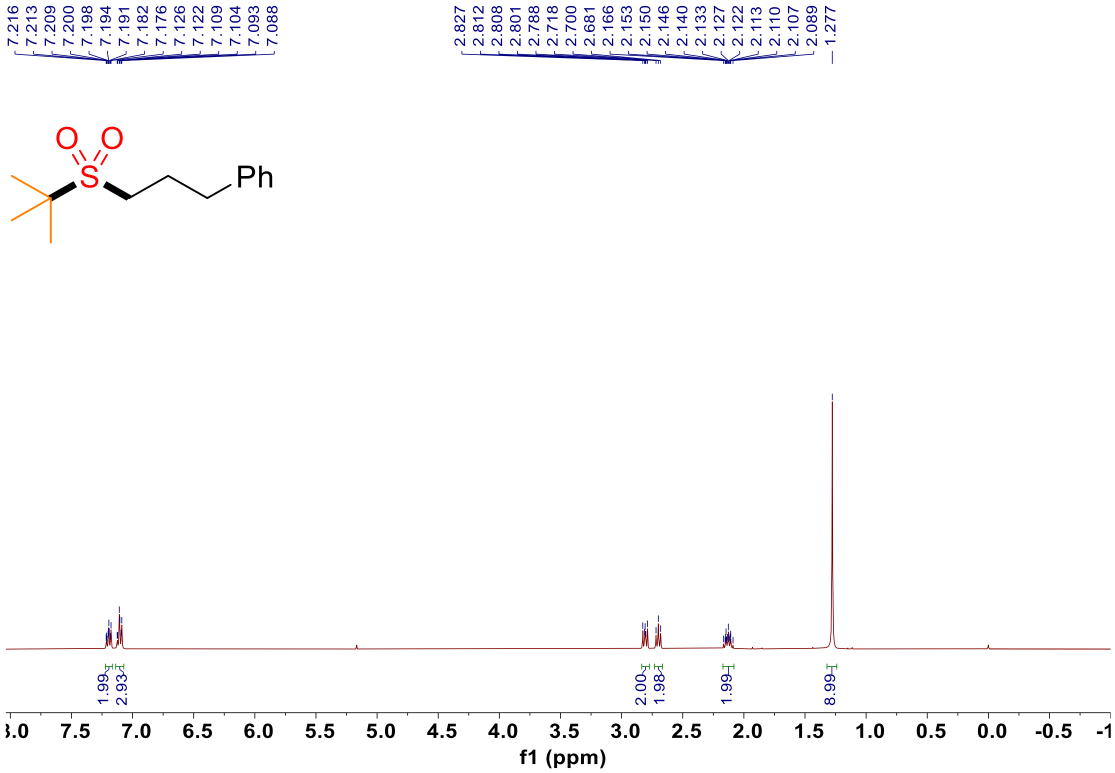

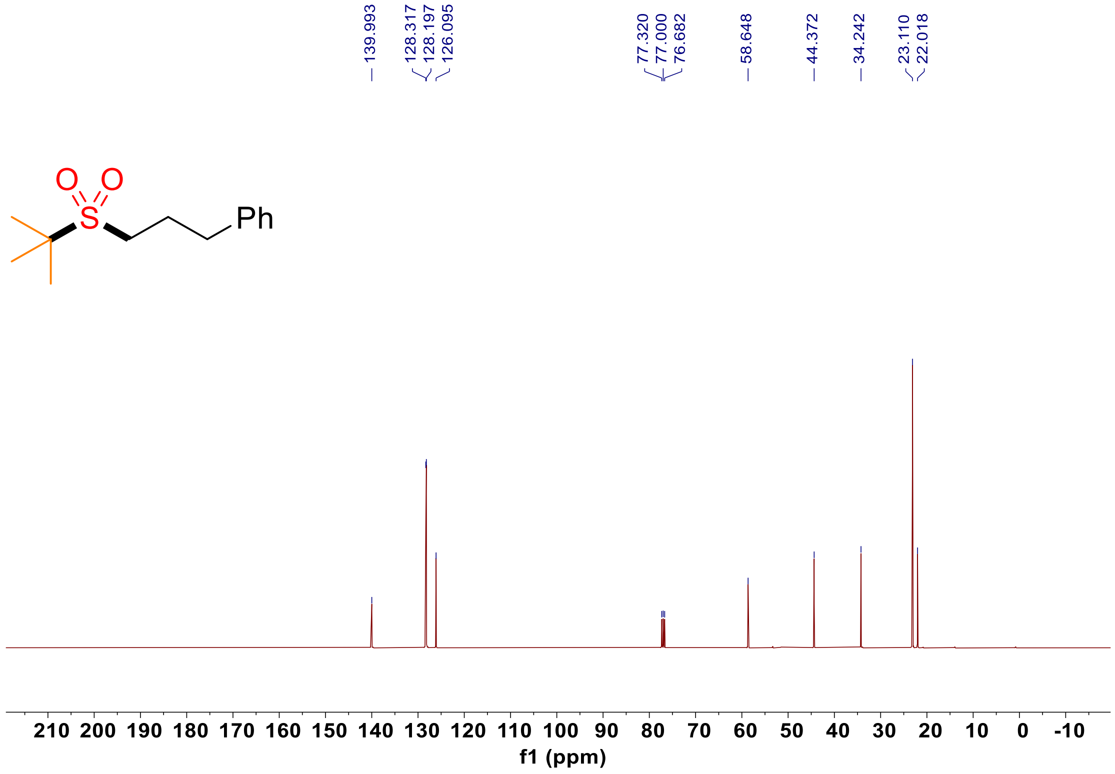

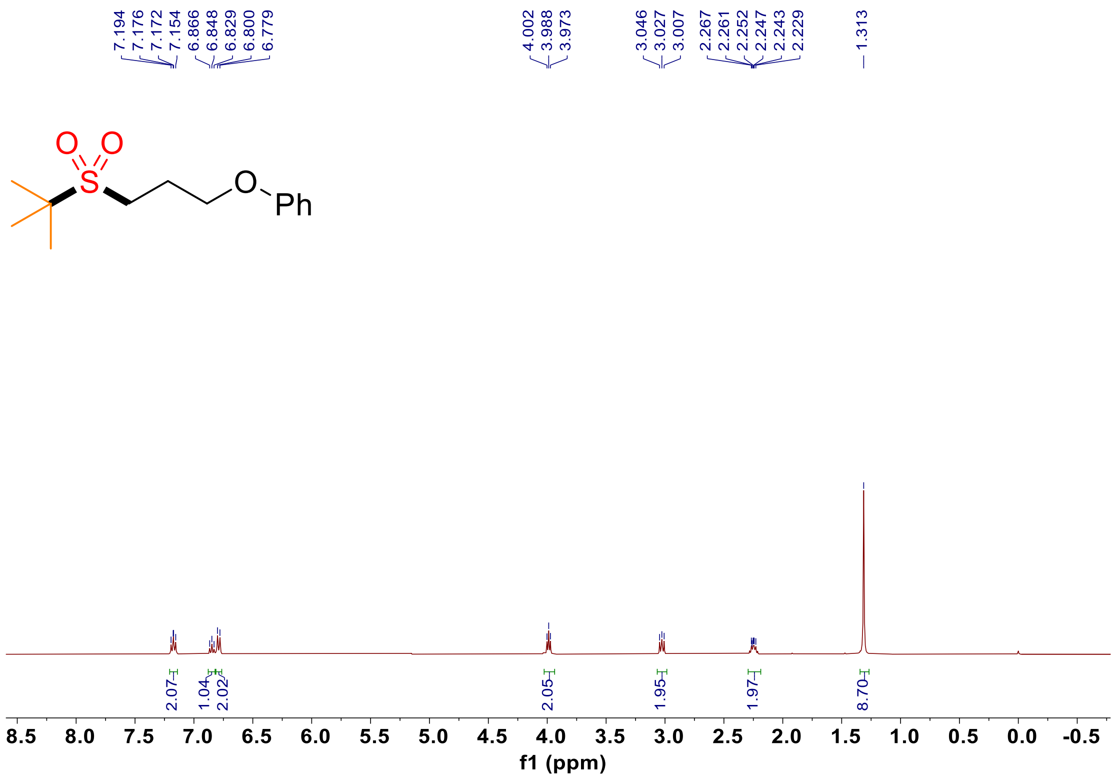
7.194
7.176
7.172
7.154
6.866
6.848
6.829
6.800
6.779
4.002
3.988
3.973
3.046
3.027
3.007
2.267
2.261
2.252
2.247
2.243
2.229
1.313
O
O
S
O
Ph
2.07
1.04
2.02
2.05
1.95
1.97
8.70
8.5 8.0 7.5 7.0 6.5 6.0 5.5 5.0 4.5 4.0 3.5 3.0 2.5 2.0 1.5 1.0 0.5 0.0 -0.5
f1 (ppm)

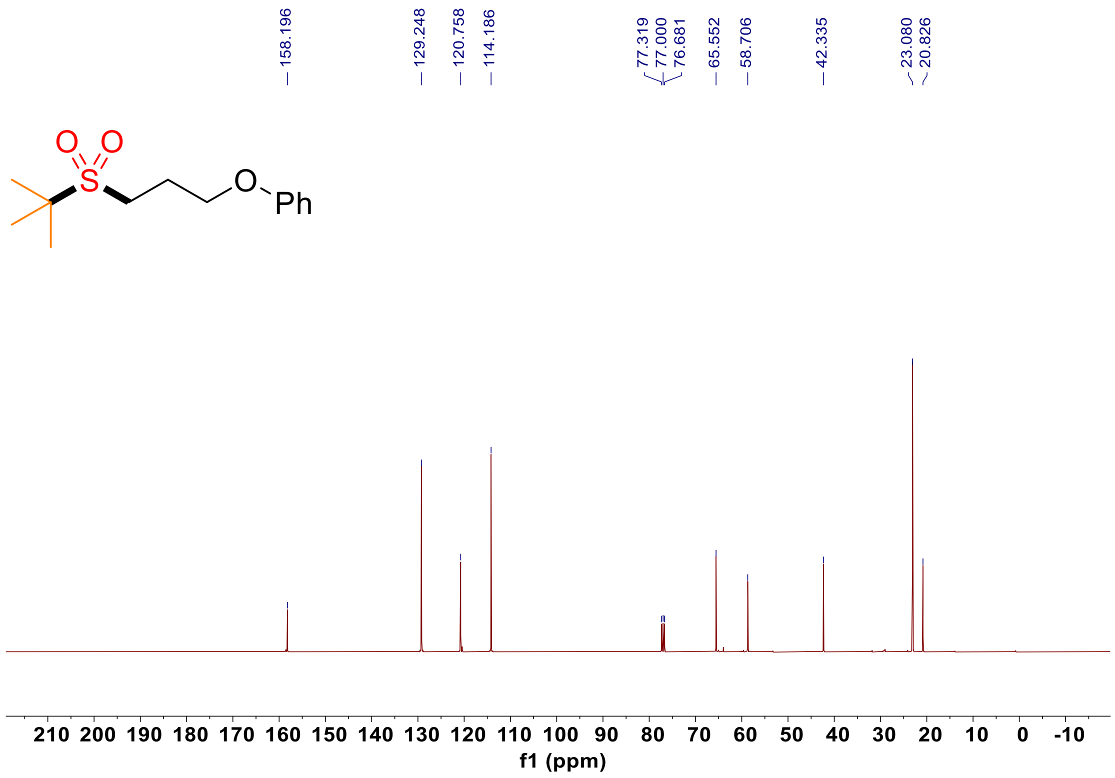
158.196
129.248
120.758
114.186
77.319
77.000
76.681
65.552
58.706
42.335
23.080
20.826
O
O
S
O
Ph
210 200 190 180 170 160 150 140 130 120 110 100 90 80 70 60 50 40 30 20 10 0 -10
f1 (ppm)

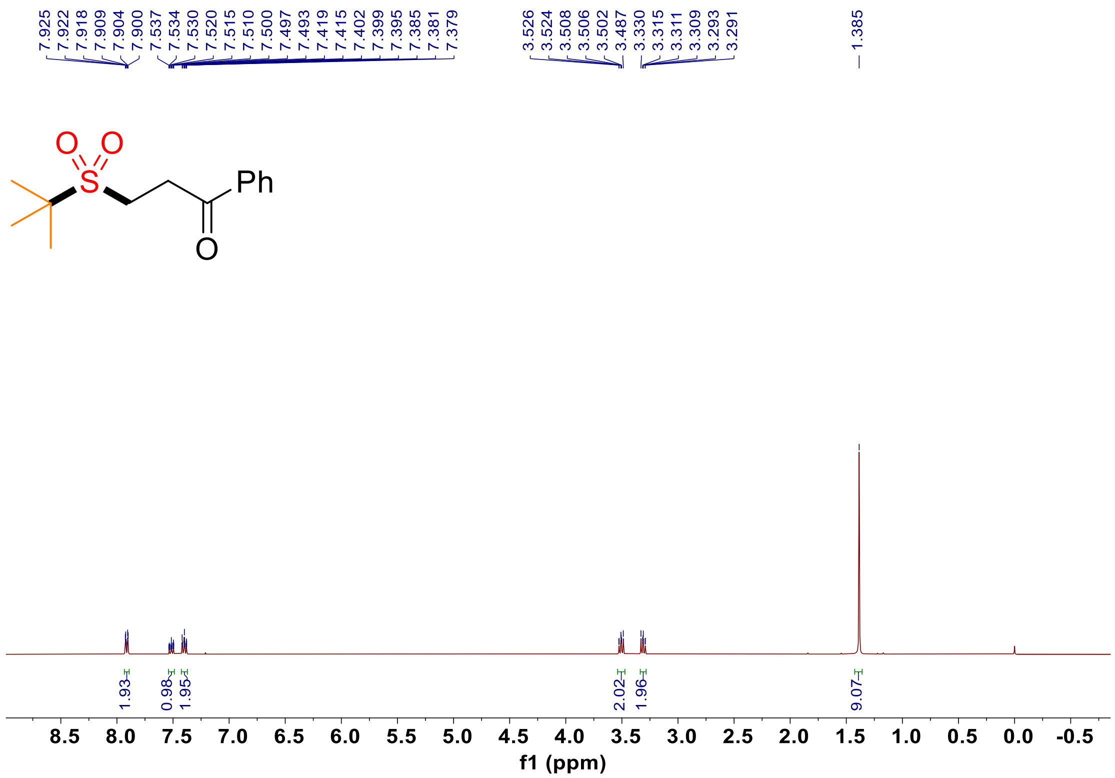
7.925
7.922
7.918
7.909
7.904
7.900
7.537
7.534
7.530
7.520
7.515
7.510
7.500
7.497
7.493
7.419
7.415
7.402
7.399
7.395
7.385
7.381
7.379
3.526
3.524
3.508
3.506
3.502
3.487
3.330
3.315
3.311
3.309
3.293
3.291
1.385
O
O
S
Ph
O
1.93
0.98
1.95
2.02
1.96
9.07
8.5 8.0 7.5 7.0 6.5 6.0 5.5 5.0 4.5 4.0 3.5 3.0 2.5 2.0 1.5 1.0 0.5 0.0 -0.5
f1 (ppm)

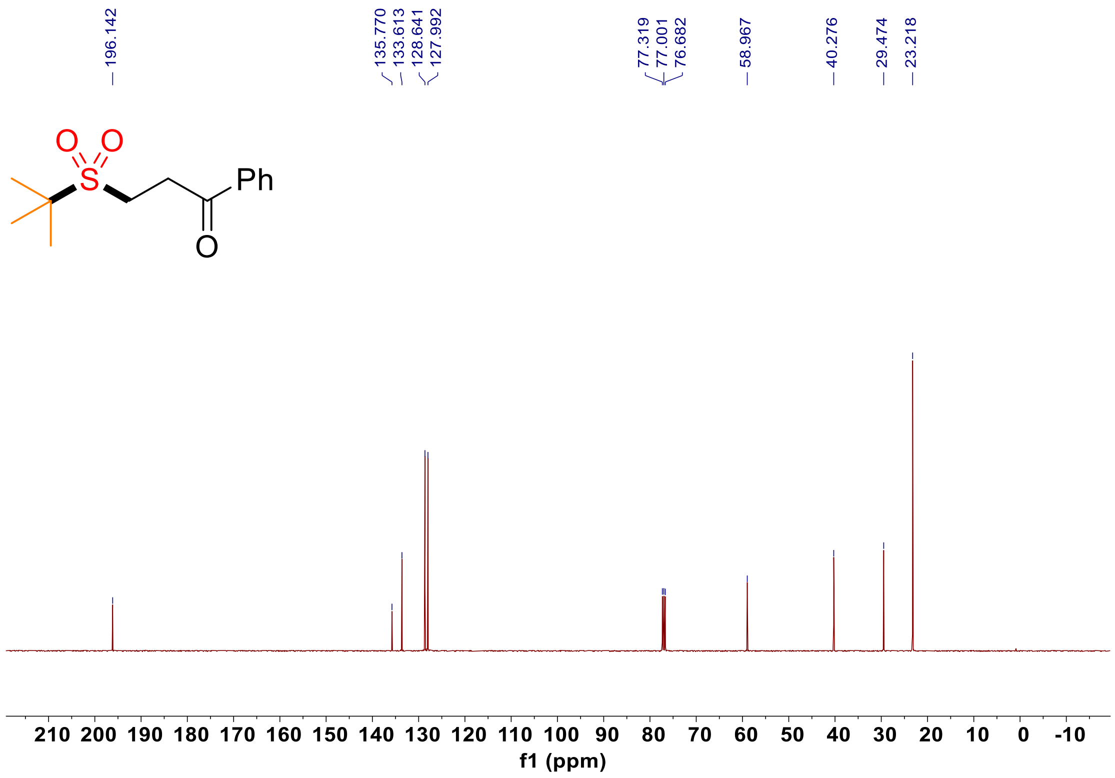
196.142
135.770
133.613
128.641
127.992
77.319
77.001
76.682
58.967
40.276
29.474
23.218
O
O
S
Ph
O
210 200 190 180 170 160 150 140 130 120 110 100 90 80 70 60 50 40 30 20 10 0 -10
f1 (ppm)

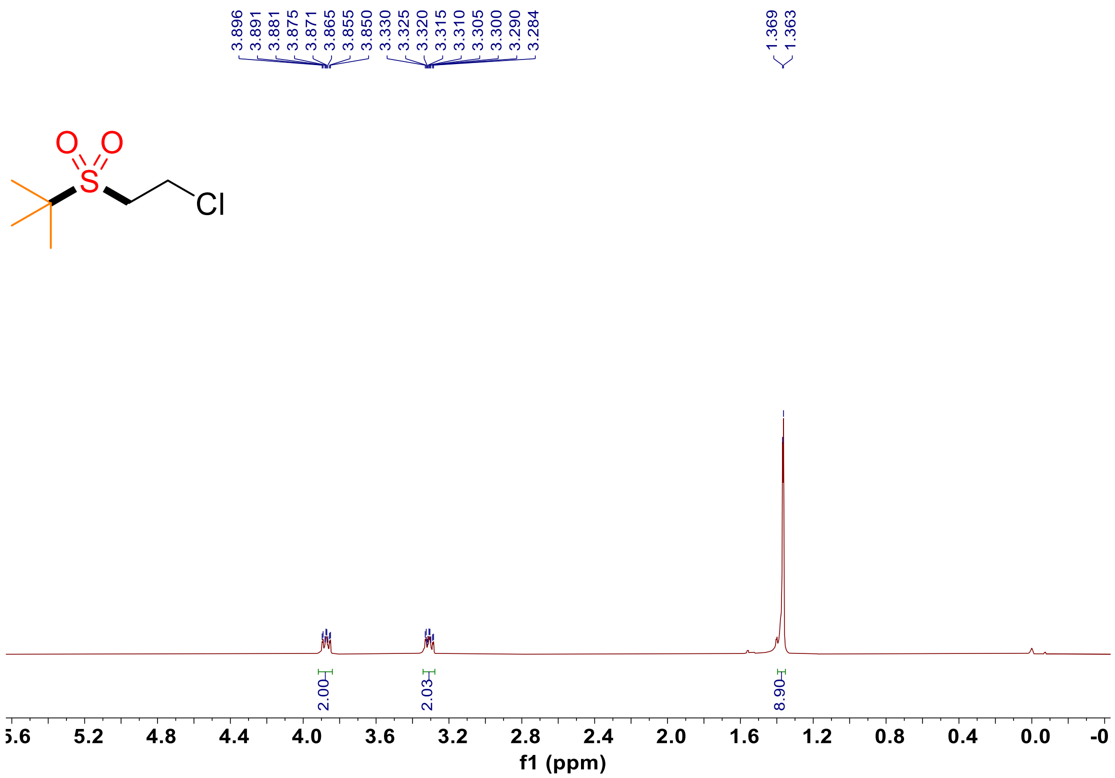
3.896
3.891
3.881
3.875
3.871
3.865
3.855
3.850
3.330
3.325
3.320
3.315
3.310
3.305
3.300
3.290
3.284
1.369
1.363
O
O
S
Cl
2.00
2.03
8.90
5.6
5.2
4.8
4.4
4.0
3.6
3.2
2.8
2.4
2.0
1.6
1.2
0.8
0.4
0.0
-0
f1 (ppm)

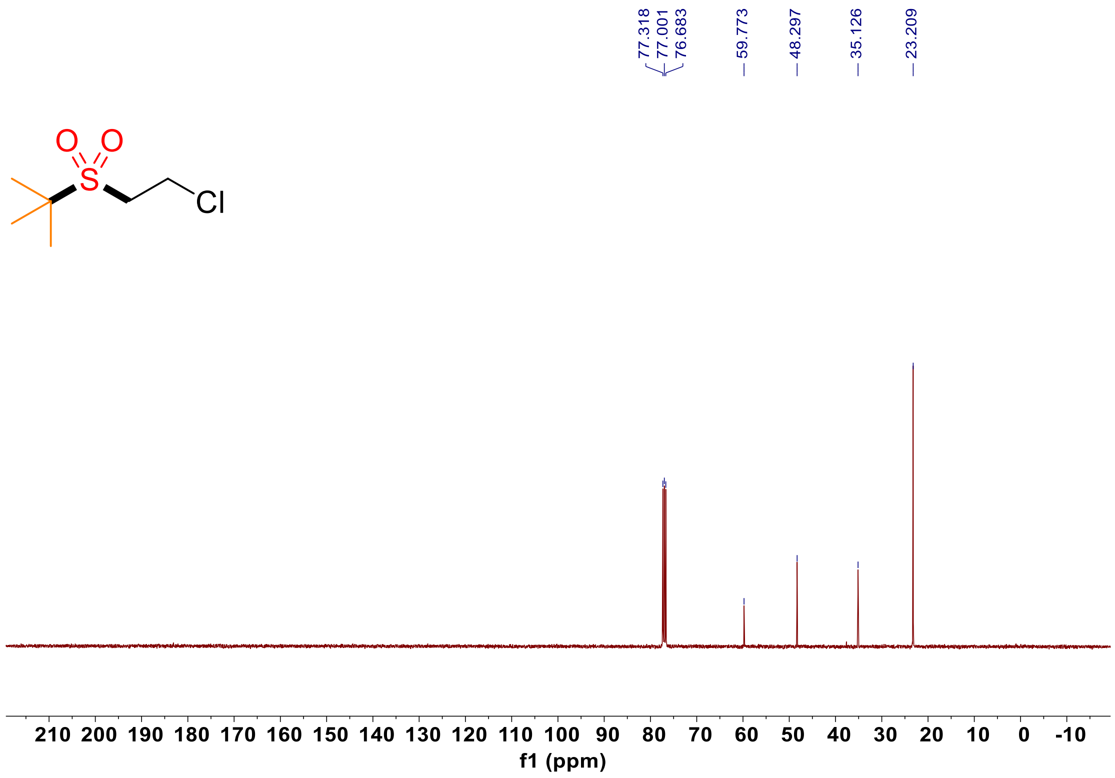
77.318
77.001
76.683
59.773
48.297
35.126
23.209
O
O
S
Cl
210 200 190 180 170 160 150 140 130 120 110 100 90 80 70 60 50 40 30 20 10 0 -10
f1 (ppm)

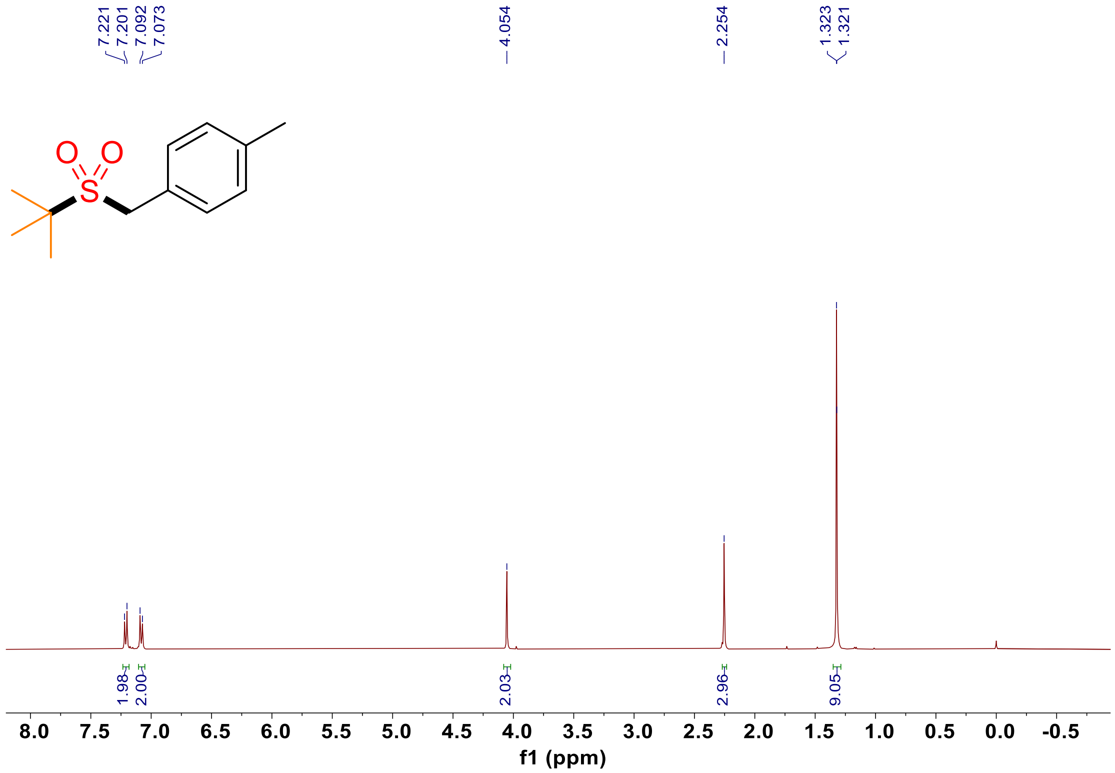
7.221
7.201
7.092
7.073
4.054
2.254
1.323
1.321
1.98
2.00
2.03
2.96
9.05
8.0 7.5 7.0 6.5 6.0 5.5 5.0 4.5 4.0 3.5 3.0 2.5 2.0 1.5 1.0 0.5 0.0 -0.5
f1 (ppm)

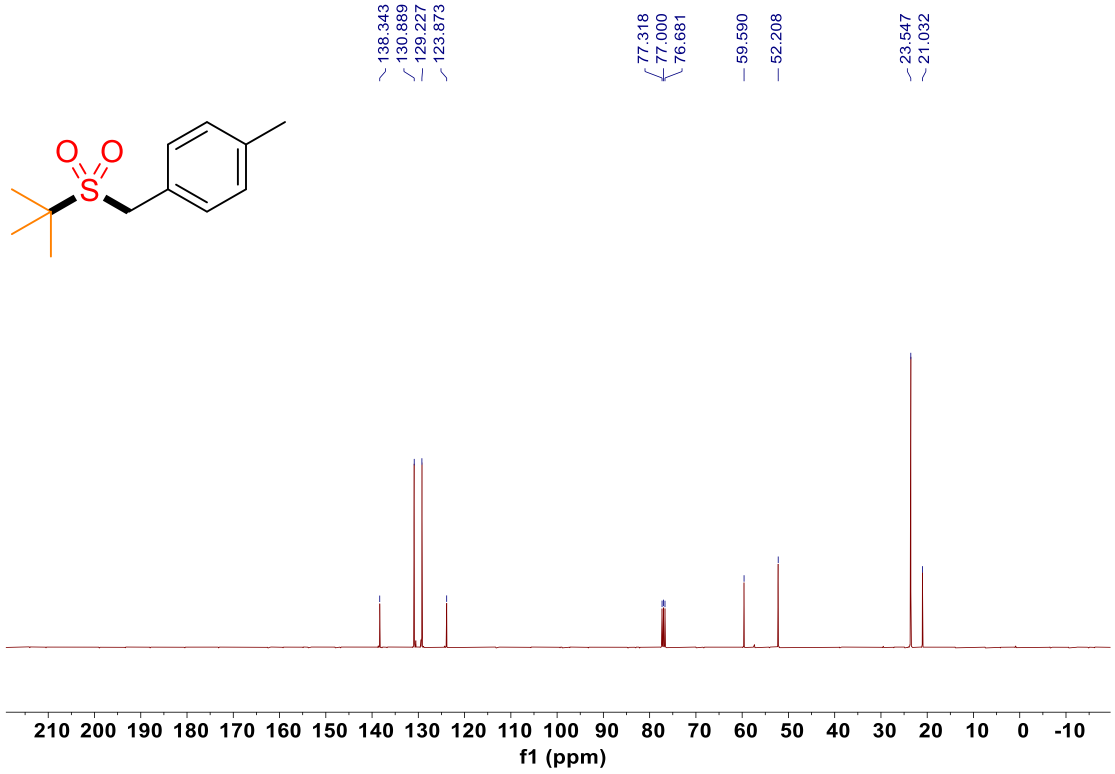
138.343
130.889
129.227
123.873
77.318
77.000
76.681
59.590
52.208
23.547
21.032
210 200 190 180 170 160 150 140 130 120 110 100 90 80 70 60 50 40 30 20 10 0 -10
f1 (ppm)

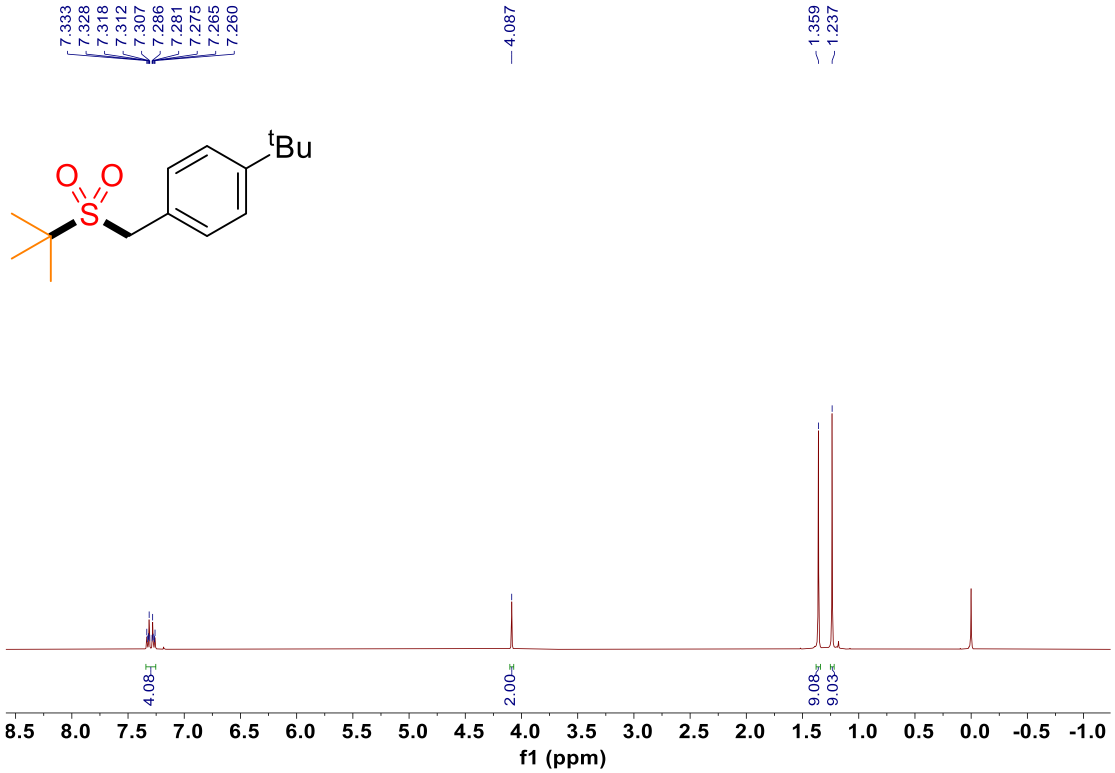
7.333
7.328
7.318
7.312
7.307
7.286
7.281
7.275
7.265
7.260
4.087
1.359
1.237
tBu
O
O
S
4.08
2.00
9.08
9.03
8.5 8.0 7.5 7.0 6.5 6.0 5.5 5.0 4.5 4.0 3.5 3.0 2.5 2.0 1.5 1.0 0.5 0.0 -0.5 -1.0
f1 (ppm)

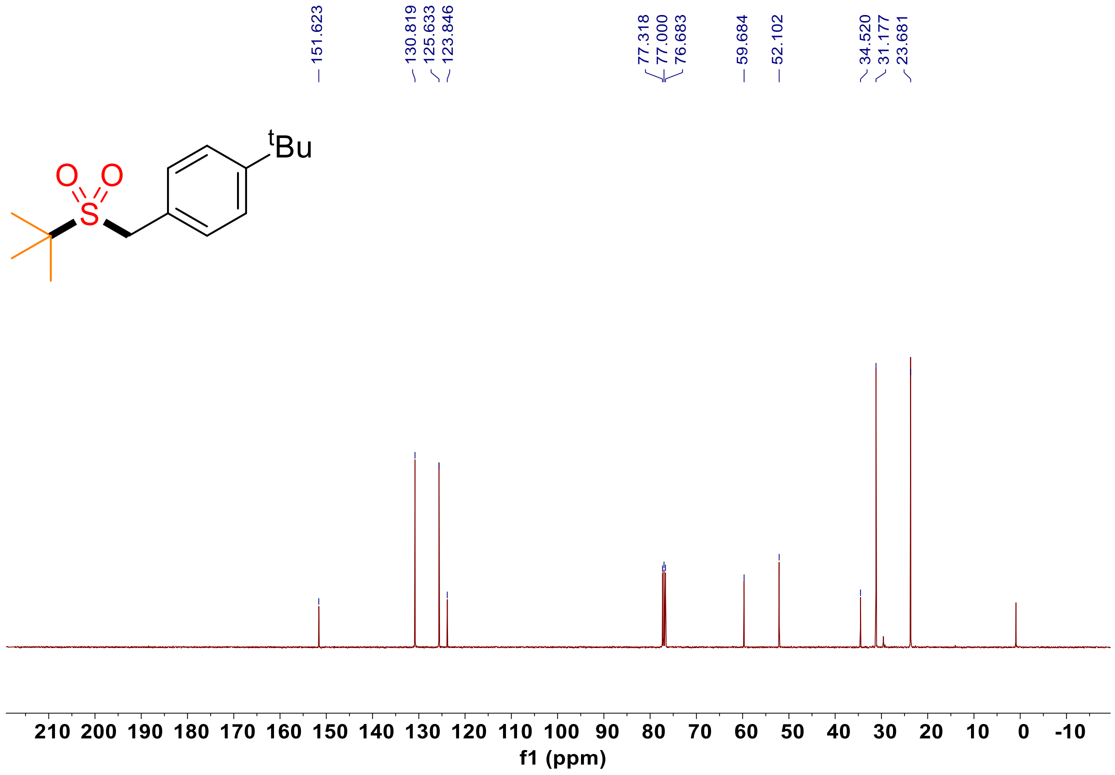
151.623
130.819
125.633
123.846
77.318
77.000
76.683
59.684
52.102
34.520
31.177
23.681
tBu
O
O
S
210 200 190 180 170 160 150 140 130 120 110 100 90 80 70 60 50 40 30 20 10 0 -10
f1 (ppm)

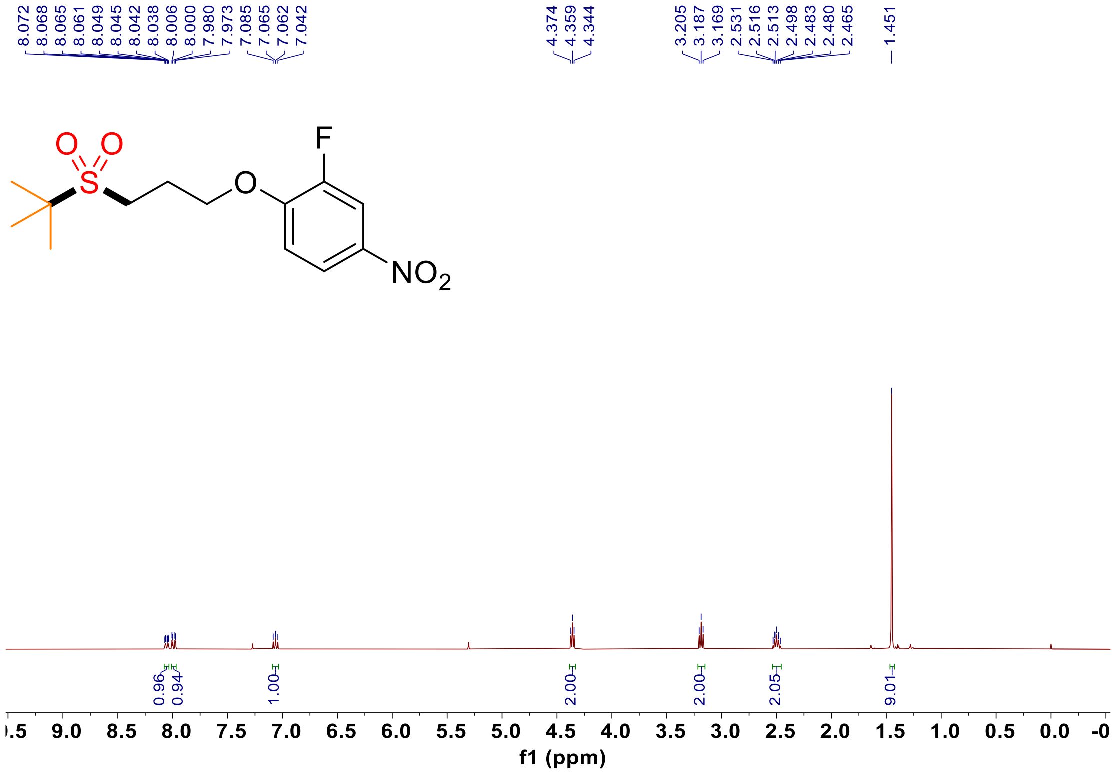
8.072
8.068
8.065
8.061
8.049
8.045
8.042
8.038
8.006
8.000
7.980
7.973
7.085
7.065
7.062
7.042
4.374
4.359
4.344
3.205
3.187
3.169
2.531
2.516
2.513
2.498
2.483
2.480
2.465
1.451
O
O
S
O
F
NO2
0.96
0.94
1.00
2.00
2.00
2.05
9.01
9.0
8.5
8.0
7.5
7.0
6.5
6.0
5.5
5.0
4.5
4.0
3.5
3.0
2.5
2.0
1.5
1.0
0.5
0.0
f1 (ppm)

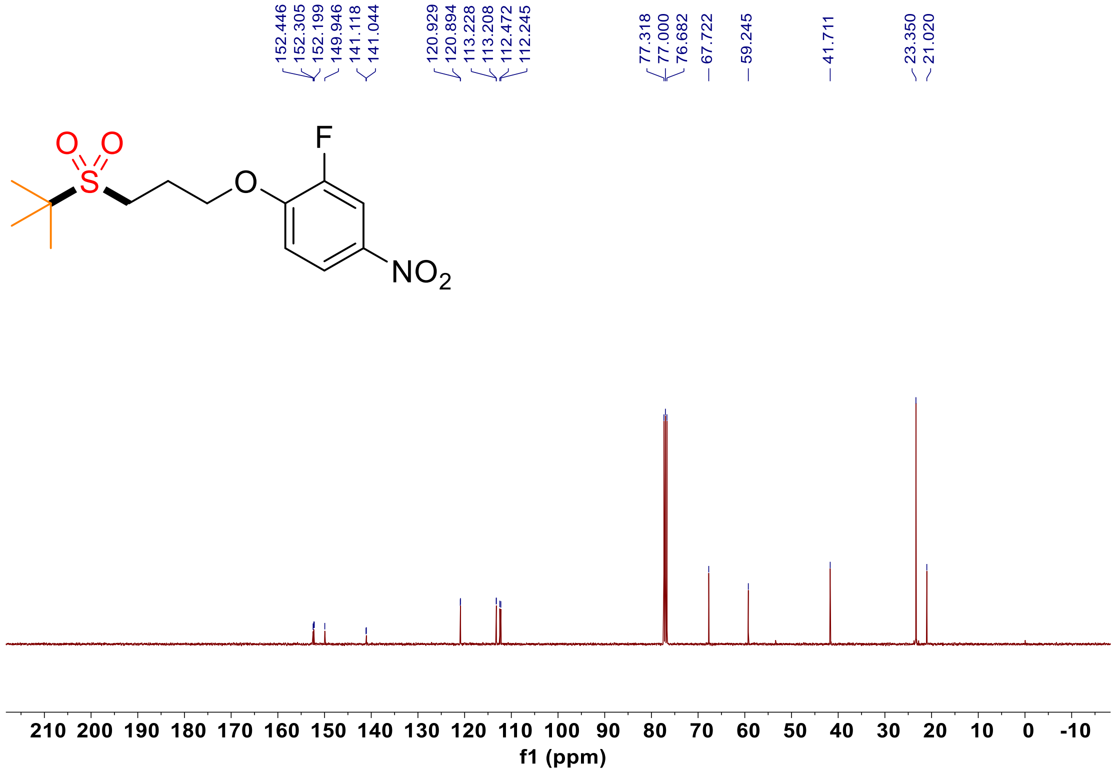
152.446
152.305
152.199
149.946
141.118
141.044
120.929
120.894
113.228
113.208
112.472
112.245
77.318
77.000
76.682
67.722
59.245
41.711
23.350
21.020
O
O
S
O
F
NO2
210
200
190
180
170
160
150
140
130
120
110
100
90
80
70
60
50
40
30
20
10
0
-10
f1 (ppm)

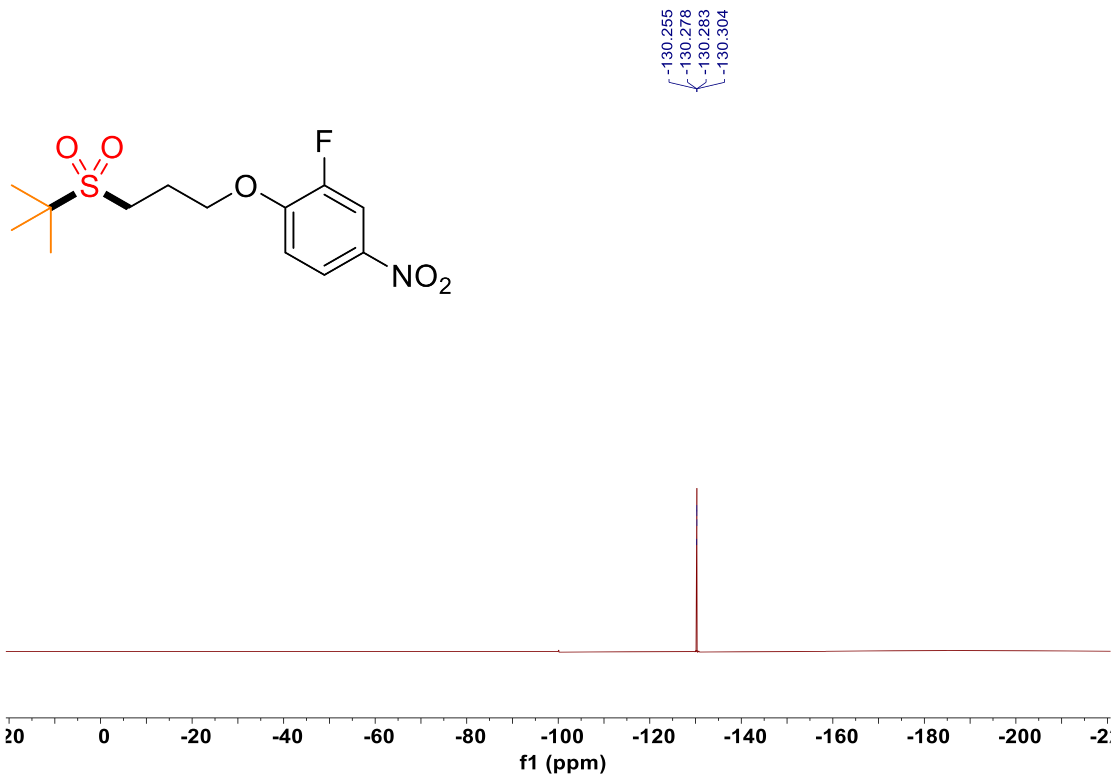

-130.255
-130.278
-130.283
-130.304
F
O
NO2
20
0
-20
-40
-60
-80
-100
-120
-140
-160
-180
-200
f1 (ppm)

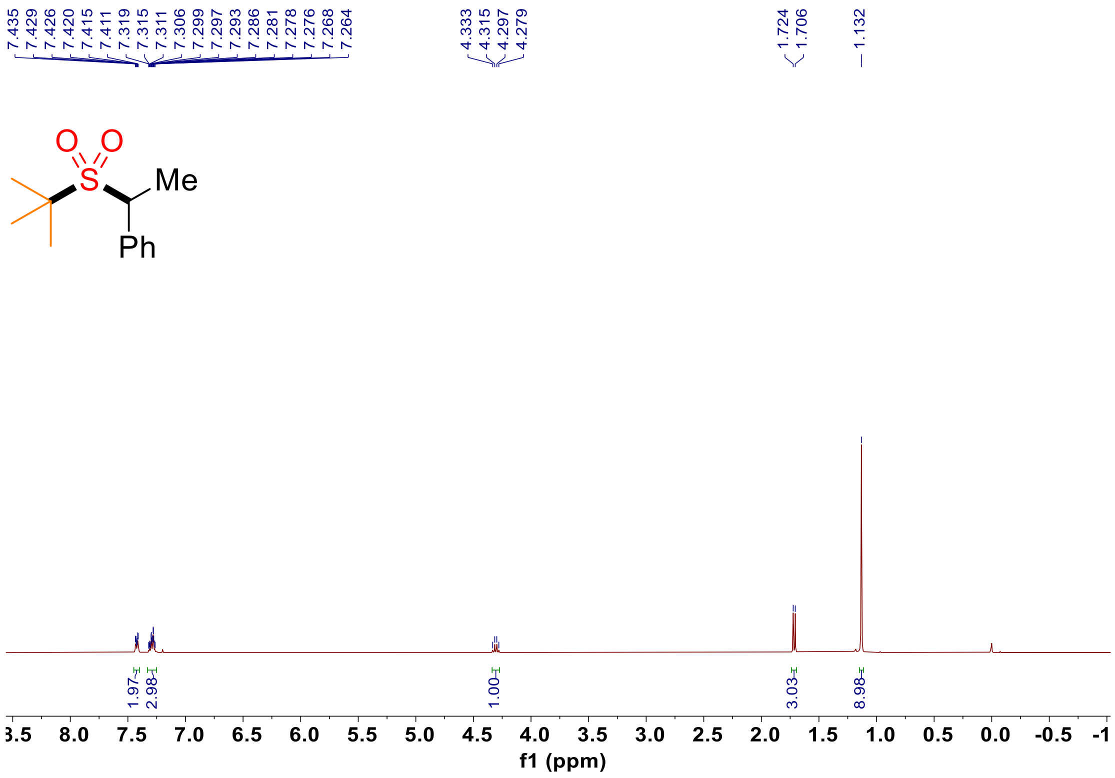

7.435
7.429
7.426
7.420
7.415
7.411
7.319
7.315
7.311
7.306
7.299
7.297
7.293
7.286
7.281
7.278
7.276
7.268
7.264
4.333
4.315
4.297
4.279
1.724
1.706
1.132
Me
Ph
1.97
2.98
1.00
3.03
8.98
8.0
7.5
7.0
6.5
6.0
5.5
5.0
4.5
4.0
3.5
3.0
2.5
2.0
1.5
1.0
0.5
0.0
-0.5
f1 (ppm)

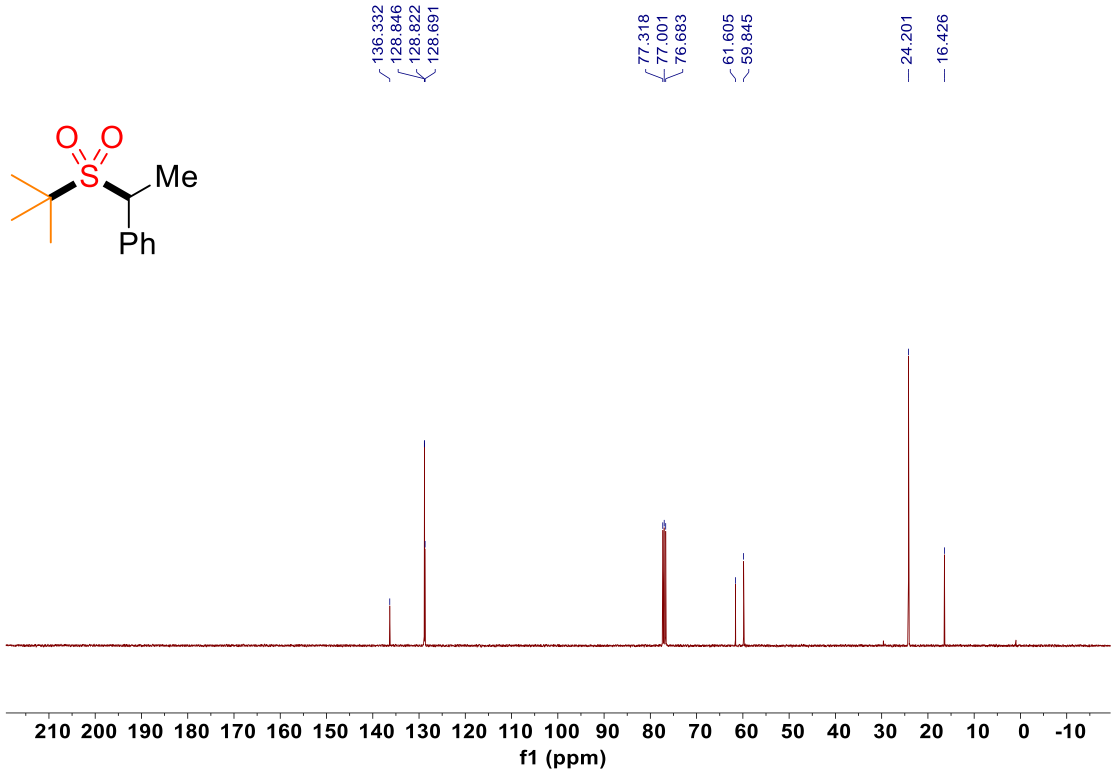
136.332
128.846
128.822
128.691
77.318
77.001
76.683
61.605
59.845
24.201
16.426
O
O
S
Me
Ph
210 200 190 180 170 160 150 140 130 120 110 100 90 80 70 60 50 40 30 20 10 0 -10
f1 (ppm)

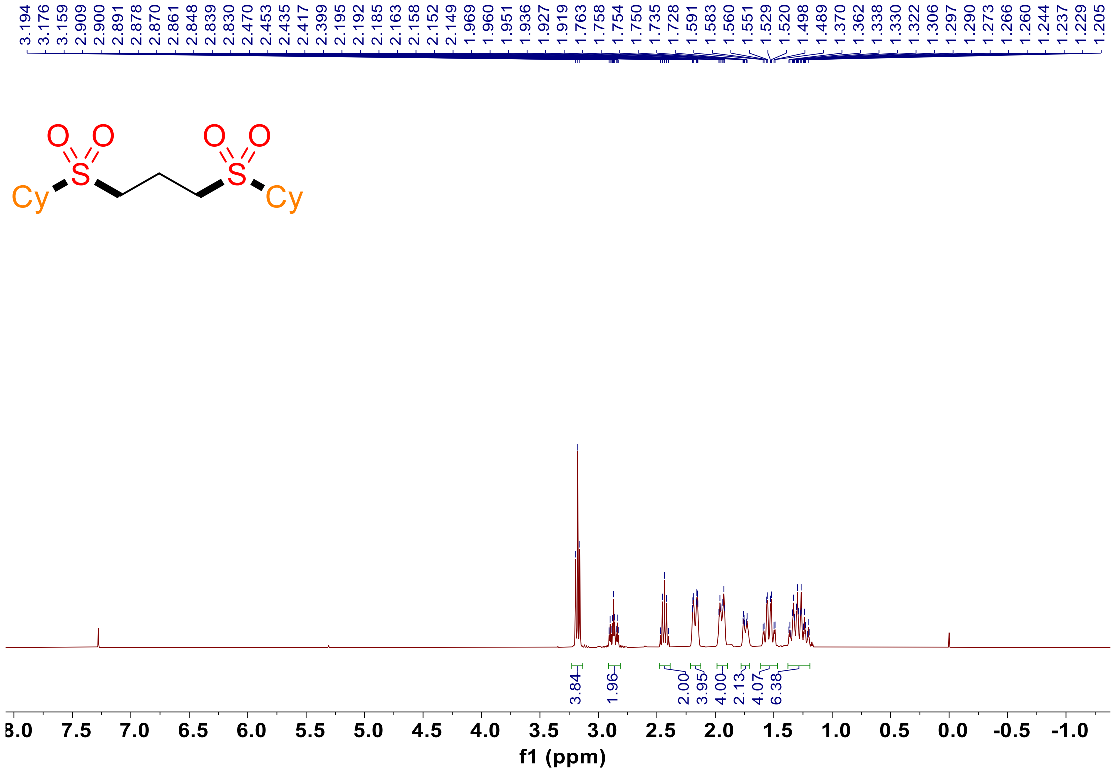
3.194
3.176
3.159
2.909
2.900
2.891
2.878
2.870
2.861
2.848
2.839
2.830
2.470
2.453
2.435
2.417
2.399
2.195
2.192
2.185
2.163
2.158
2.152
2.149
1.969
1.960
1.951
1.936
1.927
1.919
1.763
1.758
1.754
1.750
1.735
1.728
1.591
1.583
1.560
1.551
1.529
1.520
1.498
1.489
1.370
1.362
1.338
1.330
1.322
1.306
1.297
1.290
1.273
1.266
1.260
1.244
1.237
1.229
1.205
O
O
O
O
S
S
Cy
Cy
3.84
1.96
2.00
3.95
4.00
2.13
4.07
6.38
8.0 7.5 7.0 6.5 6.0 5.5 5.0 4.5 4.0 3.5 3.0 2.5 2.0 1.5 1.0 0.5 0.0 -0.5 -1.0
f1 (ppm)

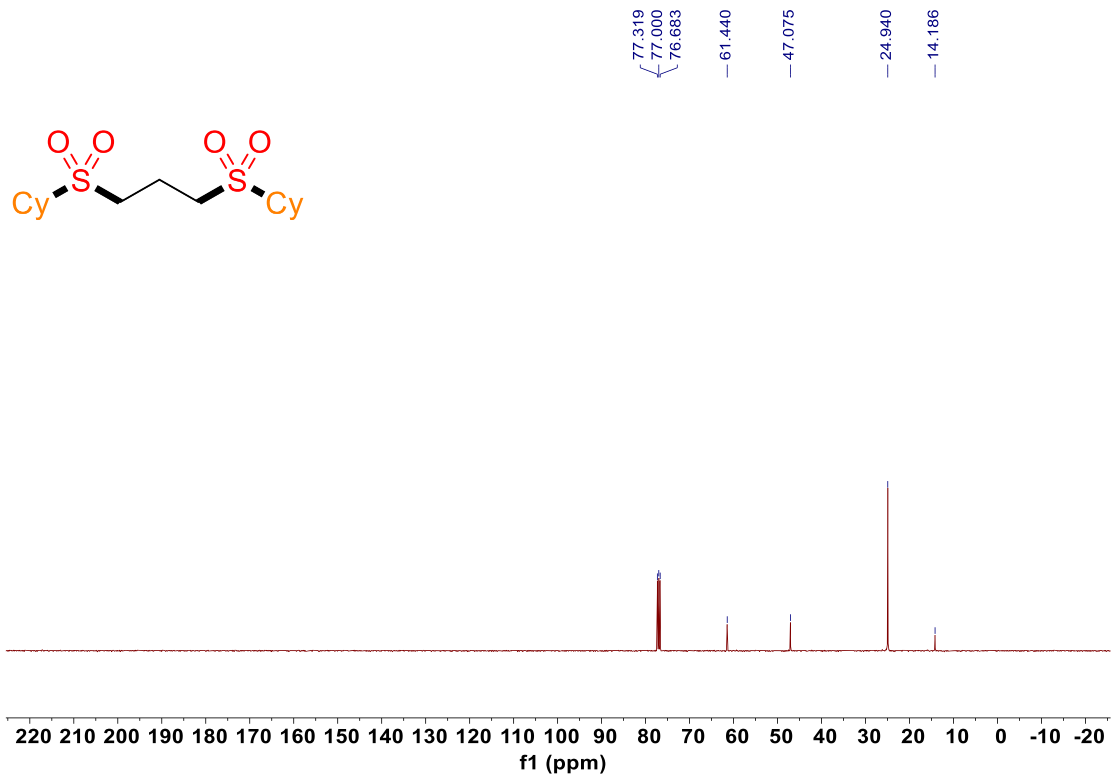
77.319
77.000
76.683
61.440
47.075
24.940
14.186
Cy
Cy
220 210 200 190 180 170 160 150 140 130 120 110 100 90 80 70 60 50 40 30 20 10 0 -10 -20
f1 (ppm)

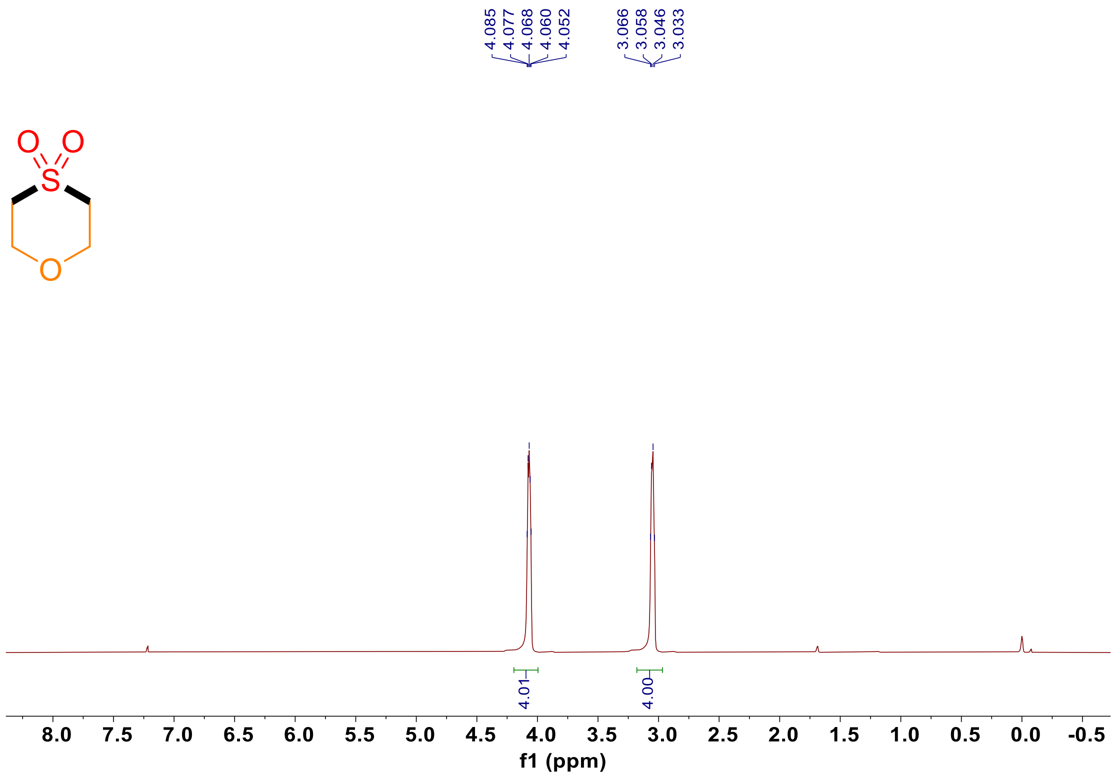
4.085
4.077
4.068
4.060
4.052
3.066
3.058
3.046
3.033
4.01
4.00
8.0 7.5 7.0 6.5 6.0 5.5 5.0 4.5 4.0 3.5 3.0 2.5 2.0 1.5 1.0 0.5 0.0 -0.5
f1 (ppm)

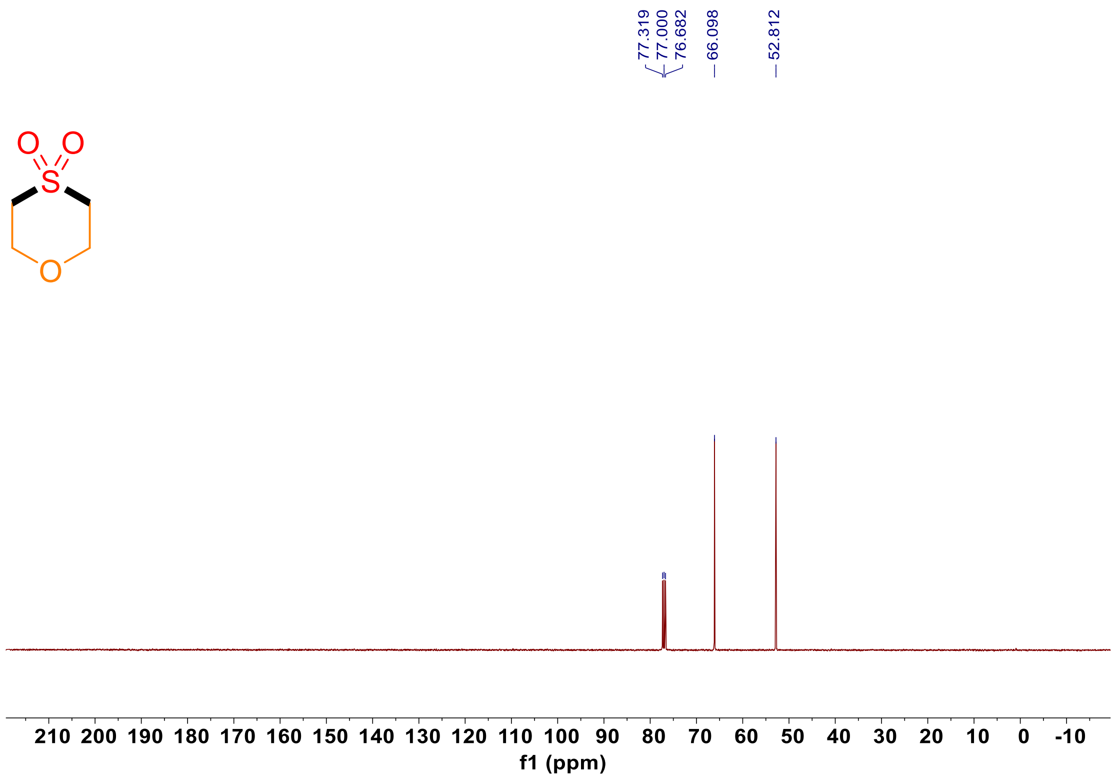
77.319
77.000
76.682
66.098
52.812
210 200 190 180 170 160 150 140 130 120 110 100 90 80 70 60 50 40 30 20 10 0 -10
f1 (ppm)

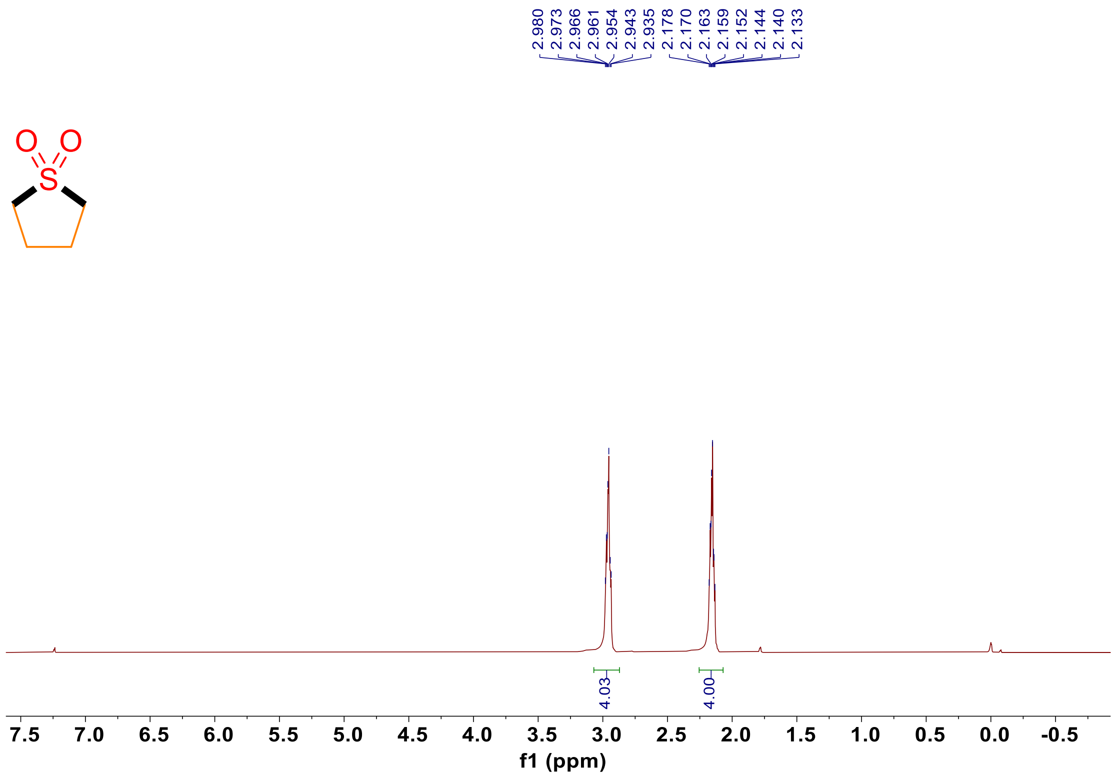
2.980
2.973
2.966
2.961
2.954
2.943
2.935
2.178
2.170
2.163
2.159
2.152
2.144
2.140
2.133
4.03
4.00
7.5 7.0 6.5 6.0 5.5 5.0 4.5 4.0 3.5 3.0 2.5 2.0 1.5 1.0 0.5 0.0 -0.5
f1 (ppm)

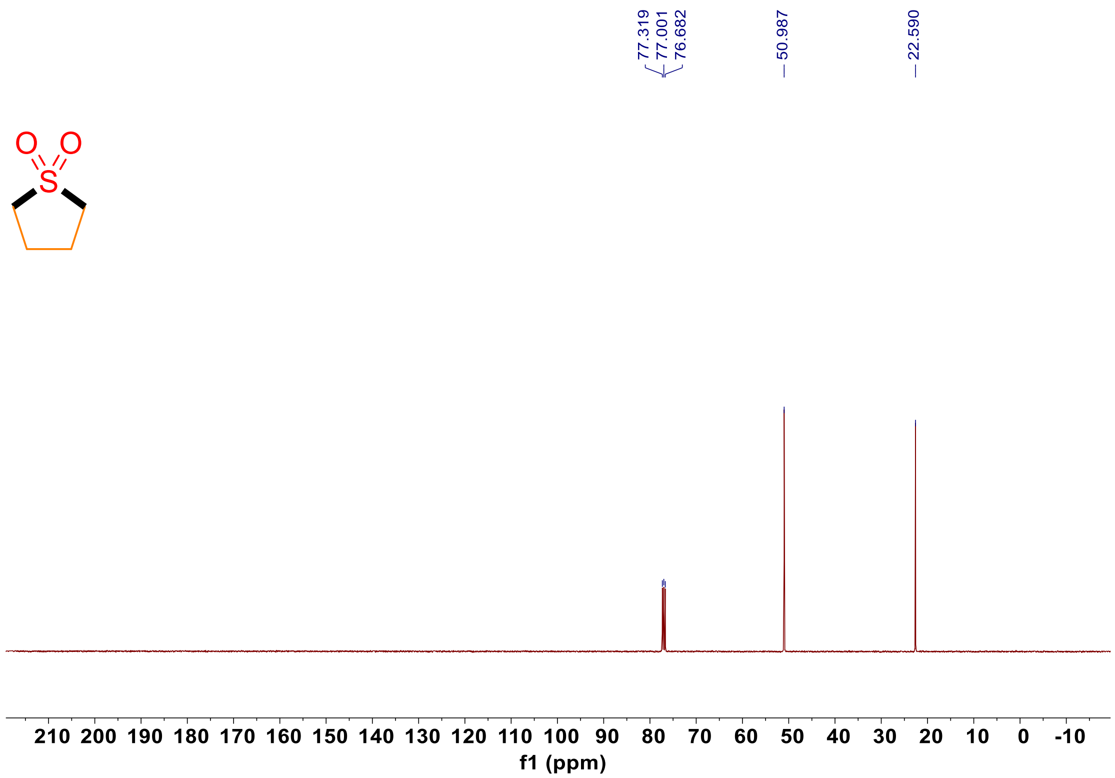
77.319
77.001
76.682
50.987
22.590
O
O
S
210 200 190 180 170 160 150 140 130 120 110 100 90 80 70 60 50 40 30 20 10 0 -10
f1 (ppm)

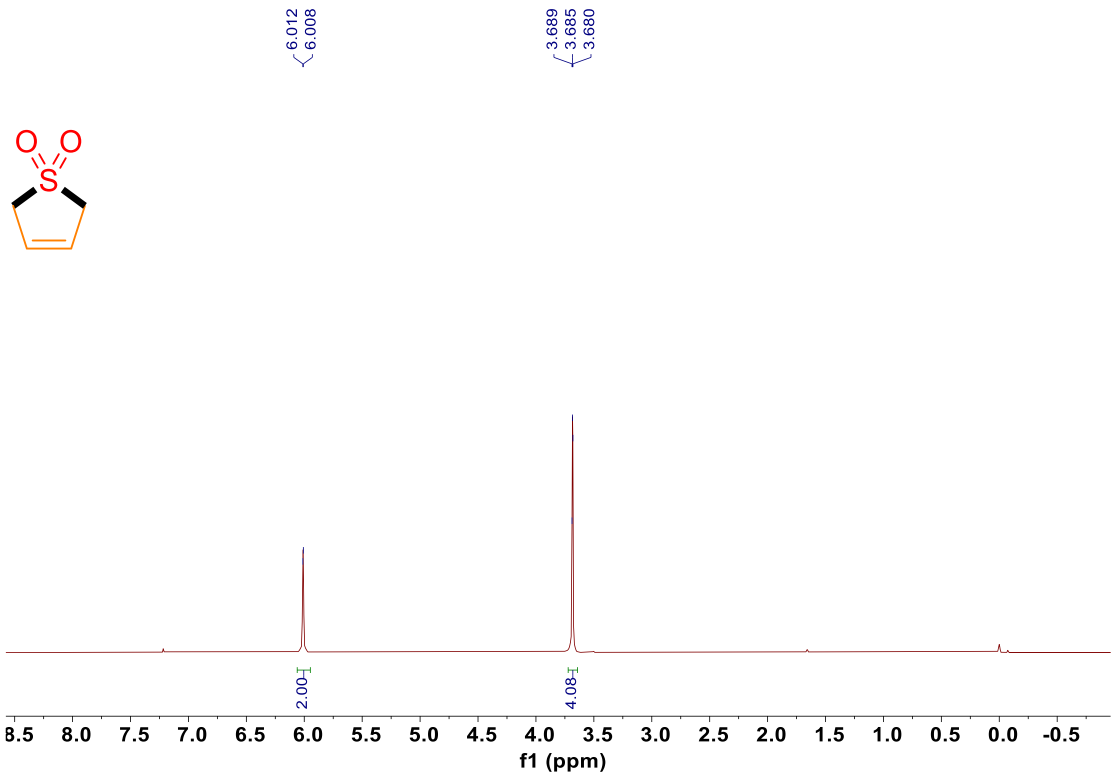
6.012
6.008
3.689
3.685
3.680
O
O
S
2.00
4.08
8.5 8.0 7.5 7.0 6.5 6.0 5.5 5.0 4.5 4.0 3.5 3.0 2.5 2.0 1.5 1.0 0.5 0.0 -0.5
f1 (ppm)

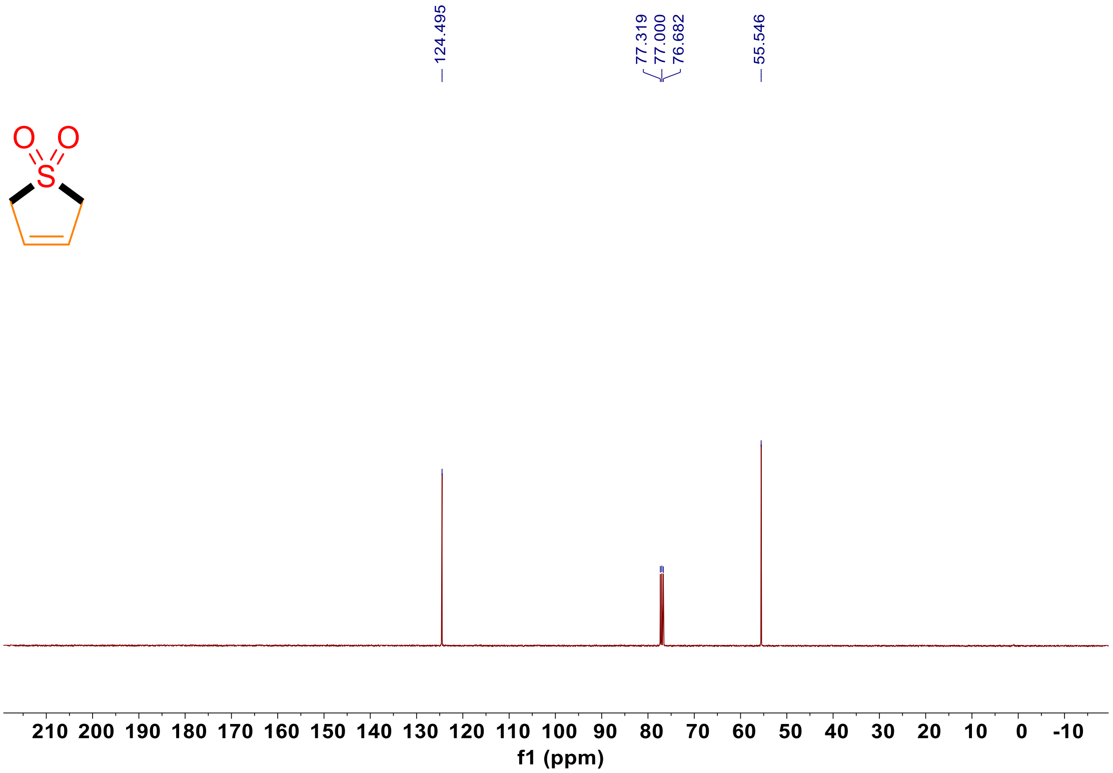
124.495
77.319
77.000
76.682
55.546
210 200 190 180 170 160 150 140 130 120 110 100 90 80 70 60 50 40 30 20 10 0 -10
f1 (ppm)

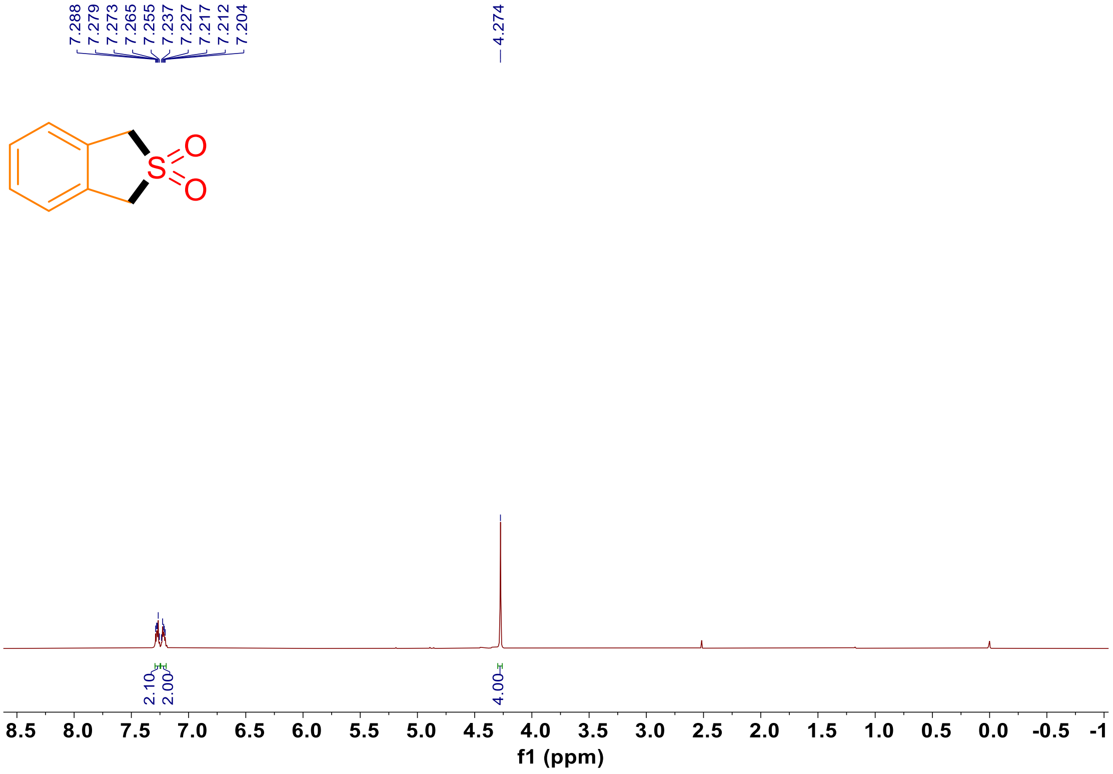
7.288
7.279
7.273
7.265
7.255
7.237
7.227
7.217
7.212
7.204
4.274
2.10
2.00
4.00
8.5 8.0 7.5 7.0 6.5 6.0 5.5 5.0 4.5 4.0 3.5 3.0 2.5 2.0 1.5 1.0 0.5 0.0 -0.5 -1
f1 (ppm)

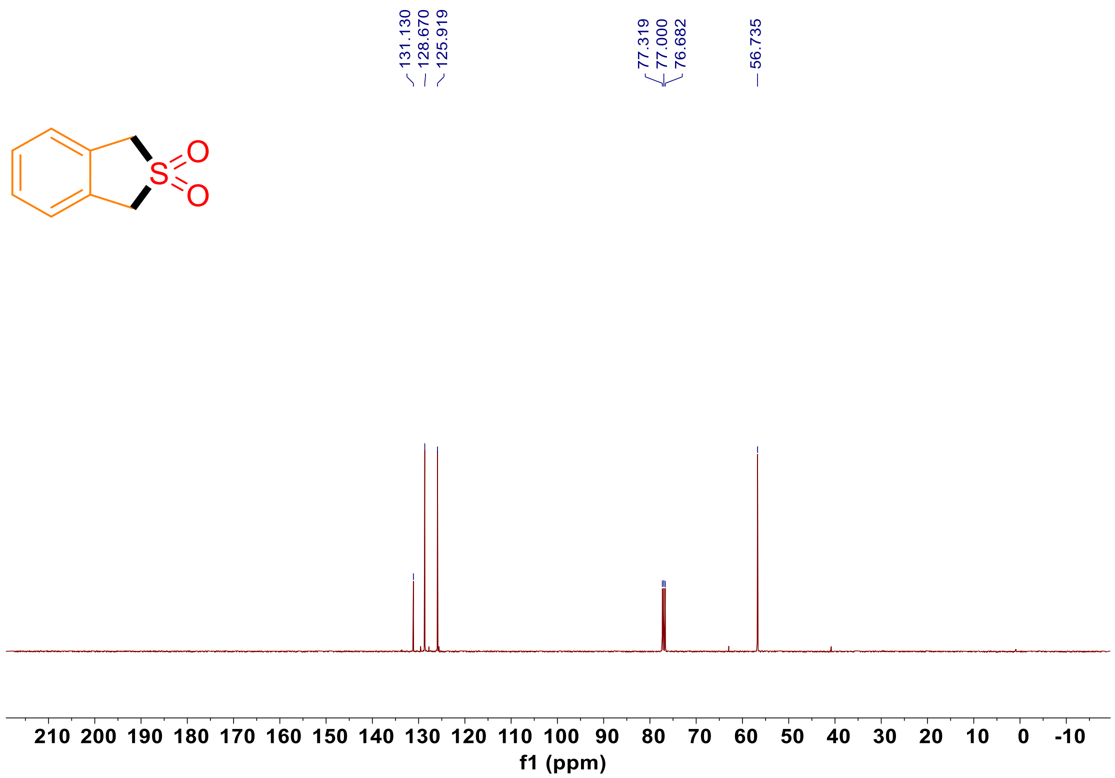
131.130
128.670
125.919
77.319
77.000
76.682
56.735
O
S
O
210 200 190 180 170 160 150 140 130 120 110 100 90 80 70 60 50 40 30 20 10 0 -10
f1 (ppm)

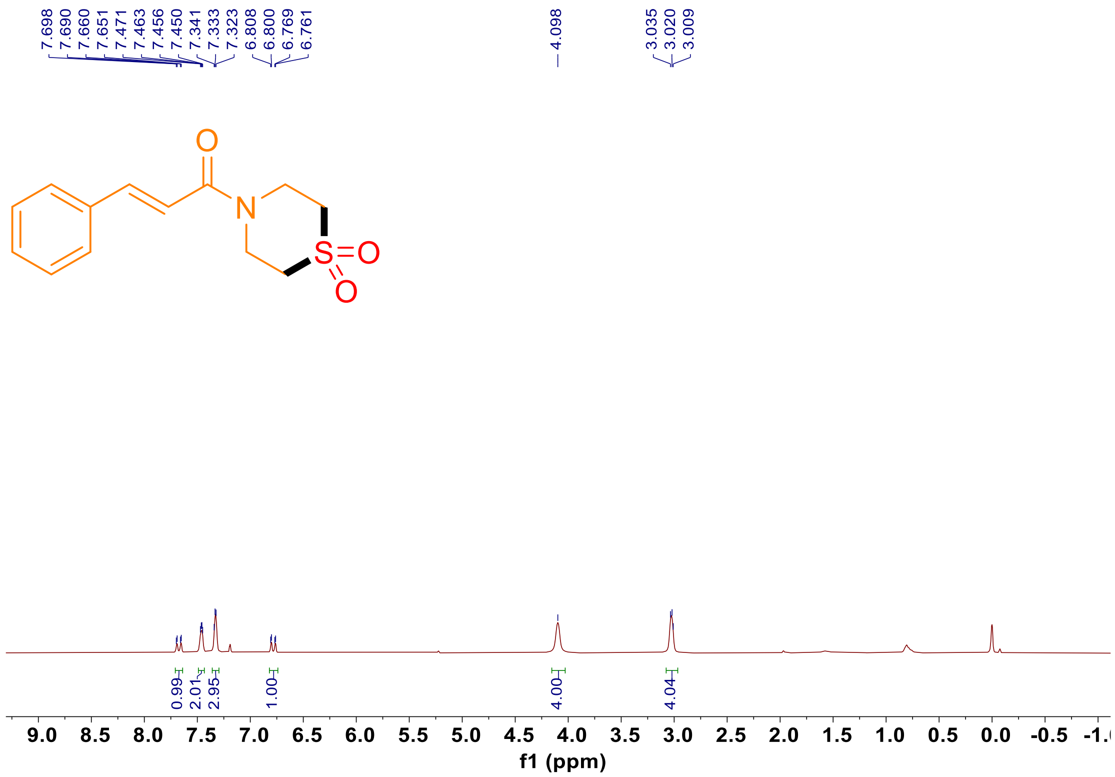
7.698
7.690
7.660
7.651
7.471
7.463
7.456
7.450
7.341
7.333
7.323
6.808
6.800
6.769
6.761
4.098
3.035
3.020
3.009
O
N
S
O
O
0.99
2.01
2.95
1.00
4.00
4.04
9.0 8.5 8.0 7.5 7.0 6.5 6.0 5.5 5.0 4.5 4.0 3.5 3.0 2.5 2.0 1.5 1.0 0.5 0.0 -0.5
f1 (ppm)

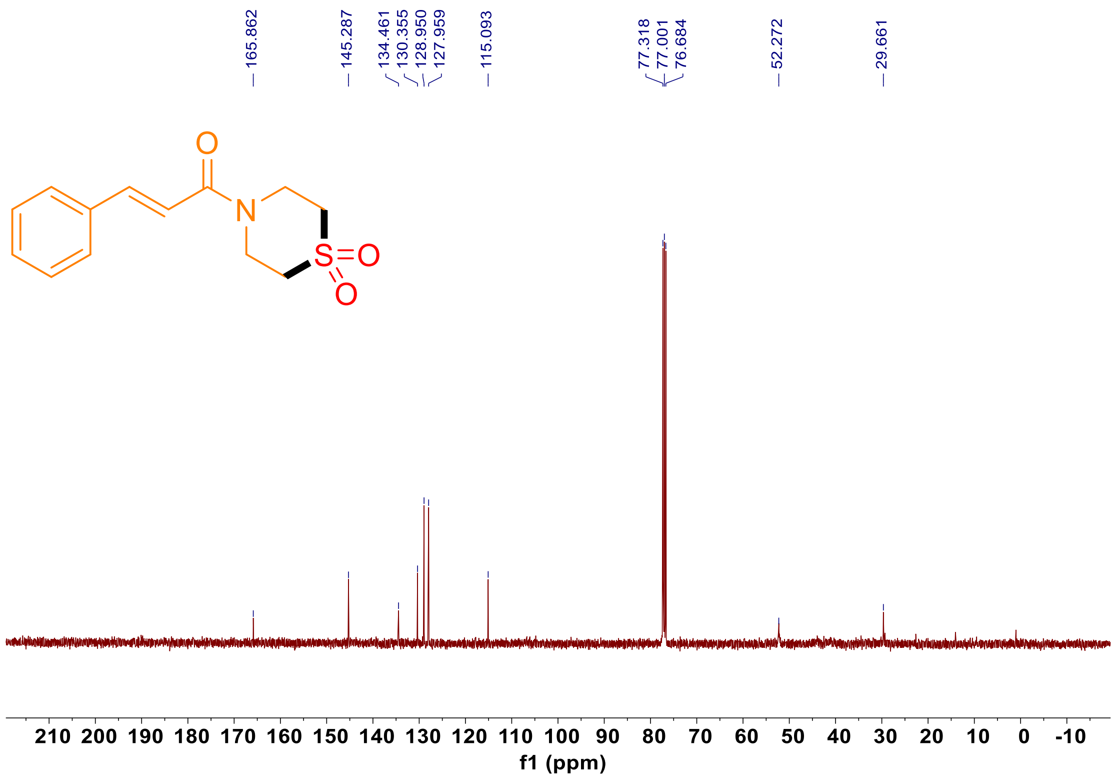
165.862
145.287
134.461
130.355
128.950
127.959
115.093
77.318
77.001
76.684
52.272
29.661
210 200 190 180 170 160 150 140 130 120 110 100 90 80 70 60 50 40 30 20 10 0 -10
f1 (ppm)

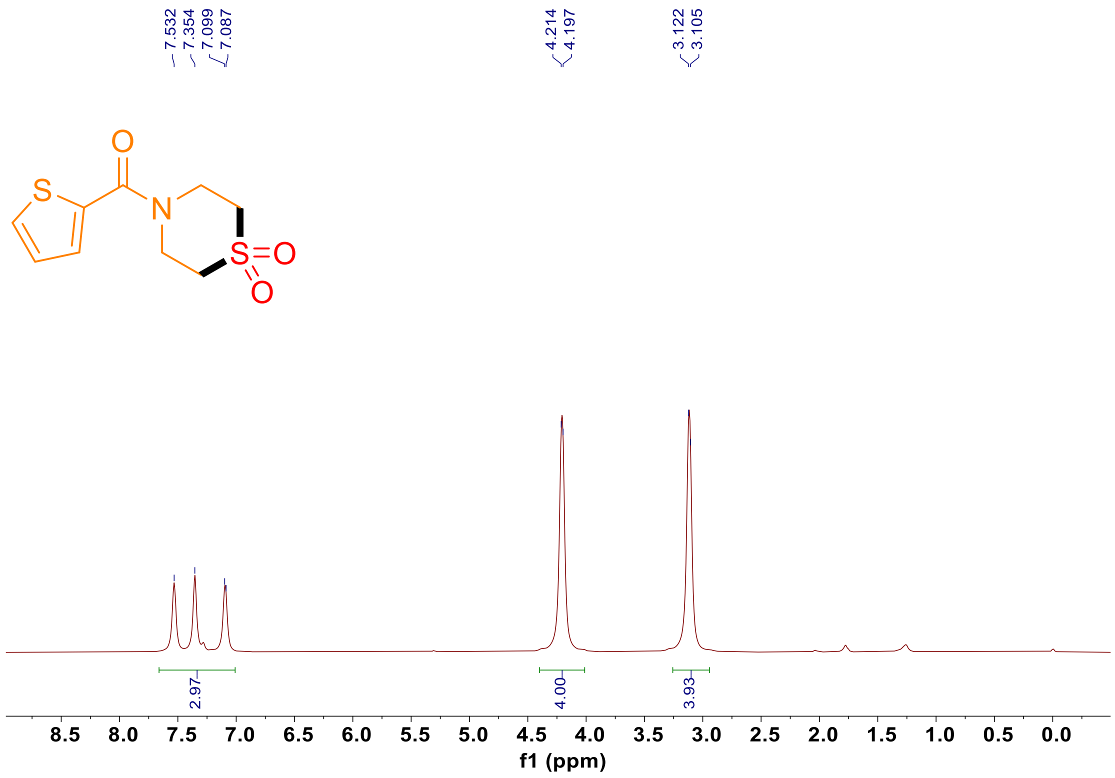
7.532
7.354
7.099
7.087
4.214
4.197
3.122
3.105
2.97
4.00
3.93
8.5 8.0 7.5 7.0 6.5 6.0 5.5 5.0 4.5 4.0 3.5 3.0 2.5 2.0 1.5 1.0 0.5 0.0
f1 (ppm)

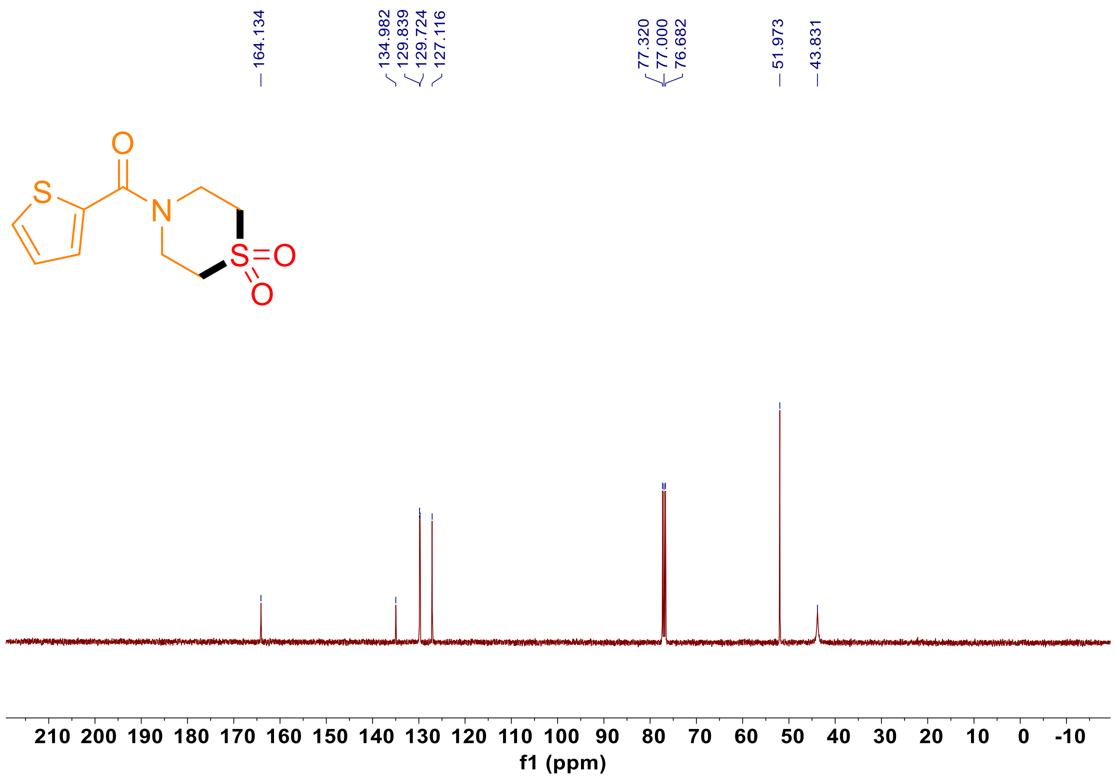
164.134
134.982
129.839
129.724
127.116
77.320
77.000
76.682
51.973
43.831
S
O
N
S
O
O
210 200 190 180 170 160 150 140 130 120 110 100 90 80 70 60 50 40 30 20 10 0 -10
f1 (ppm)

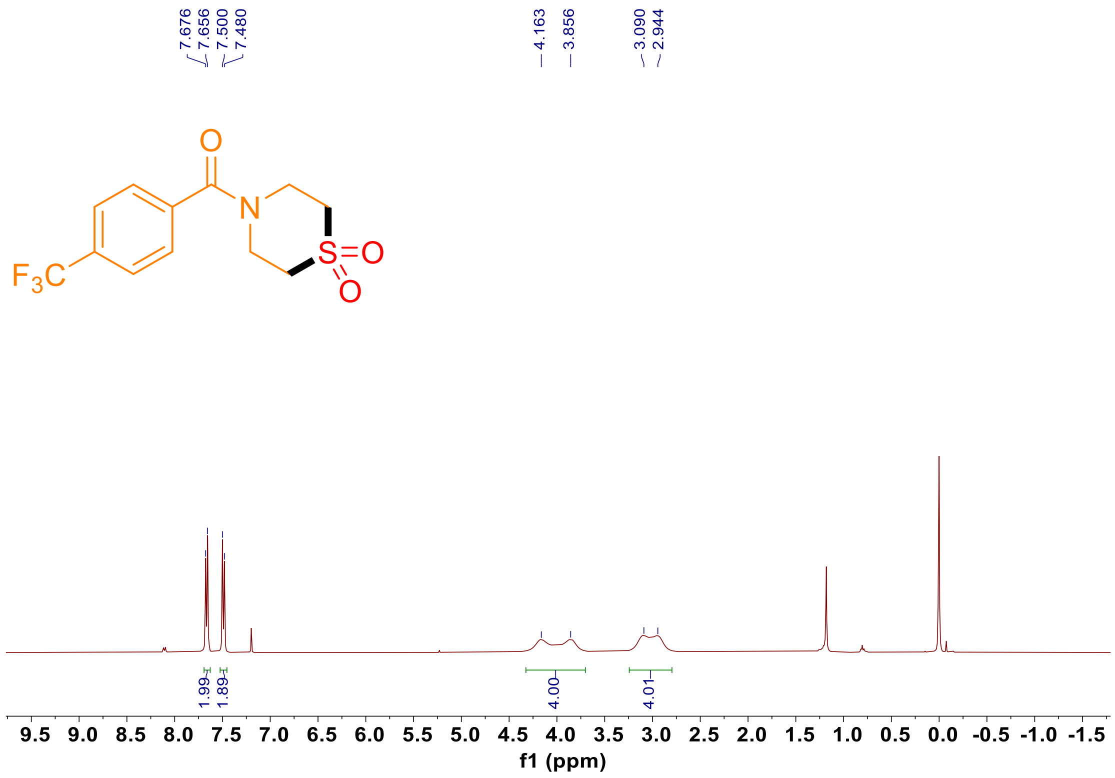
7.676
7.656
7.500
7.480
4.163
3.856
3.090
2.944
O
N
S
O
O
F3C
1.99
1.89
4.00
4.01
9.5 9.0 8.5 8.0 7.5 7.0 6.5 6.0 5.5 5.0 4.5 4.0 3.5 3.0 2.5 2.0 1.5 1.0 0.5 0.0 -0.5 -1.0 -1.5
f1 (ppm)

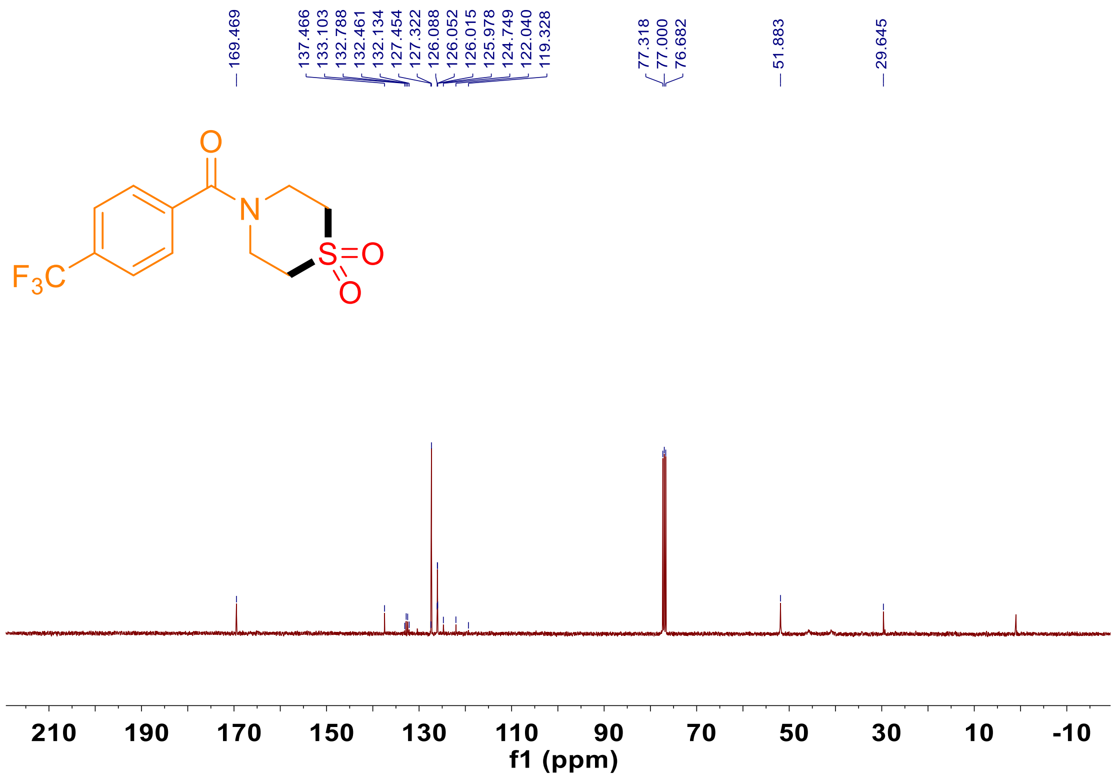
169.469
137.466
133.103
132.788
132.461
132.134
127.454
127.322
126.088
126.052
126.015
125.978
124.749
122.040
119.328
77.318
77.000
76.682
51.883
29.645
F3C
O
N
S
O
O
210
190
170
150
130
110
90
70
50
30
10
-10
f1 (ppm)

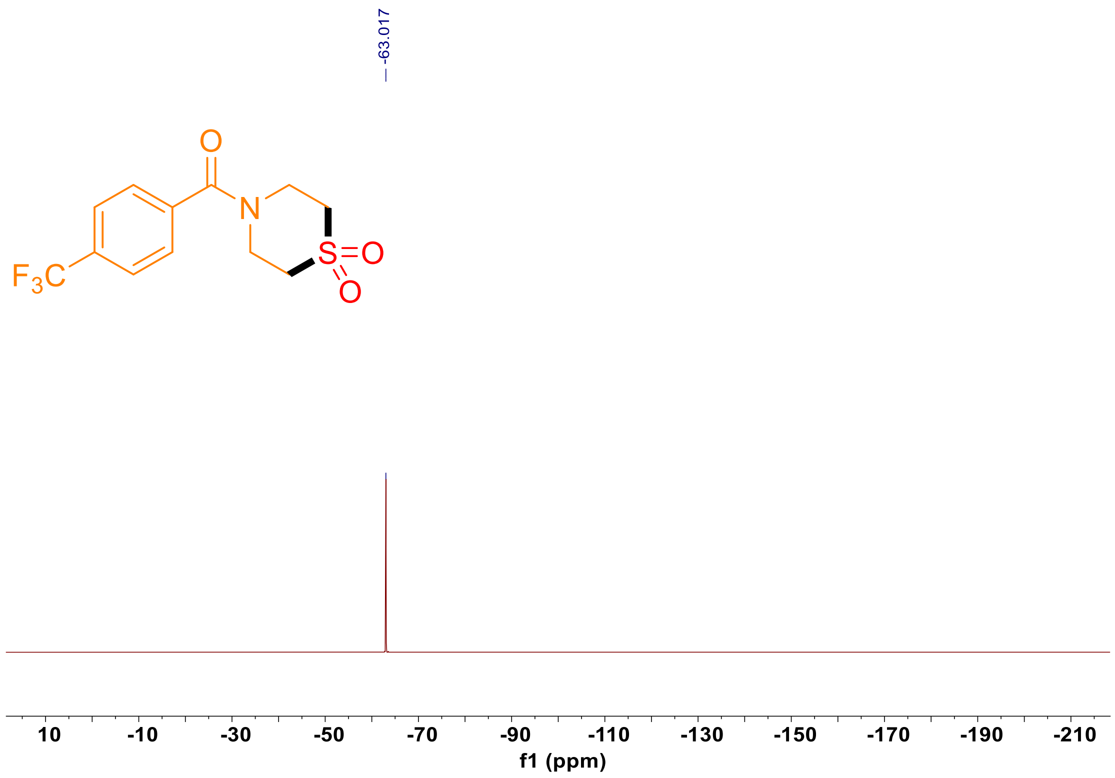
-63.017
F3C
O
N
S
O
O
10
-10
-30
-50
-70
-90
-110
-130
-150
-170
-190
-210
f1 (ppm)

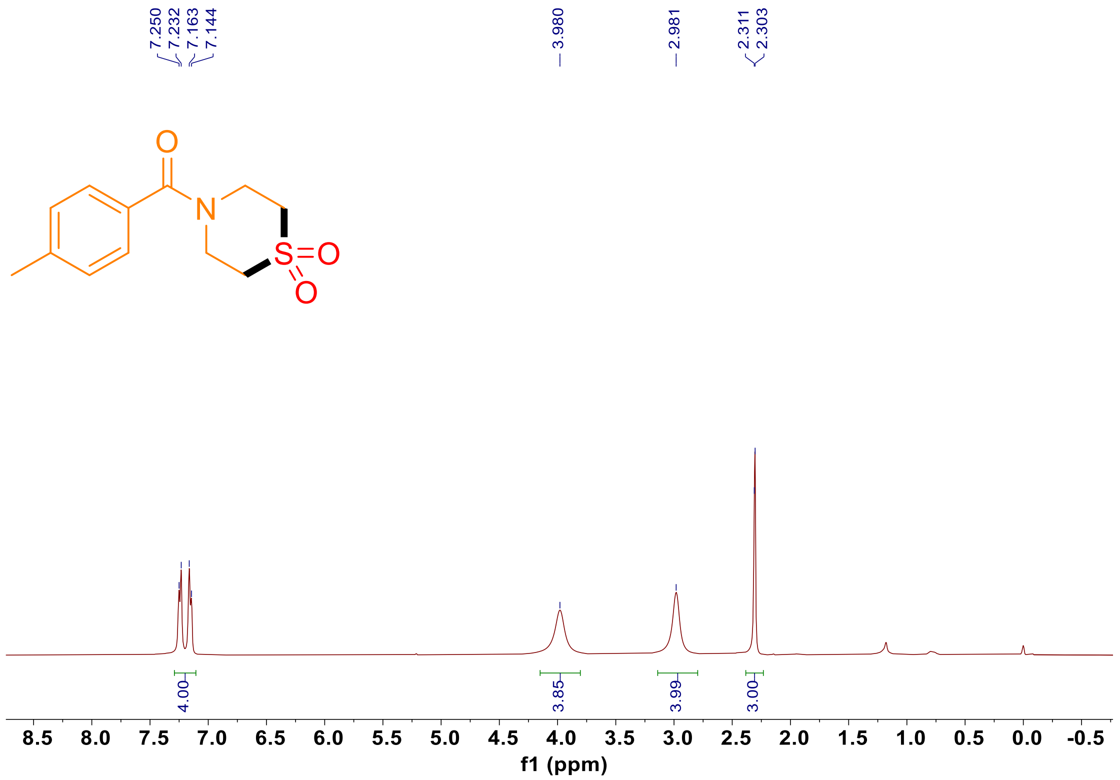
7.250
7.232
7.163
7.144
3.980
2.981
2.311
2.303
O
N
S
O
O
4.00
3.85
3.99
3.00
8.5 8.0 7.5 7.0 6.5 6.0 5.5 5.0 4.5 4.0 3.5 3.0 2.5 2.0 1.5 1.0 0.5 0.0 -0.5
f1 (ppm)

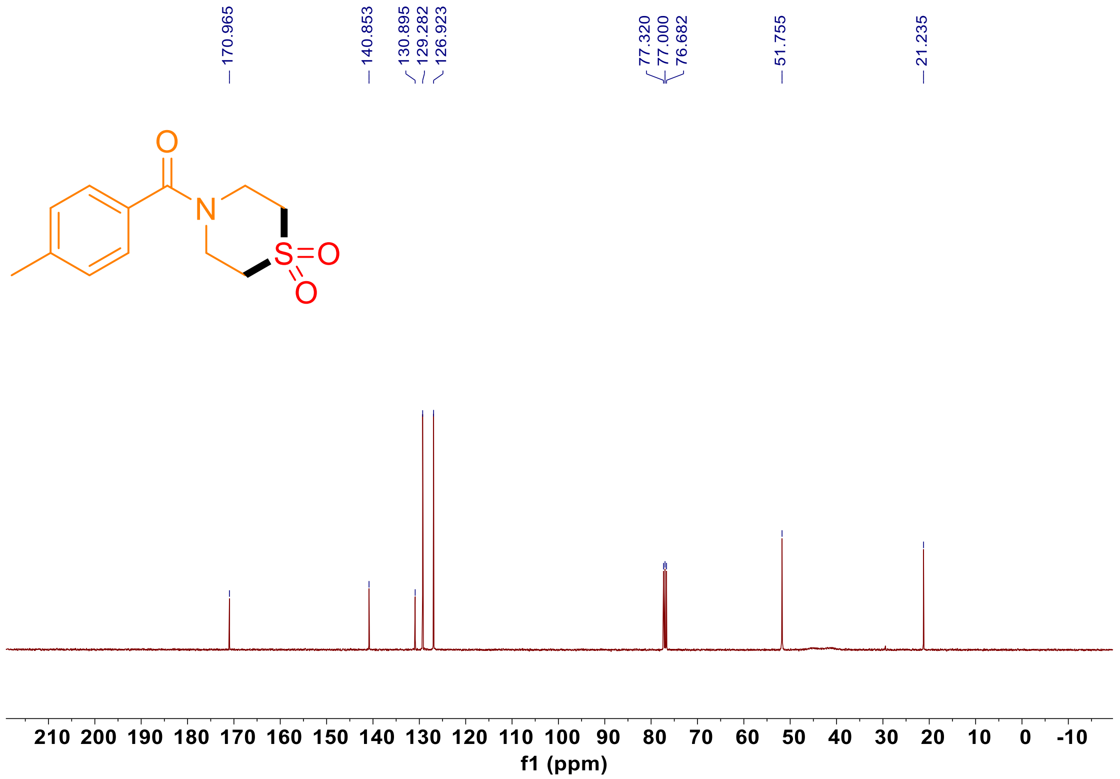
170.965
140.853
130.895
129.282
126.923
77.320
77.000
76.682
51.755
21.235
O
N
S
O
O
210 200 190 180 170 160 150 140 130 120 110 100 90 80 70 60 50 40 30 20 10 0 -10
f1 (ppm)

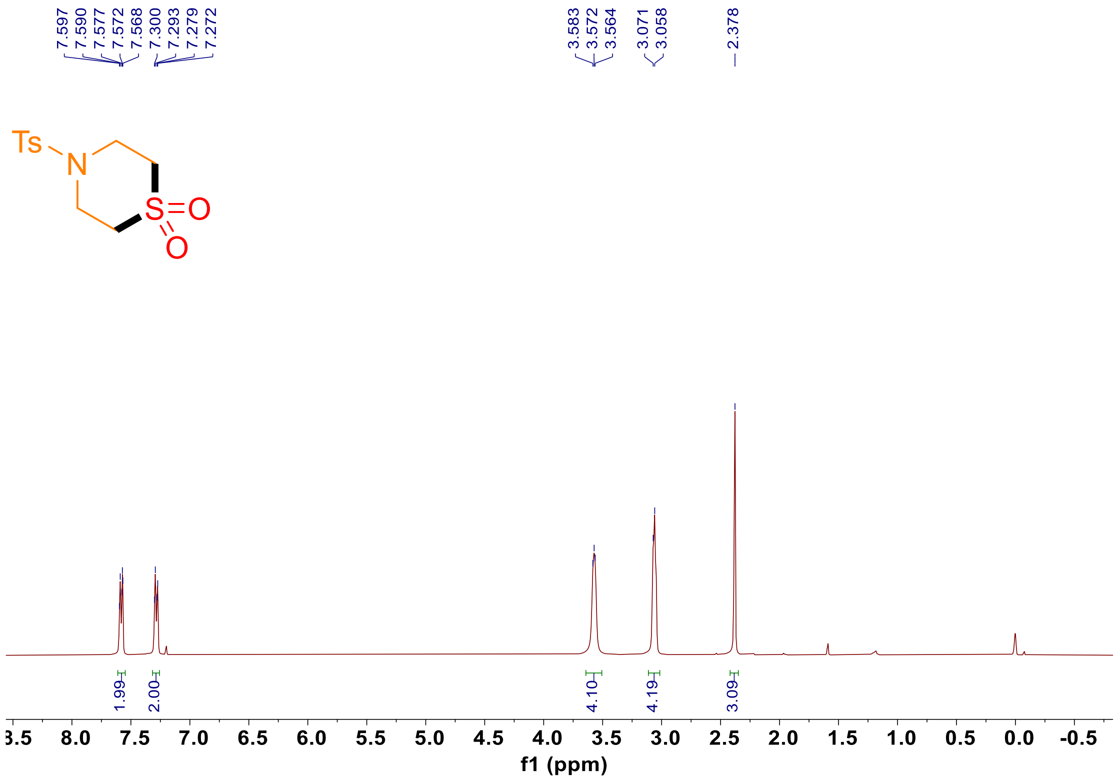
7.597
7.590
7.577
7.572
7.568
7.300
7.293
7.279
7.272
3.583
3.572
3.564
3.071
3.058
2.378
Ts
N
S
O
O
1.99
2.00
4.10
4.19
3.09
f1 (ppm)

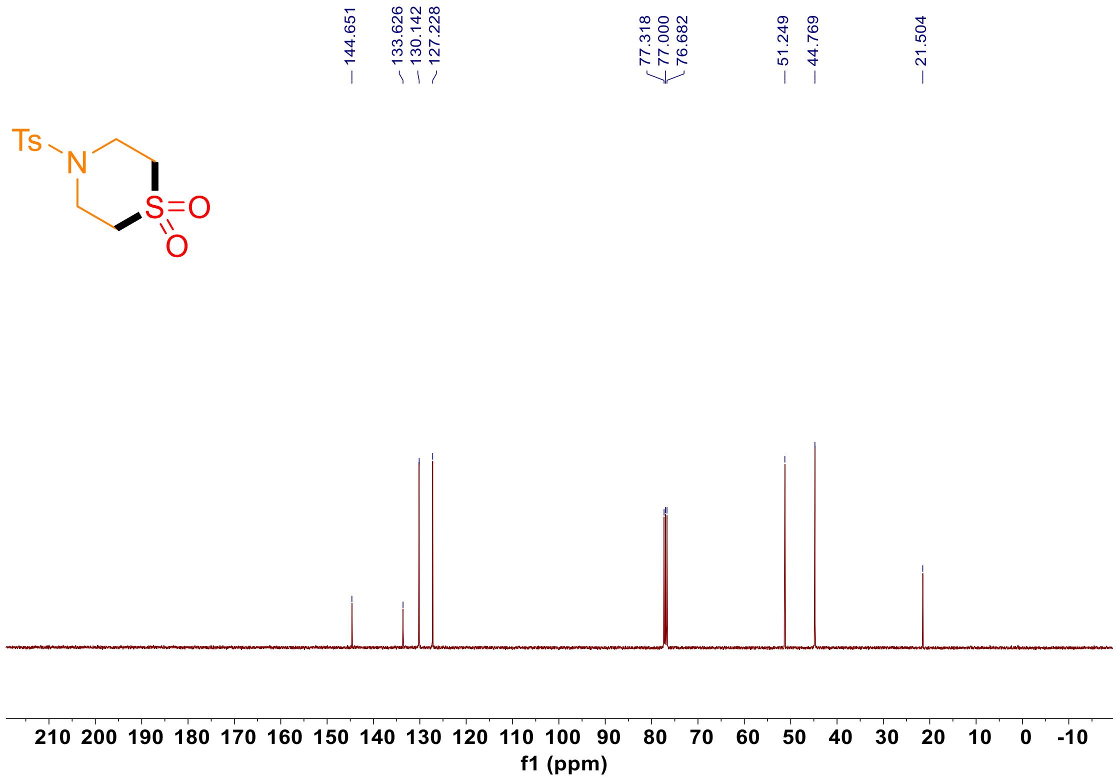
144.651
133.626
130.142
127.228
77.318
77.000
76.682
51.249
44.769
21.504
Ts
N
S
O
O
f1 (ppm)

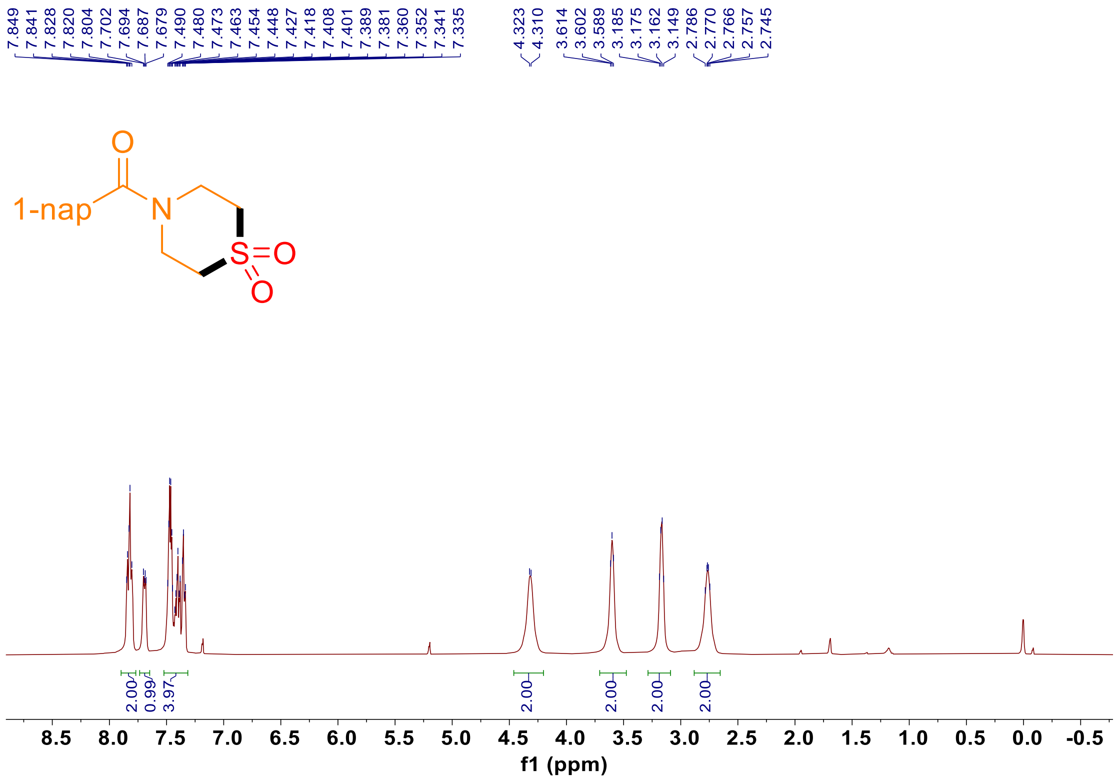
7.849
7.841
7.828
7.820
7.804
7.702
7.694
7.687
7.679
7.490
7.480
7.473
7.463
7.454
7.448
7.427
7.418
7.408
7.401
7.389
7.381
7.360
7.352
7.341
7.335
4.323
4.310
3.614
3.602
3.589
3.185
3.175
3.162
3.149
2.786
2.770
2.766
2.757
2.745
O
1-nap
N
S
O
O
2.00
0.99
3.97
2.00
2.00
2.00
2.00
8.5 8.0 7.5 7.0 6.5 6.0 5.5 5.0 4.5 4.0 3.5 3.0 2.5 2.0 1.5 1.0 0.5 0.0 -0.5
f1 (ppm)

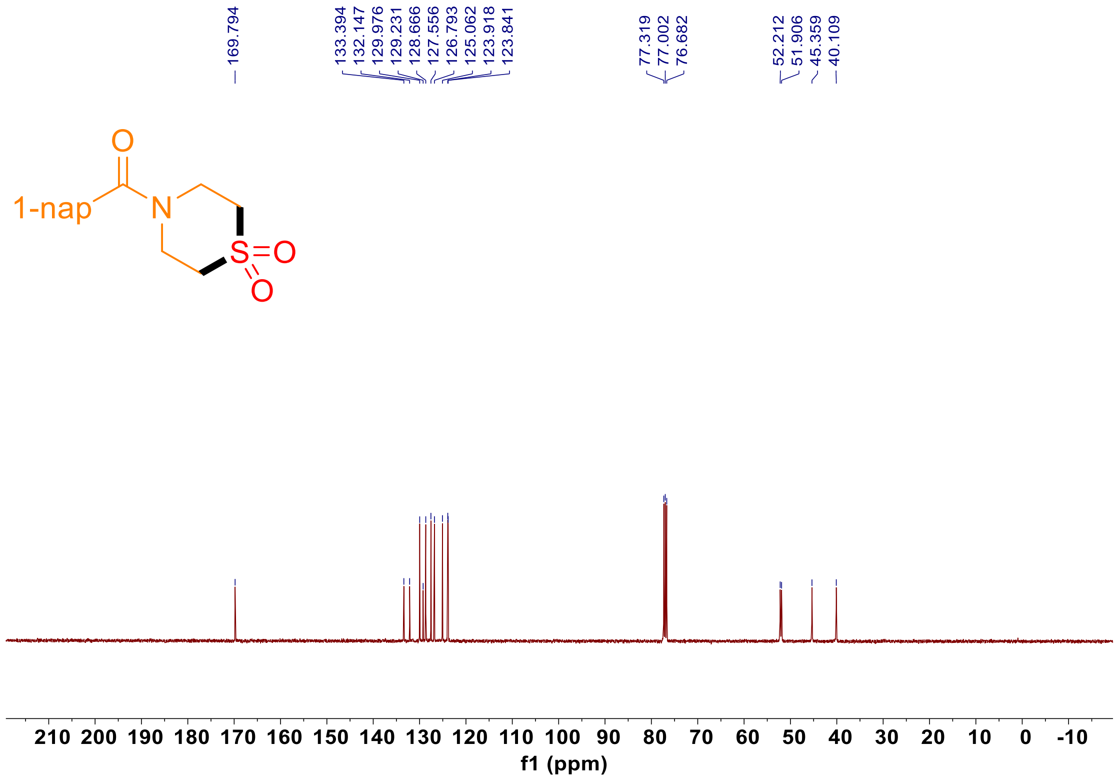
169.794
133.394
132.147
129.976
129.231
128.666
127.556
126.793
125.062
123.918
123.841
77.319
77.002
76.682
52.212
51.906
45.359
40.109
O
1-nap
N
S
O
O
210 200 190 180 170 160 150 140 130 120 110 100 90 80 70 60 50 40 30 20 10 0 -10
f1 (ppm)

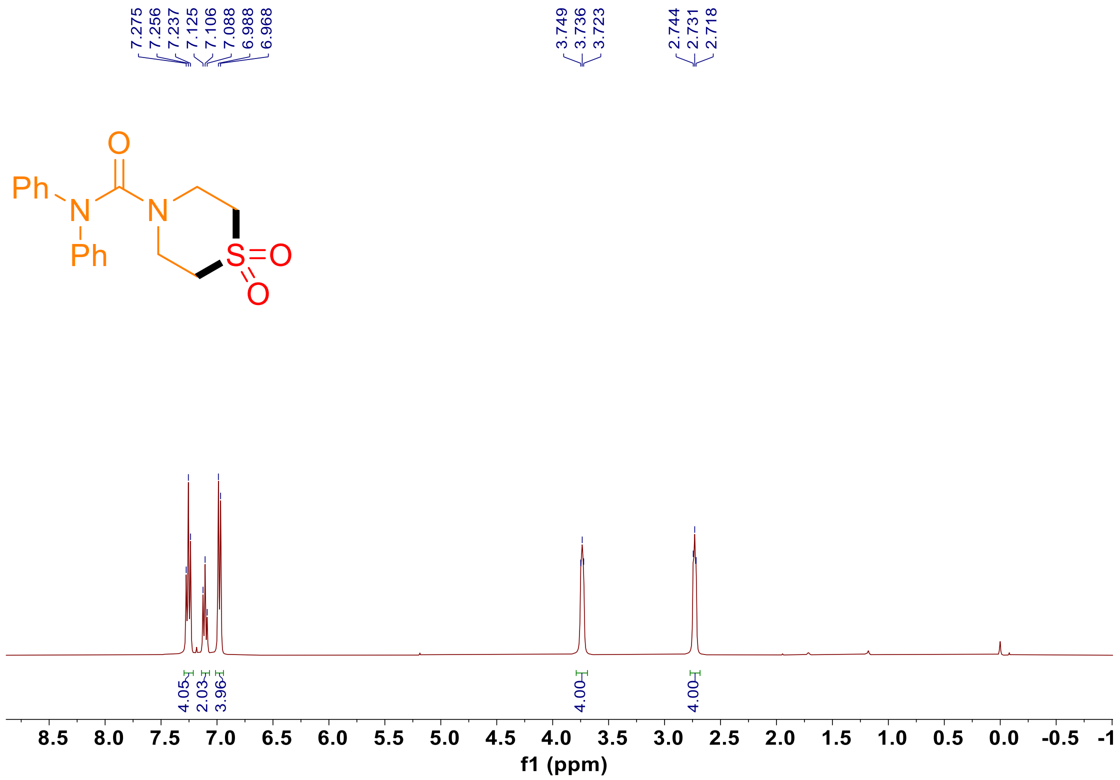
7.275
7.256
7.237
7.125
7.106
7.088
6.988
6.968
3.749
3.736
3.723
2.744
2.731
2.718
O
Ph
N
N
Ph
S
O
O
4.05
2.03
3.96
4.00
4.00
8.5 8.0 7.5 7.0 6.5 6.0 5.5 5.0 4.5 4.0 3.5 3.0 2.5 2.0 1.5 1.0 0.5 0.0 -0.5 -1
f1 (ppm)

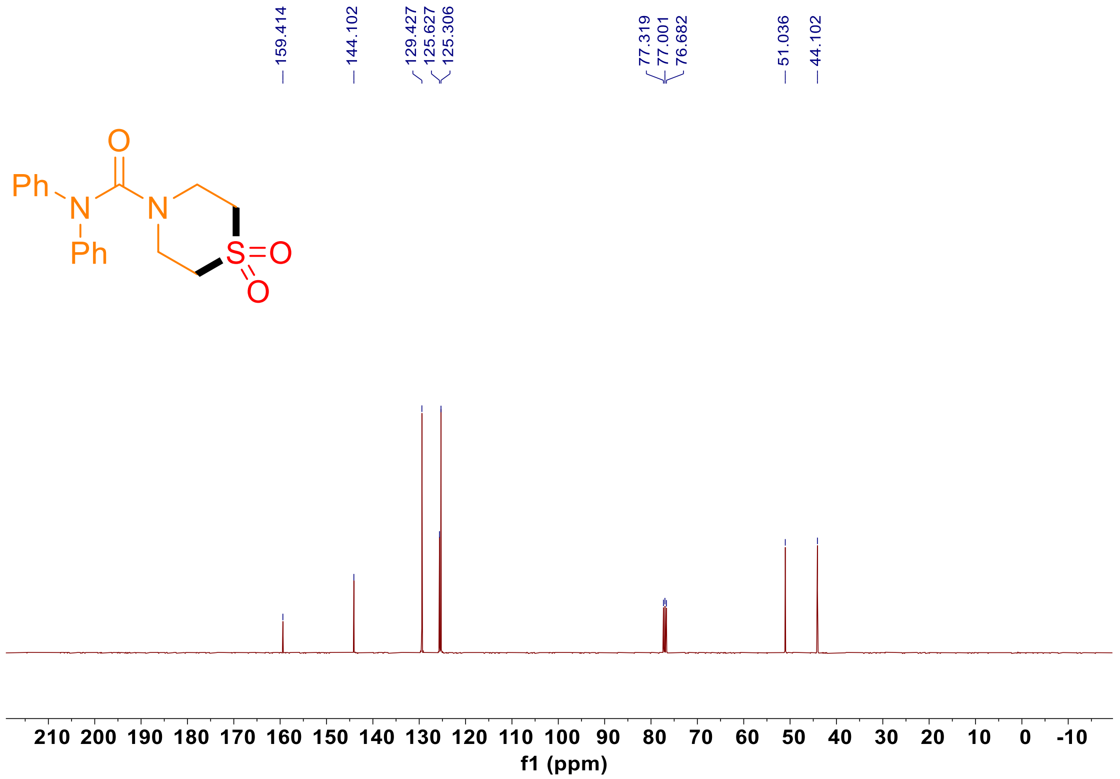
159.414
144.102
129.427
125.627
125.306
77.319
77.001
76.682
51.036
44.102
O
Ph
N
N
Ph
S
O
O
210 200 190 180 170 160 150 140 130 120 110 100 90 80 70 60 50 40 30 20 10 0 -10
f1 (ppm)

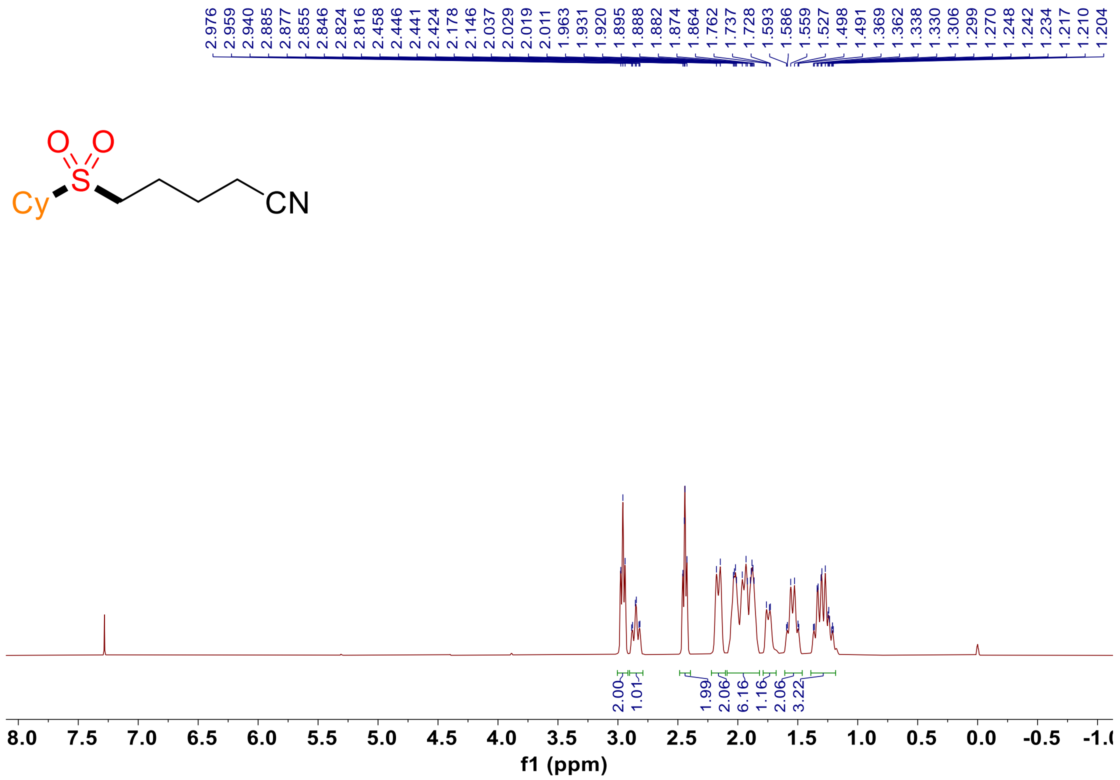

2.976
2.959
2.940
2.885
2.877
2.855
2.846
2.824
2.816
2.458
2.446
2.441
2.424
2.178
2.146
2.037
2.029
2.019
2.011
1.963
1.931
1.920
1.895
1.888
1.882
1.874
1.864
1.762
1.737
1.728
1.593
1.586
1.559
1.527
1.498
1.491
1.369
1.362
1.338
1.330
1.306
1.299
1.270
1.248
1.242
1.234
1.217
1.210
1.204
Cy
S
O O
CN
2.00
1.01
1.99
2.06
6.16
1.16
2.06
3.22
8.0 7.5 7.0 6.5 6.0 5.5 5.0 4.5 4.0 3.5 3.0 2.5 2.0 1.5 1.0 0.5 0.0 -0.5
f1 (ppm)

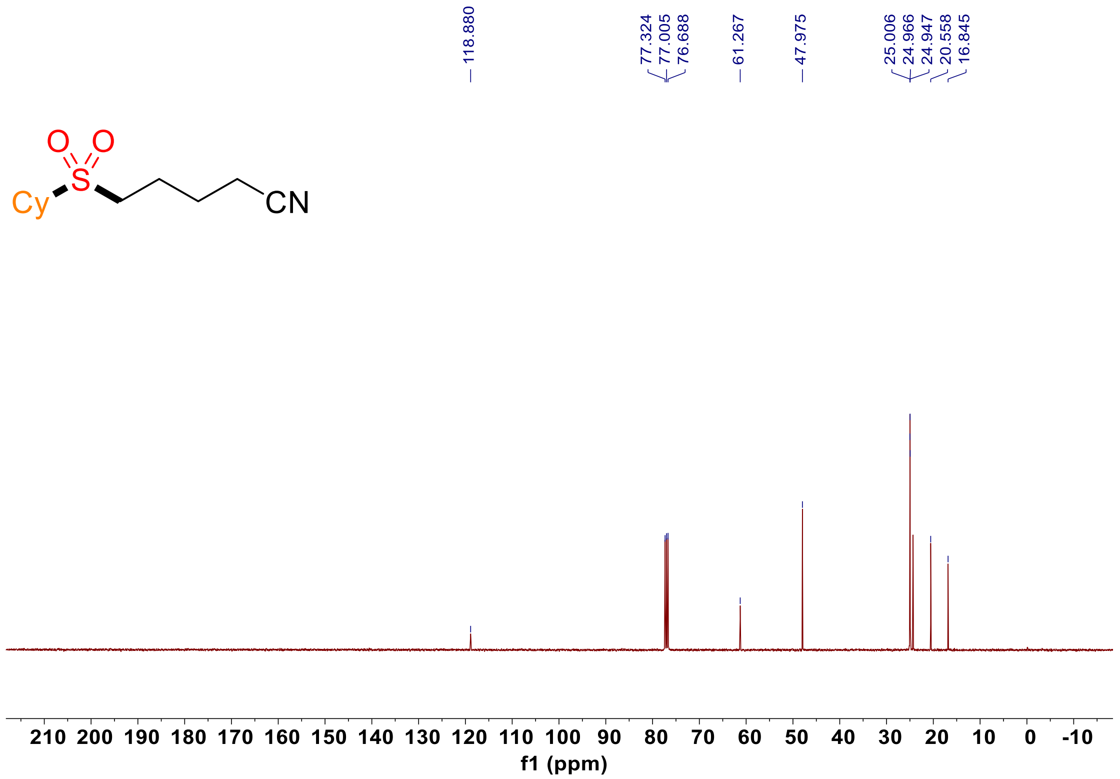

118.880
77.324
77.005
76.688
61.267
47.975
25.006
24.966
24.947
20.558
16.845
Cy
S
O O
CN
210 200 190 180 170 160 150 140 130 120 110 100 90 80 70 60 50 40 30 20 10 0 -10
f1 (ppm)

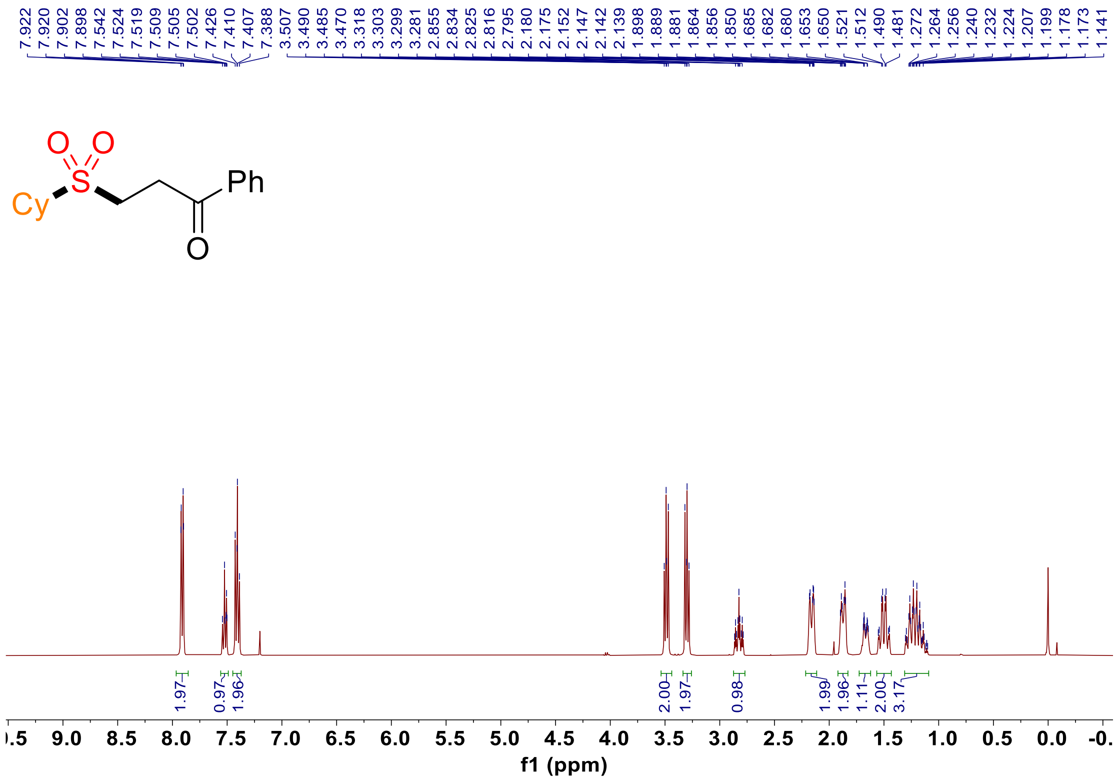

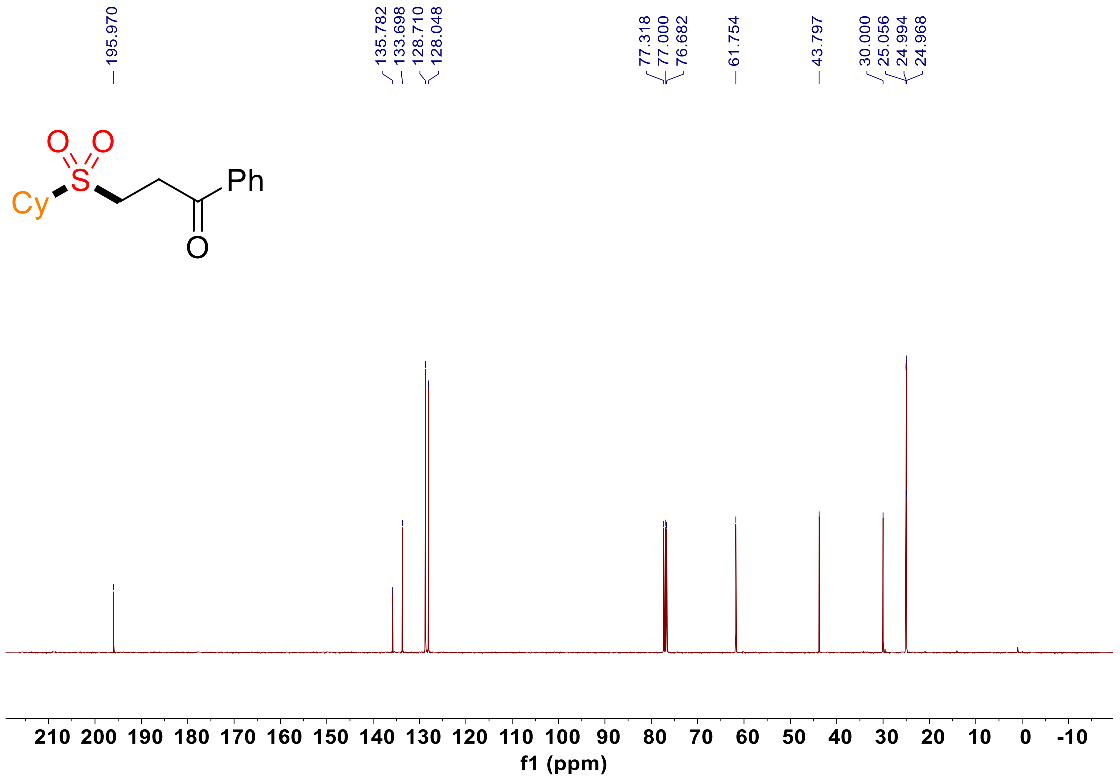

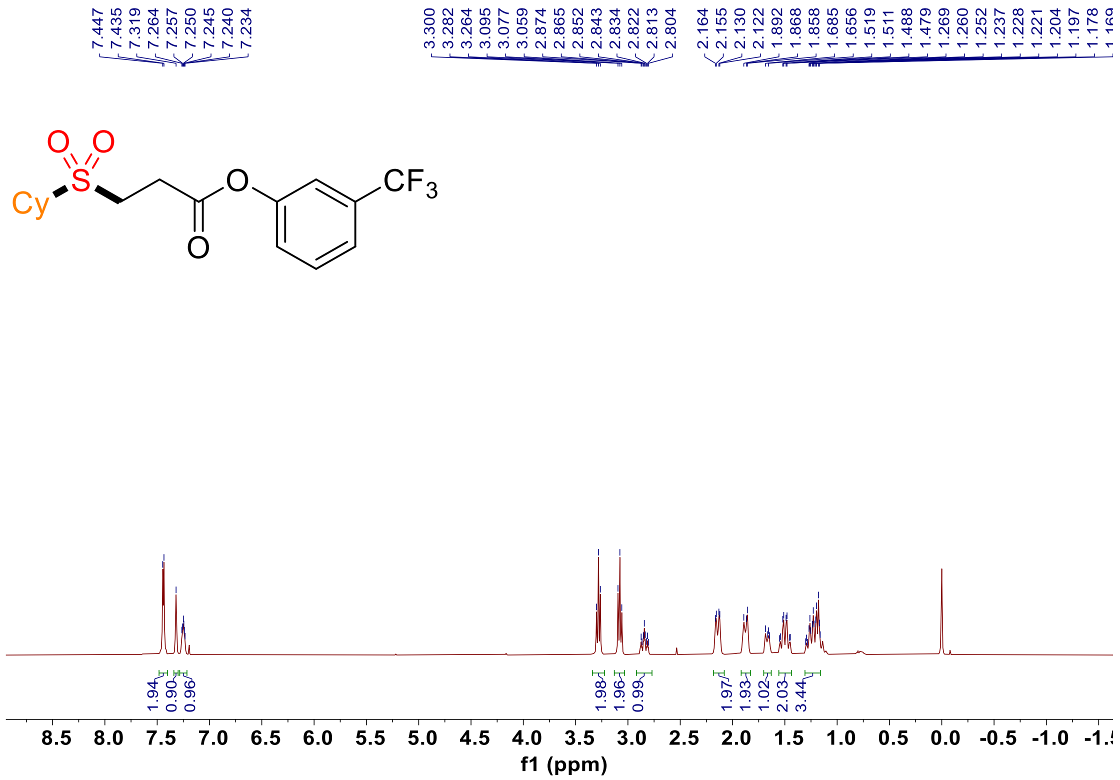

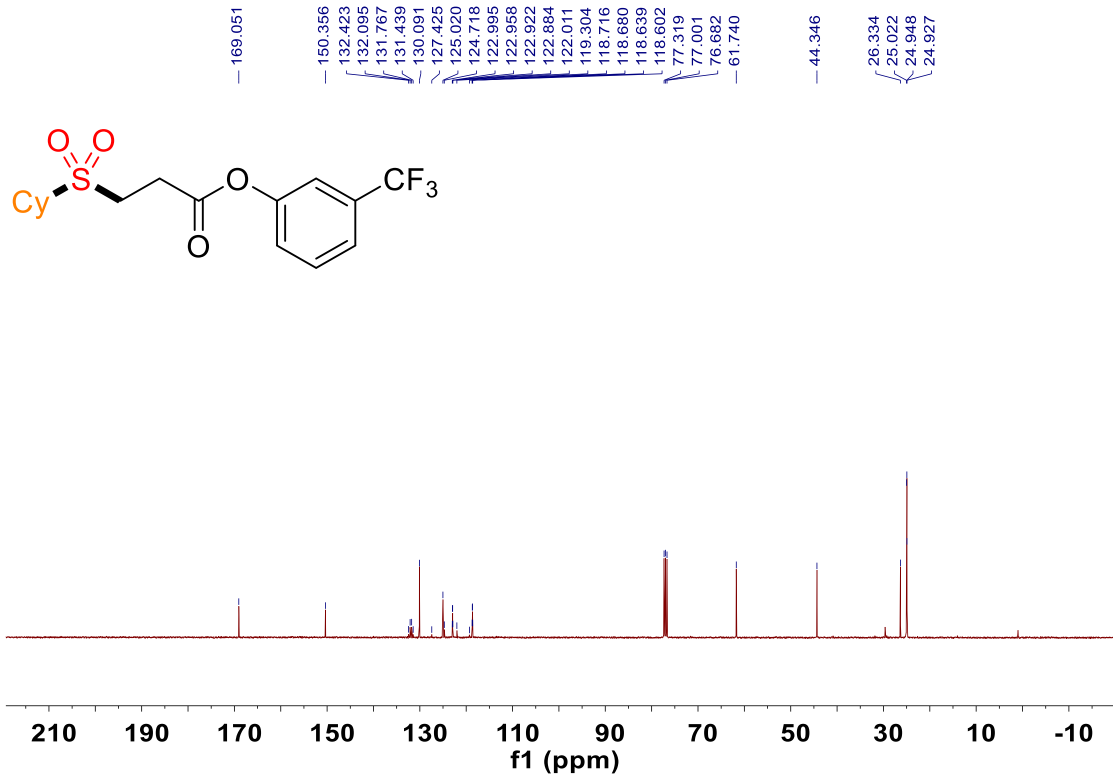

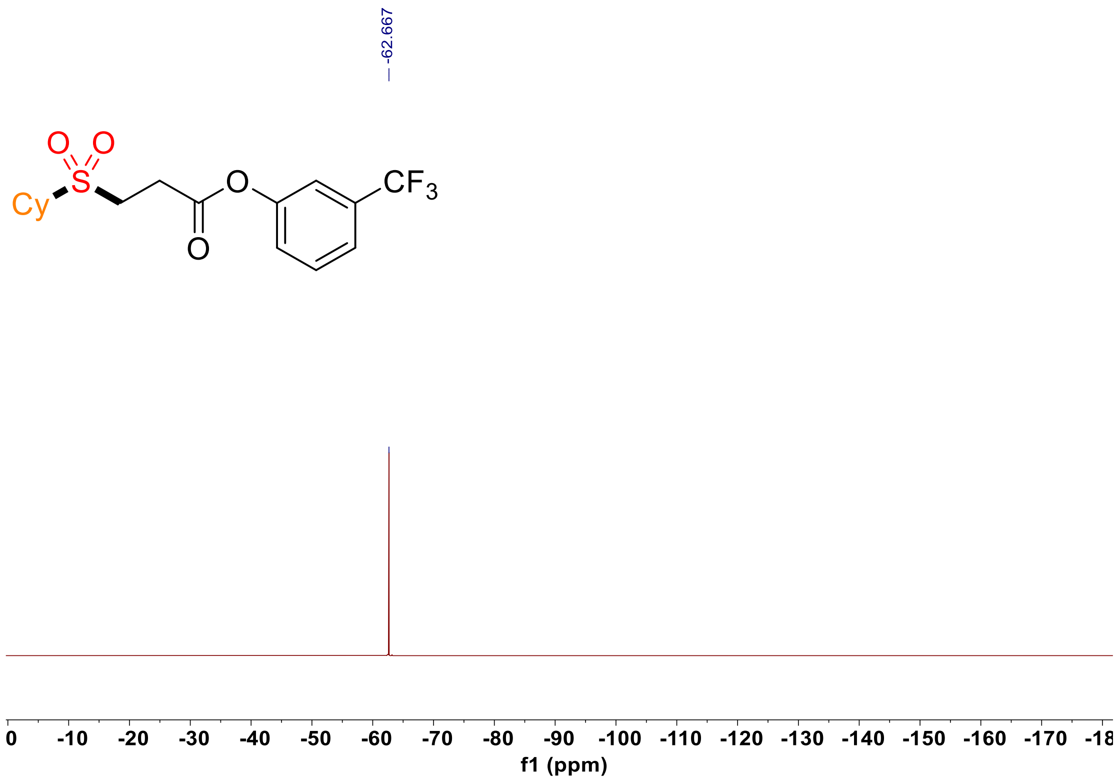
-62.667
Cy
CF3
f1 (ppm)

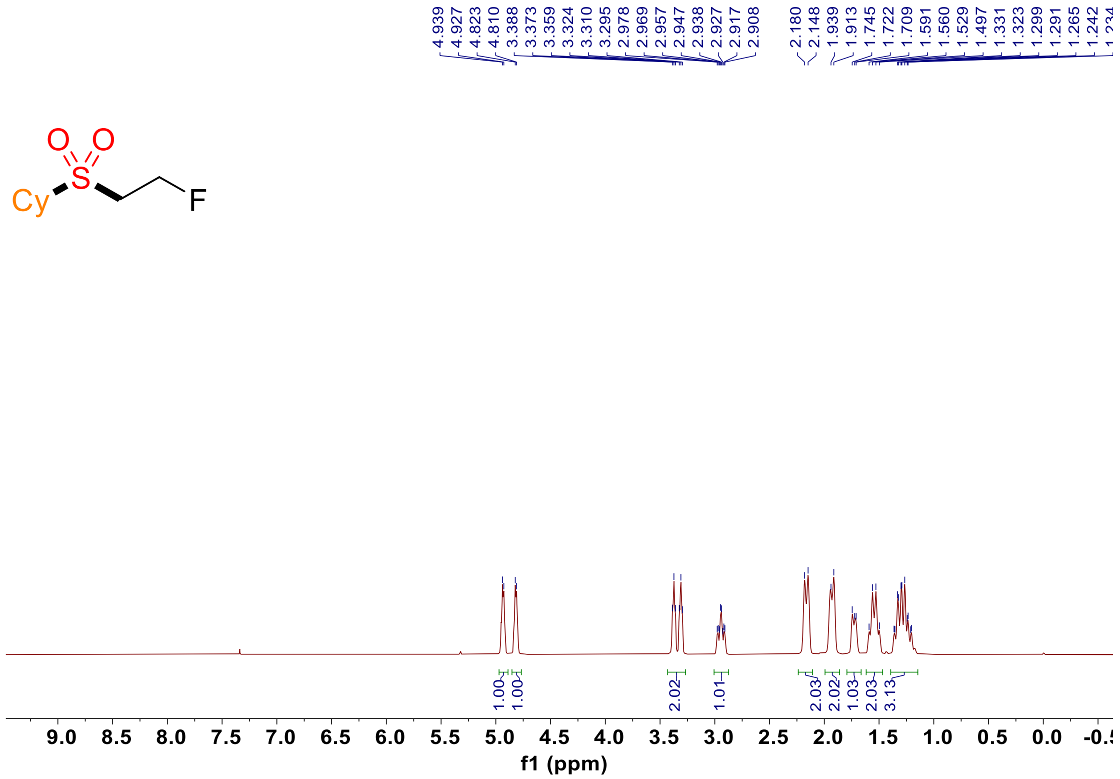
4.939
4.927
4.823
4.810
3.388
3.373
3.359
3.324
3.310
3.295
2.978
2.969
2.957
2.947
2.938
2.927
2.917
2.908
2.180
2.148
1.939
1.913
1.745
1.722
1.709
1.591
1.560
1.529
1.497
1.331
1.323
1.299
1.291
1.265
1.242
Cy
F
1.00
1.00
2.02
1.01
2.03
2.02
1.03
2.03
3.13
f1 (ppm)

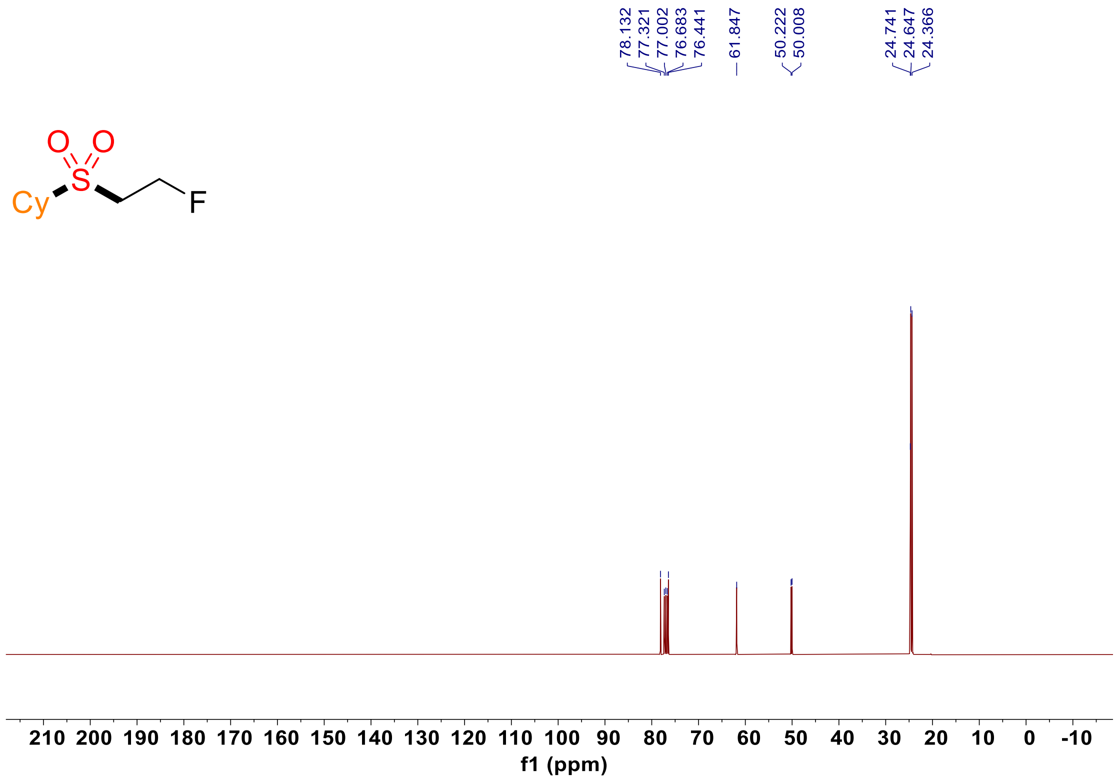

78.132
77.321
77.002
76.683
76.441
61.847
50.222
50.008
24.741
24.647
24.366
Cy
S
O
O
F
210 200 190 180 170 160 150 140 130 120 110 100 90 80 70 60 50 40 30 20 10 0 -10
f1 (ppm)

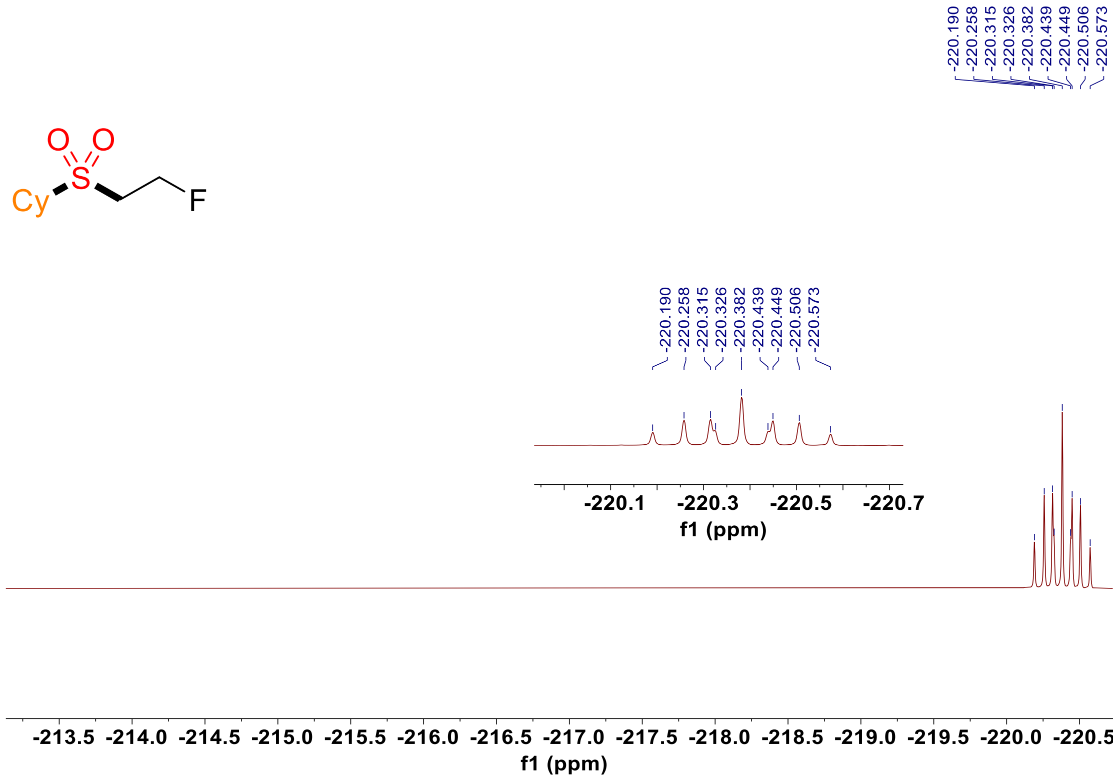

-220.190
-220.258
-220.315
-220.326
-220.382
-220.439
-220.449
-220.506
-220.573
Cy
S
O
O
F
-220.1 -220.3 -220.5 -220.7
f1 (ppm)
-213.5 -214.0 -214.5 -215.0 -215.5 -216.0 -216.5 -217.0 -217.5 -218.0 -218.5 -219.0 -219.5 -220.0 -220.5
f1 (ppm)

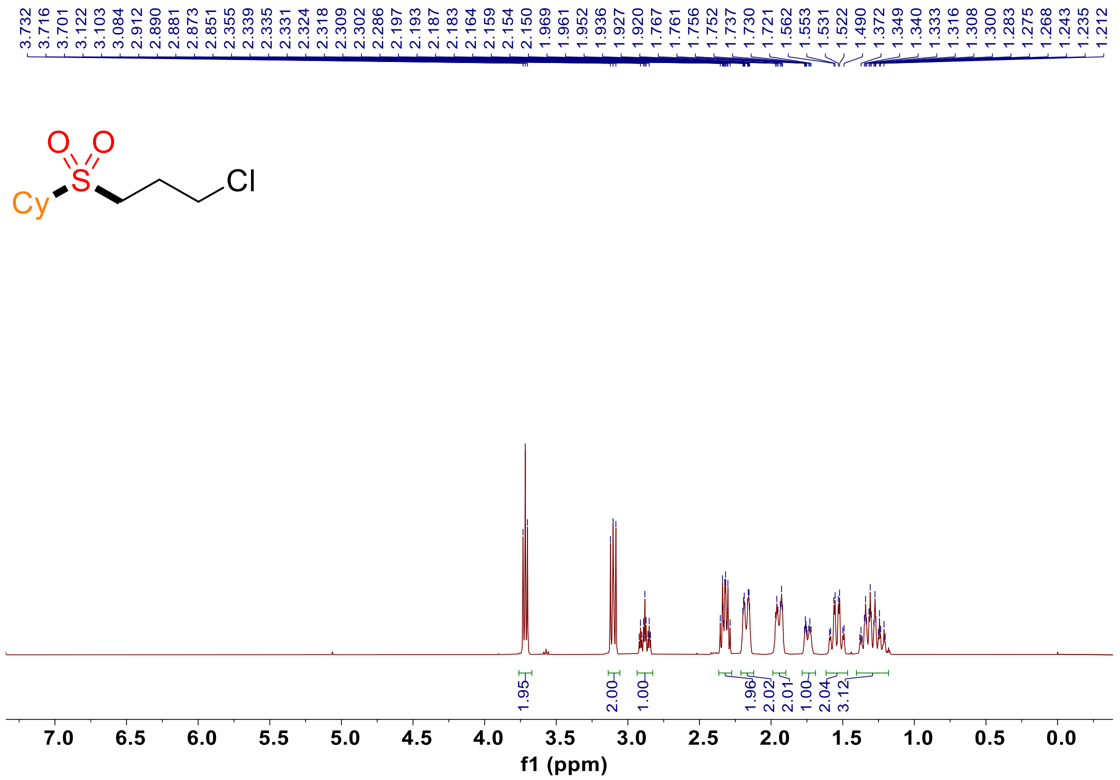
3.732
3.716
3.701
3.122
3.103
3.084
2.912
2.890
2.881
2.873
2.851
2.355
2.339
2.335
2.331
2.324
2.318
2.309
2.302
2.286
2.197
2.193
2.187
2.183
2.164
2.159
2.154
2.150
1.969
1.961
1.952
1.936
1.927
1.920
1.767
1.761
1.756
1.752
1.737
1.730
1.721
1.562
1.553
1.531
1.522
1.490
1.372
1.349
1.340
1.333
1.316
1.308
1.300
1.283
1.275
1.268
1.243
1.235
1.212
O
O
S
Cy
Cl
1.95
2.00
1.00
1.96
2.02
2.01
1.00
2.04
3.12
7.0 6.5 6.0 5.5 5.0 4.5 4.0 3.5 3.0 2.5 2.0 1.5 1.0 0.5 0.0
f1 (ppm)

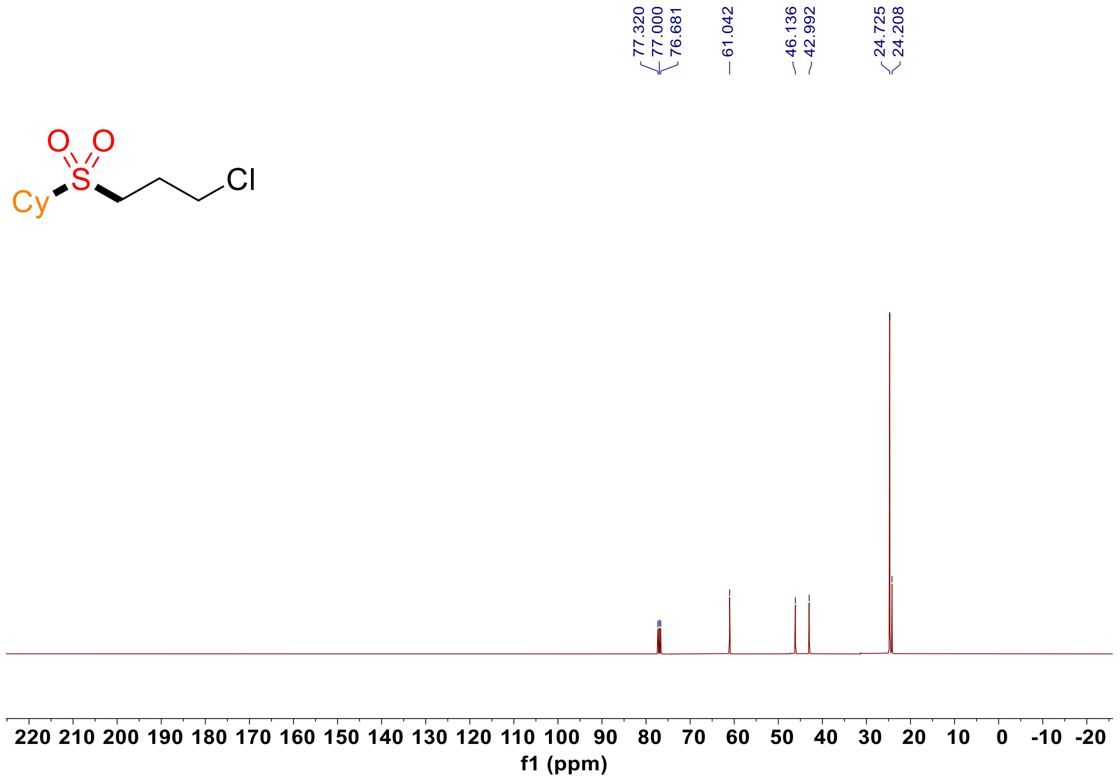
77.320
77.000
76.681
61.042
46.136
42.992
24.725
24.208
O
O
S
Cy
Cl
220 210 200 190 180 170 160 150 140 130 120 110 100 90 80 70 60 50 40 30 20 10 0 -10 -20
f1 (ppm)

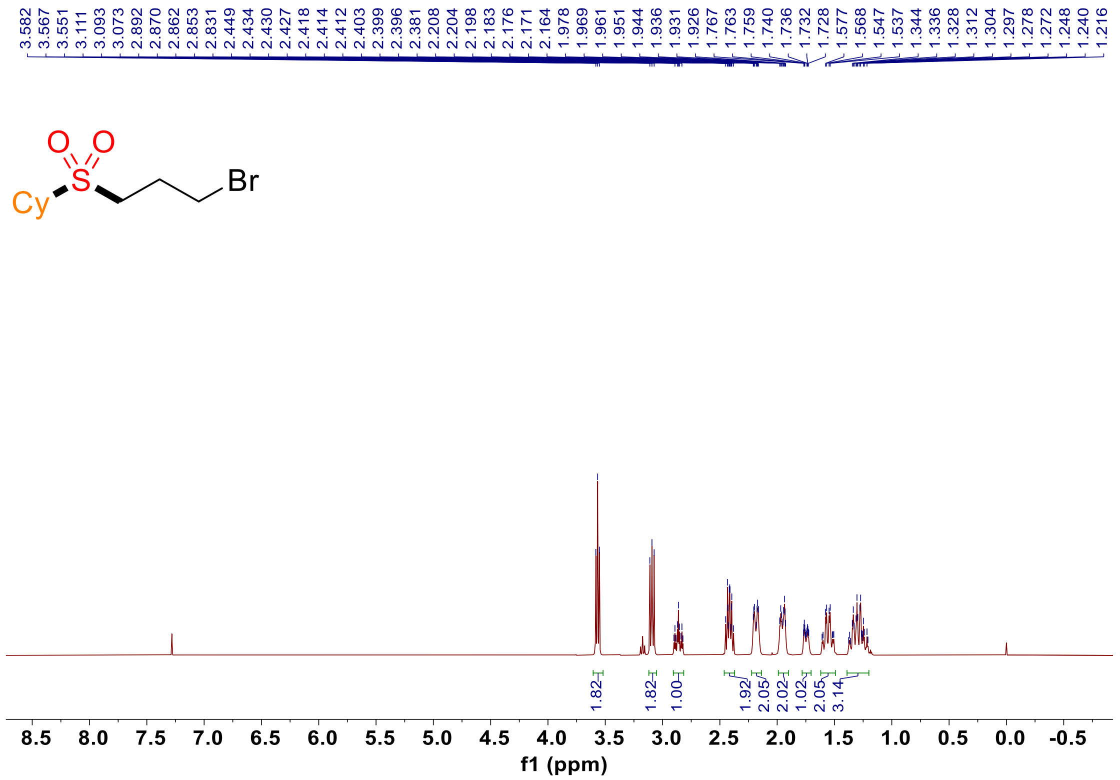
3.582
3.567
3.551
3.111
3.093
3.073
2.892
2.870
2.862
2.853
2.831
2.449
2.434
2.430
2.427
2.418
2.414
2.412
2.403
2.399
2.396
2.381
2.208
2.204
2.198
2.183
2.176
2.171
2.164
1.978
1.969
1.961
1.951
1.944
1.936
1.931
1.926
1.767
1.763
1.759
1.740
1.736
1.732
1.728
1.577
1.568
1.547
1.537
1.344
1.336
1.328
1.312
1.304
1.297
1.278
1.272
1.248
1.240
1.216
Cy
S
O
O
Br
1.82
1.82
1.00
1.92
2.05
2.02
1.02
2.05
3.14
8.5 8.0 7.5 7.0 6.5 6.0 5.5 5.0 4.5 4.0 3.5 3.0 2.5 2.0 1.5 1.0 0.5 0.0 -0.5
f1 (ppm)

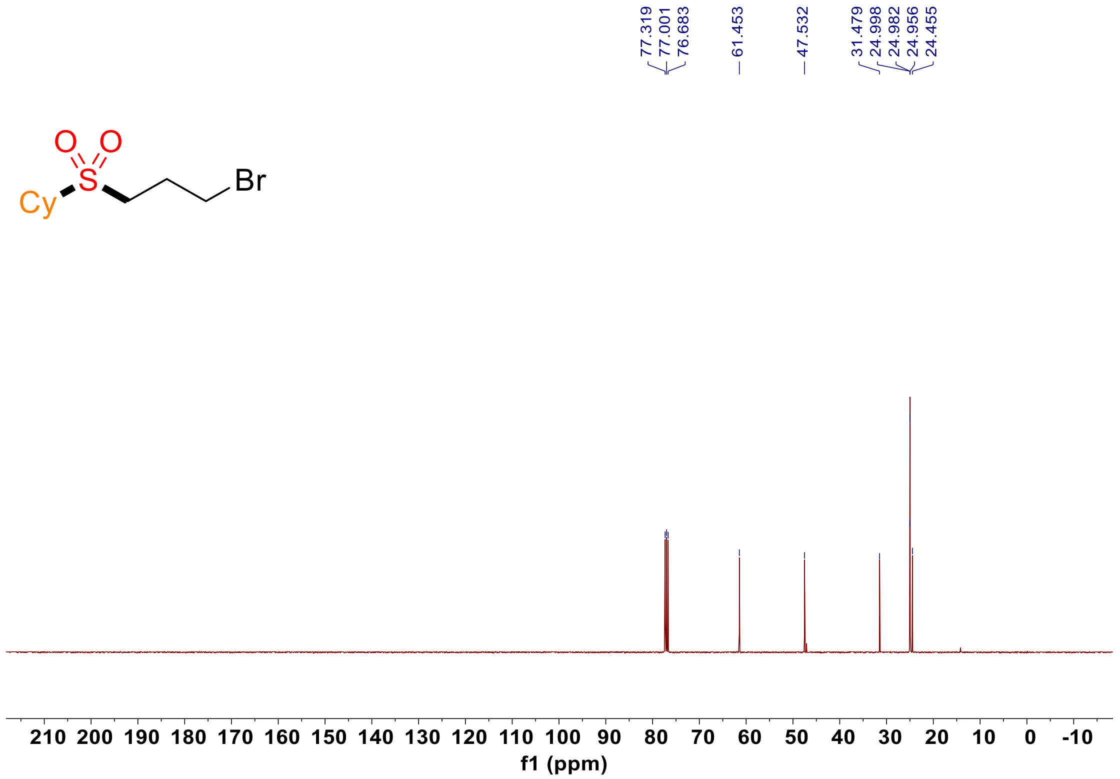
77.319
77.001
76.683
61.453
47.532
31.479
24.998
24.982
24.956
24.455
Cy
S
O
O
Br
210 200 190 180 170 160 150 140 130 120 110 100 90 80 70 60 50 40 30 20 10 0 -10
f1 (ppm)

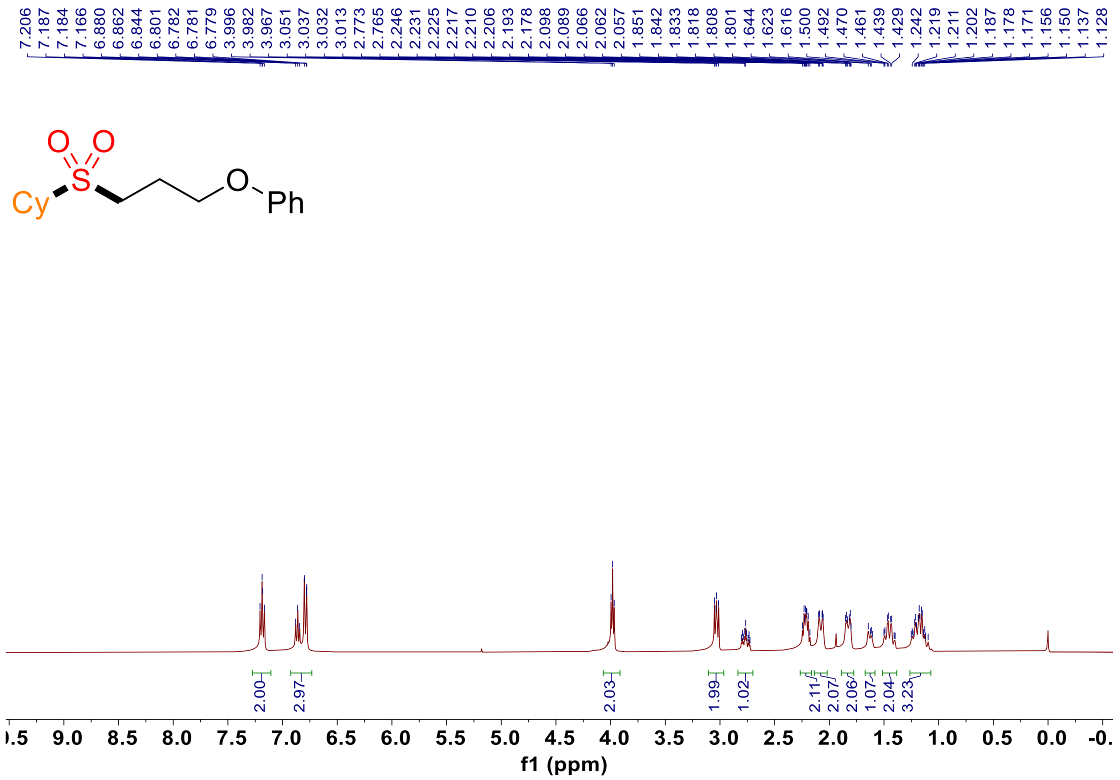
7.206
7.187
7.184
7.166
6.880
6.862
6.844
6.801
6.782
6.781
6.779
3.996
3.982
3.967
3.051
3.037
3.032
3.013
2.773
2.765
2.246
2.231
2.225
2.217
2.210
2.206
2.193
2.178
2.098
2.089
2.066
2.062
2.057
1.851
1.842
1.833
1.818
1.808
1.801
1.644
1.623
1.616
1.500
1.492
1.470
1.461
1.439
1.429
1.242
1.219
1.211
1.202
1.187
1.178
1.171
1.156
1.150
1.137
1.128
Cy
S
O
O
O
Ph
2.00
2.97
2.03
1.99
1.02
2.11
2.07
2.06
1.07
2.04
3.23
f1 (ppm)

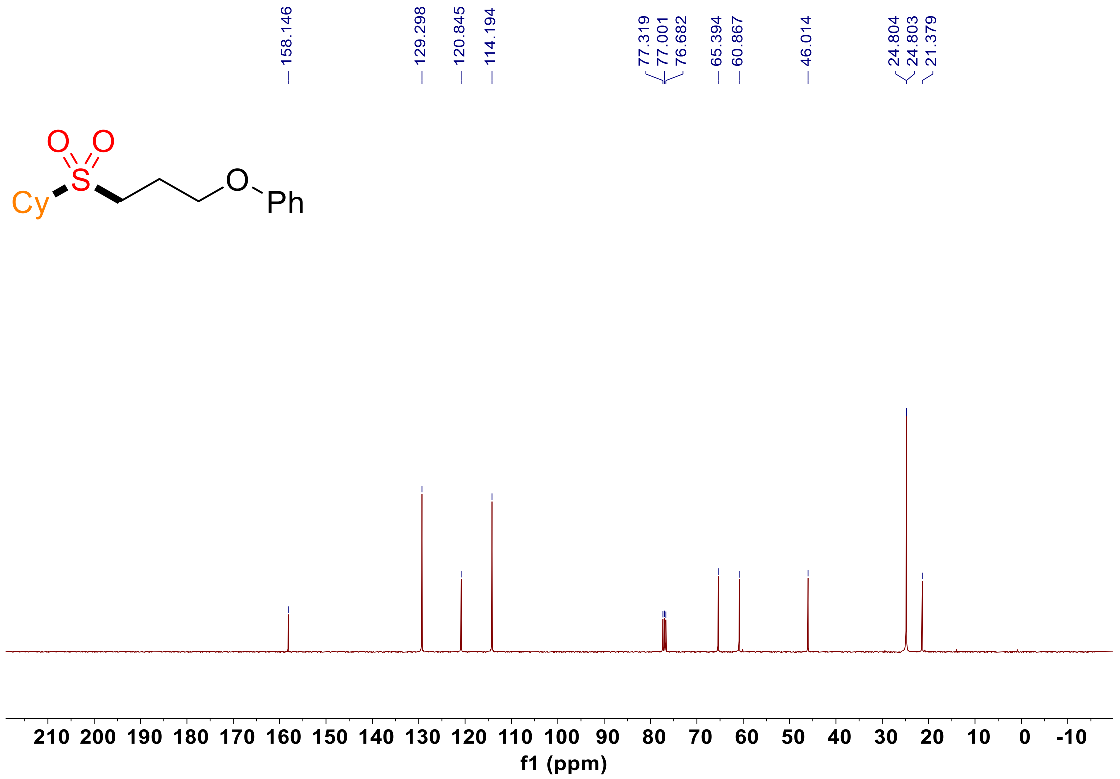
158.146
129.298
120.845
114.194
77.319
77.001
76.682
65.394
60.867
46.014
24.804
24.803
21.379
Cy
S
O
O
O
Ph
f1 (ppm)

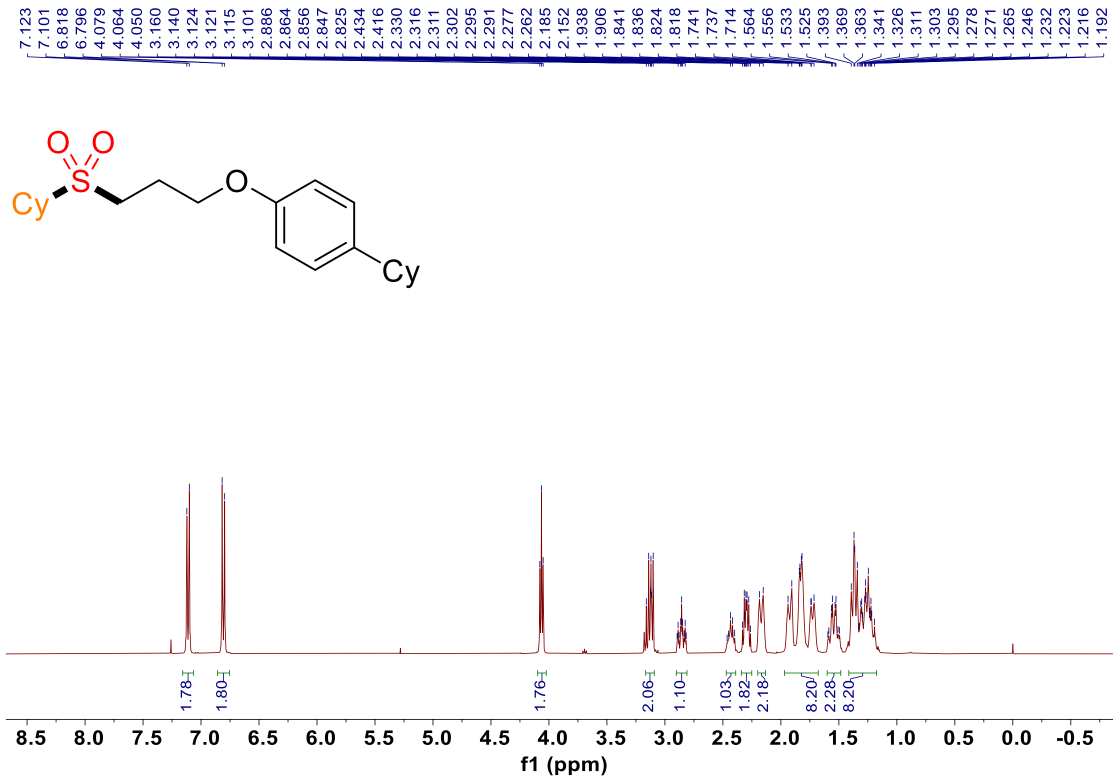
7.123
7.101
6.818
6.796
4.079
4.064
4.050
3.160
3.140
3.124
3.121
3.115
3.101
2.886
2.864
2.856
2.847
2.825
2.434
2.416
2.330
2.316
2.311
2.302
2.295
2.291
2.277
2.262
2.185
2.152
1.938
1.906
1.841
1.836
1.824
1.818
1.741
1.737
1.714
1.564
1.556
1.533
1.525
1.393
1.369
1.363
1.341
1.326
1.311
1.303
1.295
1.278
1.271
1.265
1.246
1.232
1.223
1.216
1.192
O
O
S
Cy
O
Cy
1.78
1.80
1.76
2.06
1.10
1.03
1.82
2.18
8.20
2.28
8.20
8.5 8.0 7.5 7.0 6.5 6.0 5.5 5.0 4.5 4.0 3.5 3.0 2.5 2.0 1.5 1.0 0.5 0.0 -0.5
f1 (ppm)

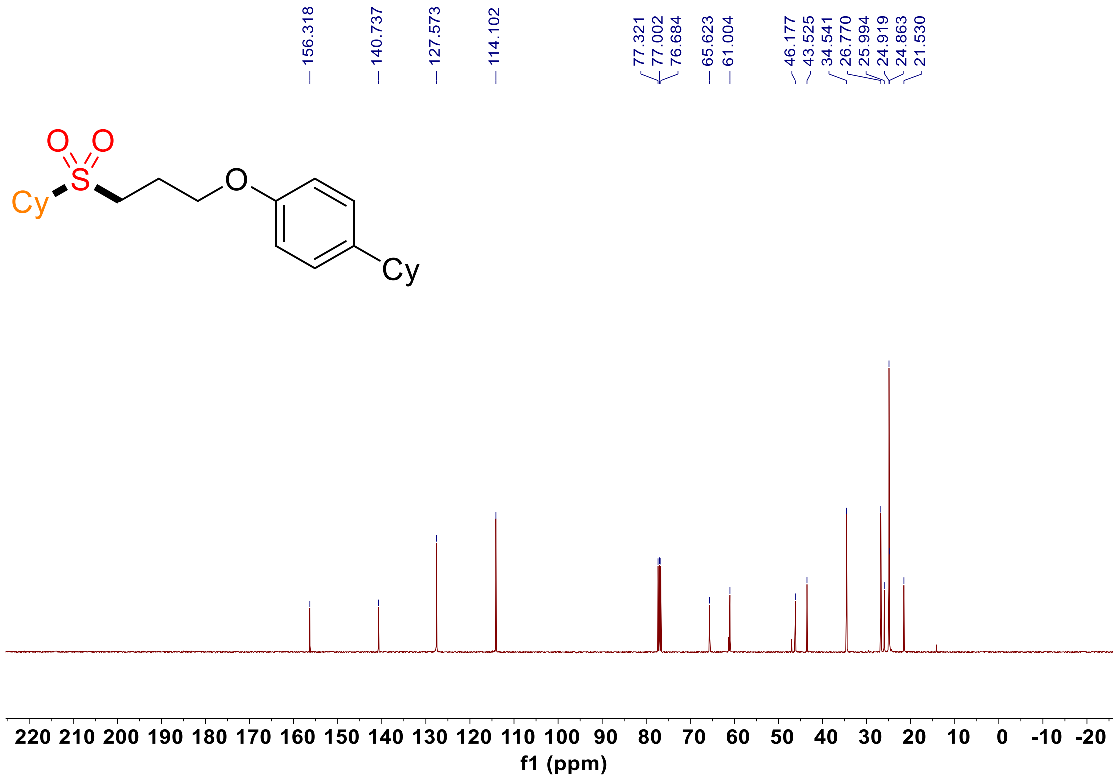
156.318
140.737
127.573
114.102
77.321
77.002
76.684
65.623
61.004
46.177
43.525
34.541
26.770
25.994
24.919
24.863
21.530
O
O
S
Cy
O
Cy
220 210 200 190 180 170 160 150 140 130 120 110 100 90 80 70 60 50 40 30 20 10 0 -10 -20
f1 (ppm)

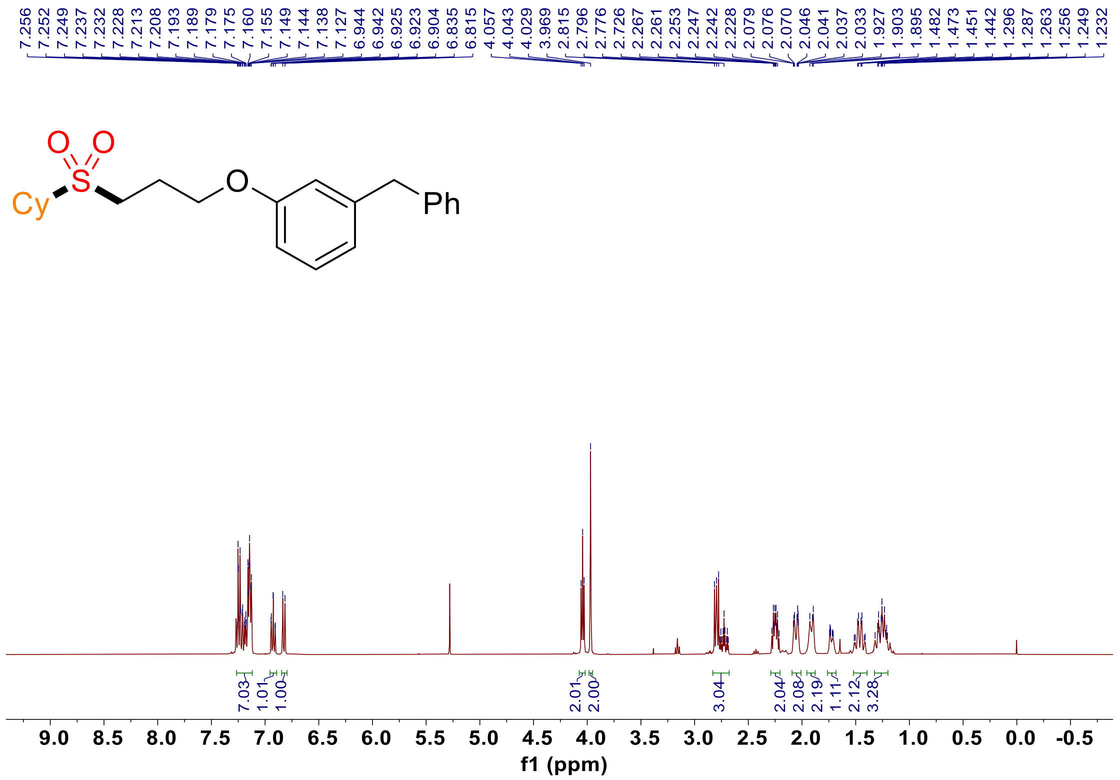
7.256
7.252
7.249
7.237
7.232
7.228
7.213
7.208
7.193
7.189
7.179
7.175
7.160
7.155
7.149
7.144
7.138
7.127
6.944
6.942
6.925
6.923
6.904
6.835
6.815
4.057
4.043
4.029
3.969
2.815
2.796
2.776
2.726
2.267
2.261
2.253
2.247
2.242
2.228
2.079
2.076
2.070
2.046
2.041
2.037
2.033
1.927
1.903
1.895
1.482
1.473
1.451
1.442
1.296
1.287
1.263
1.256
1.249
1.232
Cy
S
O
O
O
Ph
7.03
1.01
1.00
2.01
2.00
3.04
2.04
2.08
2.19
1.11
2.12
3.28
f1 (ppm)

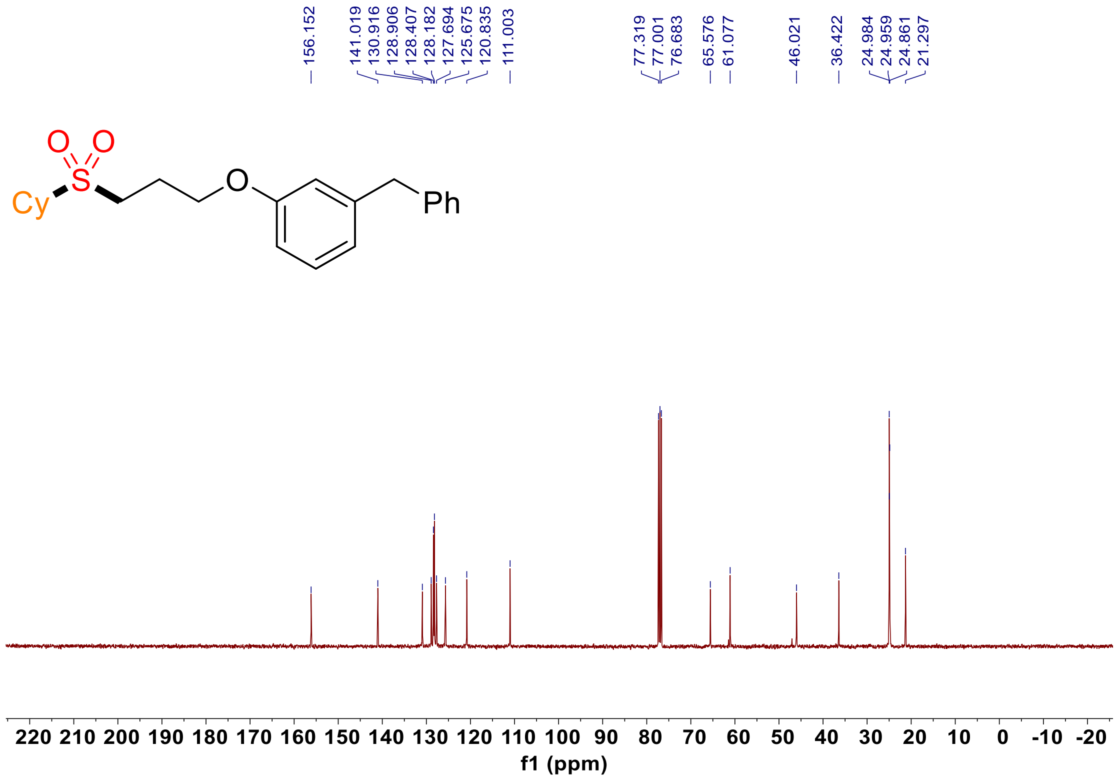
156.152
141.019
130.916
128.906
128.407
128.182
127.694
125.675
120.835
111.003
77.319
77.001
76.683
65.576
61.077
46.021
36.422
24.984
24.959
24.861
21.297
Cy
S
O
O
O
Ph
f1 (ppm)

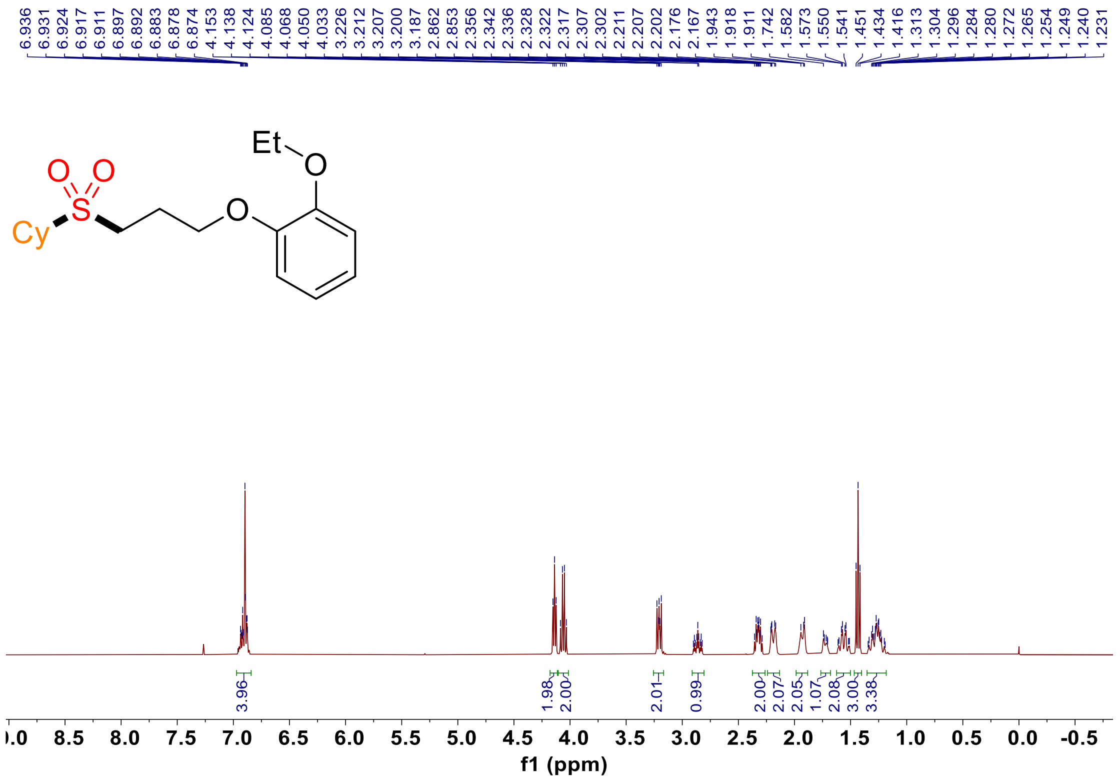
6.936 6.931 6.924 6.917 6.911 6.897 6.892 6.883 6.878 6.874 4.153 4.138 4.124 4.085 4.068 4.050 4.033 3.226 3.212 3.207 3.200 3.187 2.862 2.853 2.356 2.342 2.336 2.328 2.322 2.317 2.307 2.302 2.211 2.207 2.202 2.176 2.167 1.943 1.918 1.911 1.742 1.582 1.573 1.550 1.541 1.451 1.434 1.416 1.313 1.304 1.296 1.284 1.280 1.272 1.265 1.254 1.249 1.240 1.231
Et
O
O
O
S
O
Cy
3.96
1.98
2.00
2.01
0.99
2.00
2.07
2.05
1.07
2.08
3.00
3.38
f1 (ppm)

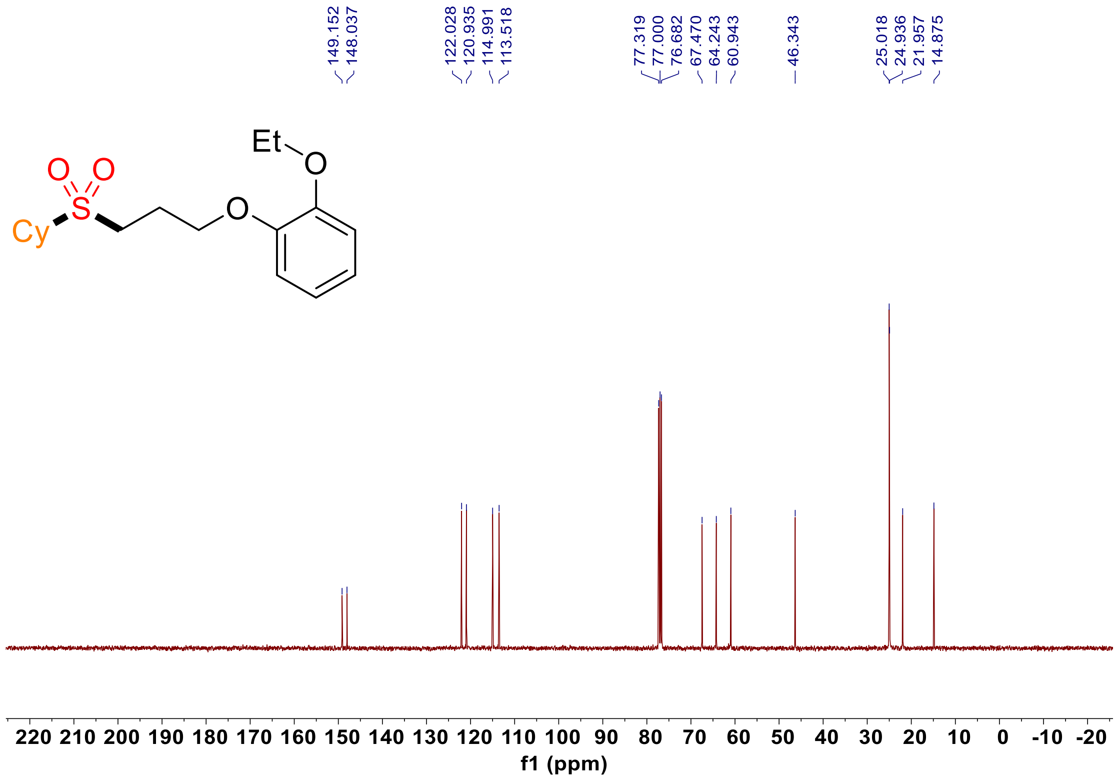
149.152 148.037
122.028 120.935 114.991 113.518
77.319 77.000 76.682 67.470 64.243 60.943
46.343
25.018 24.936 21.957 14.875
Et
O
O
O
S
O
Cy
f1 (ppm)

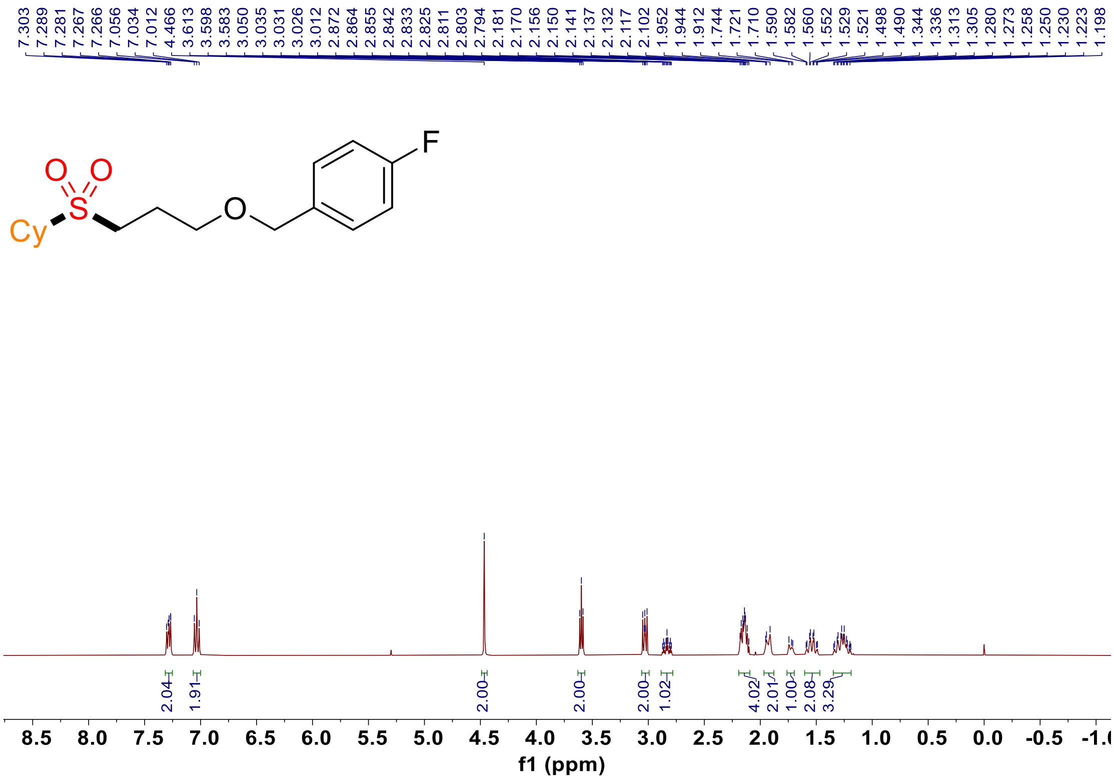
7.303
7.289
7.281
7.267
7.266
7.056
7.034
7.012
4.466
3.613
3.598
3.583
3.050
3.035
3.031
3.026
3.012
2.872
2.864
2.855
2.842
2.833
2.825
2.811
2.803
2.794
2.181
2.170
2.156
2.150
2.141
2.137
2.132
2.117
2.102
1.952
1.944
1.912
1.744
1.721
1.710
1.590
1.582
1.560
1.552
1.529
1.521
1.498
1.490
1.344
1.336
1.313
1.305
1.280
1.273
1.258
1.250
1.230
1.223
1.198
Cy
S
O
O
O
F
2.04
1.91
2.00
2.00
2.00
1.02
4.02
2.01
1.00
2.08
3.29
8.5 8.0 7.5 7.0 6.5 6.0 5.5 5.0 4.5 4.0 3.5 3.0 2.5 2.0 1.5 1.0 0.5 0.0 -0.5
f1 (ppm)

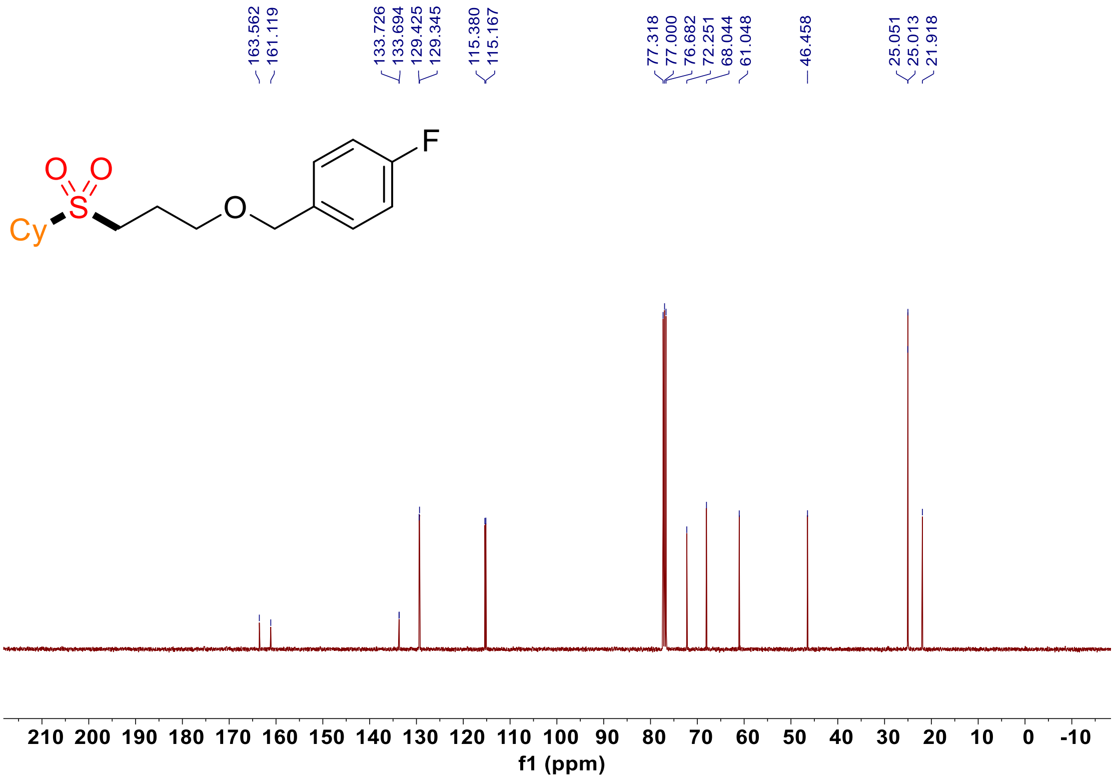
163.562
161.119
133.726
133.694
129.425
129.345
115.380
115.167
77.318
77.000
76.682
72.251
68.044
61.048
46.458
25.051
25.013
21.918
Cy
S
O
O
O
F
210 200 190 180 170 160 150 140 130 120 110 100 90 80 70 60 50 40 30 20 10 0 -10
f1 (ppm)

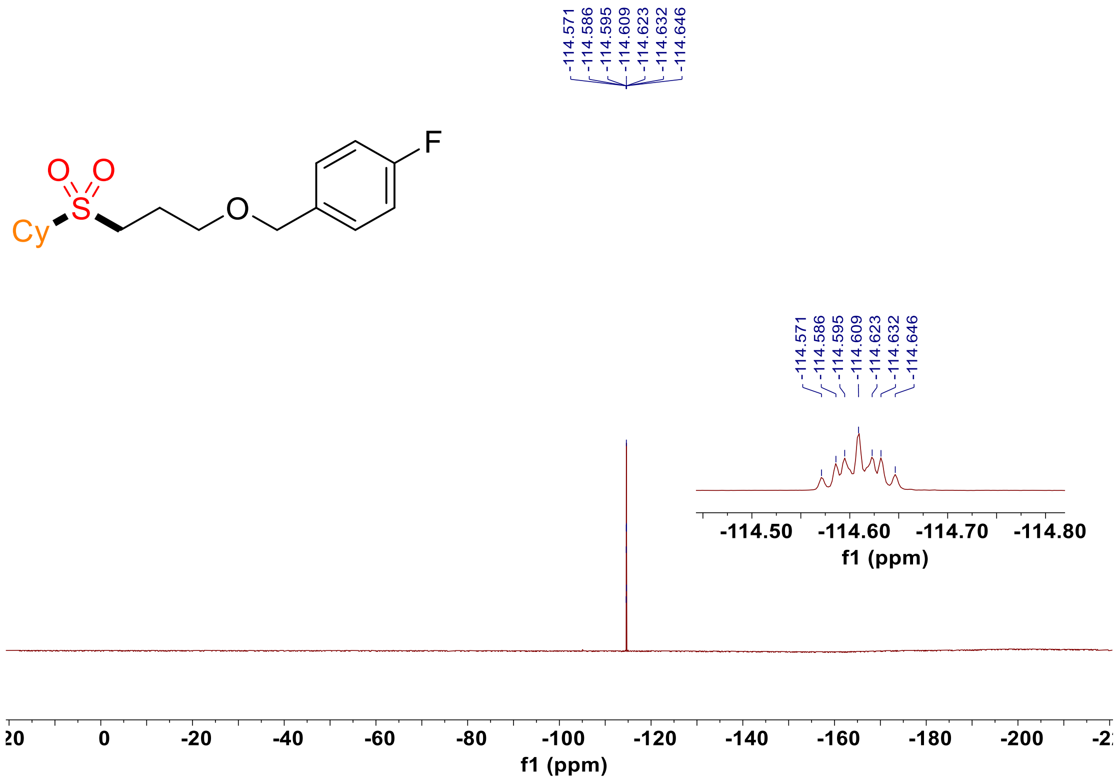

-114.571
-114.586
-114.595
-114.609
-114.623
-114.632
-114.646
F
O
O
S
Cy
O
-114.50
-114.60
-114.70
-114.80
f1 (ppm)
20
0
-20
-40
-60
-80
-100
-120
-140
-160
-180
-200
f1 (ppm)

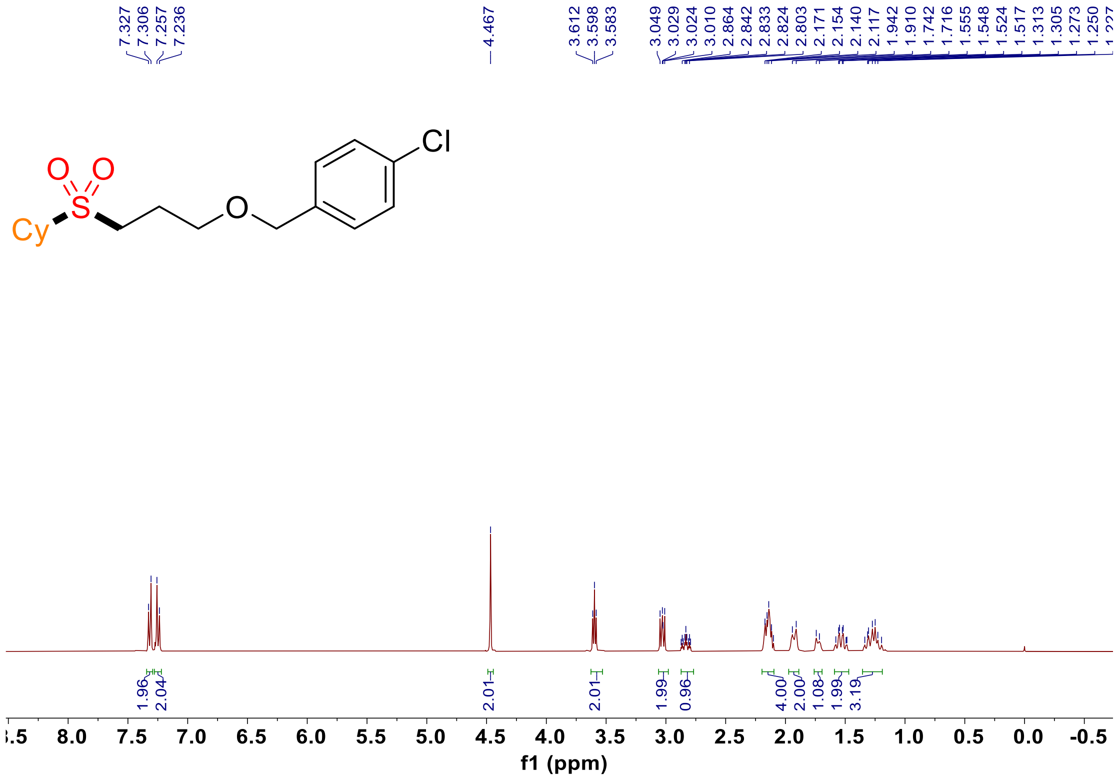

7.327
7.306
7.257
7.236
4.467
3.612
3.598
3.583
3.049
3.029
3.024
3.010
2.864
2.842
2.833
2.824
2.803
2.171
2.154
2.140
2.117
1.942
1.910
1.742
1.716
1.555
1.548
1.524
1.517
1.313
1.305
1.273
1.250
Cl
O
O
S
Cy
O
1.96
2.04
2.01
2.01
1.99
0.96
4.00
2.00
1.08
1.99
3.19
8.0
7.5
7.0
6.5
6.0
5.5
5.0
4.5
4.0
3.5
3.0
2.5
2.0
1.5
1.0
0.5
0.0
-0.5
f1 (ppm)

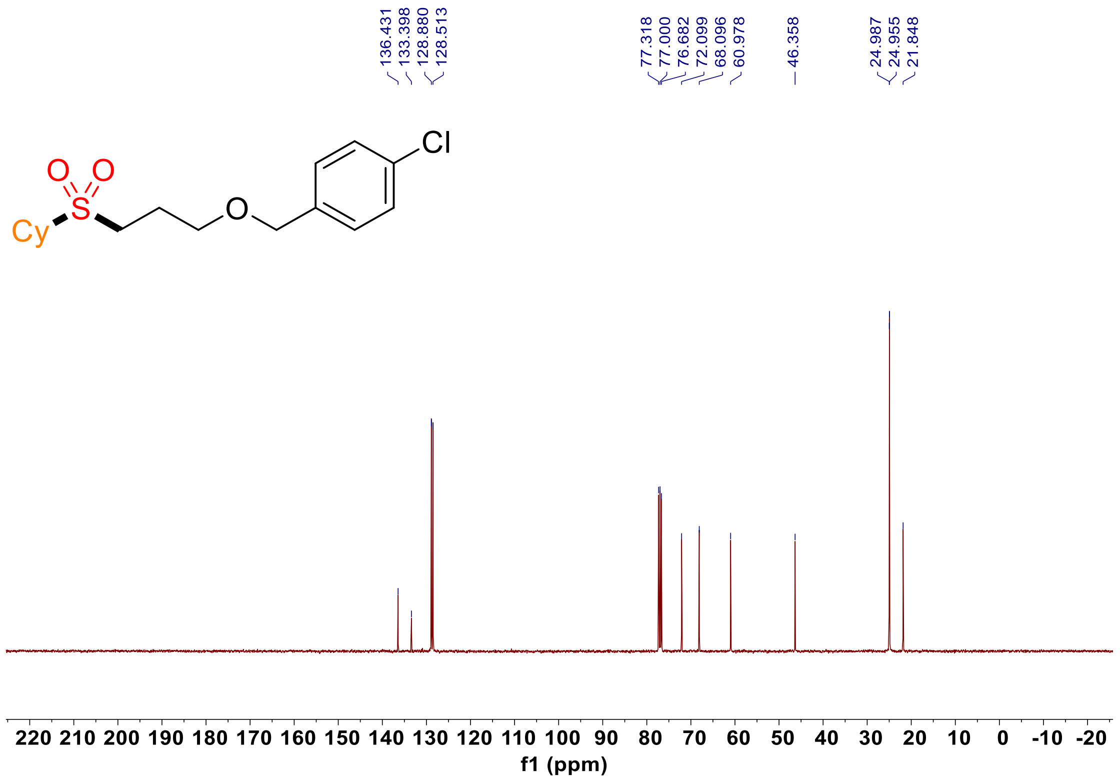
136.431
133.398
128.880
128.513
77.318
77.000
76.682
72.099
68.096
60.978
46.358
24.987
24.955
21.848
Cl
O
O
S
Cy
O
220 210 200 190 180 170 160 150 140 130 120 110 100 90 80 70 60 50 40 30 20 10 0 -10 -20
f1 (ppm)

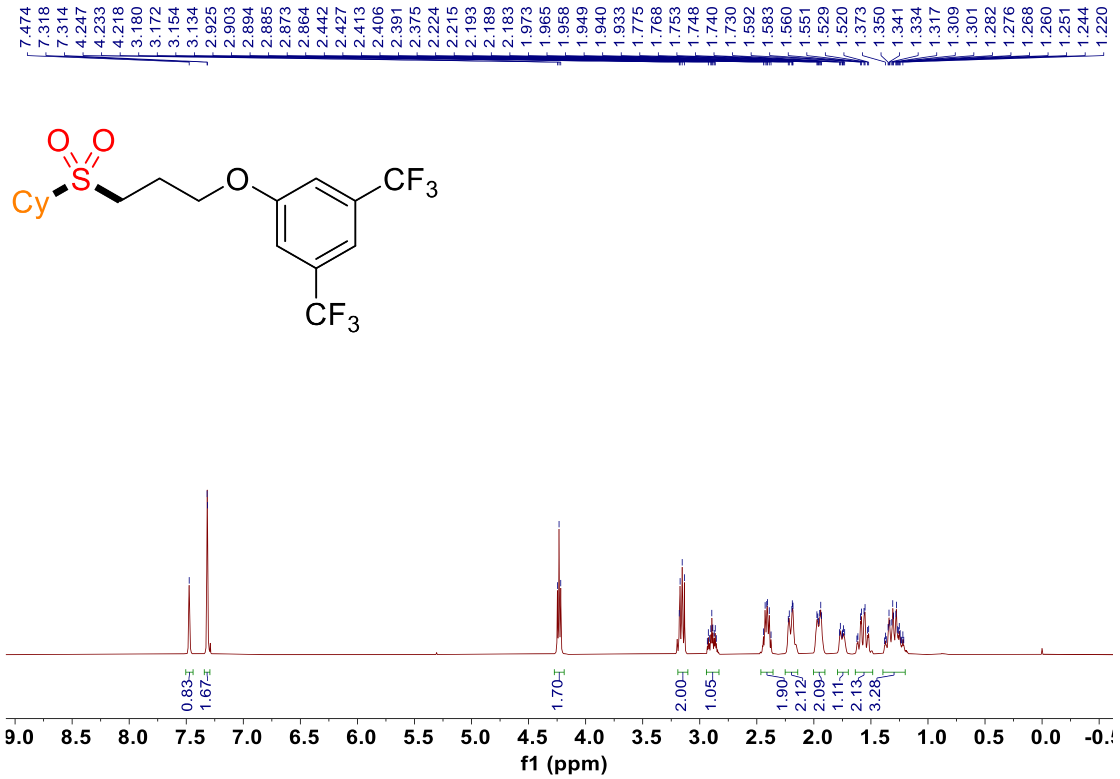
7.474
7.318
7.314
4.247
4.233
4.218
3.180
3.172
3.154
3.134
2.925
2.903
2.894
2.885
2.873
2.864
2.442
2.427
2.413
2.406
2.391
2.375
2.224
2.215
2.193
2.189
2.183
1.973
1.965
1.958
1.949
1.940
1.933
1.775
1.768
1.753
1.748
1.740
1.730
1.592
1.583
1.560
1.551
1.529
1.520
1.373
1.350
1.341
1.334
1.317
1.309
1.301
1.282
1.276
1.268
1.260
1.251
1.244
1.220
O
O
S
Cy
O
CF3
CF3
0.83
1.67
1.70
2.00
1.05
1.90
2.12
2.09
1.11
2.13
3.28
9.0 8.5 8.0 7.5 7.0 6.5 6.0 5.5 5.0 4.5 4.0 3.5 3.0 2.5 2.0 1.5 1.0 0.5 0.0
f1 (ppm)

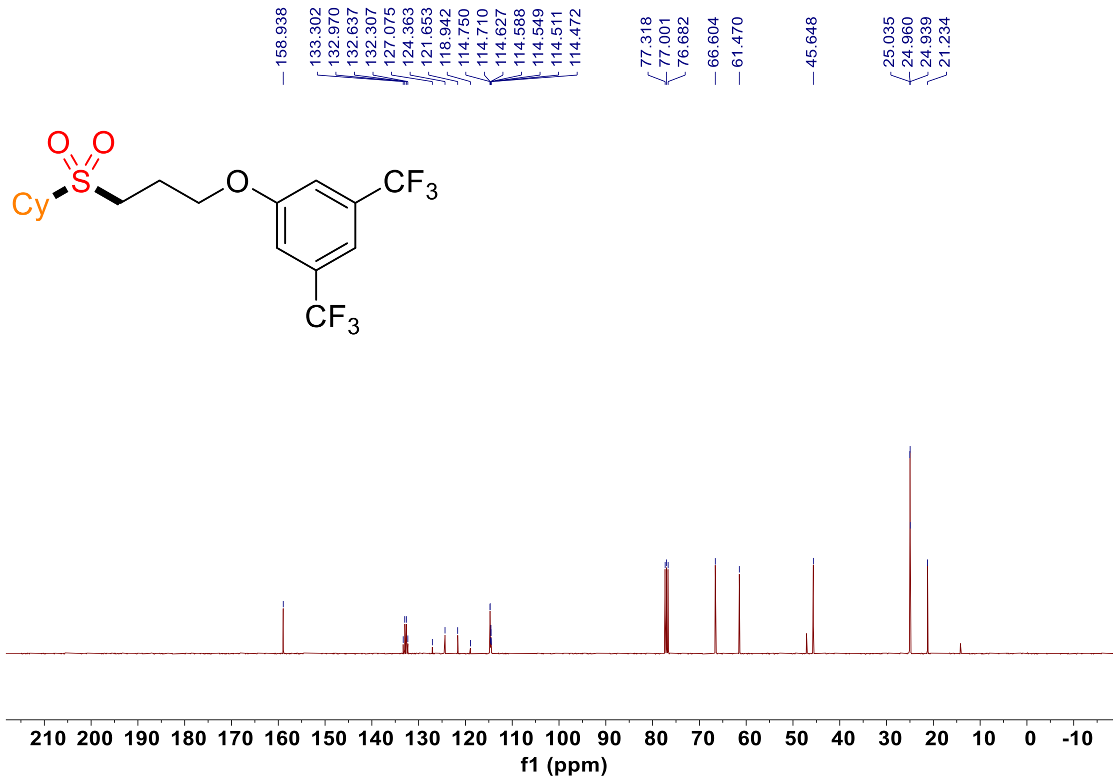
158.938
133.302
132.970
132.637
132.307
127.075
124.363
121.653
118.942
114.750
114.710
114.627
114.588
114.549
114.511
114.472
77.318
77.001
76.682
66.604
61.470
45.648
25.035
24.960
24.939
21.234
O
O
S
Cy
O
CF_3
CF_3
210 200 190 180 170 160 150 140 130 120 110 100 90 80 70 60 50 40 30 20 10 0 -10
f1 (ppm)

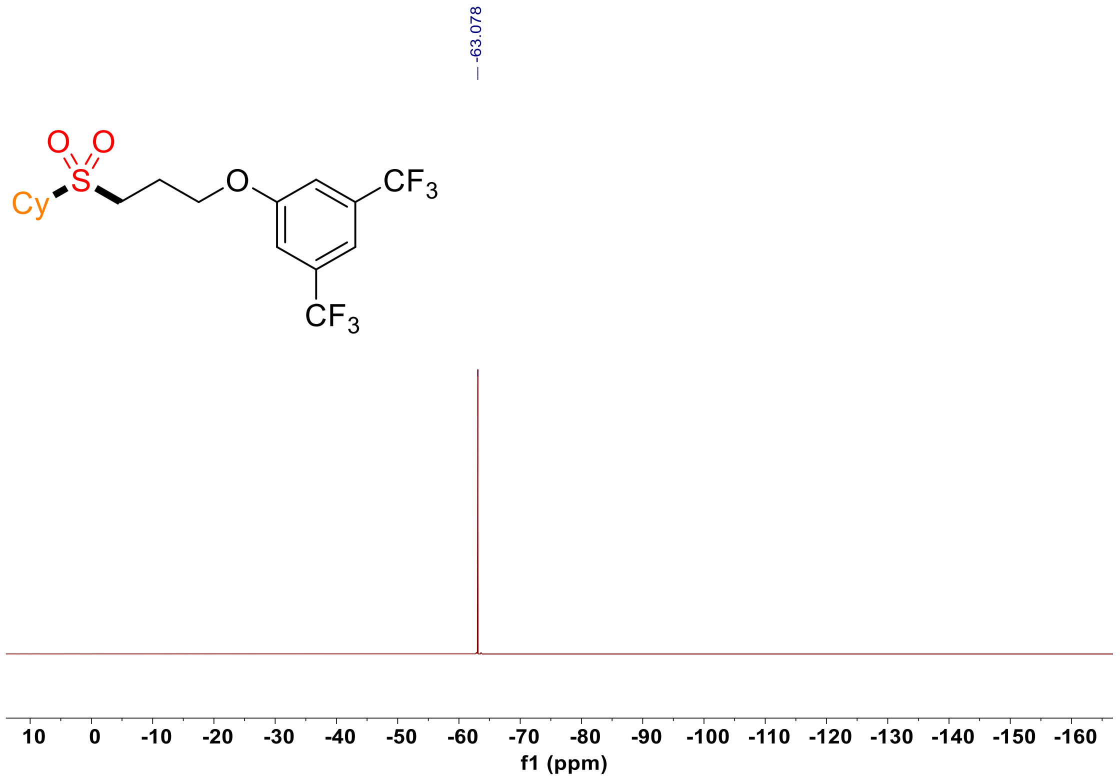
-63.078
O
O
S
Cy
O
CF_3
CF_3
10 0 -10 -20 -30 -40 -50 -60 -70 -80 -90 -100 -110 -120 -130 -140 -150 -160
f1 (ppm)

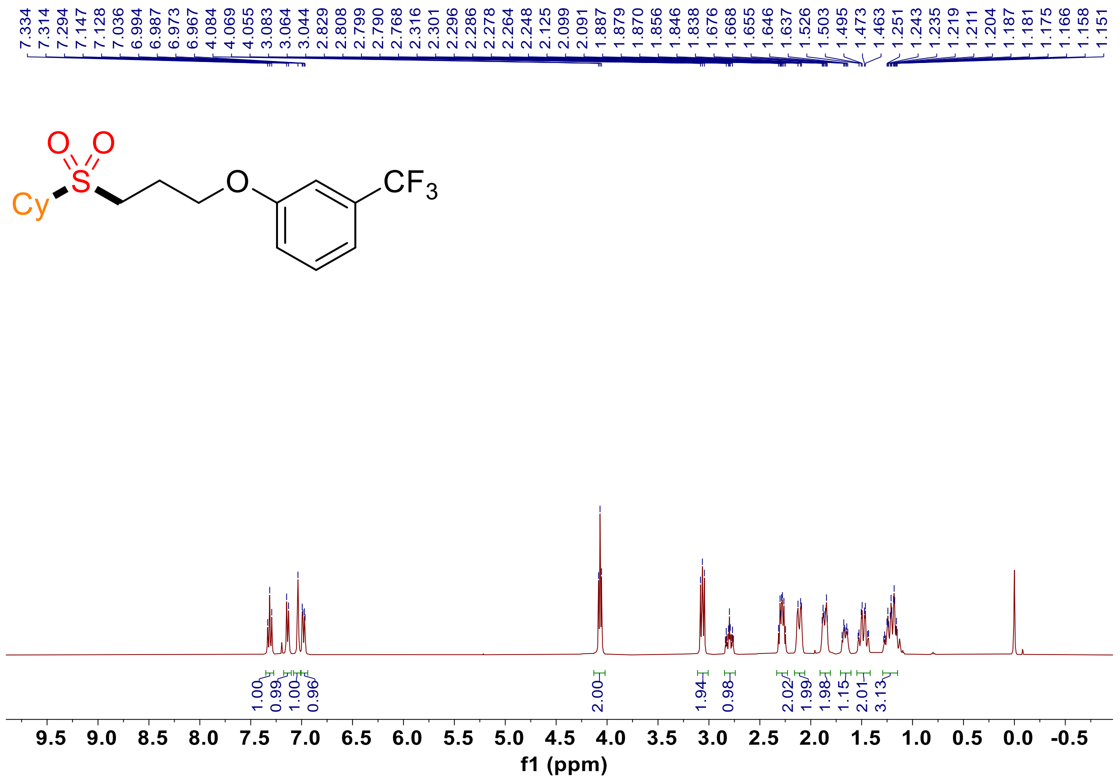

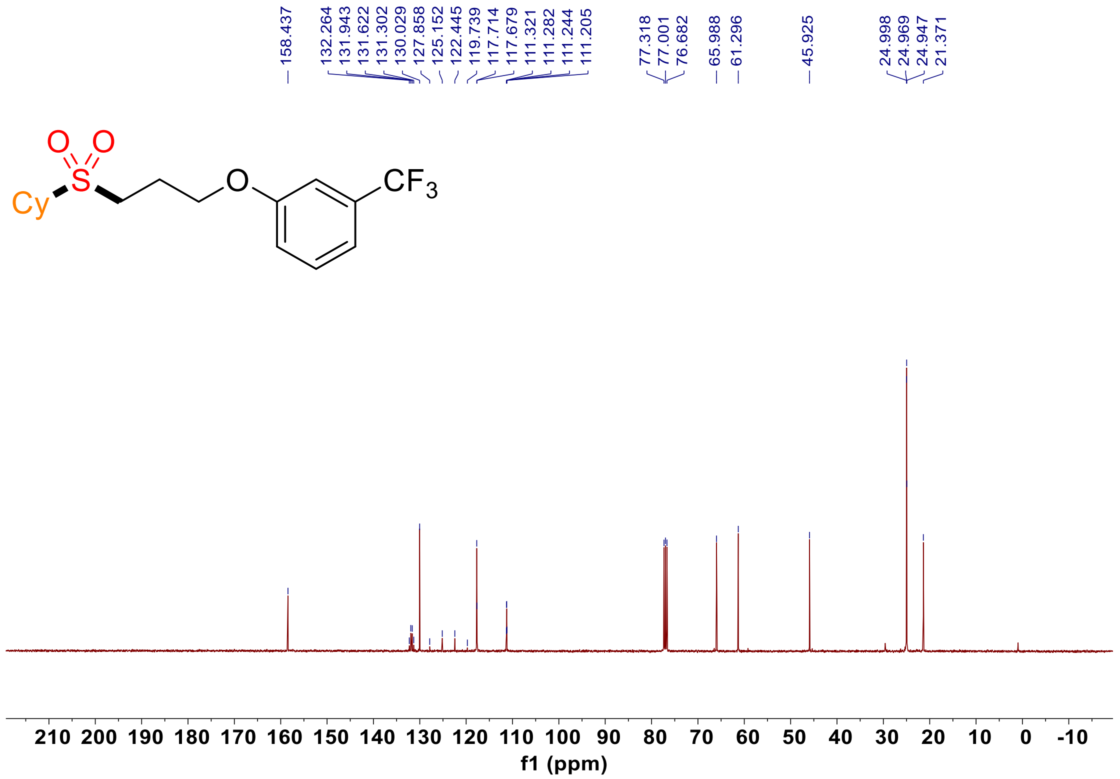

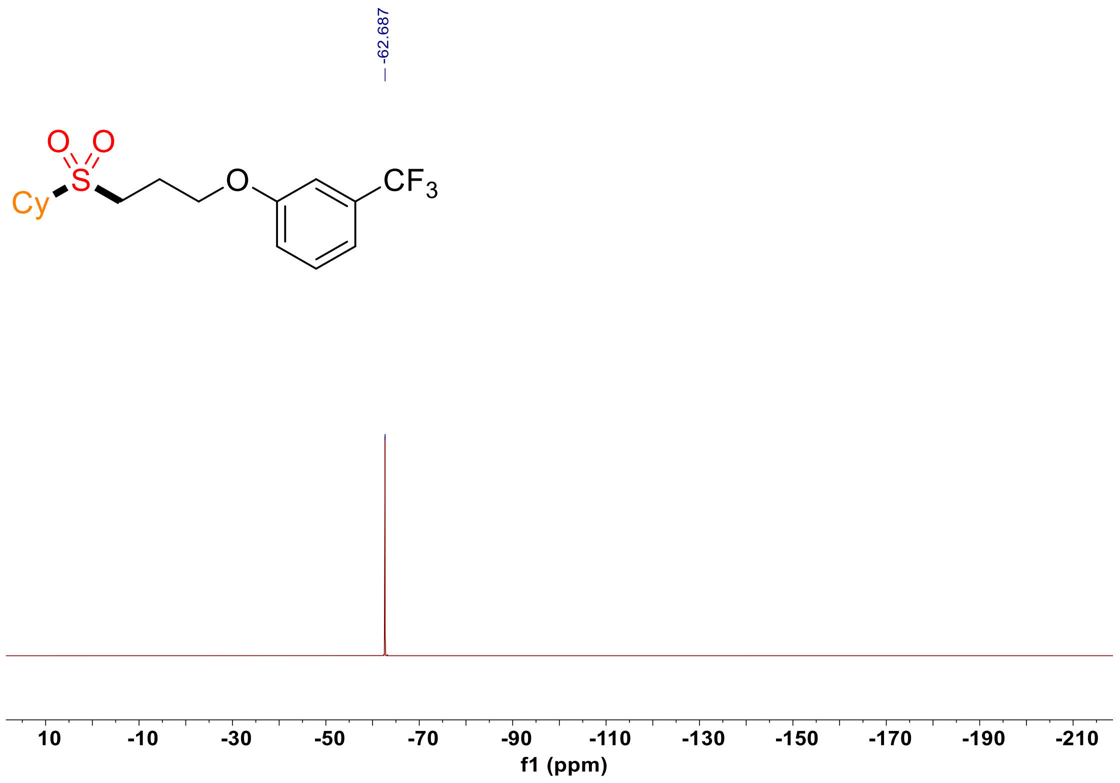
-62.687
O O
S
Cy
O
CF3
10 -10 -30 -50 -70 -90 -110 -130 -150 -170 -190 -210
f1 (ppm)

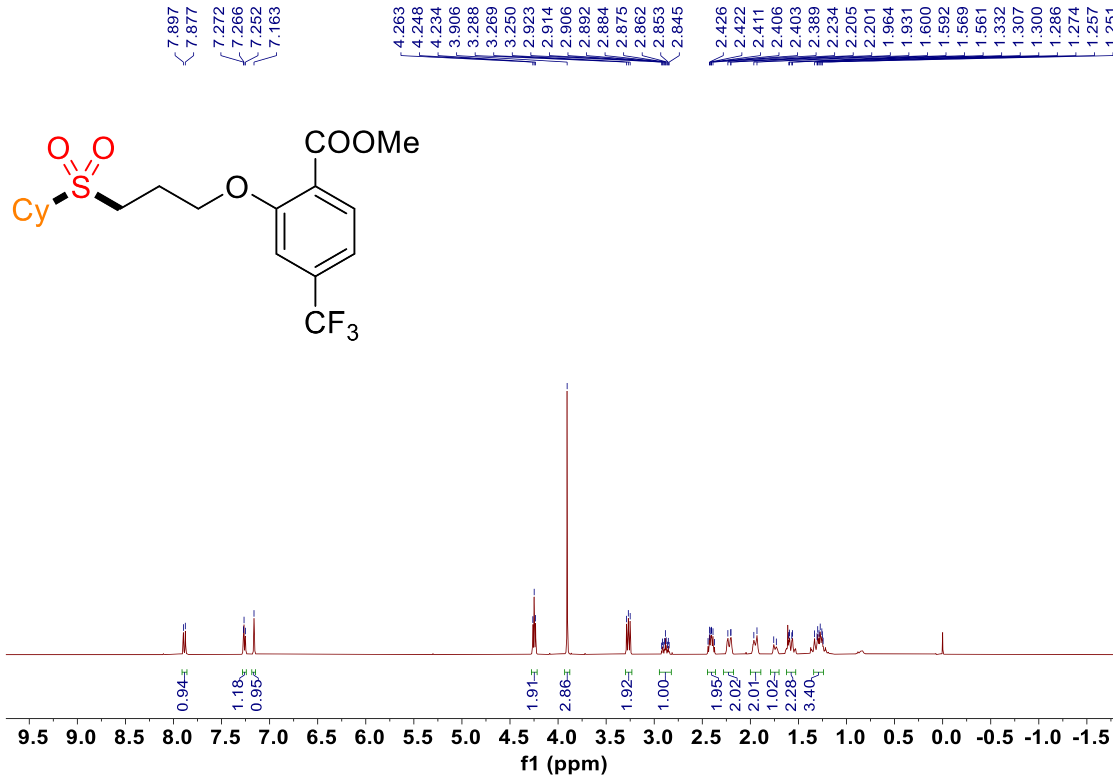
7.897 7.877 7.272 7.266 7.252 7.163
4.263 4.248 4.234 3.906 3.288 3.269 3.250 2.923 2.914 2.906 2.892 2.884 2.875 2.862 2.853 2.845
2.426 2.422 2.411 2.406 2.403 2.389 2.234 2.205 2.201 1.964 1.931 1.600 1.592 1.569 1.561 1.332 1.307 1.300 1.286 1.274 1.257
COOMe
O O
S
Cy
O
CF3
0.94 1.18 0.95 1.91 2.86 1.92 1.00 1.95 2.02 2.01 1.02 2.28 3.40
9.5 9.0 8.5 8.0 7.5 7.0 6.5 6.0 5.5 5.0 4.5 4.0 3.5 3.0 2.5 2.0 1.5 1.0 0.5 0.0 -0.5 -1.0 -1.5
f1 (ppm)

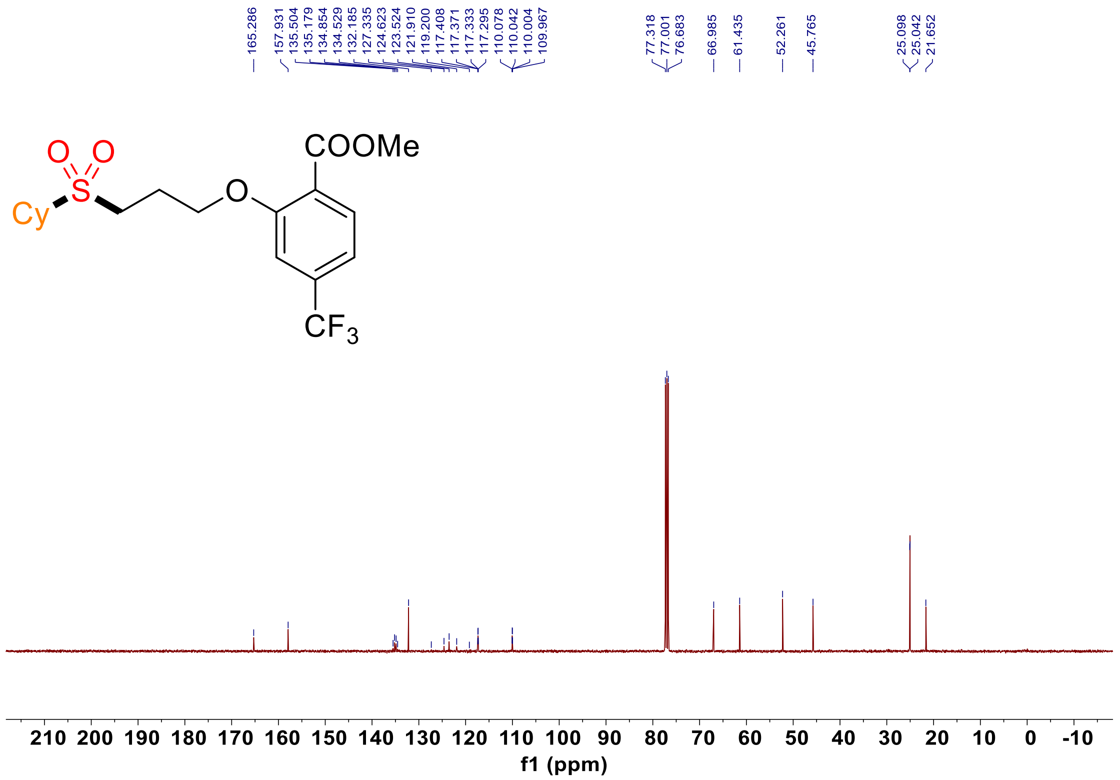
165.286
157.931
135.504
135.179
134.854
134.529
132.185
127.335
124.623
123.524
121.910
119.200
117.408
117.371
117.333
117.295
110.078
110.042
110.004
109.967
77.318
77.001
76.683
66.985
61.435
52.261
45.765
25.098
25.042
21.652
COOMe
O
O
S
Cy
O
CF3
210 200 190 180 170 160 150 140 130 120 110 100 90 80 70 60 50 40 30 20 10 0 -10
f1 (ppm)

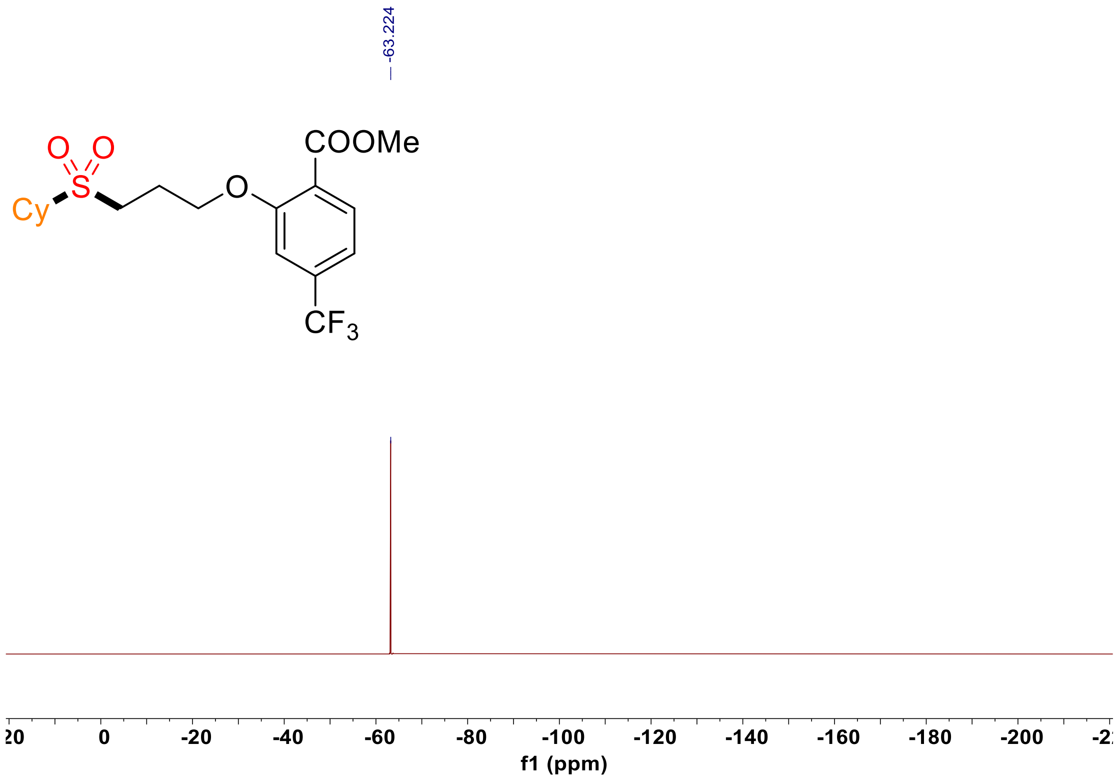
-63.224
COOMe
O
O
S
Cy
O
CF3
20 0 -20 -40 -60 -80 -100 -120 -140 -160 -180 -200
f1 (ppm)

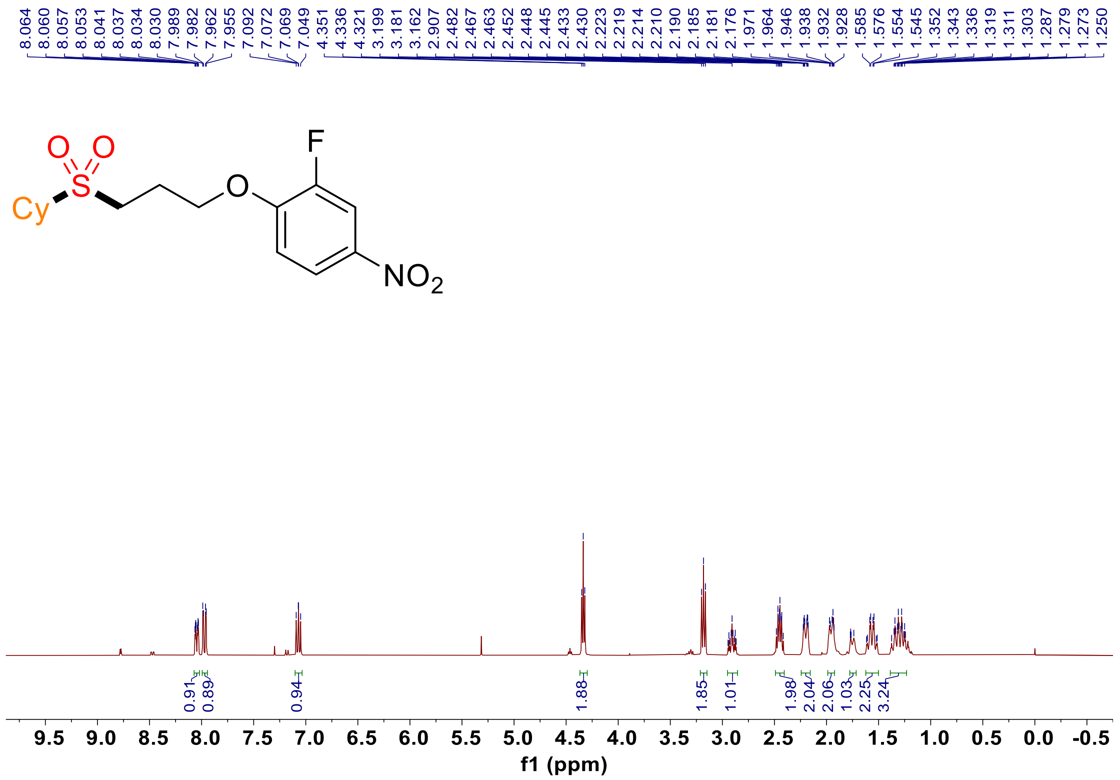

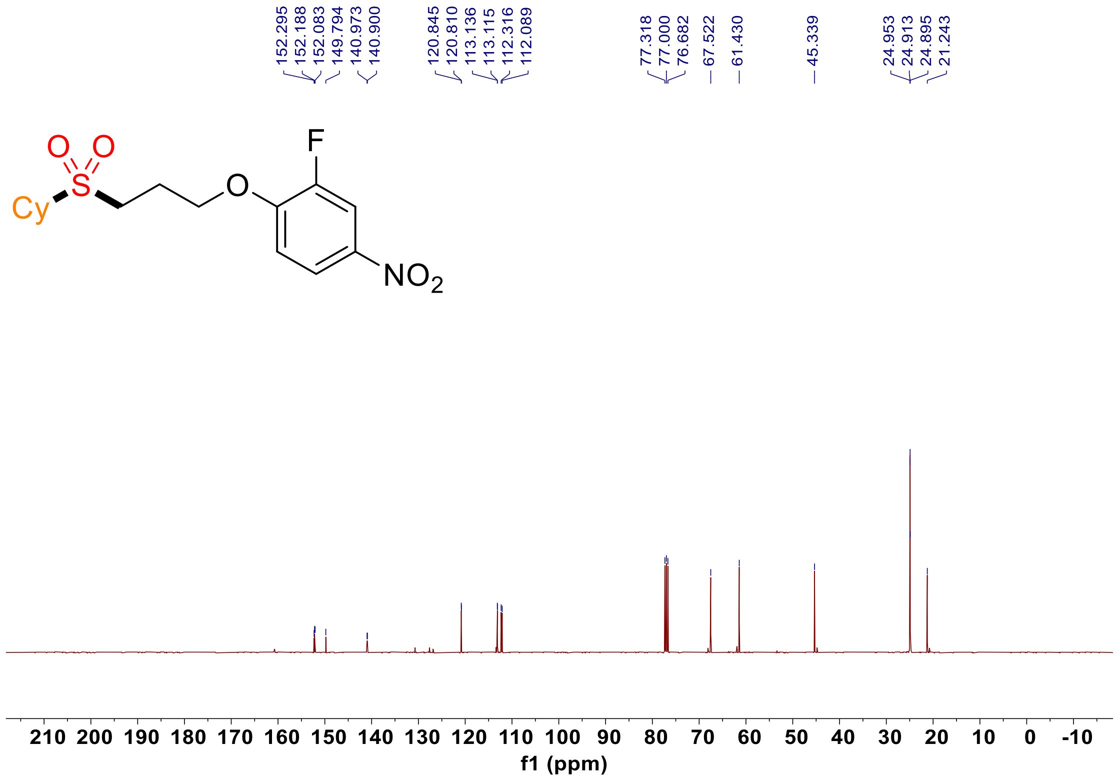

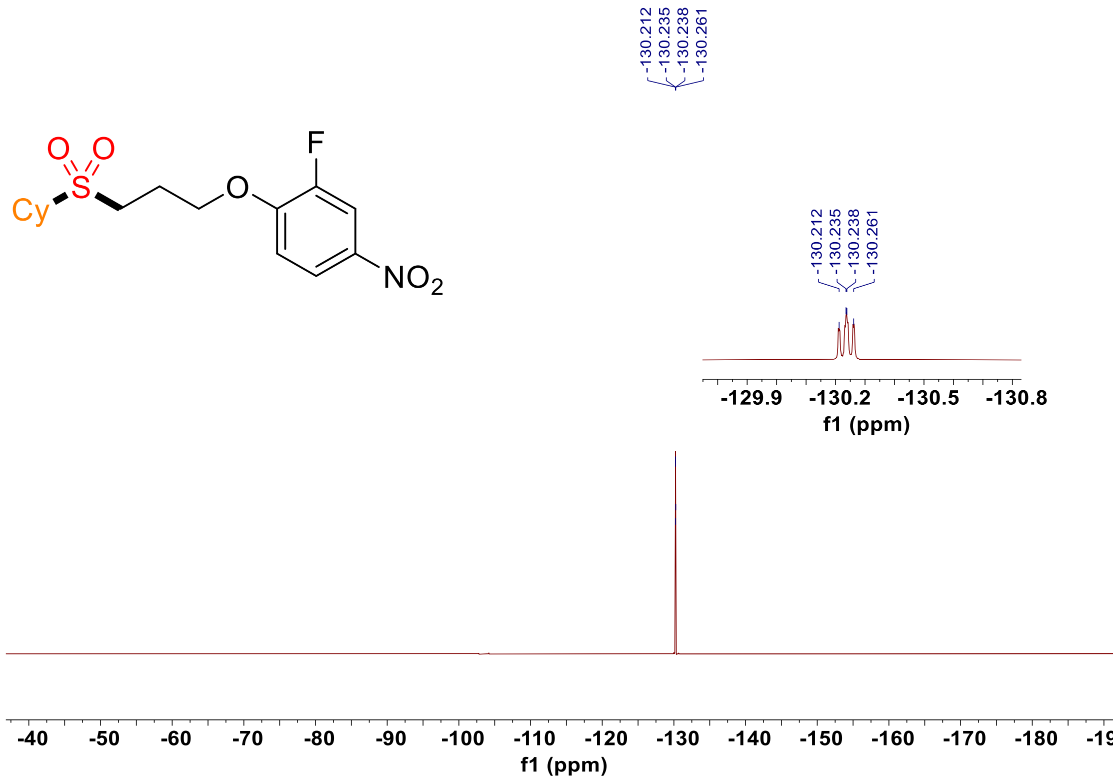

-130.212
-130.235
-130.238
-130.261
Cy
S
O
O
O
F
NO2
-129.9
-130.2
-130.5
-130.8
f1 (ppm)
-40
-50
-60
-70
-80
-90
-100
-110
-120
-130
-140
-150
-160
-170
-180
f1 (ppm)

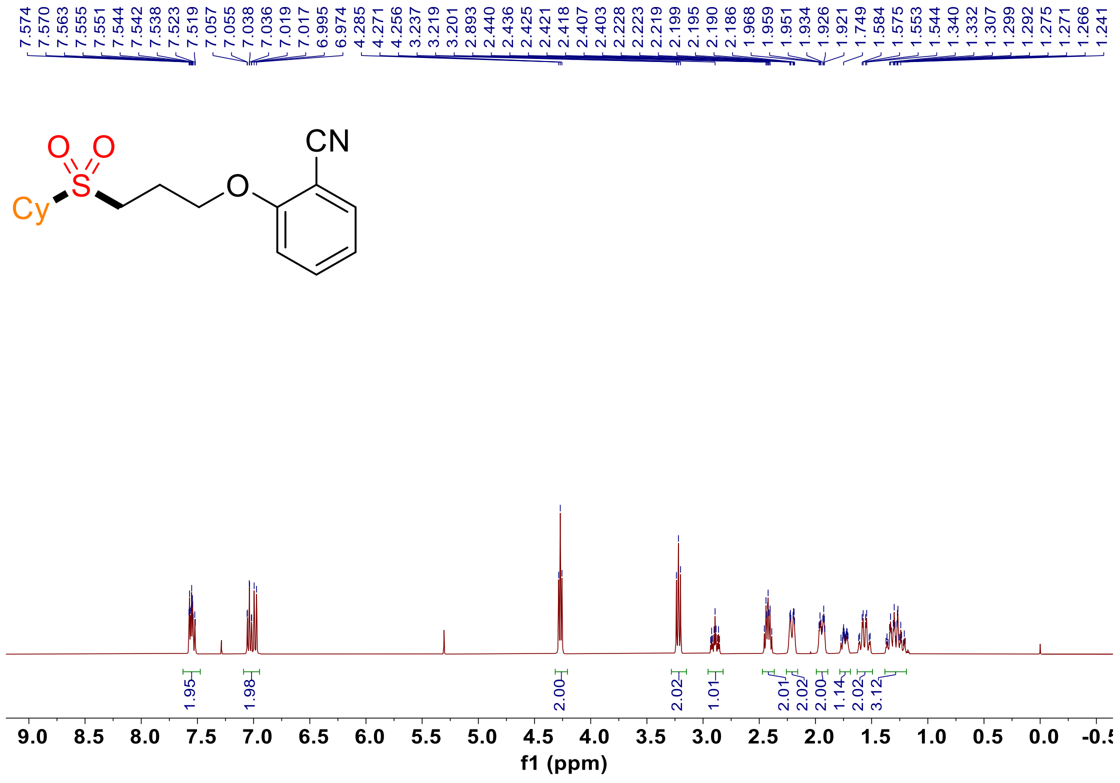

7.574
7.570
7.563
7.555
7.551
7.544
7.542
7.538
7.523
7.519
7.057
7.055
7.038
7.036
7.019
7.017
6.995
6.974
4.285
4.271
4.256
3.237
3.219
3.201
2.893
2.440
2.436
2.425
2.421
2.418
2.407
2.403
2.228
2.223
2.219
2.199
2.195
2.190
2.186
1.968
1.959
1.951
1.934
1.926
1.921
1.749
1.584
1.575
1.553
1.544
1.340
1.332
1.307
1.299
1.292
1.275
1.271
1.266
1.241
Cy
S
O
O
O
CN
1.95
1.98
2.00
2.02
1.01
2.01
2.02
2.00
1.14
2.02
3.12
9.0
8.5
8.0
7.5
7.0
6.5
6.0
5.5
5.0
4.5
4.0
3.5
3.0
2.5
2.0
1.5
1.0
0.5
0.0
f1 (ppm)

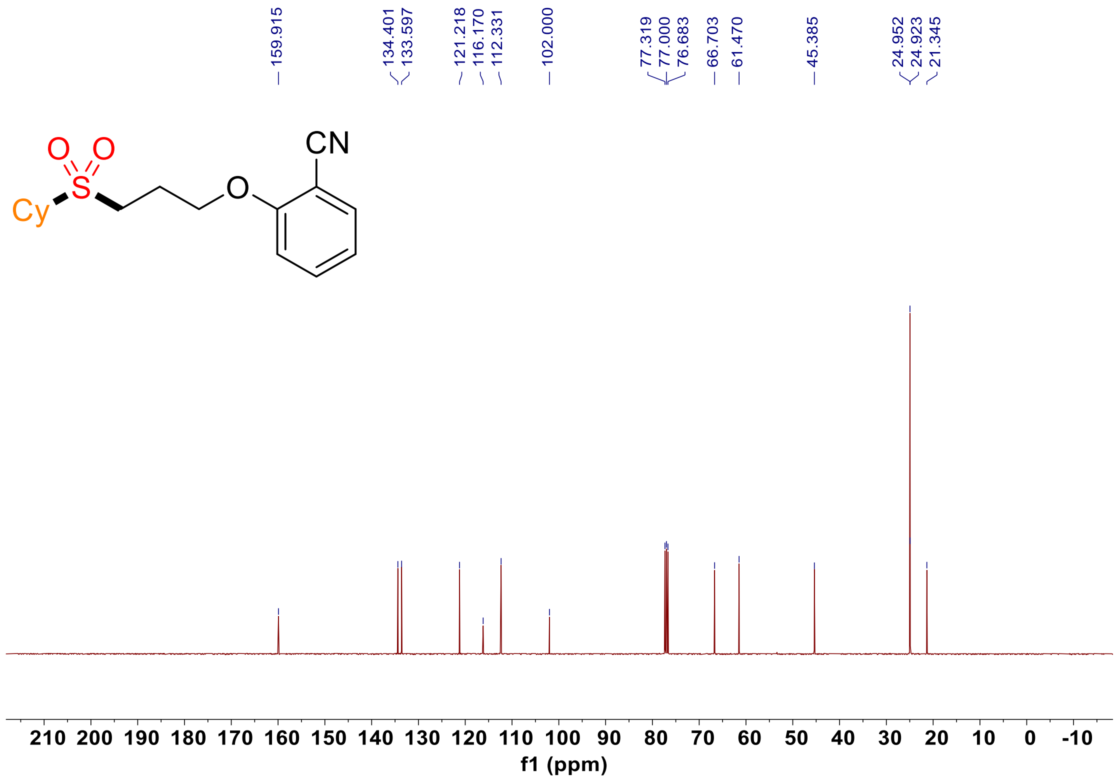

159.915
134.401
133.597
121.218
116.170
112.331
102.000
77.319
77.000
76.683
66.703
61.470
45.385
24.952
24.923
21.345
CN
O
O
S
O
Cy
210 200 190 180 170 160 150 140 130 120 110 100 90 80 70 60 50 40 30 20 10 0 -10
f1 (ppm)

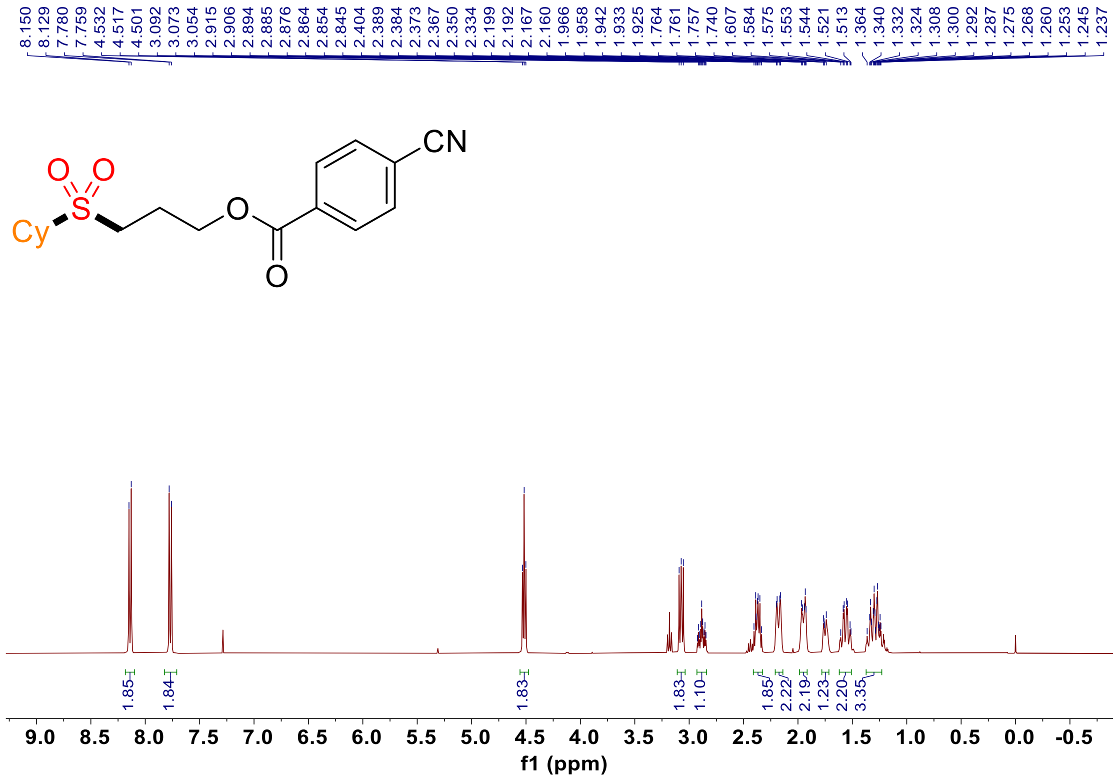

8.150
8.129
7.780
7.759
4.532
4.517
4.501
3.092
3.073
3.054
2.915
2.906
2.894
2.885
2.876
2.864
2.854
2.845
2.404
2.389
2.384
2.373
2.367
2.350
2.334
2.199
2.192
2.167
2.160
1.966
1.958
1.942
1.933
1.925
1.764
1.761
1.757
1.740
1.607
1.584
1.575
1.553
1.544
1.521
1.513
1.364
1.340
1.332
1.324
1.308
1.300
1.292
1.287
1.275
1.268
1.260
1.253
1.245
1.237
CN
O
O
S
O
Cy
O
1.85
1.84
1.83
1.83
1.10
1.85
2.22
2.19
1.23
2.20
3.35
9.0 8.5 8.0 7.5 7.0 6.5 6.0 5.5 5.0 4.5 4.0 3.5 3.0 2.5 2.0 1.5 1.0 0.5 0.0 -0.5
f1 (ppm)

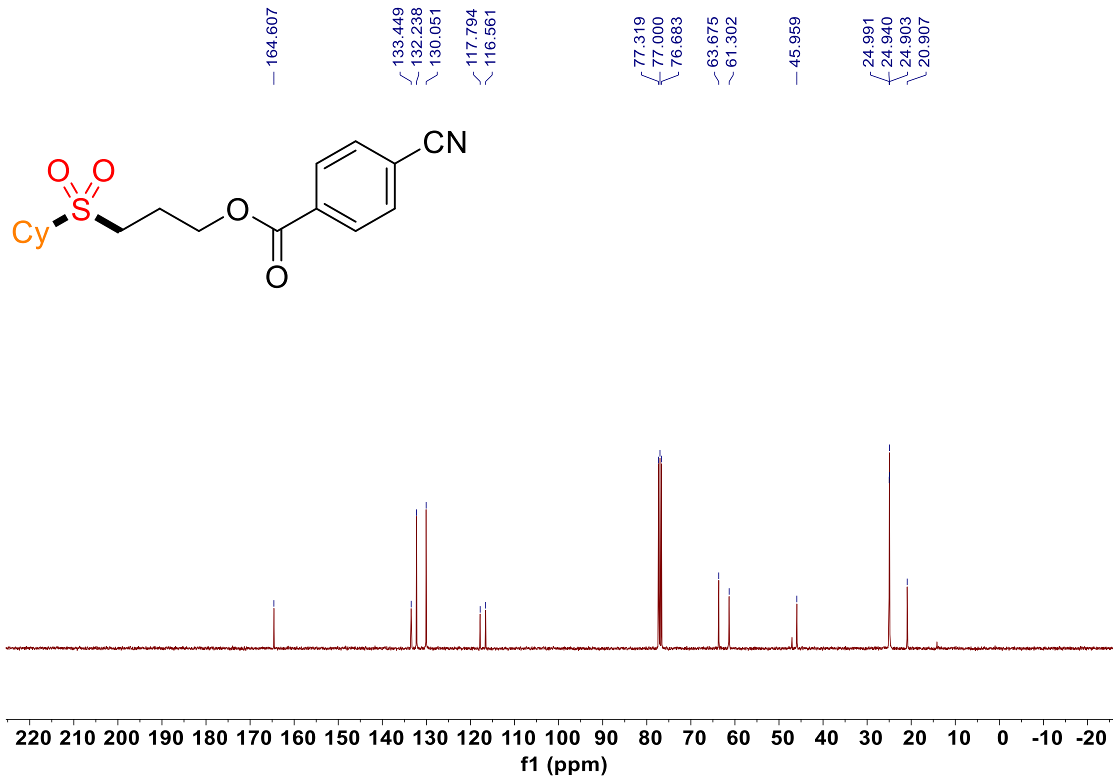
164.607
133.449
132.238
130.051
117.794
116.561
77.319
77.000
76.683
63.675
61.302
45.959
24.991
24.940
24.903
20.907
CN
O
O
S
O
O
O
Cy
220 210 200 190 180 170 160 150 140 130 120 110 100 90 80 70 60 50 40 30 20 10 0 -10 -20
f1 (ppm)

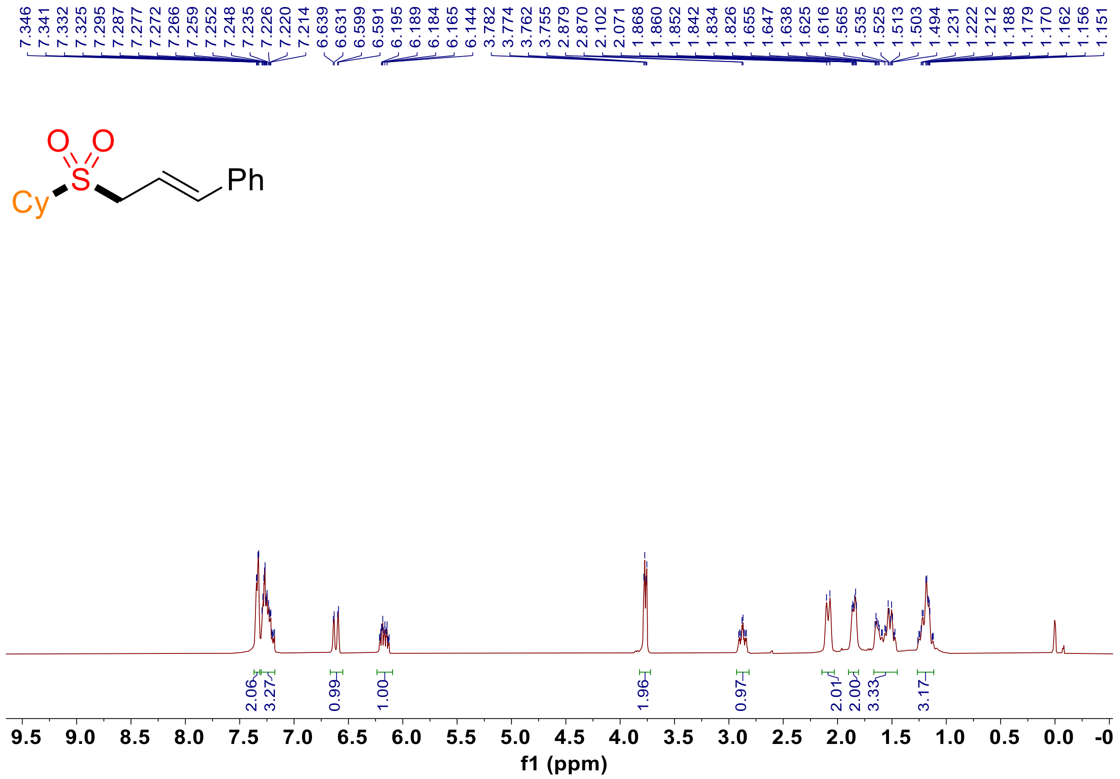
7.346
7.341
7.332
7.325
7.295
7.287
7.277
7.272
7.266
7.259
7.252
7.248
7.235
7.226
7.220
7.214
6.639
6.631
6.599
6.591
6.195
6.189
6.184
6.165
6.144
3.782
3.774
3.762
3.755
2.879
2.870
2.102
2.071
1.868
1.860
1.852
1.842
1.834
1.826
1.655
1.647
1.638
1.625
1.616
1.565
1.535
1.525
1.513
1.503
1.494
1.231
1.222
1.212
1.188
1.179
1.170
1.162
1.156
1.151
O
O
S
Ph
Cy
2.06
3.27
0.99
1.00
1.96
0.97
2.01
2.00
3.33
3.17
9.5 9.0 8.5 8.0 7.5 7.0 6.5 6.0 5.5 5.0 4.5 4.0 3.5 3.0 2.5 2.0 1.5 1.0 0.5 0.0 -0
f1 (ppm)

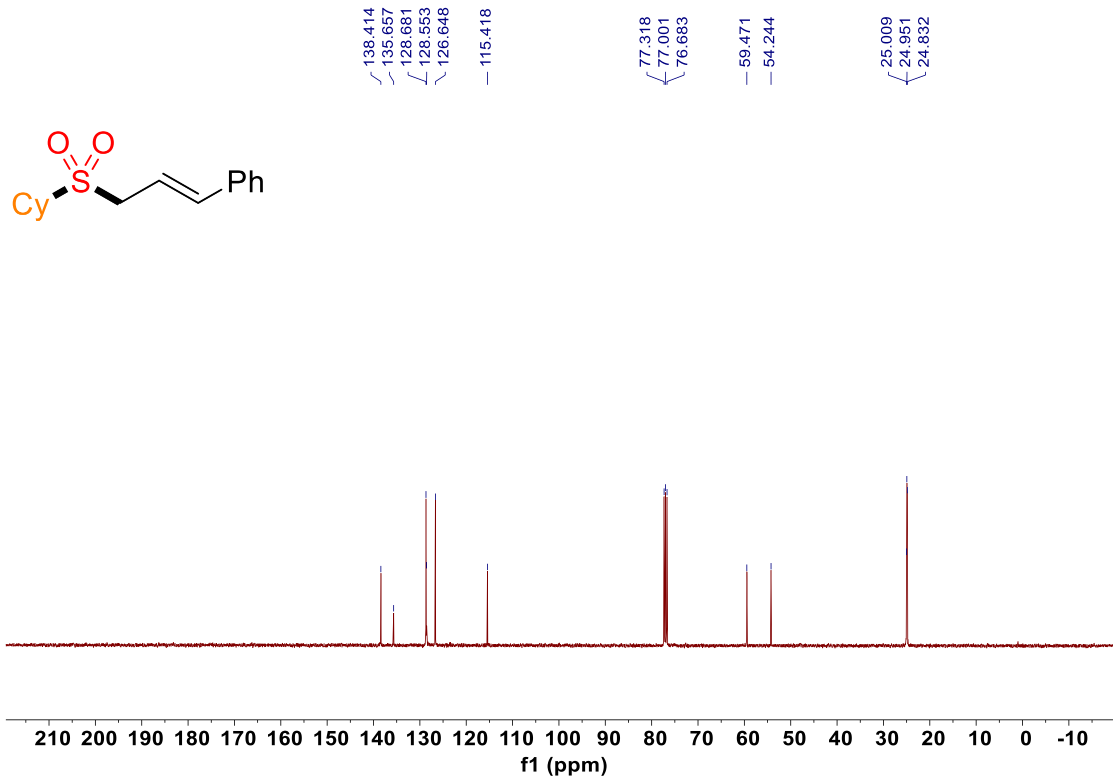
138.414
135.657
128.681
128.553
126.648
115.418
77.318
77.001
76.683
59.471
54.244
25.009
24.951
24.832
Cy
S
O O
Ph
210 200 190 180 170 160 150 140 130 120 110 100 90 80 70 60 50 40 30 20 10 0 -10
f1 (ppm)

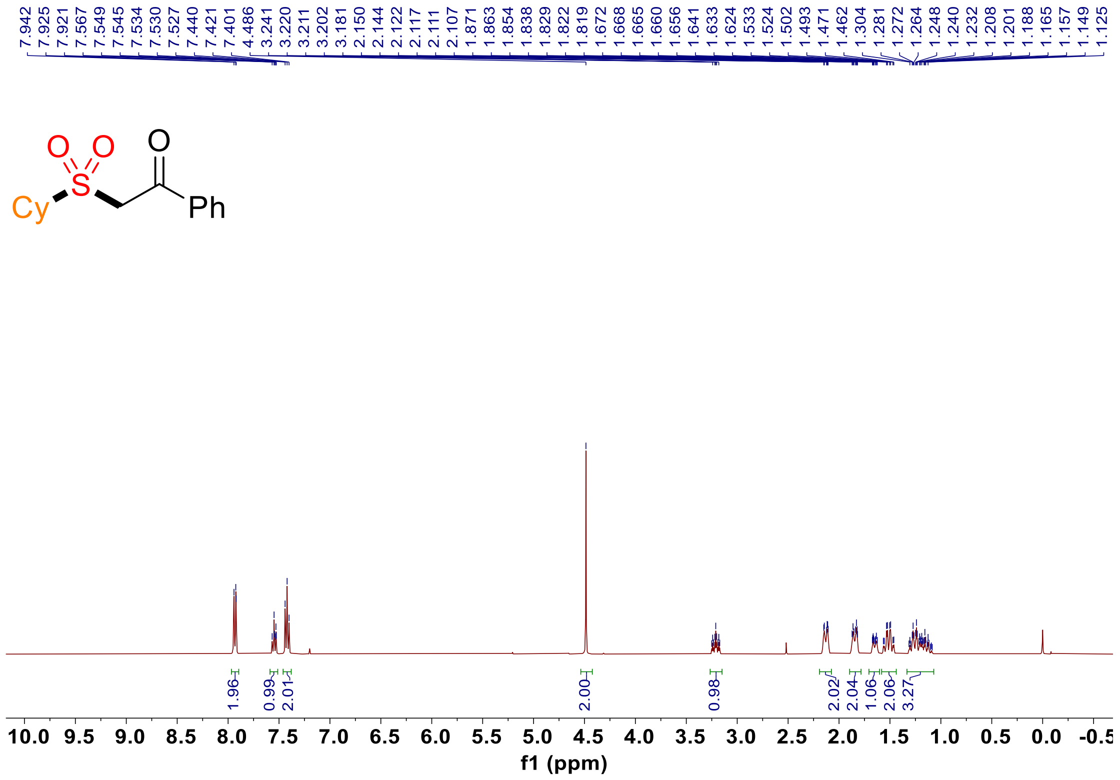
7.942 7.925 7.921 7.567 7.549 7.545 7.534 7.530 7.527 7.440 7.421 7.401 4.486 3.241 3.220 3.211 3.202 3.181 2.150 2.144 2.122 2.117 2.111 2.107 1.871 1.863 1.854 1.838 1.829 1.822 1.819 1.672 1.668 1.665 1.660 1.656 1.641 1.633 1.624 1.533 1.524 1.502 1.493 1.471 1.462 1.304 1.281 1.272 1.264 1.248 1.240 1.232 1.208 1.201 1.188 1.165 1.157 1.149 1.125
Cy
S
O O
O
Ph
1.96
0.99
2.01
2.00
0.98
2.02
2.04
1.06
2.06
3.27
10.0 9.5 9.0 8.5 8.0 7.5 7.0 6.5 6.0 5.5 5.0 4.5 4.0 3.5 3.0 2.5 2.0 1.5 1.0 0.5 0.0 -0.5
f1 (ppm)

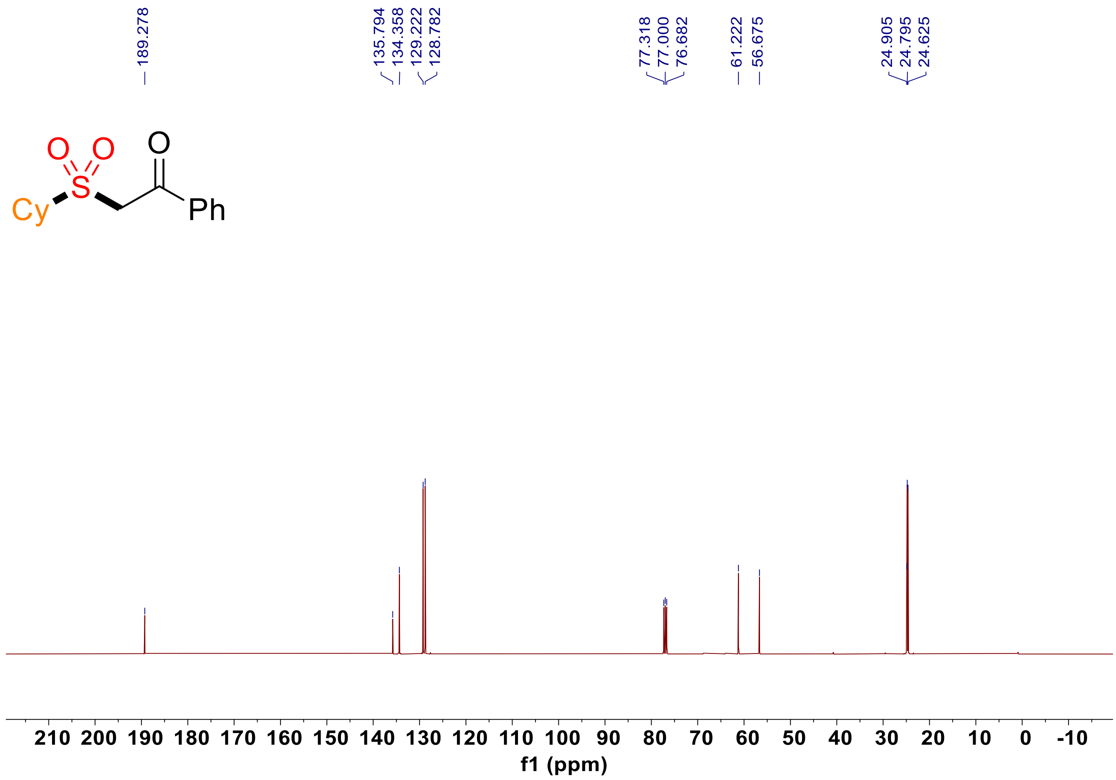
189.278
135.794
134.358
129.222
128.782
77.318
77.000
76.682
61.222
56.675
24.905
24.795
24.625
O
O
S
O
Cy
Ph
210 200 190 180 170 160 150 140 130 120 110 100 90 80 70 60 50 40 30 20 10 0 -10
f1 (ppm)

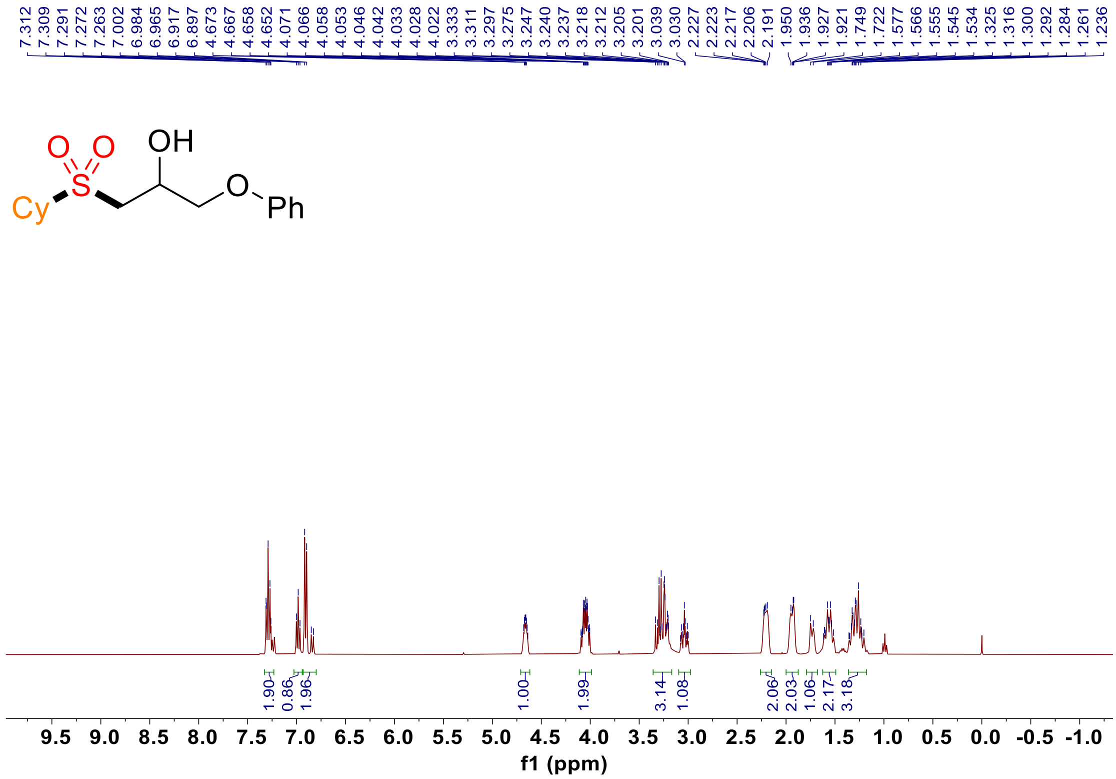
7.312
7.309
7.291
7.272
7.263
7.002
6.984
6.965
6.917
6.897
4.673
4.667
4.658
4.652
4.071
4.066
4.058
4.053
4.046
4.042
4.033
4.028
4.022
3.333
3.311
3.297
3.275
3.247
3.240
3.237
3.218
3.212
3.205
3.201
3.039
3.030
2.227
2.223
2.217
2.206
2.191
1.950
1.936
1.927
1.921
1.749
1.722
1.577
1.566
1.555
1.545
1.534
1.325
1.316
1.300
1.292
1.284
1.261
1.236
O
O
S
OH
O
Cy
Ph
1.90
0.86
1.96
1.00
1.99
3.14
1.08
2.06
2.03
1.06
2.17
3.18
9.5 9.0 8.5 8.0 7.5 7.0 6.5 6.0 5.5 5.0 4.5 4.0 3.5 3.0 2.5 2.0 1.5 1.0 0.5 0.0 -0.5 -1.0
f1 (ppm)

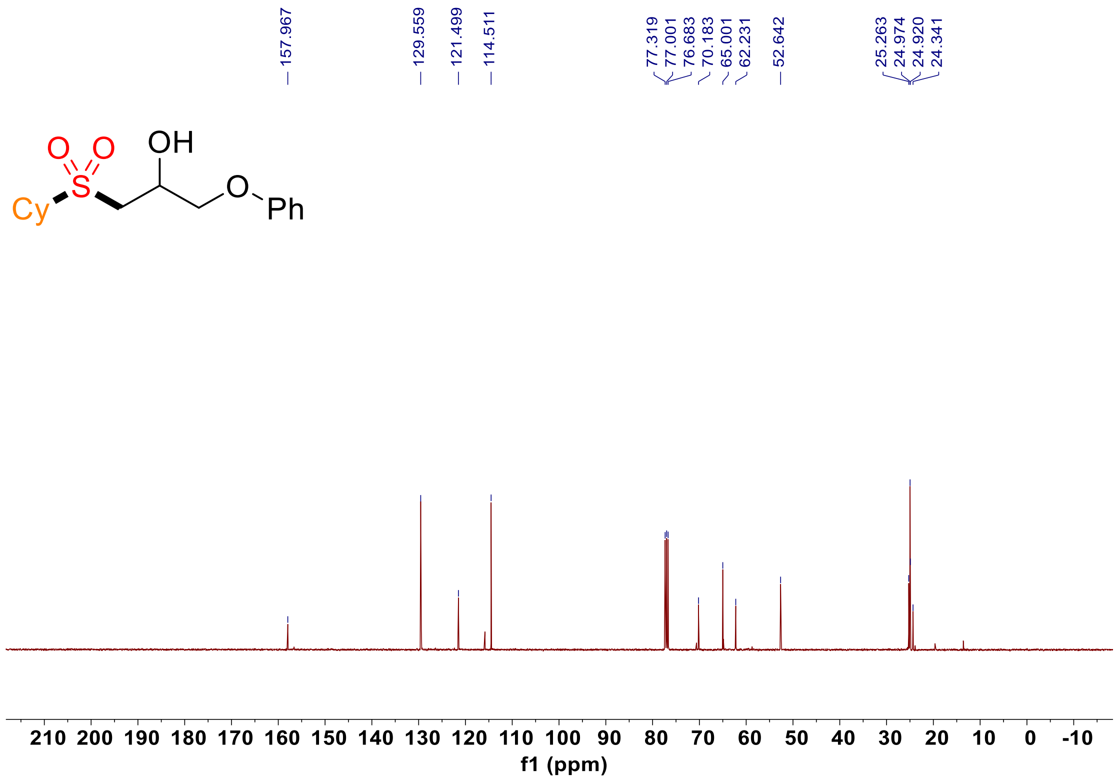

157.967
129.559
121.499
114.511
77.319
77.001
76.683
70.183
65.001
62.231
52.642
25.263
24.974
24.920
24.341
OH
O
Ph
Cy
S
f1 (ppm)

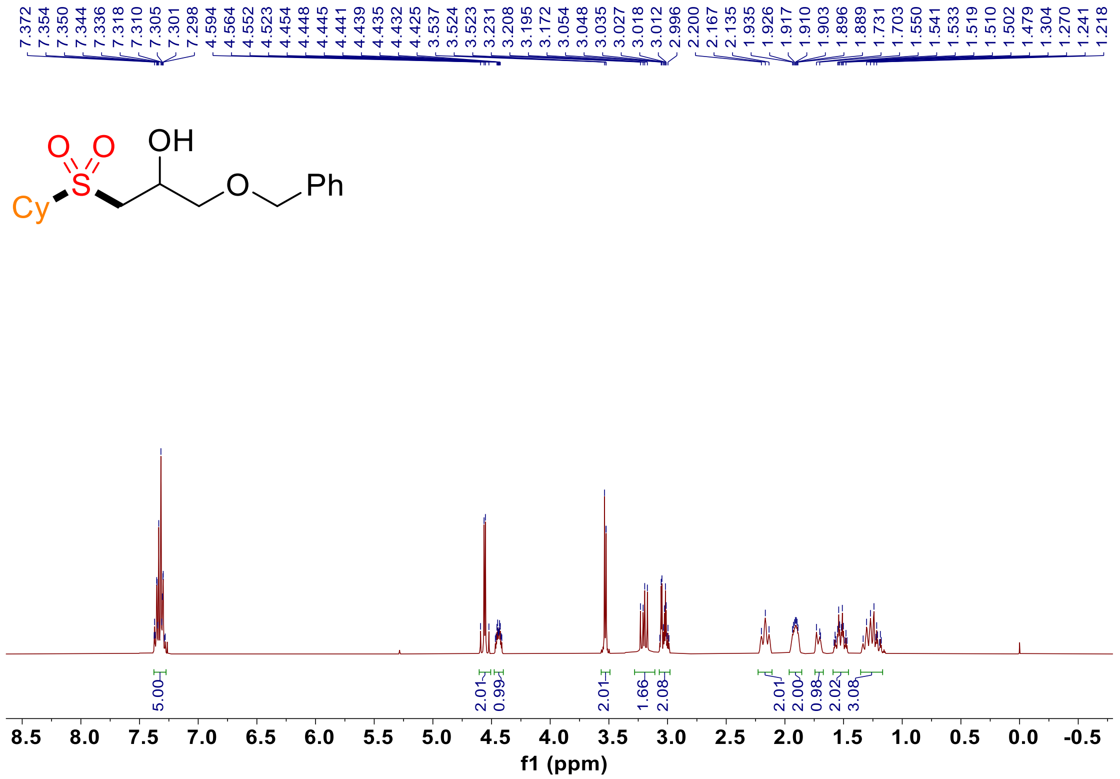

7.372
7.354
7.350
7.344
7.336
7.318
7.310
7.305
7.301
7.298
4.594
4.564
4.552
4.523
4.454
4.448
4.445
4.441
4.439
4.435
4.432
4.425
3.537
3.524
3.523
3.231
3.208
3.195
3.172
3.054
3.048
3.035
3.027
3.018
3.012
2.996
2.200
2.167
2.135
1.935
1.926
1.917
1.910
1.903
1.896
1.889
1.731
1.703
1.550
1.541
1.533
1.519
1.510
1.502
1.479
1.304
1.270
1.241
1.218
OH
O
Ph
Cy
S
5.00
2.01
0.99
2.01
1.66
2.08
2.01
2.00
0.98
2.02
3.08
f1 (ppm)

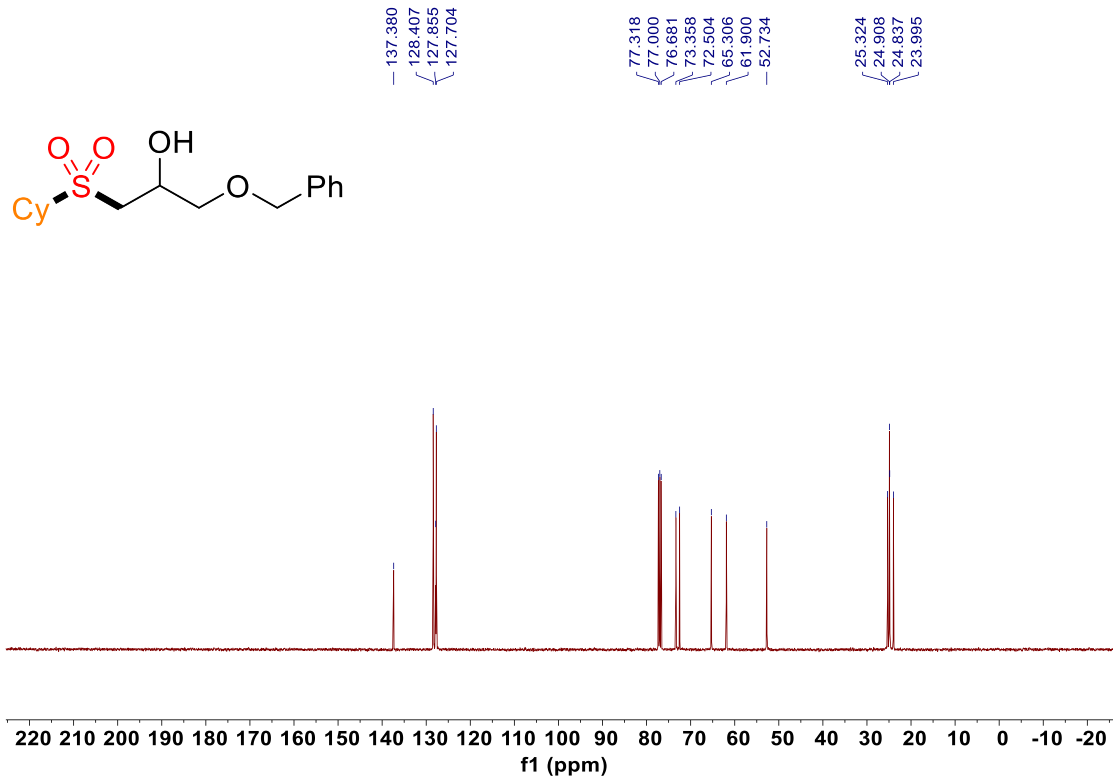

137.380
128.407
127.855
127.704
77.318
77.000
76.681
73.358
72.504
65.306
61.900
52.734
25.324
24.908
24.837
23.995
OH
O
Ph
Cy
S
O O
220 210 200 190 180 170 160 150 140 130 120 110 100 90 80 70 60 50 40 30 20 10 0 -10 -20
f1 (ppm)

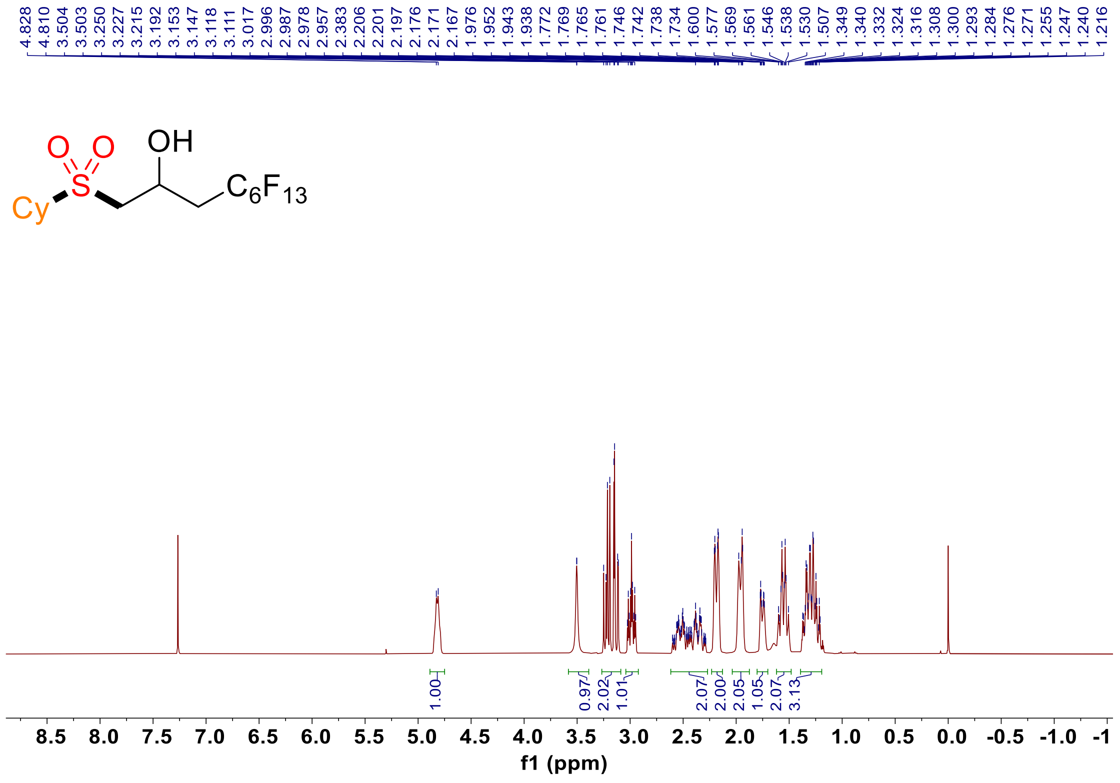

4.828
4.810
3.504
3.503
3.250
3.227
3.215
3.192
3.153
3.147
3.118
3.111
3.017
2.996
2.987
2.978
2.957
2.383
2.206
2.201
2.197
2.176
2.171
2.167
1.976
1.952
1.943
1.938
1.772
1.769
1.765
1.761
1.746
1.742
1.738
1.734
1.600
1.577
1.569
1.561
1.546
1.538
1.530
1.507
1.349
1.340
1.332
1.324
1.316
1.308
1.300
1.293
1.284
1.276
1.271
1.255
1.247
1.240
1.216
OH
C_6F_{13}
Cy
S
O O
1.00
0.97
2.02
1.01
2.07
2.00
2.05
1.05
2.07
3.13
8.5 8.0 7.5 7.0 6.5 6.0 5.5 5.0 4.5 4.0 3.5 3.0 2.5 2.0 1.5 1.0 0.5 0.0 -0.5 -1.0 -1
f1 (ppm)

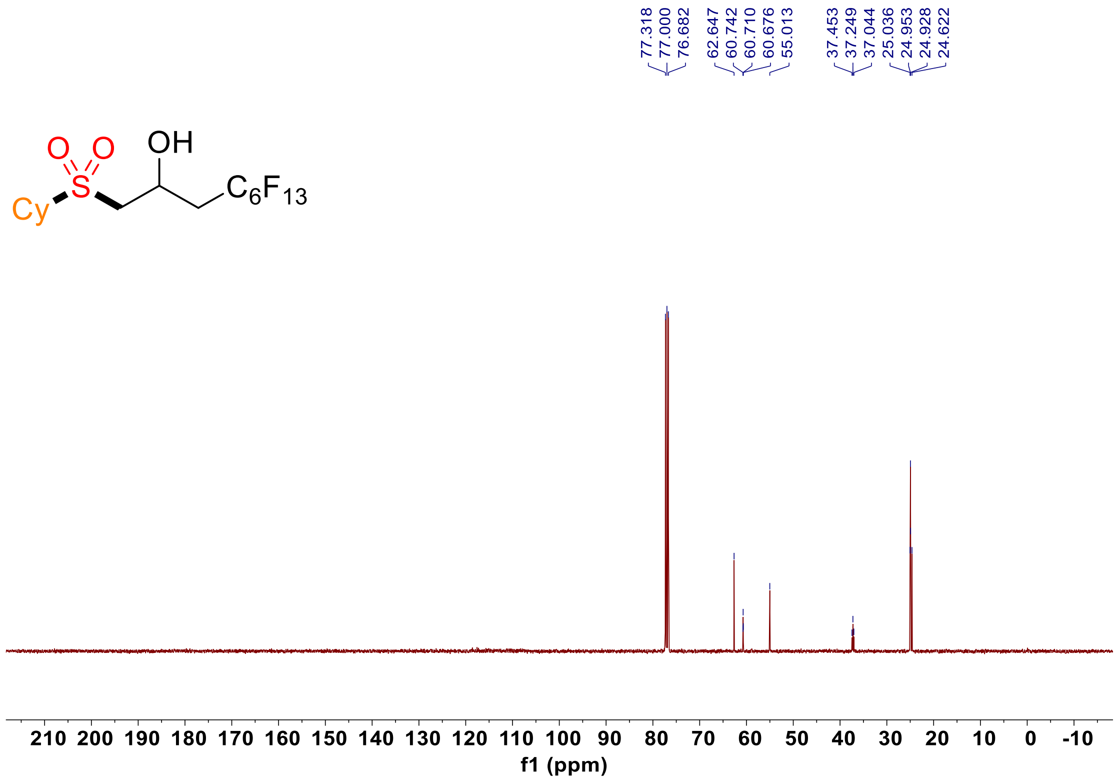

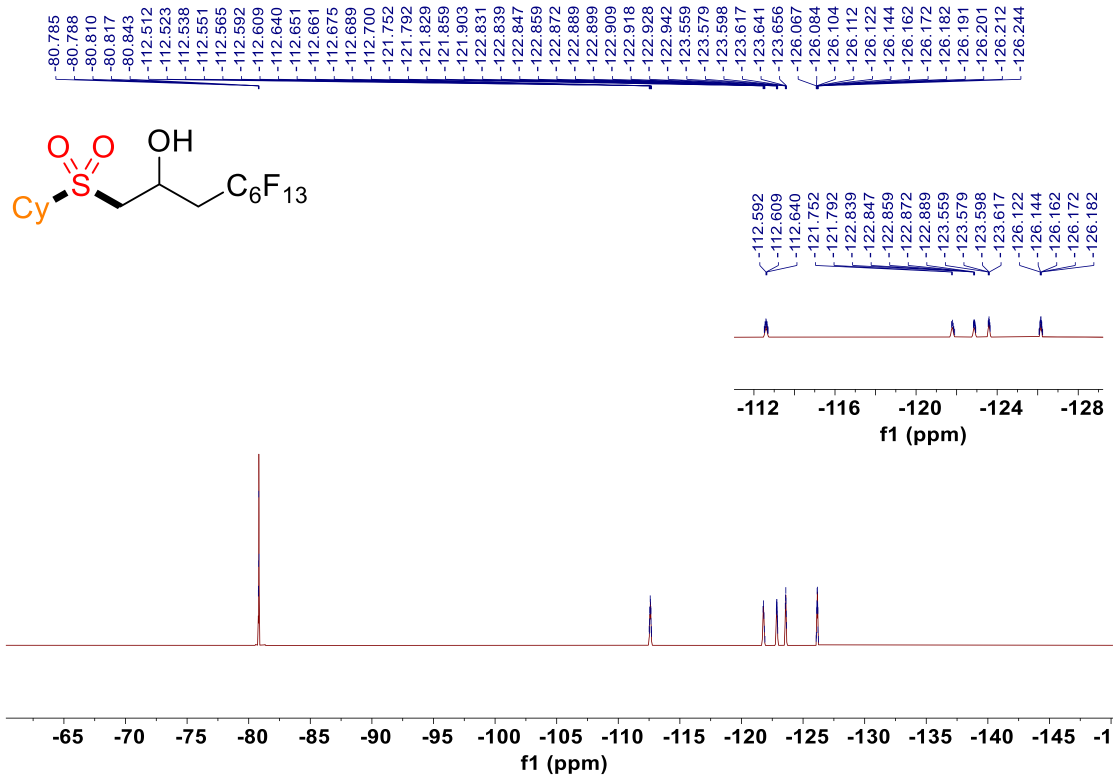

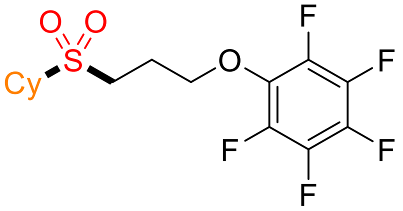

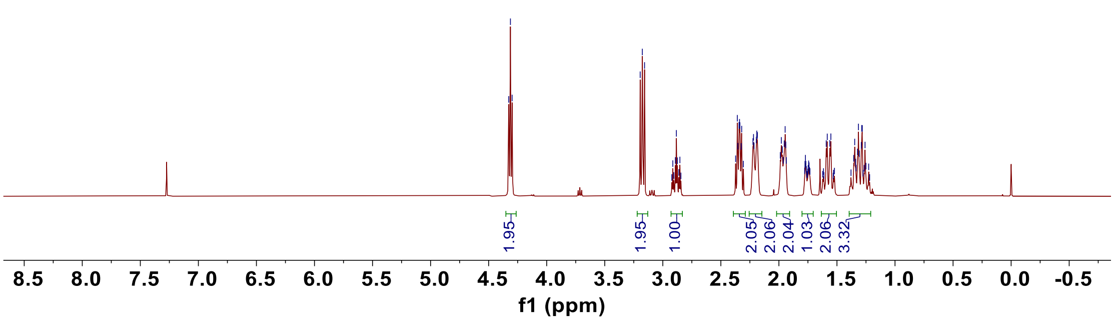
4.327
4.312
4.298
3.195
3.176
3.157
2.914
2.892
2.883
2.874
2.853
2.373
2.358
2.353
2.344
2.339
2.336
2.325
2.321
2.306
2.223
2.219
2.214
2.195
2.190
2.185
1.988
1.979
1.971
1.963
1.954
1.946
1.941
1.937
1.775
1.772
1.768
1.764
1.748
1.744
1.740
1.593
1.584
1.562
1.553
1.356
1.347
1.339
1.335
1.323
1.315
1.308
1.290
1.287
1.282
1.266
1.258
1.250
1.227
1.95
1.95
1.00
2.05
2.06
2.04
1.03
2.06
3.32
8.5 8.0 7.5 7.0 6.5 6.0 5.5 5.0 4.5 4.0 3.5 3.0 2.5 2.0 1.5 1.0 0.5 0.0 -0.5
f1 (ppm)

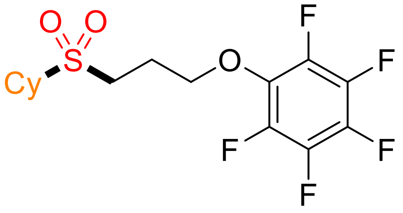

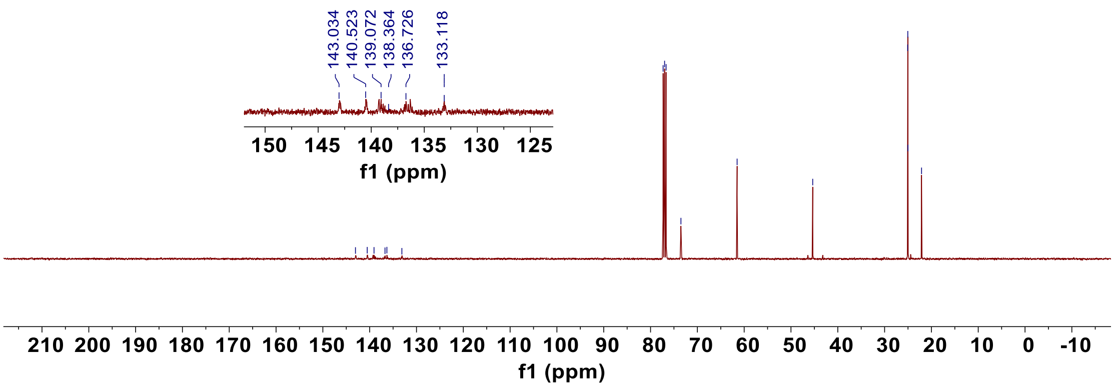
143.034
140.523
139.072
136.726
136.322
133.118
77.318
77.000
76.682
73.506
61.507
45.357
25.071
25.045
25.005
22.106
143.034
140.523
139.072
138.364
136.726
133.118
150 145 140 135 130 125
f1 (ppm)
210 200 190 180 170 160 150 140 130 120 110 100 90 80 70 60 50 40 30 20 10 0 -10
f1 (ppm)

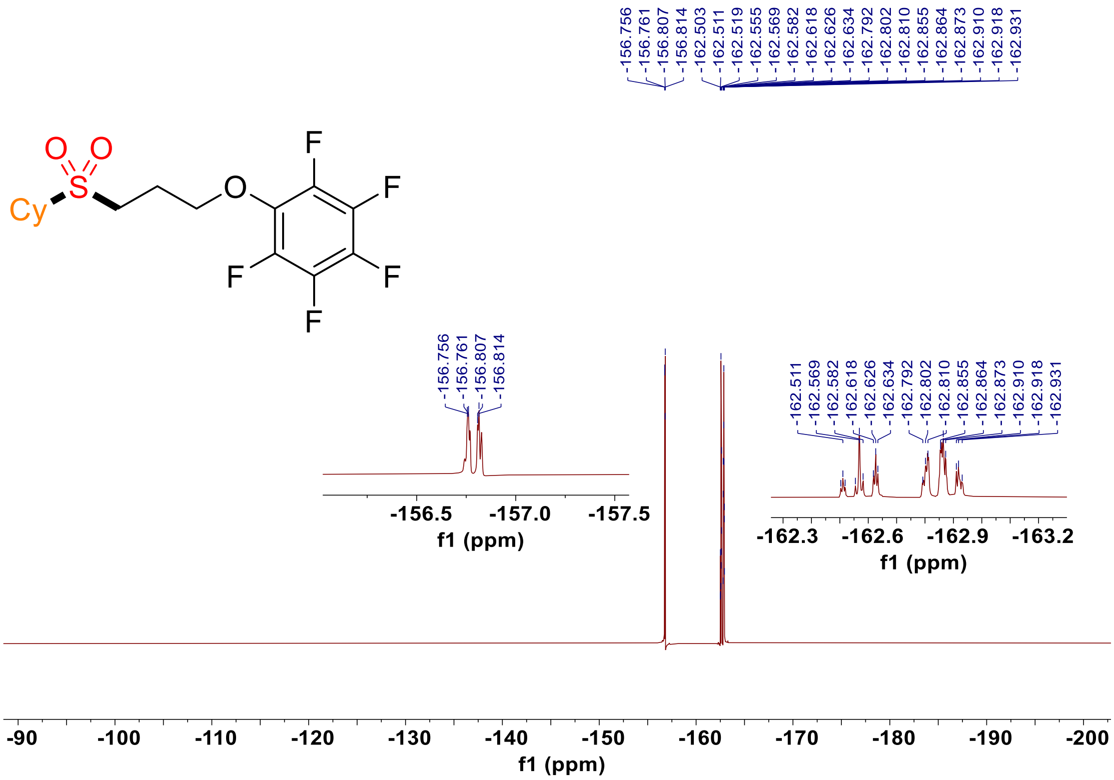
-156.756
-156.761
-156.807
-156.814
-162.503
-162.511
-162.519
-162.555
-162.569
-162.582
-162.618
-162.626
-162.634
-162.792
-162.802
-162.810
-162.855
-162.864
-162.873
-162.910
-162.918
-162.931
O O
S
Cy
O
F
F
F
F
F
-156.5 -157.0 -157.5
f1 (ppm)
-162.3 -162.6 -162.9 -163.2
f1 (ppm)
-90 -100 -110 -120 -130 -140 -150 -160 -170 -180 -190 -200
f1 (ppm)

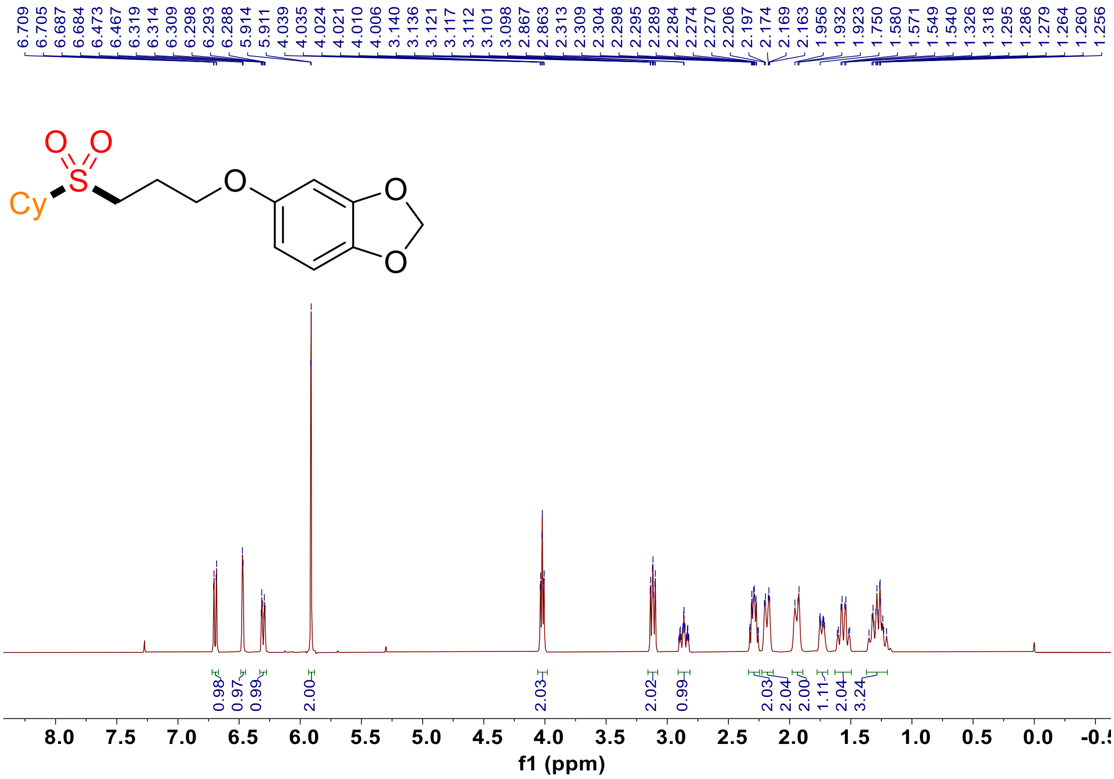
6.709
6.705
6.687
6.684
6.473
6.467
6.319
6.314
6.309
6.298
6.293
6.288
5.914
5.911
4.039
4.035
4.024
4.021
4.010
4.006
3.140
3.136
3.121
3.117
3.112
3.101
3.098
2.867
2.863
2.313
2.309
2.304
2.298
2.295
2.289
2.284
2.274
2.270
2.206
2.197
2.174
2.169
2.163
1.956
1.932
1.923
1.750
1.580
1.571
1.549
1.540
1.326
1.318
1.295
1.286
1.279
1.264
1.260
1.256
O O
S
Cy
O
O
O
0.98
0.97
0.99
2.00
2.03
2.02
0.99
2.03
2.04
2.00
1.11
2.04
3.24
8.0 7.5 7.0 6.5 6.0 5.5 5.0 4.5 4.0 3.5 3.0 2.5 2.0 1.5 1.0 0.5 0.0 -0.
f1 (ppm)

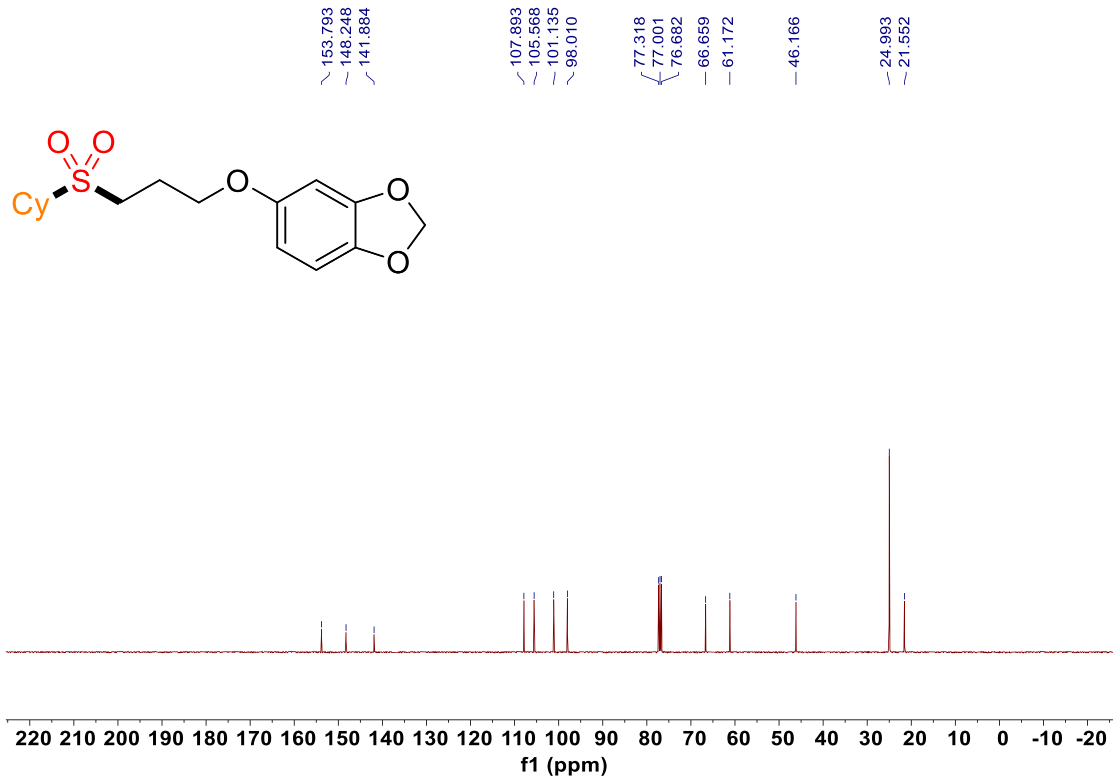
153.793
148.248
141.884
107.893
105.568
101.135
98.010
77.318
77.001
76.682
66.659
61.172
46.166
24.993
21.552
Cy
S
O
O
O
O
O
220 210 200 190 180 170 160 150 140 130 120 110 100 90 80 70 60 50 40 30 20 10 0 -10 -20
f1 (ppm)

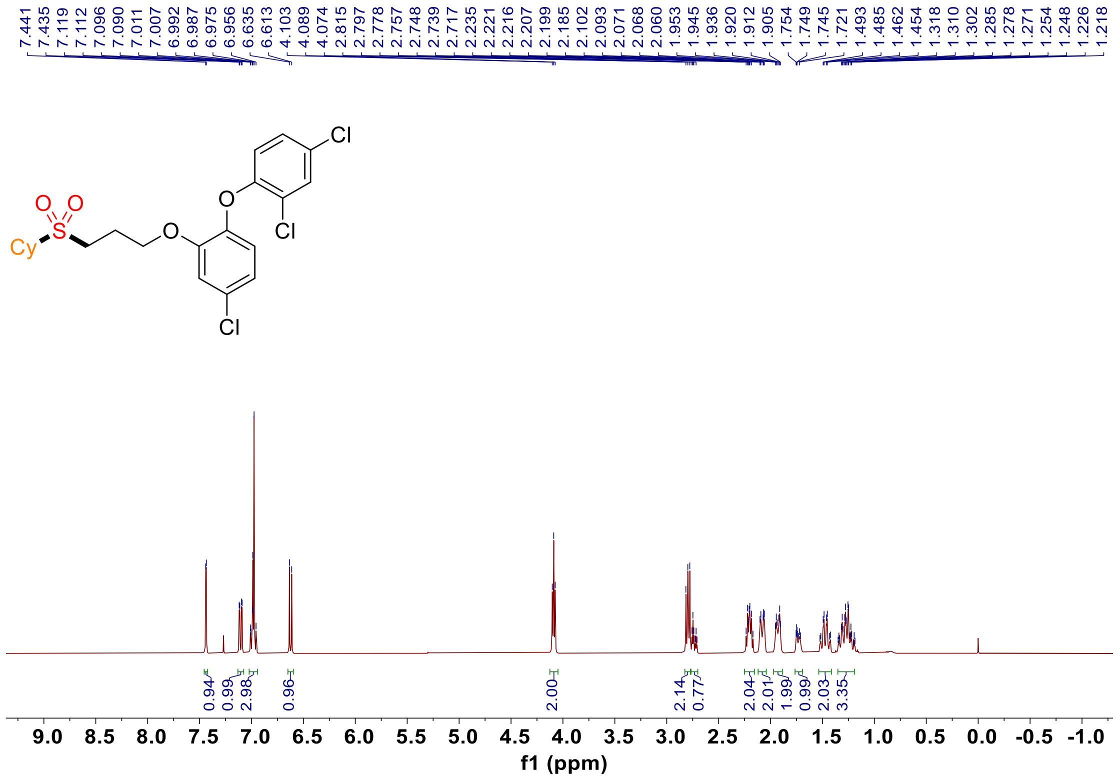
7.441
7.435
7.119
7.112
7.096
7.090
7.011
7.007
6.992
6.987
6.975
6.956
6.635
6.613
4.103
4.089
4.074
2.815
2.797
2.778
2.757
2.748
2.739
2.717
2.235
2.221
2.216
2.207
2.199
2.185
2.102
2.093
2.071
2.068
2.060
1.953
1.945
1.936
1.920
1.912
1.905
1.754
1.749
1.745
1.721
1.493
1.485
1.462
1.454
1.318
1.310
1.302
1.285
1.278
1.271
1.254
1.248
1.226
1.218
Cl
O
Cl
Cy
S
O
O
O
Cl
0.94
0.99
2.98
0.96
2.00
2.14
0.77
2.04
2.01
1.99
0.99
2.03
3.35
9.0 8.5 8.0 7.5 7.0 6.5 6.0 5.5 5.0 4.5 4.0 3.5 3.0 2.5 2.0 1.5 1.0 0.5 0.0 -0.5 -1.0
f1 (ppm)

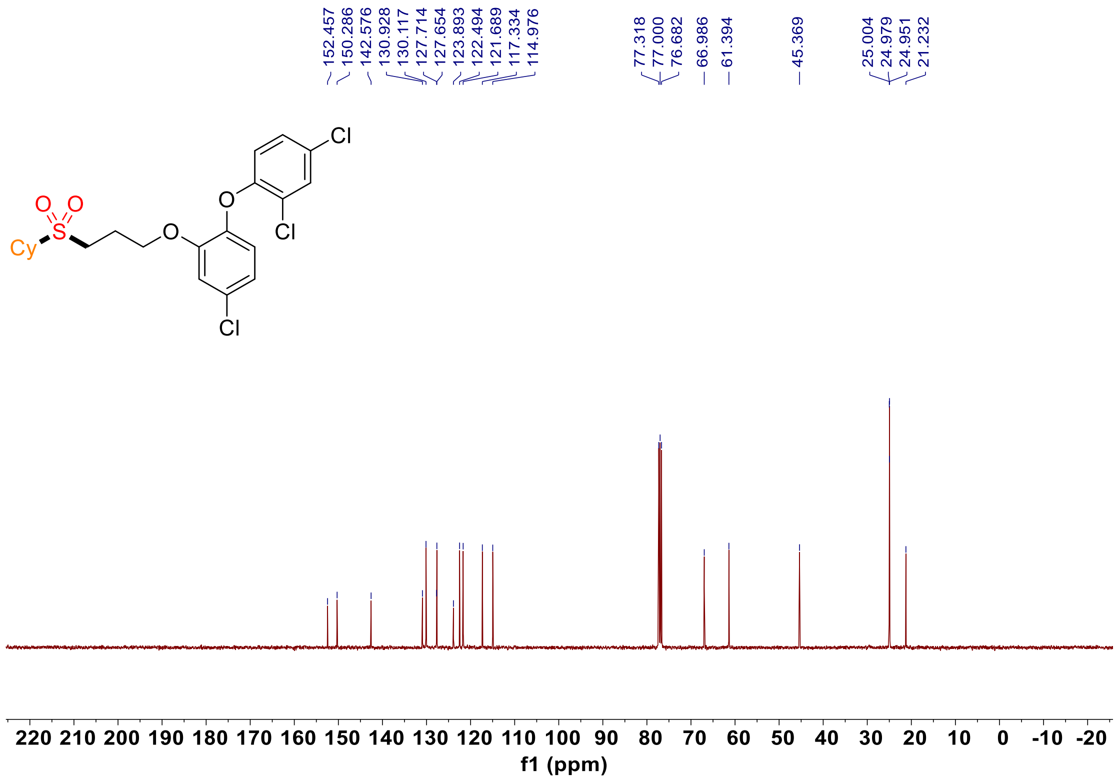
152.457
150.286
142.576
130.928
130.117
127.714
127.654
123.893
122.494
121.689
117.334
114.976
77.318
77.000
76.682
66.986
61.394
45.369
25.004
24.979
24.951
21.232
Cl
O
Cl
O
O
S
O
Cy
Cl
220 210 200 190 180 170 160 150 140 130 120 110 100 90 80 70 60 50 40 30 20 10 0 -10 -20
f1 (ppm)

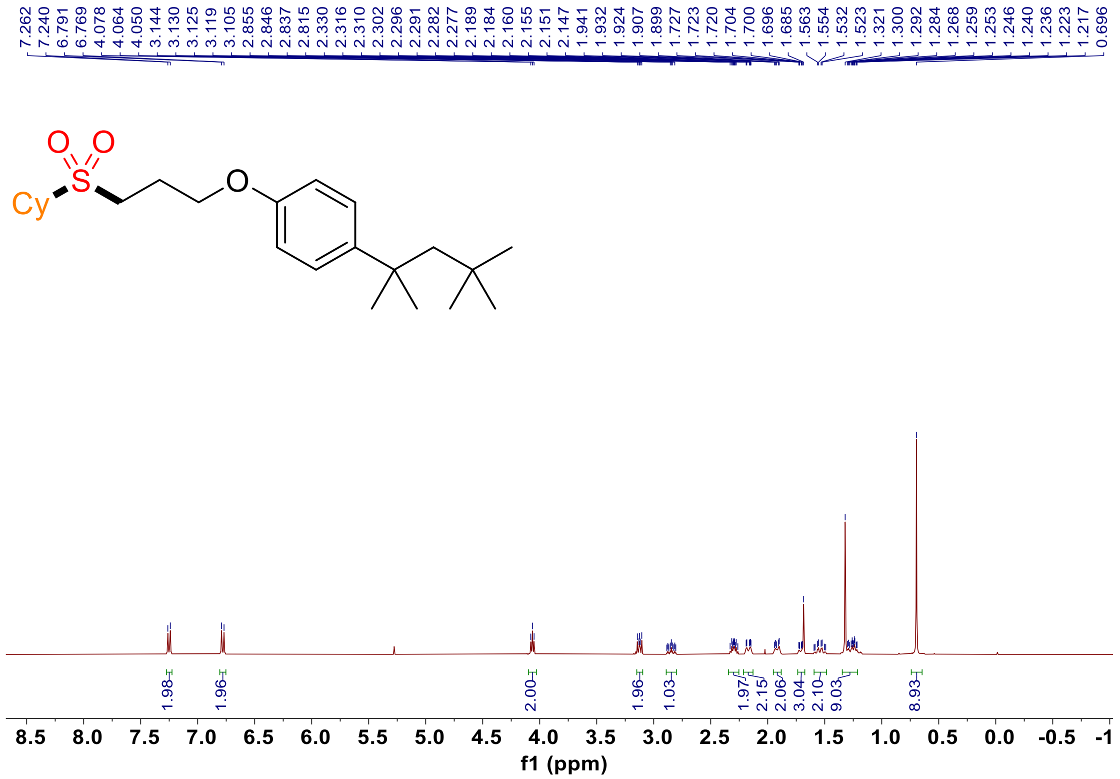
7.262
7.240
6.791
6.769
4.078
4.064
4.050
3.144
3.130
3.125
3.119
3.105
2.855
2.846
2.837
2.815
2.330
2.316
2.310
2.302
2.296
2.291
2.282
2.277
2.189
2.184
2.160
2.155
2.151
2.147
1.941
1.932
1.924
1.907
1.899
1.727
1.723
1.720
1.704
1.700
1.696
1.685
1.563
1.554
1.532
1.523
1.321
1.300
1.292
1.284
1.268
1.259
1.253
1.246
1.240
1.236
1.223
1.217
0.696
O
O
S
Cy
O
1.98
1.96
2.00
1.96
1.03
1.97
2.15
2.06
3.04
2.10
9.03
8.93
8.5 8.0 7.5 7.0 6.5 6.0 5.5 5.0 4.5 4.0 3.5 3.0 2.5 2.0 1.5 1.0 0.5 0.0 -0.5 -1
f1 (ppm)

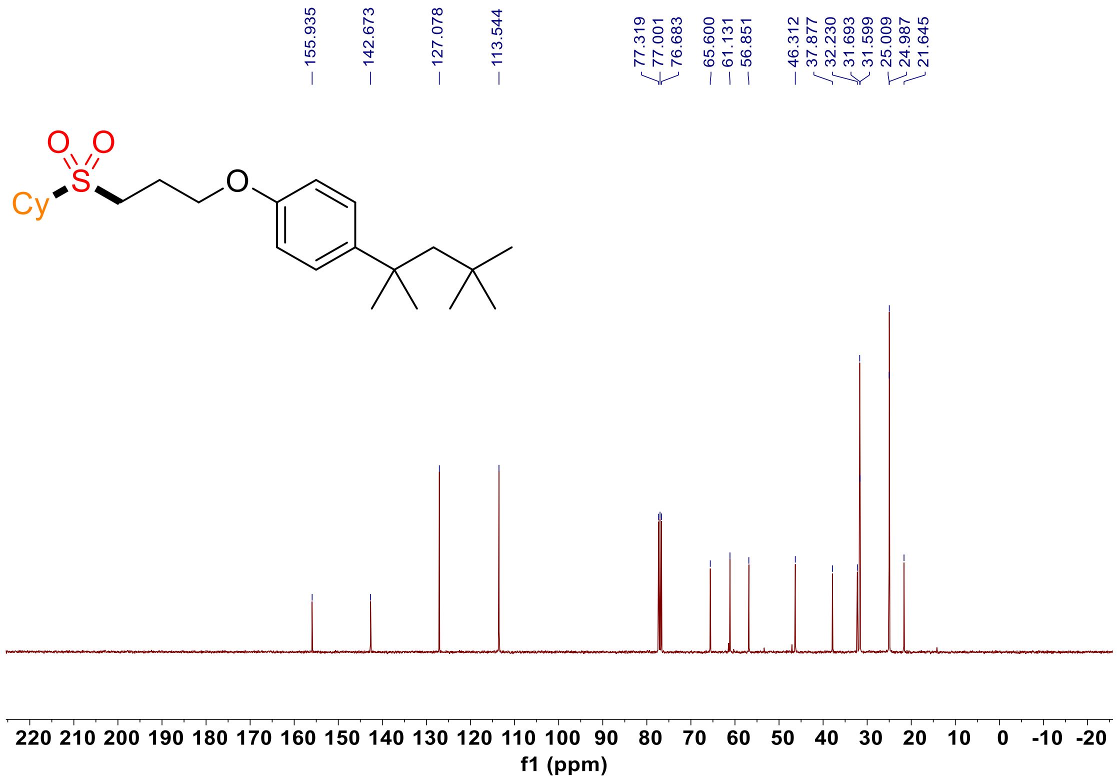

155.935
142.673
127.078
113.544
77.319
77.001
76.683
65.600
61.131
56.851
46.312
37.877
32.230
31.693
31.599
25.009
24.987
21.645
Cy
S
O
O
O
220 210 200 190 180 170 160 150 140 130 120 110 100 90 80 70 60 50 40 30 20 10 0 -10 -20
f1 (ppm)

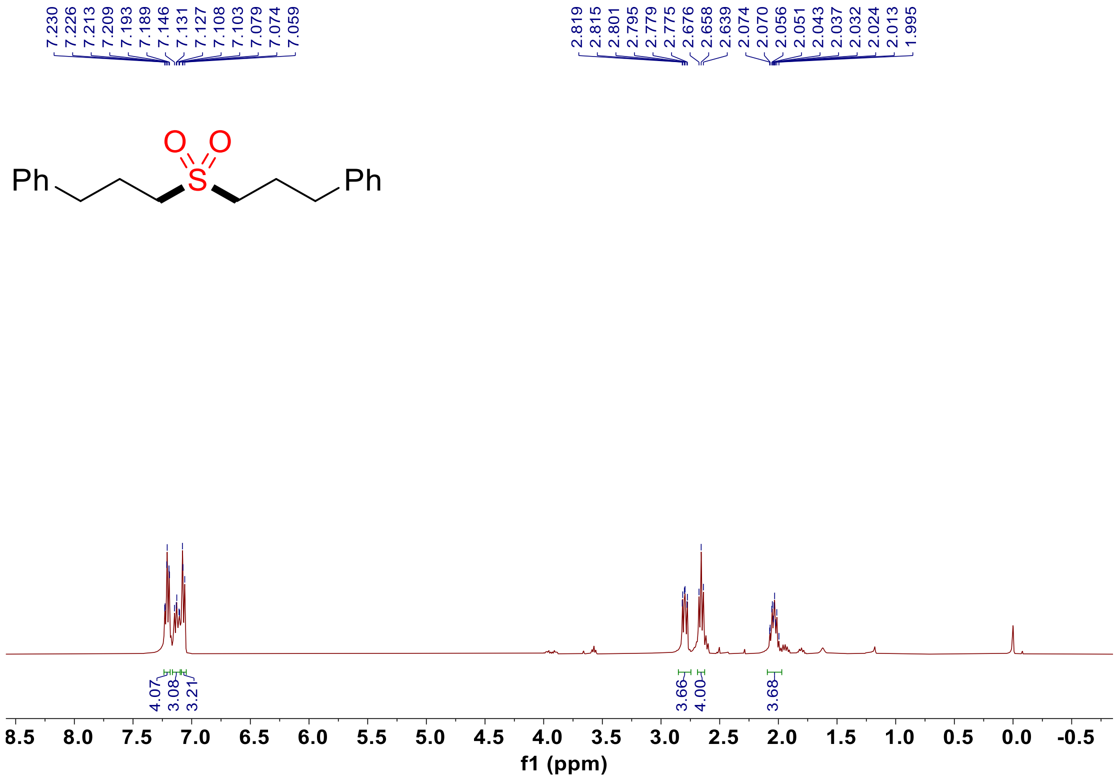

7.230
7.226
7.213
7.209
7.193
7.189
7.146
7.131
7.127
7.108
7.103
7.079
7.074
7.059
2.819
2.815
2.801
2.795
2.779
2.775
2.676
2.658
2.639
2.074
2.070
2.056
2.051
2.043
2.037
2.032
2.024
2.013
1.995
Ph
S
O
O
Ph
4.07
3.08
3.21
3.66
4.00
3.68
8.5 8.0 7.5 7.0 6.5 6.0 5.5 5.0 4.5 4.0 3.5 3.0 2.5 2.0 1.5 1.0 0.5 0.0 -0.5
f1 (ppm)

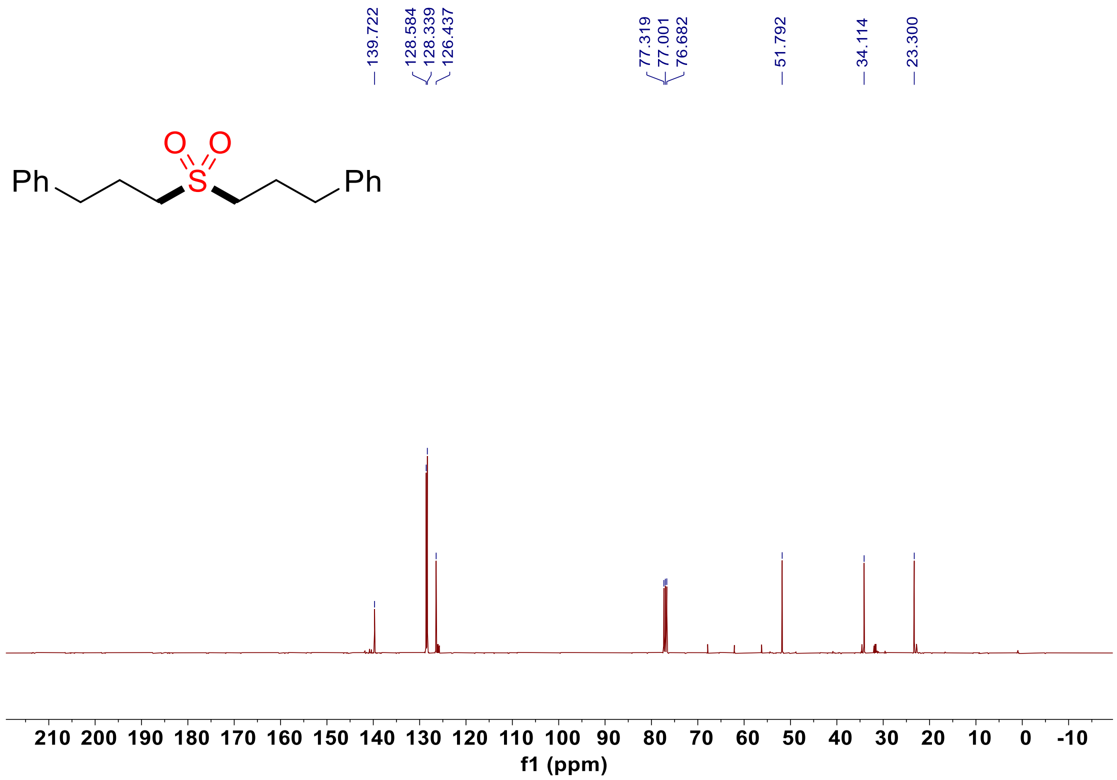
139.722
128.584
128.339
126.437
77.319
77.001
76.682
51.792
34.114
23.300
Ph
O
O
S
Ph
210 200 190 180 170 160 150 140 130 120 110 100 90 80 70 60 50 40 30 20 10 0 -10
f1 (ppm)

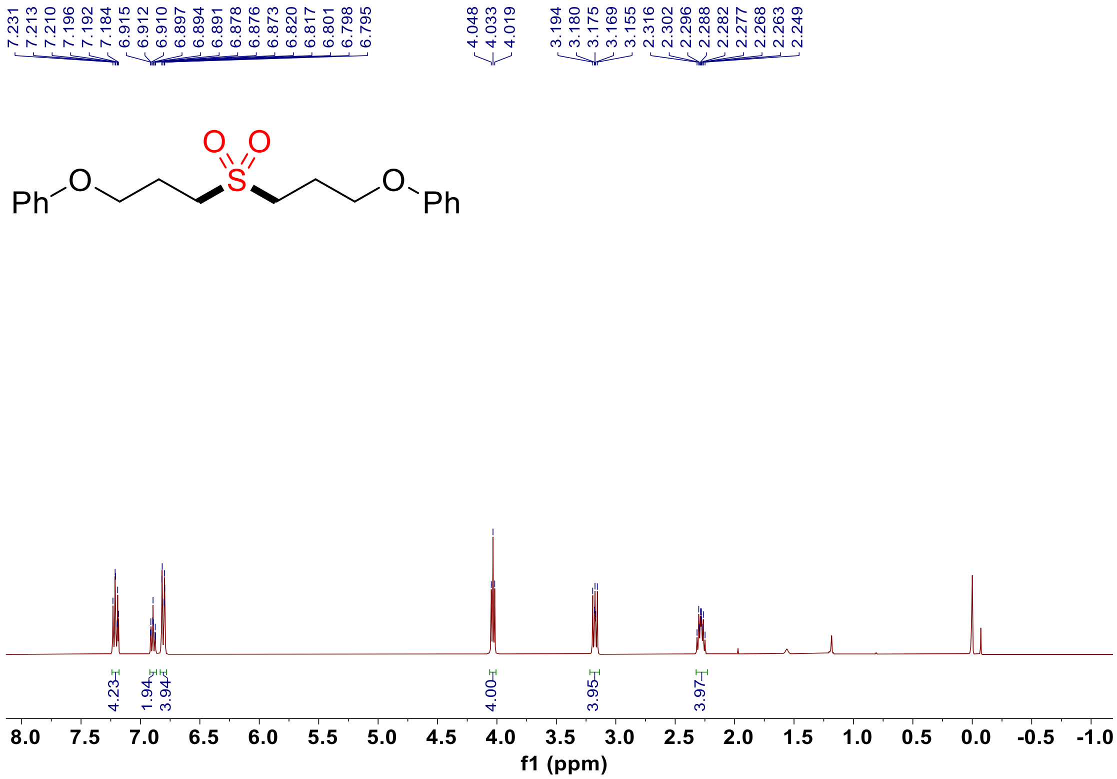
7.231
7.213
7.210
7.196
7.192
7.184
6.915
6.912
6.910
6.897
6.894
6.891
6.878
6.876
6.873
6.820
6.817
6.801
6.798
6.795
4.048
4.033
4.019
3.194
3.180
3.175
3.169
3.155
2.316
2.302
2.296
2.288
2.282
2.277
2.268
2.263
2.249
Ph
O
O
O
S
O
Ph
4.23
1.94
3.94
4.00
3.95
3.97
8.0 7.5 7.0 6.5 6.0 5.5 5.0 4.5 4.0 3.5 3.0 2.5 2.0 1.5 1.0 0.5 0.0 -0.5 -1.0
f1 (ppm)

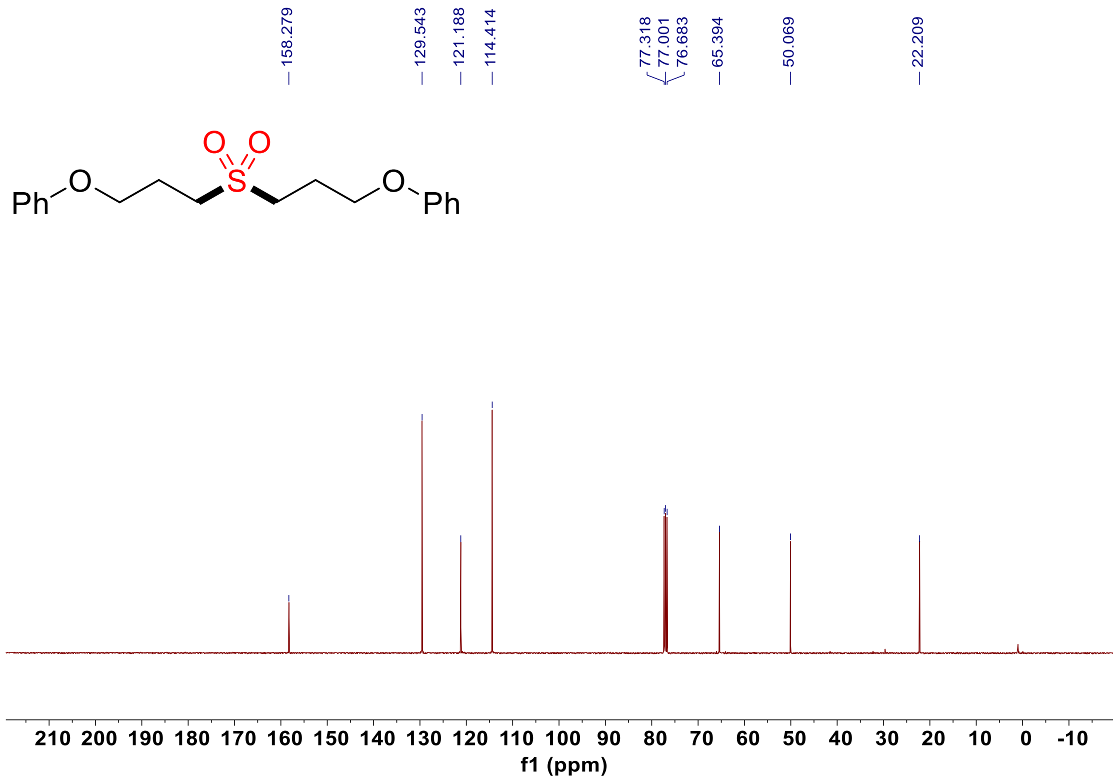
158.279
129.543
121.188
114.414
77.318
77.001
76.683
65.394
50.069
22.209
Ph
O
S
O
O
O
Ph
210 200 190 180 170 160 150 140 130 120 110 100 90 80 70 60 50 40 30 20 10 0 -10
f1 (ppm)

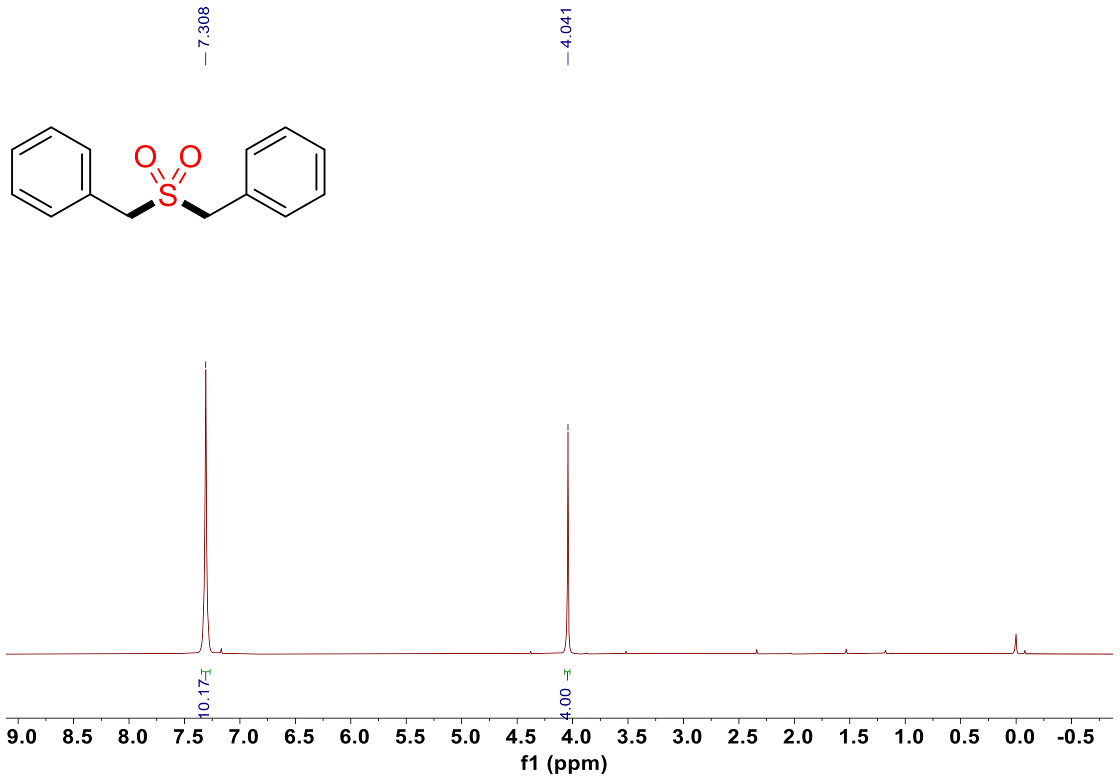
7.308
4.041
O
O
S
10.17
4.00
9.0 8.5 8.0 7.5 7.0 6.5 6.0 5.5 5.0 4.5 4.0 3.5 3.0 2.5 2.0 1.5 1.0 0.5 0.0 -0.5
f1 (ppm)

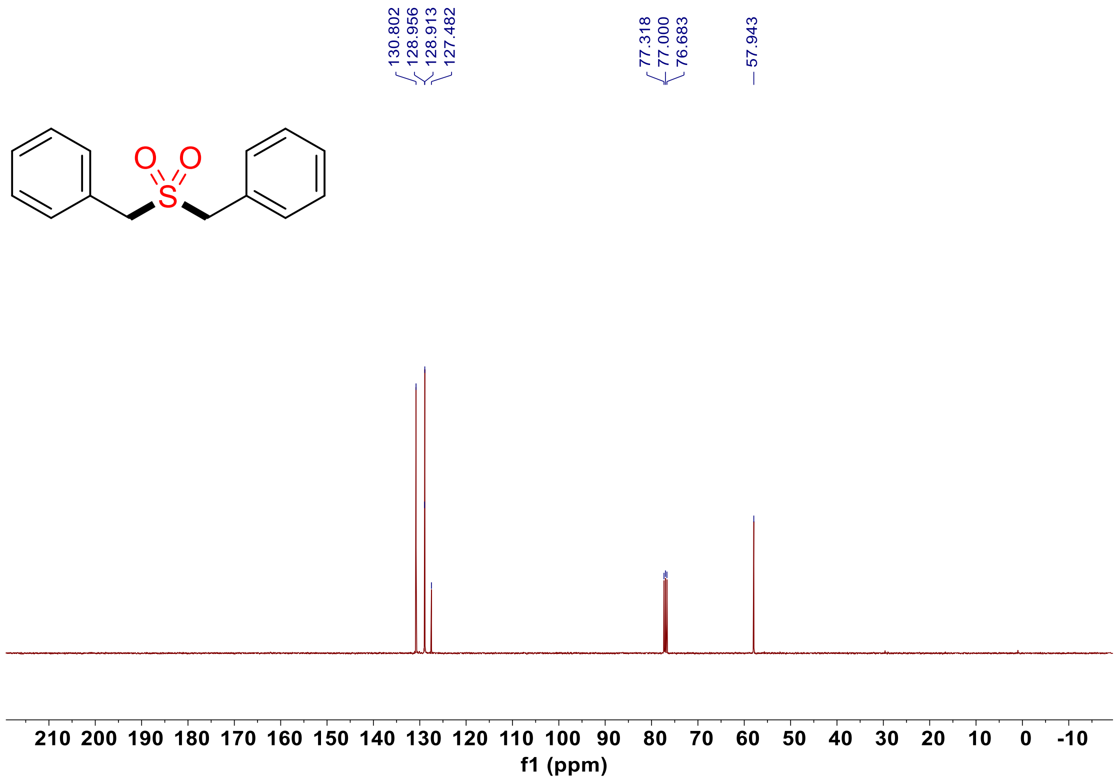
130.802
128.956
128.913
127.482
77.318
77.000
76.683
57.943
O
O
S
210 200 190 180 170 160 150 140 130 120 110 100 90 80 70 60 50 40 30 20 10 0 -10
f1 (ppm)

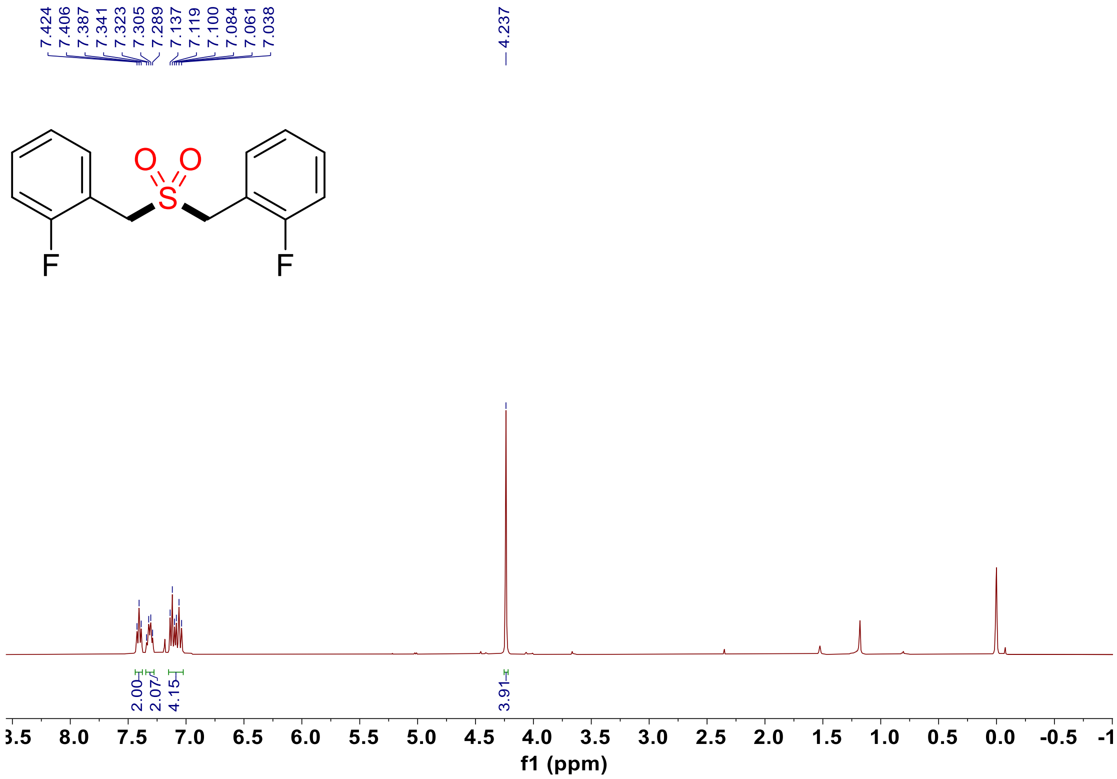
7.424
7.406
7.387
7.341
7.323
7.305
7.289
7.137
7.119
7.100
7.084
7.061
7.038
4.237
O
O
S
F
F
2.00
2.07
4.15
3.91
8.5 8.0 7.5 7.0 6.5 6.0 5.5 5.0 4.5 4.0 3.5 3.0 2.5 2.0 1.5 1.0 0.5 0.0 -0.5 -1
f1 (ppm)

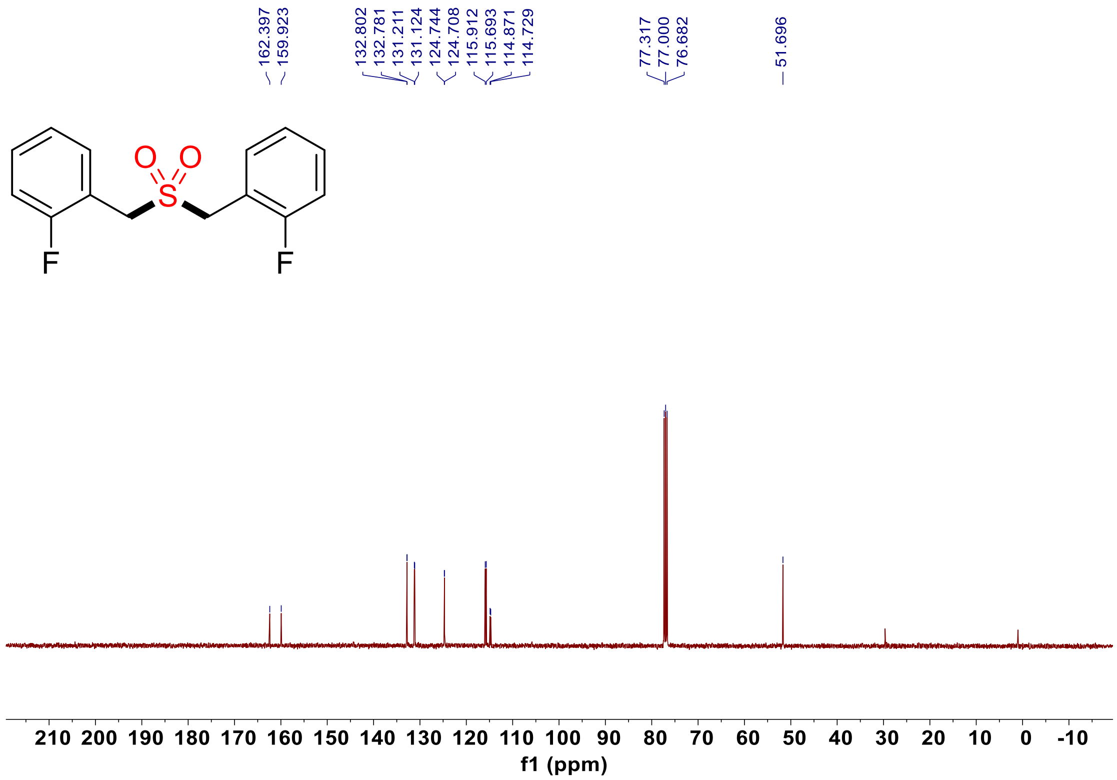
162.397
159.923
132.802
132.781
131.211
131.124
124.744
124.708
115.912
115.693
114.871
114.729
77.317
77.000
76.682
51.696
210 200 190 180 170 160 150 140 130 120 110 100 90 80 70 60 50 40 30 20 10 0 -10
f1 (ppm)

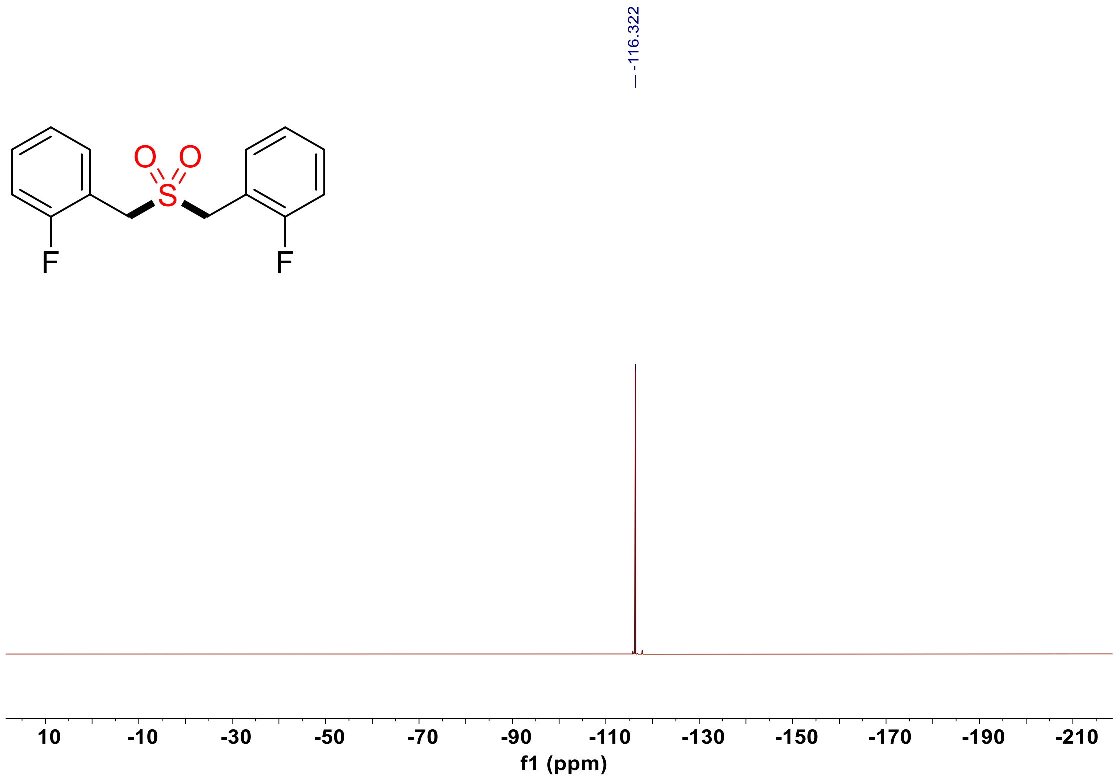
-116.322
10 -10 -30 -50 -70 -90 -110 -130 -150 -170 -190 -210
f1 (ppm)

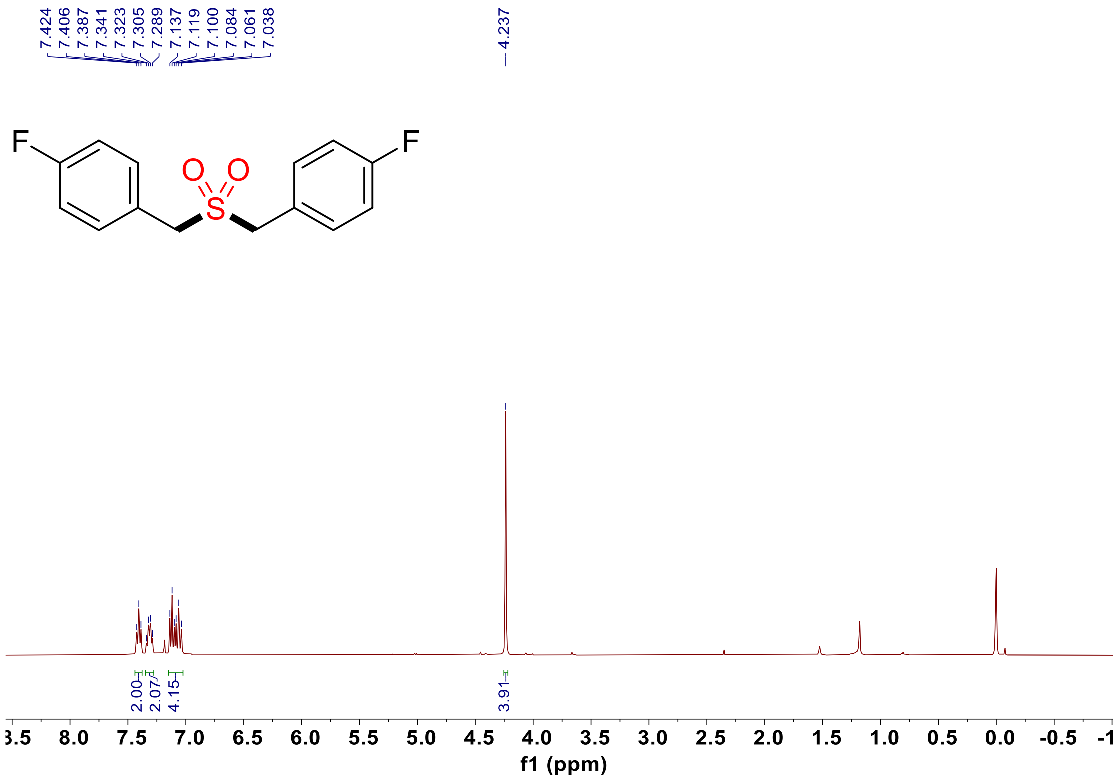
7.424
7.406
7.387
7.341
7.323
7.305
7.289
7.137
7.119
7.100
7.084
7.061
7.038
4.237
F
O
O
S
F
2.00
2.07
4.15
3.91
8.5
8.0
7.5
7.0
6.5
6.0
5.5
5.0
4.5
4.0
3.5
3.0
2.5
2.0
1.5
1.0
0.5
0.0
-0.5
-1
f1 (ppm)

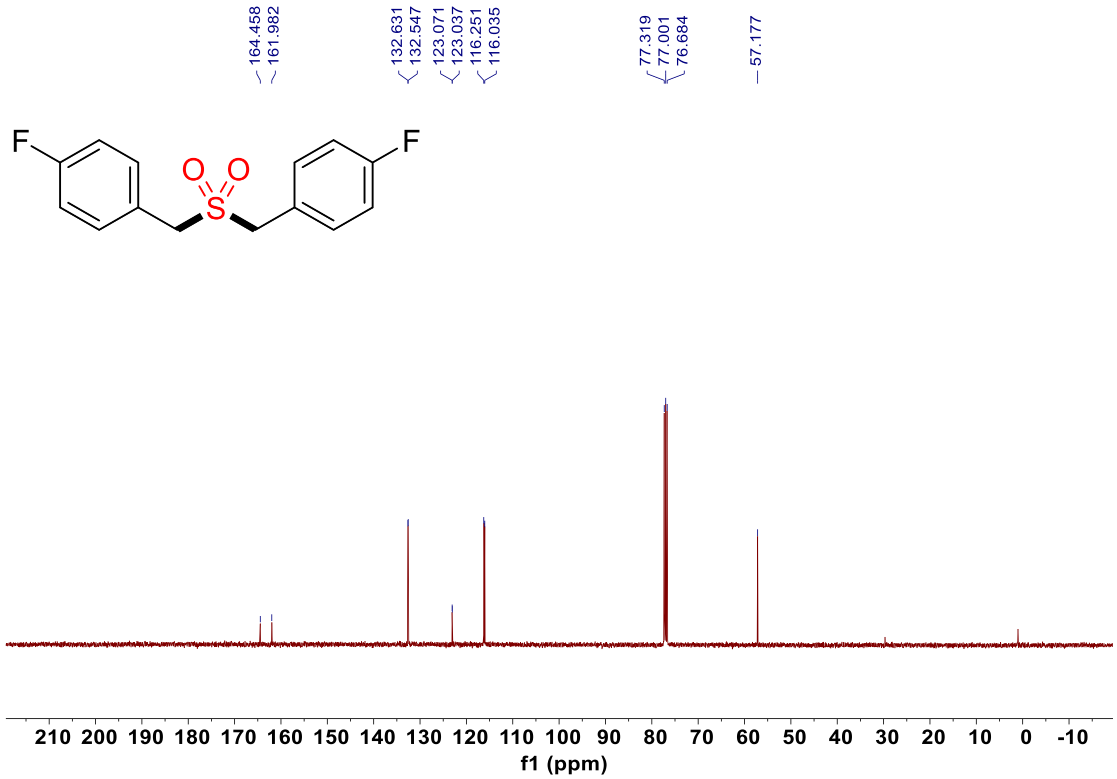
164.458
161.982
132.631
132.547
123.071
123.037
116.251
116.035
77.319
77.001
76.684
57.177
F
O
O
S
F
210
200
190
180
170
160
150
140
130
120
110
100
90
80
70
60
50
40
30
20
10
0
-10
f1 (ppm)

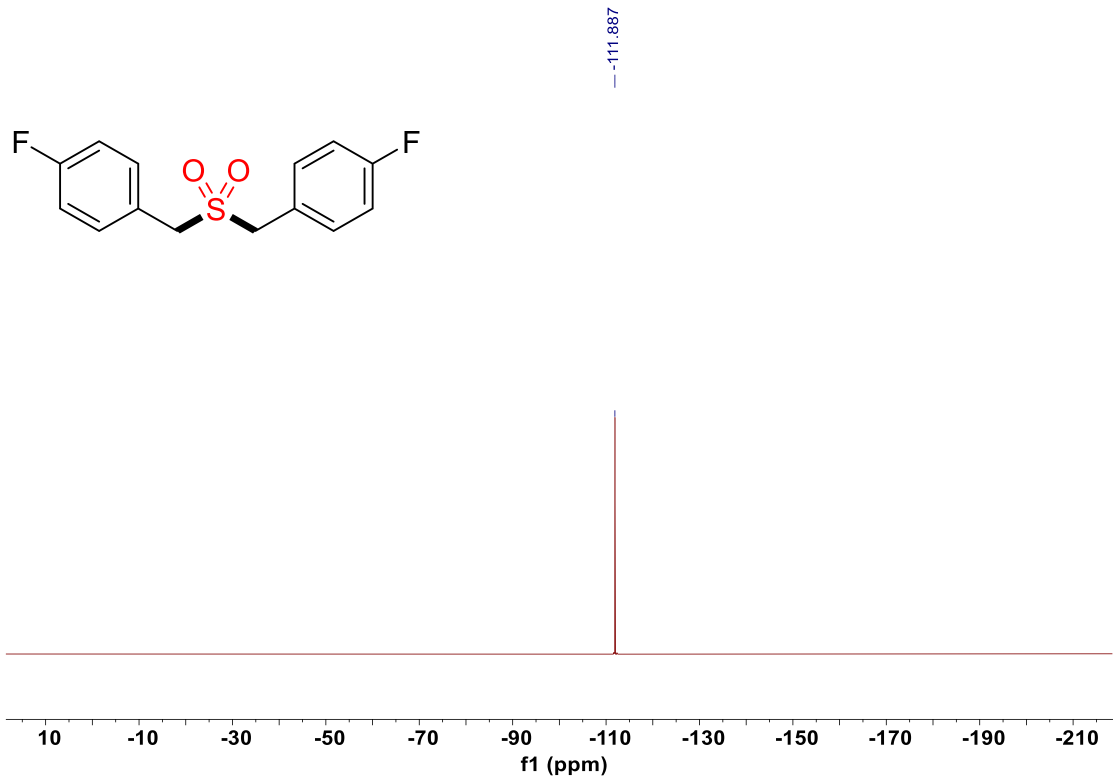
-111.887
F
O
O
S
F
10
-10
-30
-50
-70
-90
-110
-130
-150
-170
-190
-210
f1 (ppm)

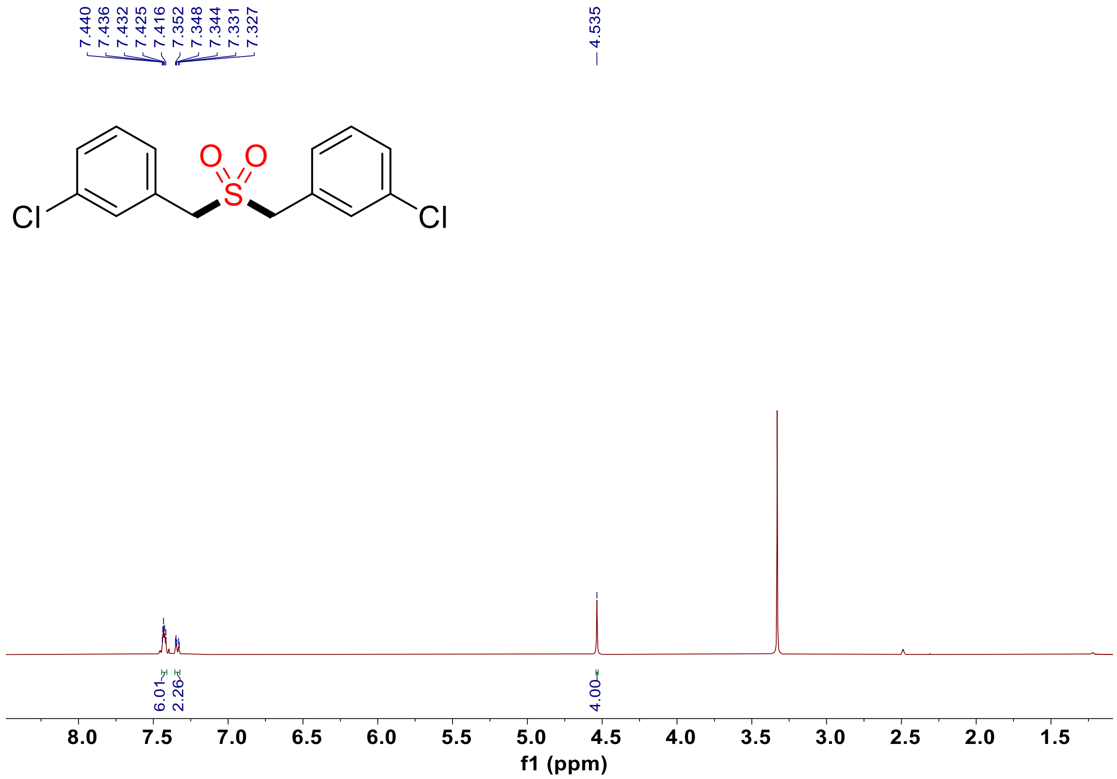
7.440
7.436
7.432
7.425
7.416
7.352
7.348
7.344
7.331
7.327
4.535
Cl
O
O
S
Cl
6.01
2.26
4.00
8.0
7.5
7.0
6.5
6.0
5.5
5.0
4.5
4.0
3.5
3.0
2.5
2.0
1.5
f1 (ppm)

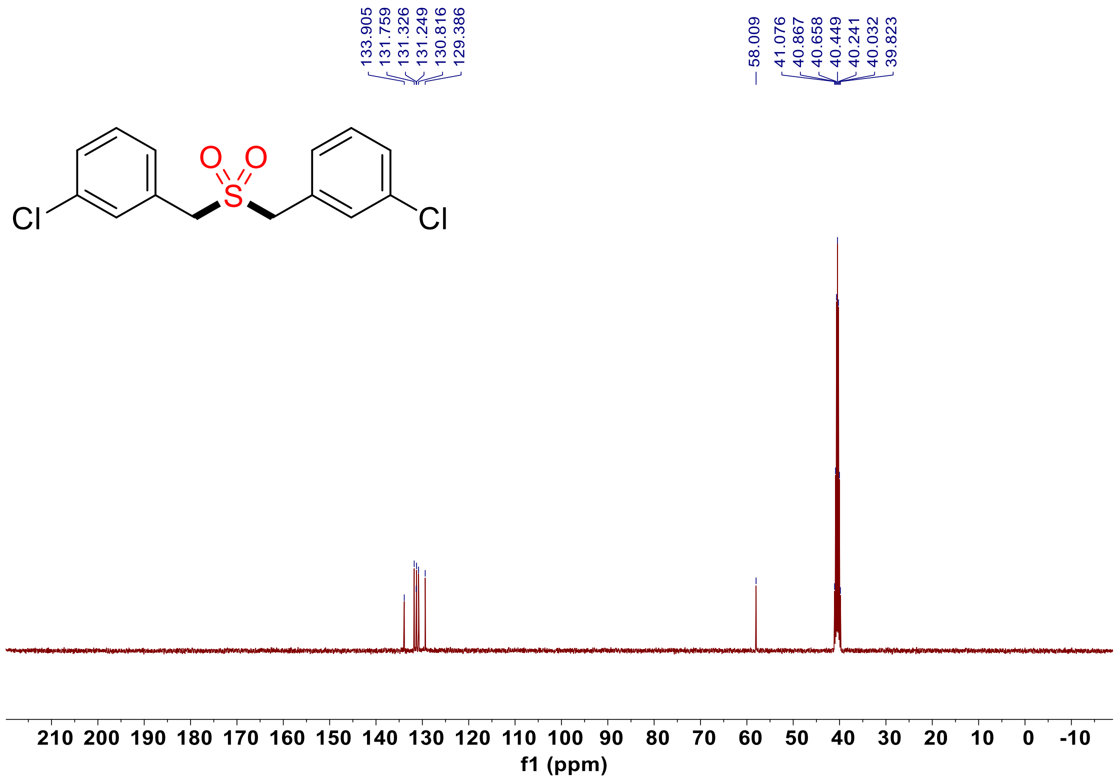

133.905
131.759
131.326
131.249
130.816
129.386
58.009
41.076
40.867
40.658
40.449
40.241
40.032
39.823
Cl
O
S
O
Cl
210 200 190 180 170 160 150 140 130 120 110 100 90 80 70 60 50 40 30 20 10 0 -10
f1 (ppm)

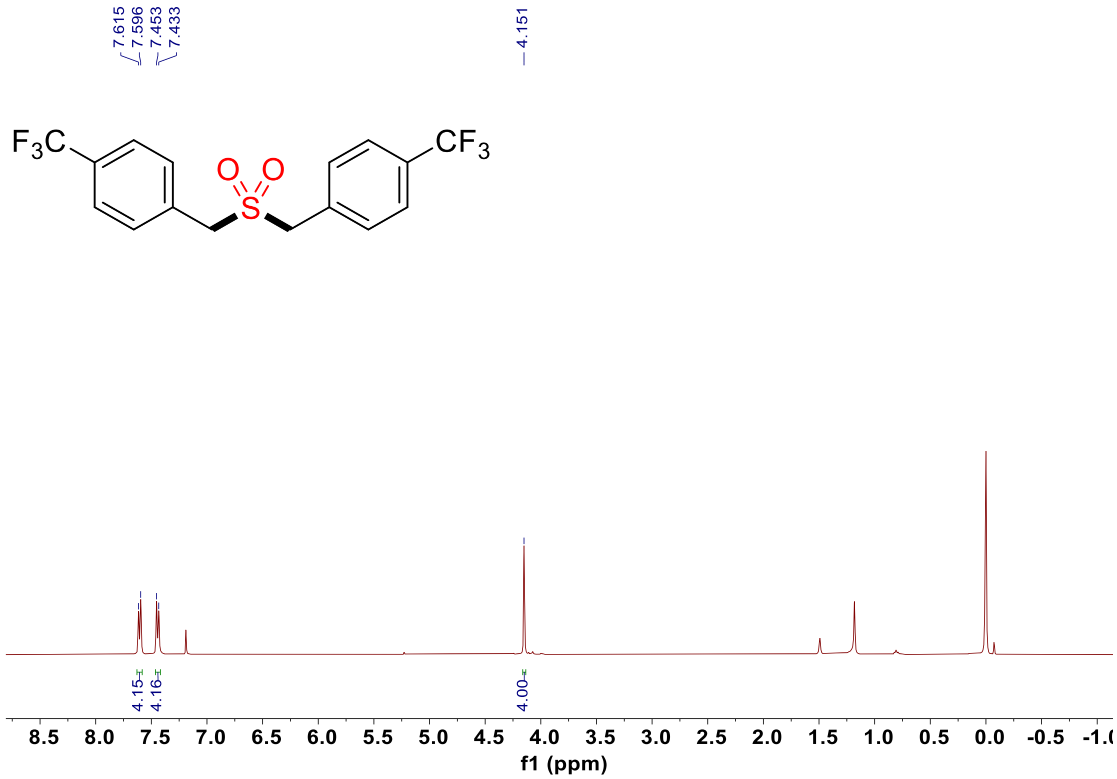

7.615
7.596
7.453
7.433
4.151
F3C
O
S
O
CF3
4.15
4.16
4.00
8.5 8.0 7.5 7.0 6.5 6.0 5.5 5.0 4.5 4.0 3.5 3.0 2.5 2.0 1.5 1.0 0.5 0.0 -0.5
f1 (ppm)

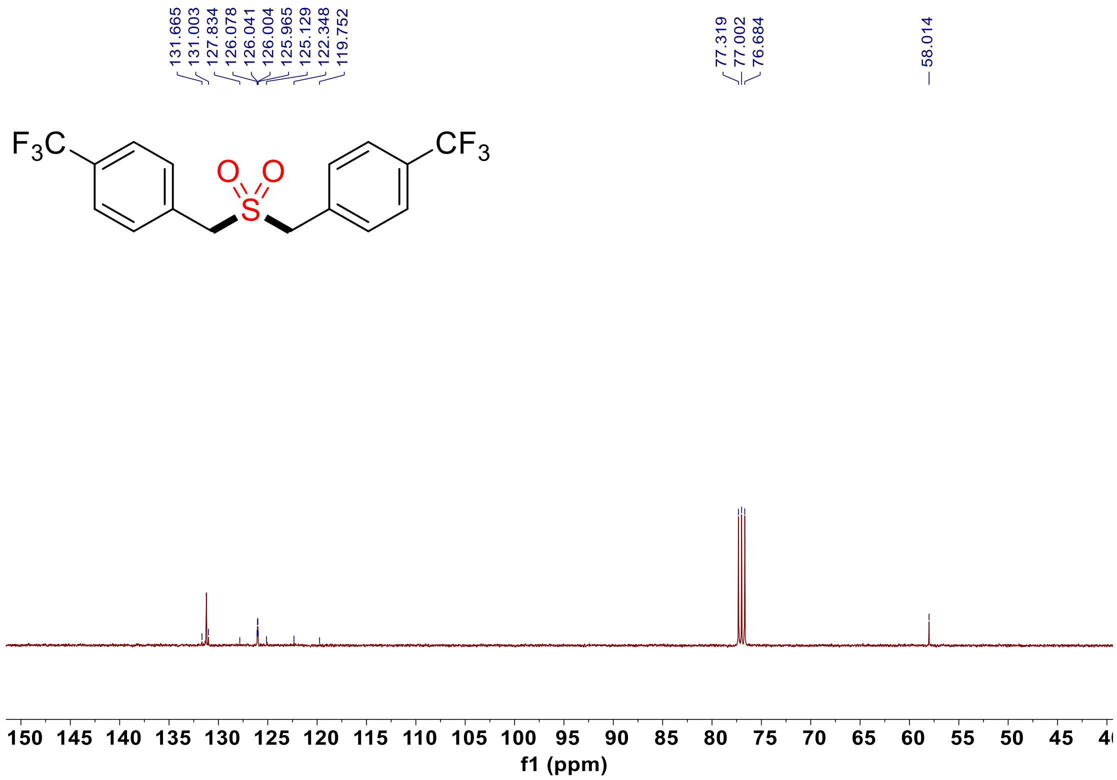

131.665
131.003
127.834
126.078
126.041
126.004
125.965
125.129
122.348
119.752
77.319
77.002
76.684
58.014
F3C
CF3
O
O
S
150 145 140 135 130 125 120 115 110 105 100 95 90 85 80 75 70 65 60 55 50 45
f1 (ppm)

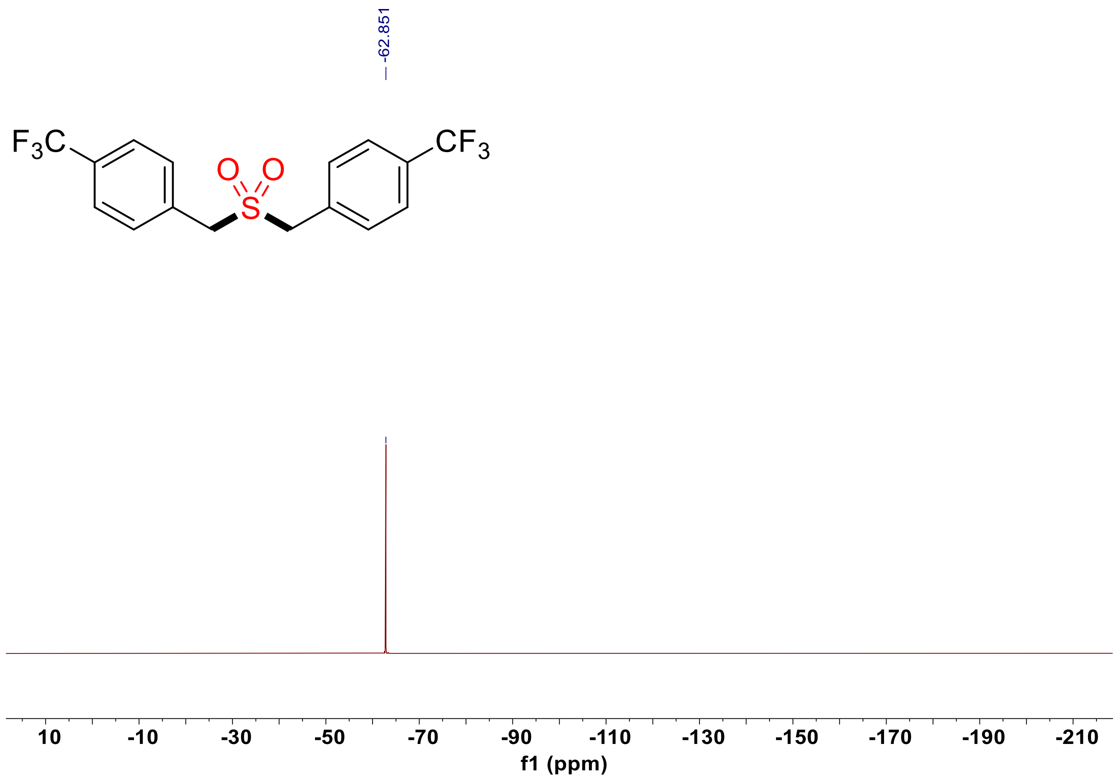

-62.851
F3C
CF3
O
O
S
10 -10 -30 -50 -70 -90 -110 -130 -150 -170 -190 -210
f1 (ppm)

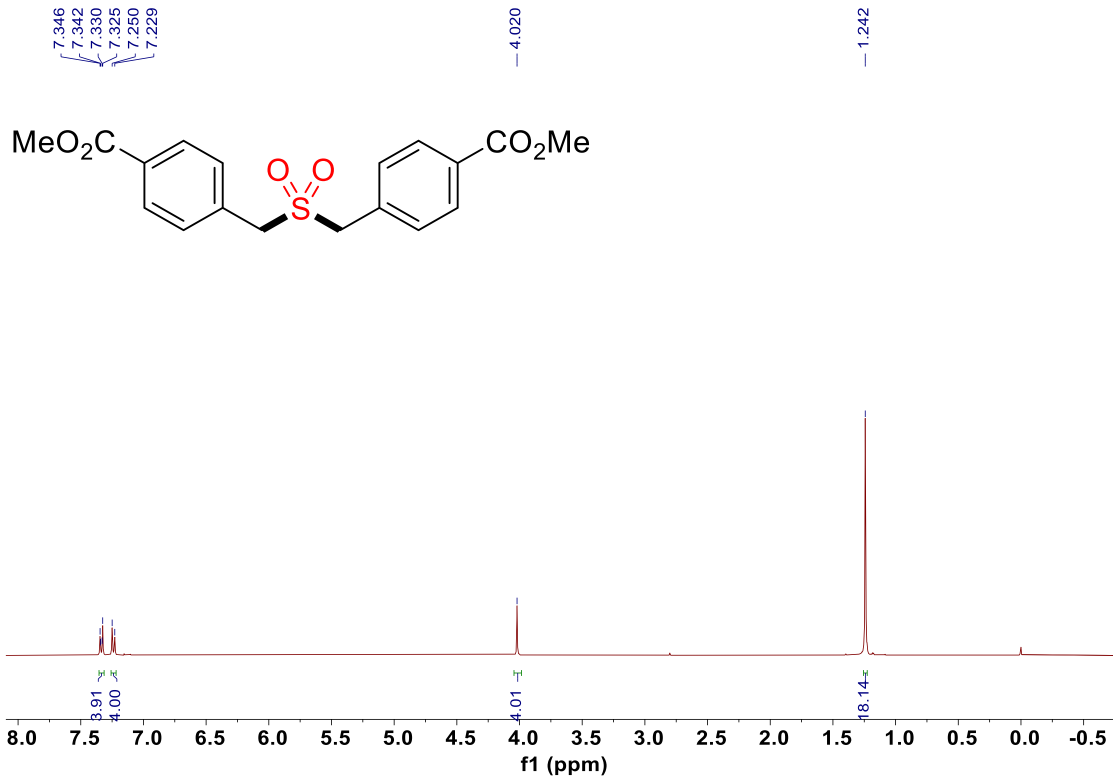

7.346
7.342
7.330
7.325
7.250
7.229
4.020
1.242
MeO2C
CO2Me
O
O
S
3.91
4.00
4.01
18.14
8.0 7.5 7.0 6.5 6.0 5.5 5.0 4.5 4.0 3.5 3.0 2.5 2.0 1.5 1.0 0.5 0.0 -0.5
f1 (ppm)

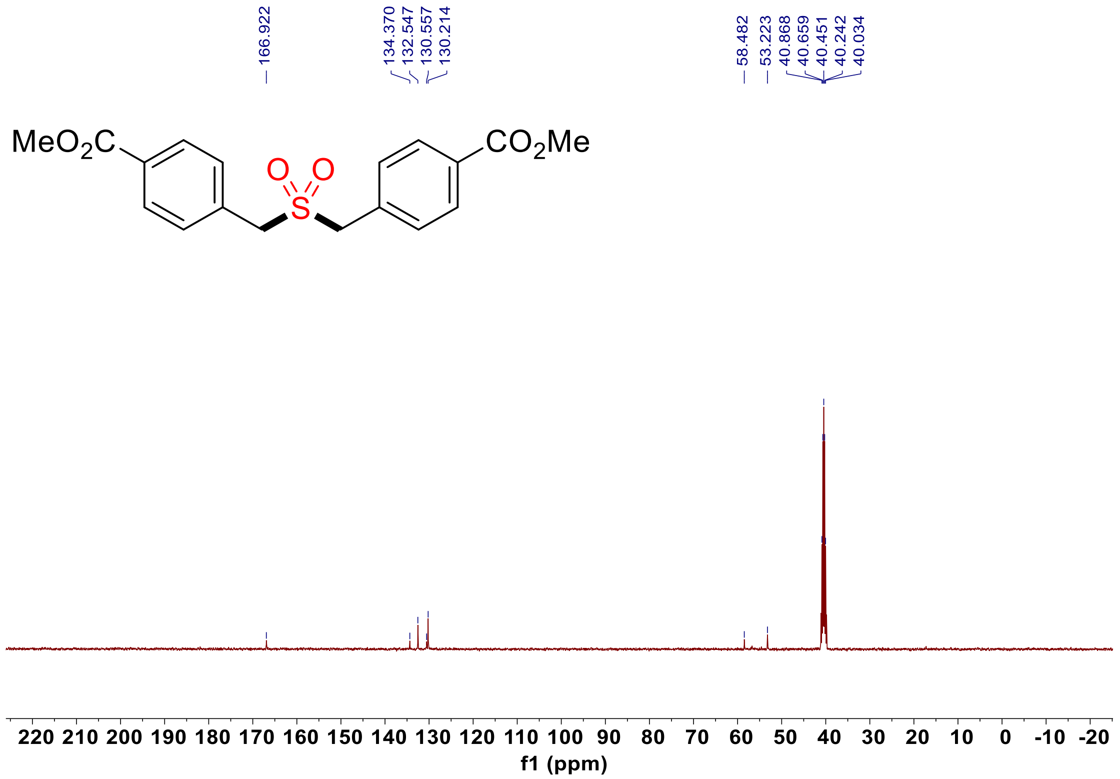

166.922
134.370
132.547
130.557
130.214
58.482
53.223
40.868
40.659
40.451
40.242
40.034
MeO2C
CO2Me
O
O
S
220 210 200 190 180 170 160 150 140 130 120 110 100 90 80 70 60 50 40 30 20 10 0 -10 -20
f1 (ppm)

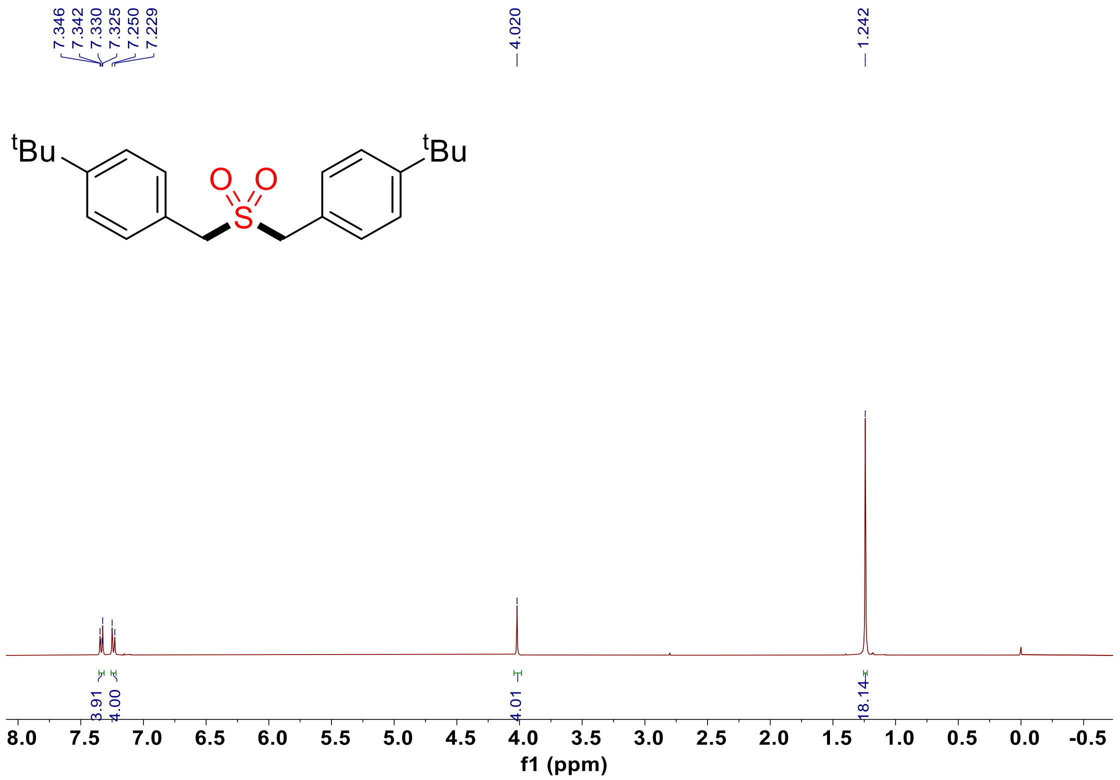

7.346
7.342
7.330
7.325
7.250
7.229
4.020
1.242
tBu
O
O
S
tBu
3.91
4.00
4.01
18.14
8.0 7.5 7.0 6.5 6.0 5.5 5.0 4.5 4.0 3.5 3.0 2.5 2.0 1.5 1.0 0.5 0.0 -0.5
f1 (ppm)

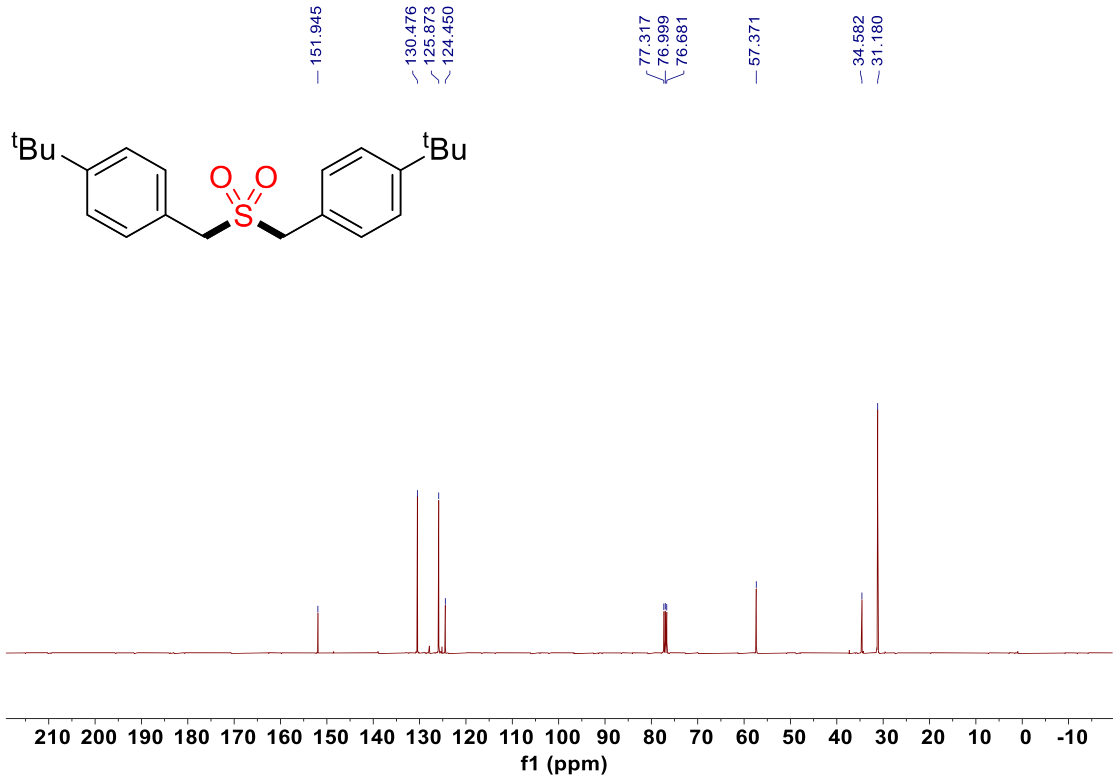

151.945
130.476
125.873
124.450
77.317
76.999
76.681
57.371
34.582
31.180
tBu
O
O
S
tBu
210 200 190 180 170 160 150 140 130 120 110 100 90 80 70 60 50 40 30 20 10 0 -10
f1 (ppm)

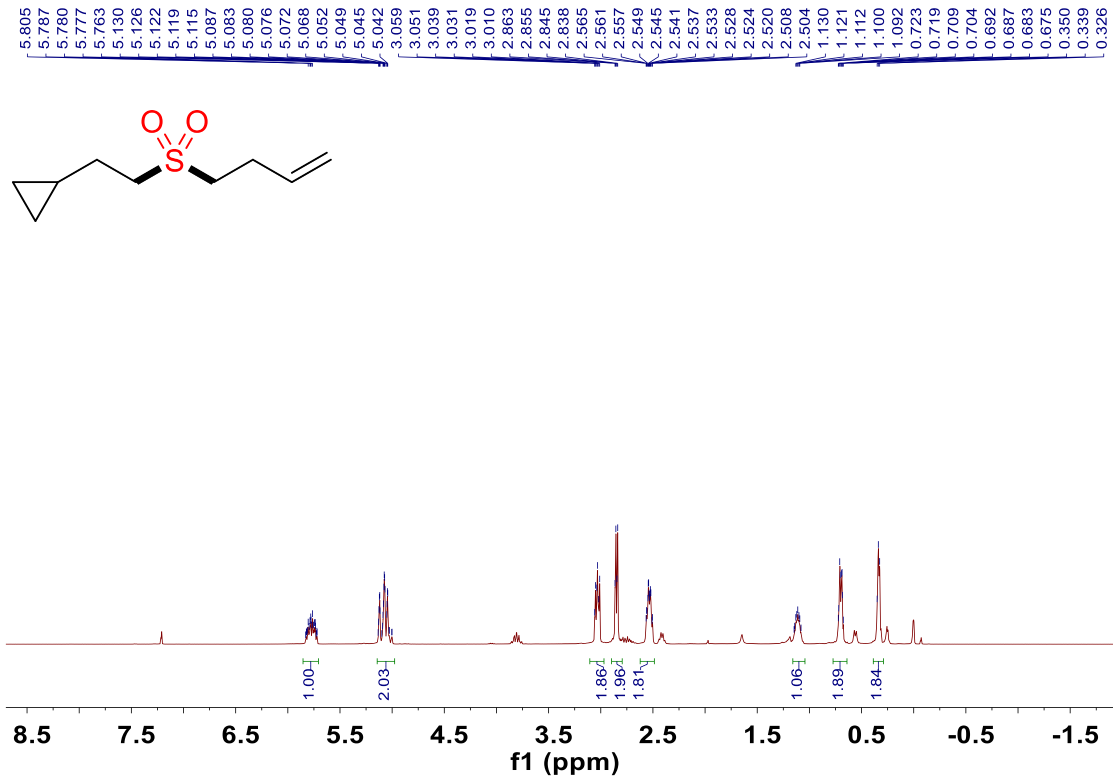
5.805
5.787
5.780
5.777
5.763
5.130
5.126
5.122
5.119
5.115
5.087
5.083
5.080
5.076
5.072
5.068
5.052
5.049
5.045
5.042
3.059
3.051
3.039
3.031
3.019
3.010
2.863
2.855
2.845
2.838
2.565
2.561
2.557
2.549
2.545
2.541
2.537
2.533
2.528
2.524
2.520
2.508
2.504
1.130
1.121
1.112
1.100
1.092
0.723
0.719
0.709
0.704
0.692
0.687
0.683
0.675
0.350
0.339
0.326
O
O
S
1.00
2.03
1.86
1.96
1.81
1.06
1.89
1.84
8.5
7.5
6.5
5.5
4.5
3.5
2.5
1.5
0.5
-0.5
-1.5
f1 (ppm)

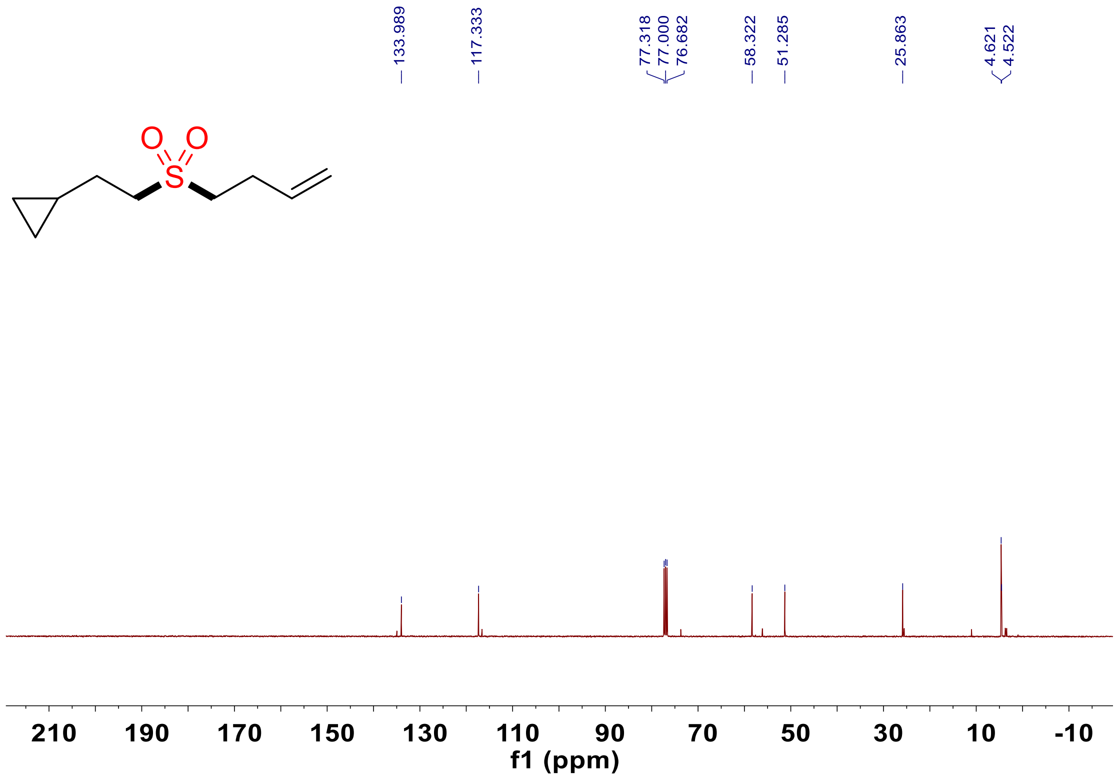
133.989
117.333
77.318
77.000
76.682
58.322
51.285
25.863
4.621
4.522
O
O
S
210
190
170
150
130
110
90
70
50
30
10
-10
f1 (ppm)

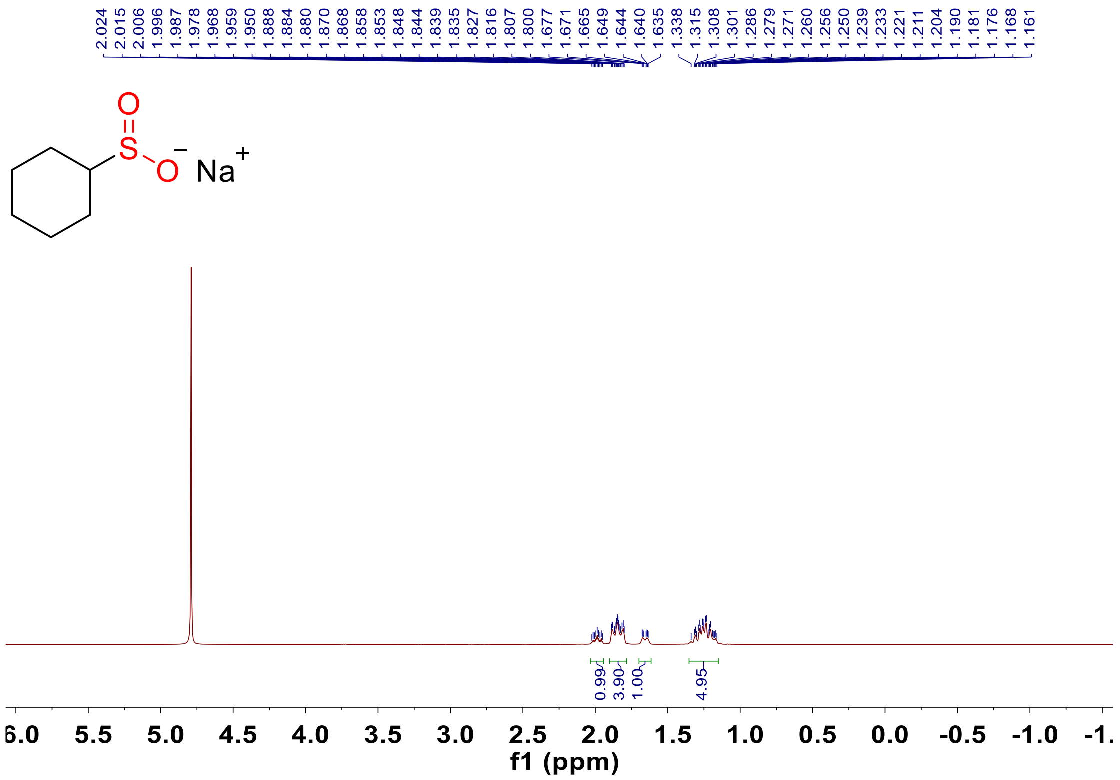
2.024
2.015
2.006
1.996
1.987
1.978
1.968
1.959
1.950
1.888
1.884
1.880
1.870
1.868
1.858
1.853
1.848
1.844
1.839
1.835
1.827
1.816
1.807
1.800
1.677
1.671
1.665
1.649
1.644
1.640
1.635
1.338
1.315
1.308
1.301
1.286
1.279
1.271
1.260
1.256
1.250
1.239
1.233
1.221
1.211
1.204
1.190
1.181
1.176
1.168
1.161
O
S
O
Na
0.99
3.90
1.00
4.95
6.0 5.5 5.0 4.5 4.0 3.5 3.0 2.5 2.0 1.5 1.0 0.5 0.0 -0.5 -1.0
f1 (ppm)

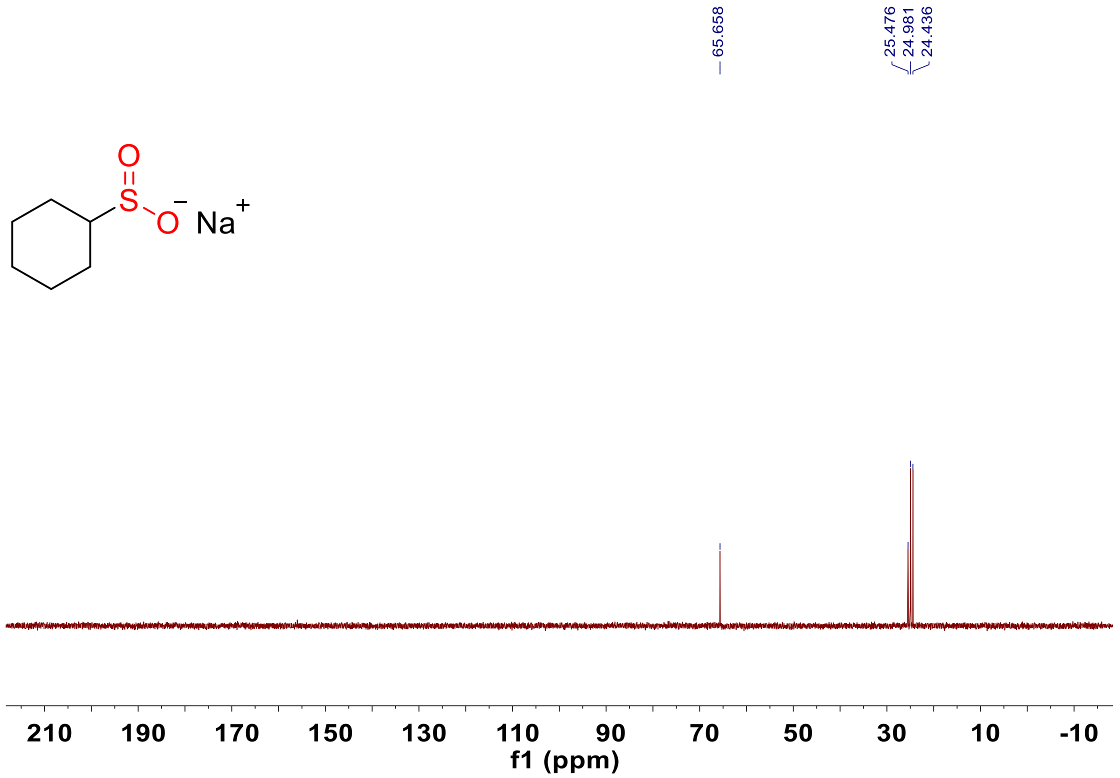
65.658
25.476
24.981
24.436
O
S
O
Na
210 190 170 150 130 110 90 70 50 30 10 -10
f1 (ppm)

References

[1] Y. Li, X. Yan, D. Chen, L. Shun, *Chinese Journal of Organic Chemistry* **2013**, *33*.

[2] R. L. Hudkins, N. C. Becknell, J. A. Lyons, L. D. Aimone, M. Olsen, R. C. Haltiwanger, J. R. Mathiasen, R. Raddatz, J. A. Gruner, *European Journal of Medicinal Chemistry* **2015**, *95*, 349-356.

[3] C. Gu, Z. Wang, R. Shi, *Chemical Communications* **2023**, *59*, 6889-6892.

[4] C. Nguyen, G. F. Ruda, A. Schipani, G. Kasinathan, I. Leal, A. Musso-Buendia, M. Kaiser, R. Brun, L. M. Ruiz-Pérez, B.-L. Sahlberg, N. G. Johansson, D. González-Pacanowska, I. H. Gilbert, *Journal of Medicinal Chemistry* **2006**, *49*, 4183-4195.

[5] L.-W. Xu, L. Li, C.-G. Xia, P.-Q. Zhao, *Tetrahedron Letters* **2004**, *45*, 2435-2438.

[6] T. A. Salama, S. S. Elmorsy, *Chinese Chemical Letters* **2011**.

[7] M. Guerfi, M. Berredjem, R. Bahadi, S.-E. Djouad, A. Bouzina, M. Aissaoui, *Journal of Molecular Structure* **2021**, *1236*.

[8] M. Leroux, T. Vorherr, I. Lewis, M. Schaefer, G. Koch, K. Karaghiosoff, P. Knochel, *Angewandte Chemie International Edition* **2019**, *58*, 8231-8234.

[9] H. Zhu, Y. Liu, Y. Zhang, L. Yang, J. Meng, Q. Li, B. Gong, Z. Xie, Z.-G. Le, *Molecular Catalysis* **2021**, *505*.

[10] R. A. Gaussian 16, M. J. Frisch, G. W. Trucks, H. B. Schlegel, G. E. Scuseria, M. A. Robb, J. R. Cheeseman, G. Scalmani, V. Barone, G. A. Petersson, H. Nakatsuji, X. Li, M. Caricato, A. V. Marenich, J. Bloino, B. G. Janesko, R. Gomperts, B. Mennucci, H. P. Hratchian, J. V. Ortiz, A. F. Izmaylov, J. L. Sonnenberg, D. Williams-Young, F. Ding, F. Lipparini, F. Egidi, J. Goings, B. Peng, A. Petrone, T. Henderson, D. Ranasinghe, V. G. Zakrzewski, J. Gao, N. Rega, G. Zheng, W. Liang, M. Hada, M. Ehara, K. Toyota, R. Fukuda, J. Hasegawa, M. Ishida, T. Nakajima, Y. Honda, O. Kitao, H. Nakai, T. Vreven, K. Throssell, J. A., Jr. Montgomery, J. E. Peralta, F. Ogliaro, M. J. Bearpark, J. J. Heyd, E. N. Brothers, K. N. Kudin, V. N. Staroverov, T. A. Keith, R. Kobayashi, J. Normand, K. Raghavachari, A. P. Rendell, J. C. Burant, S. S. Iyengar, J. Tomasi, M. Cossi, J. M. Millam, M. Klene, C. Adamo, R. Cammi, J. W. Ochterski, R. L. Martin, K. Morokuma, O. Farkas, J. B. Foresman and D. J. Fox, Gaussian, Inc., Wallingford CT, **2019.**

[11] a) A. D. Becke, *The Journal of Chemical Physics* **1993**, *98*, 5648-5652; b) S. Grimme, J. Antony, S. Ehrlich, H. Krieg, *The Journal of Chemical Physics* **2010**, *132*; c) C. Lee, W. Yang, R. G. Parr, *Physical Review B* **1988**, *37*, 785-789.

[12] Y. Zhao, D. G. Truhlar, *Theoretical Chemistry Accounts* **2007**, *120*, 215-241.

[13] A. V. Marenich, C. J. Cramer, D. G. Truhlar, *The Journal of Physical Chemistry B* **2009**, *113*, 6378-6396.

[14] b. C. Y. L. CYLview, **2009**.
